# Recent advances in functionalized electrospun nanofibers for biomedical and therapeutic applications: A comprehensive review

**DOI:** 10.1016/j.bioactmat.2026.10.009

**Published:** 2026-10-22

**Authors:** Kalim Deshmukh, Mayank Pandey, Hichem Moulahoum, Faezeh Ghorbanizamani, Tomáš Kovářík, Agata Przekora-Kuśmierz, Hemalata Jena, Miral Vora, Gautam Patel, Priyabrat Pradhan

**Affiliations:** a New Technologies Research Centre, University of West Bohemia in Pilsen, 30100, Pilsen, Czech Republic; b Department of Physical Sciences, Kristu Jayanti (Deemed to be University), Bangalore, 560077, Karnataka, India; c Department of Biochemistry, Faculty of Science, Ege University, Izmir, 35100, Türkiye; d Department of Material Science and Metallurgy, Faculty of Mechanical Engineering, University of West Bohemia in Pilsen, 30100, Pilsen, Czech Republic; e Institute of Pharmaceutical Sciences, Faculty of Medicine, KUL-The John Paul II Catholic University of Lublin, 20708, Poland; f School of Mechanical Engineering, Kalinga Institute of Industrial Technology (KIIT) Deemed to be University, Patia, Bhubaneswar, Odisha, 751024, India; g Department of Chemistry, Krishna School of Science, Drs. Kiran and Pallavi Patel Global University (KPGU), Varnama, Vadodara, Gujarat, 391243, India; h Department of Chemical Sciences, Parul Institute of Applied Sciences, Faculty of Applied Sciences, Parul University, Waghodia, Vadodara, Gujarat, 391760, India; i Department of Mechanical Engineering, Synergy Institute of Engineering and Technology, Dhenkanal, Odisha, 759001, India

**Keywords:** Electrospinning, Electrospun nanofibers, Functionalization, Biocompatibility, Biodegradability, Biomedical applications

## Abstract

Electrospun nanofibers (ESNFs) have become a major focus of modern research given their adaptable architecture, extensive interface, and distinctive physicochemical characteristics, making them appropriate for varied applications such as catalysis and environmental cleanup, drug delivery (DD), tissue engineering (TE), sensing, actuation, and various energy-related technologies. As a result, electrospinning (ES) has emerged as a versatile manufacturing technique, driving intensive efforts to engineer nanofibrous structures specifically for biomedical purposes. Functionalization of ESNFs can be achieved by embedding functional agents or altering surface chemistry to introduce specific functionalities. This review discusses the fabrication methods, optimization techniques, and broad biomedical applications of modified ESNFs, emphasizing their key properties, including biocompatibility, degradability, and safety profile. The article outlines various strategies for creating and modifying ESNFs, followed by a comprehensive analysis of their applications in tissue regeneration, DD, wound repair, antimicrobial therapy, cancer diagnostics and treatment, and biosensing. By critically evaluating recent advances, highlighting future directions, and addressing ongoing challenges about ES, this review underscores the transformative potential of functionalized ESNFs as efficient and adaptable biomaterials for biomedical technologies.

## Highlights

• Discusses Fabrication Techniques of Electrospun Nanofibers.
• Discusses Surface Chemistry and Functionalization of Electrospun Nanofibers.
• Reviewing Various Biomedical Applications of Functionalized Electrospun Nanofibers.
• Addresses Challenges and Highlights Future Directions for Using Functionalized Electrospun Nanofibers in Biomedical Field.

## Graphical abstract

**Figure:**
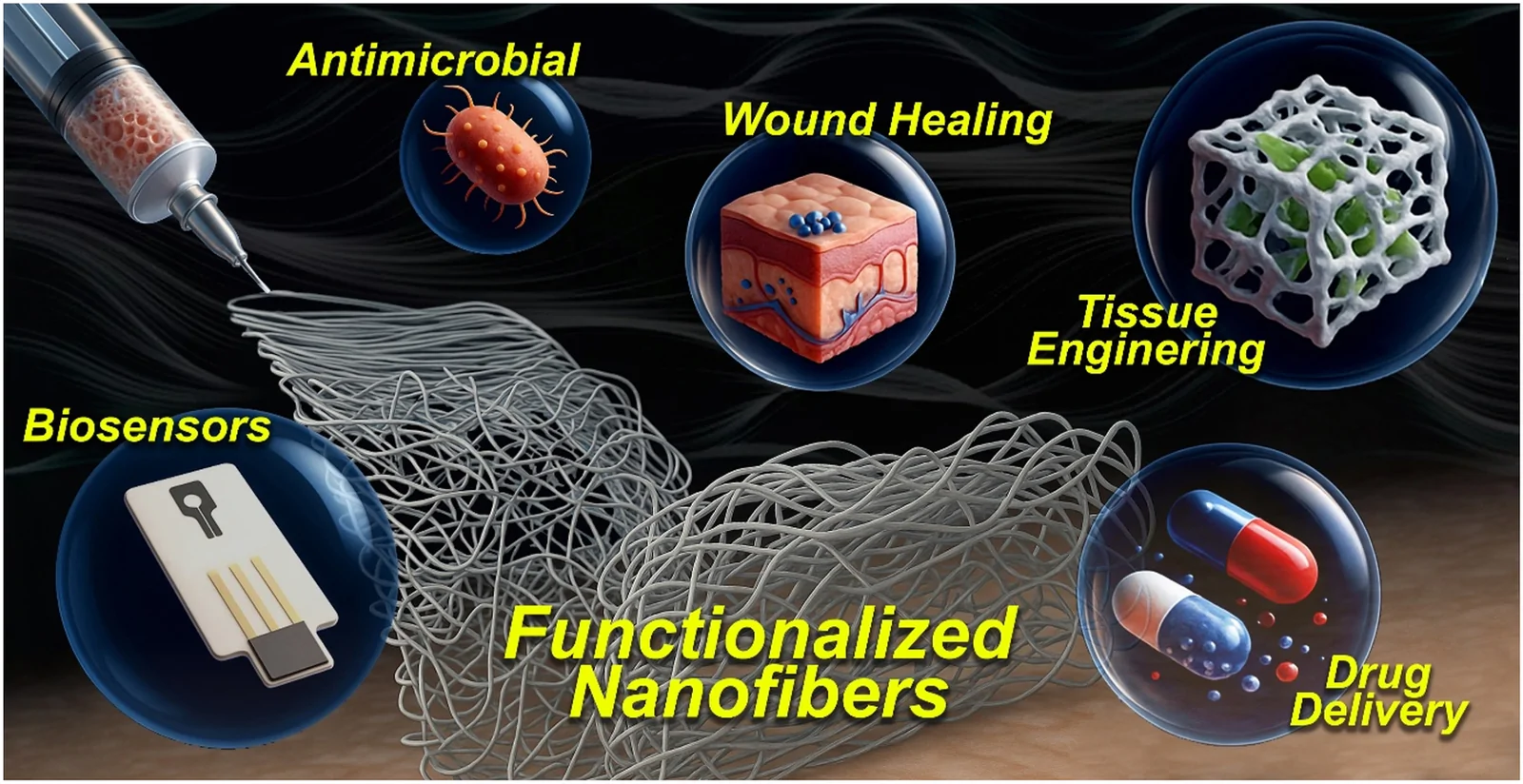

## Abbreviations:

NFs: Nanofibers
ES: Electrospinning
NPs: Nanoparticles
3D: Three-dimensional
ESNFs: Electrospun nanofibers
TE: Tissue engineering
DD: Drug delivery
THF: Tetrahydrofuran
DMF: Dimethylformamide
PLLA: Poly (L-lactic acid)
PCL: Polycaprolactone
PLGA: Poly lactic-co-glycolic acid
2D: Two-dimensional
NLES: Needleless electrospinning
ECM: Extracellular matrix
DMSO: Dimethyl sulfoxide
PU: Polyurethane
AC: Alternating current
ATR-FTIR: Attenuated Total Reflectance Fourier Transform Infrared
XPS: X-ray photoelectron spectroscopy
DSC: Differential Scanning Calorimetry
XRD: X-Ray Diffraction
SEM: Scanning electron microscopy
PAMAM: Polyamidoamine
HCl: Hydrochloric acid
H_2_SO_4_: Sulfuric acid
HClO_4_: Perchloric acid
PGA: Polyglycolic acid
CNCs: Cellulose nanocrystals
STE: Skin tissue engineering
LFAs: Lateral flow assays
LOD: Limit of detection
NFMs: Nanofiber membranes
EDAC: 1-ethyl-3-(3-dimethylaminopropyl) carbodiimide hydrochloride
ATRP: Atom transfer radical polymerization
CFRP: Controlled free-radical polymerization
APPJ: Atmospheric-pressure plasma jet
APTES: 3-aminopropyltriethoxysilane
PMPC: Poly (2-methacryloyloxyethyl phosphorylcholine)
CS: Chitosan
DBCO: Dibenzocyclooctyne
BMP-2: Bone morphogenetic protein-2
AAm: Acrylamide
APm: N-(3-aminopropyl) methacrylamide
GDMA: glycerol dimethacrylate
OA: Oleylamine
BTE: Bone tissue engineering
HRP: Horseradish peroxidase
DOX: Doxorubicin
ICA: Icariin
PVA: Polyvinyl alcohol
TAIC: Triallyl isocyanurate
CaP: Calcium phosphate
CG: Cationized gelatin
CTE: Cartilage tissue engineering
sGAG: Sulfated glycosaminoglycans
PEO: Polyethylene oxide
SBF: Simulated body fluid
RF: Radio-frequency
PBT: Polybutylene terephthalate
PEOT: Polyethylene oxide terephthalate
BSA: Bovine serum albumin
hMSCs: Human mesenchymal stem cells
PDA: Polydopamine
LPS: Lipopolysaccharide
MSCs: Mesenchymal stem cells
ALP: Alkaline phosphatase
ASP: Aspartic acid
GLU: Glutamic acid
OC: Osteocalcin
OP: Osteopontin
Col-I: Collagen type I
UV: Ultraviolet
SF: Silk fibroin
LMWH: Low-molecular-weight heparin
PVP-I: Poly (vinyl pyrrolidone)-iodine
TPU: Thermoplastic polyurethane
HUVECs: Human umbilical vein endothelial cells
VTE: Vascular tissue engineering
LBL: Layer-by-layer
QC: Quaternized chitin
TA: Tannic acid
nHAp: Nano-hydroxyapatite
MNPs: Magnetic nanoparticles
VEGF: Vascular endothelial growth factor
FGF: Fibroblast growth factor
DAPI: 4′,6-diamidino-2-phenylindole
CLSM: Confocal laser scanning microscopy
EFMs: Electrospun fiber mats
CNFs: Cellulose NFs
TCPS: Tissue culture polystyrene
CjECs: Conjunctival epithelial cells
CECs: Corneal epithelial cells
HPMC: Hydroxypropyl methylcellulose
TEBVs: Tissue-engineered blood vessels
PLCL: Poly(L-lactide-co-ε-caprolactone)
TCP: Tissue culture plastic
OS: Osteosarcoma
GBR: Guided bone regeneration
GAG: Glycosaminoglycan
DA: Dopamine
PCLEEP: Poly (ε-caprolactone-co-ethyl ethylene phosphate)
TEMPO: 2,2,6,6-tetramethylpiperidine 1-oxyl
sGAG: Sulfated glycosaminoglycan
GelMA: Gelatin methacryloyl
TCS: Thioglycolic acid-modified chitosan
ddECMMA: Methacrylated decellularized dermal extracellular matrix
2-MIM: 2-methylimidazole
CPT: Camptothecin
APS: Artemisia polysaccharide
CAP: capsaicin
QU: Quercetin
ROS: Reactive oxygen species
α-SMA: α-smooth muscle actin
MRSA: Methicillin-resistant *S. aureus*
BCM: Block copolymer micelle
NHDFs: Normal human dermal fibroblasts
CSNM: Curcumin-loaded sandwich-like NF membrane
HG: High-glucose
GEL: Gelatin
MOFs: Metal-organic frameworks
MIC: Minimum inhibitory concentration
AgNPs: Silver NPs
PAN: Polyacrylonitrile
PVDF: Polyvinylidenefluoride
WSCP: Poly[(dimethylimino)(2-hydroxy-1,3-propanedily) Chloride]
DBNPA: 2,2-dibromo-3-nitrilopropionamide
TCMTB: (2-(thiocyanomethylthio)benzothiazole)
BN: Boron nitride
ZOI: Zone-of-inhibition
CFU: Colony forming units
TSB: Tryptic soy broth
PHMBG: Polyhexamethylene biguanidine
FUC: Fucoidan
COR: Cordycepin
OHDA: Dopamine-modified hyaluronic acid
Ag-TiO_2_: Silver-doped titanium dioxide
EpCAM: Epithelial-cell adhesion molecule
DCA: Dichloroacetic acid
SPCA: Simultaneously photo-cleavable and activatable
CTCs: Circulating tumor cells
PNIPAAm: Poly(N-isopropylacrylamide)
WBCs: White blood cells
RT-qPCR: Real-time polymerase chain reaction
AMF: Alternating magnetic field
TUNEL: Terminal deoxynucleotidyl transferase-mediated dUTP nick end labelling
CMCs: Circulating melanoma cells
LMD: Laser microdissection
NHS: N-hydroxysuccinimide
PDMS: Polydimethylsiloxane
ICC: Immunocytochemistry
DMEM: Dulbecco's Modified Eagle Medium
EPR: Enhanced permeability and retention
RES: Reticuloendothelial system
PIP: Piperine
HAADF-STEM: High-angle annular dark-field scanning transmission electron mcroscopy
BCL-2: B-cell lymphoma 2
GAPDH: Glyceraldehyde 3-phosphate dehydrogenase)
SPCE: Carbon screen-printed electrode
ZIF-8: Zeolitic imidazolate framework-8
LDH: Layered double hydroxide
β-CD: β-cyclodextrin
CV: Cyclic voltammetry
BTCA: 1,2,3,4-butanetetracarboxylic acid
ChEt: Cholesterol esterase
ChOx: Cholesterol oxidase
ITO: Indium tin oxide
AA: Ascorbic acid
UA: Uric acid
RSD: Relative standard deviation
GMCFs: Graphene/metal oxide composite microfibers
GCE: Glassy carbon electrode
Mb: Myoglobin
ICP: Inductively coupled plasma
OLC: Onion-like carbon
PPC: Polypropylene carbonate
DPV: Differential pulse voltammetry
MIPs: Molecularly imprinted polymers
PEC: Photoelectrochemical
TPhP: Triphenyl phosphate
OPFRs: Organophosphate flame retardants
FMICPs: Fluorescent molecularly imprinted conjugated polythiophenes
AFP: Alpha-fetoprotein
CEA: Carcinoembryonic antigen
p-HAP: p-hydroxyacetophenone
APAP: Acetaminophen
CPE: Carbon paste electrode
MB: Methylene blue
PASP: Polyaspartate
CA: Cellulose acetate
DET: Direct electron transfer
TSP53: Tumor suppressor protein p53
ECL: Electrochemiluminescent
C_Ab_: Capture antibodies
D_Ab_: Detection antibodies
H_2_O_2_: Hydrogen peroxide
HQ: Hydroquinone
BCL: *Burkholderia cepacia* lipase
MONFs: Metal-organic NFs
Vit-D_3_: Vitamin D_3_
EGF: Electrospun graphite fibers
GMP: Good Manufacturing Practice

## 1 Introduction

Nanotechnology has rapidly evolved into a highly impactful research field with significant potential for advancements in healthcare and biomedical sciences. In particular, polymeric micro- and nanofibers (NFs) have attracted increasing attention in biomedical engineering as key building blocks for devices and functional biomaterials [1]. Among various fabrication techniques, electrospinning (ES) has become a leading method for nanofiber production due to its wide applicability in pharmaceuticals and medicine. Through electrostatic interactions, a viscoelastic polymer solution is drawn into continuous fibers, enabling the fabrication of nanoscale structures in a simple and versatile manner [2]. ES is widely valued for its operational simplicity, scalability, and the ability to finely tune fiber diameter, composition, and orientation [3]. ES permits the fabrication of fibers of diameters ranging from the micrometer-to-nanometer scale, utilizing a wide spectrum of materials, including polymers of natural or synthetic origin, ceramics, small molecules, and hybrid systems [2]. In addition, ES allows the fabrication of specialized structures, including porous, hollow, and core-sheath fibers. These structures could be further functionalized by incorporating bioactive agents or nanoparticles (NPs) during or after spinning. Beyond single fibers, more complex architectures, including aligned, layered, and three-dimensional (3D) assemblies, can be achieved through controlled deposition strategies [2], [3], [4], [5]. Owing to these versatile features, electrospun nanofibers (ESNFs) have been widely applied in filtration, catalysis, environmental protection, energy systems, and functional textiles. In biomedical applications, they are particularly important for tissue engineering (TE), wound healing, drug delivery (DD), and regenerative therapies [2].

The development of ES spans several centuries. Early observations of electrostatic effects on liquids were reported by William Gilbert in 1600 [6], [7]. In the 18th century, Stephen Gray and Abbe Nollet described electrostatically driven fluid jets and electrospraying phenomena. A major step toward modern ES occurred in 1902 with Cooley and Morton, who developed early device concepts [7]. In 1914, John Zeleny analyzed fluid behavior under the influence of electric fields using mathematical models. Geoffrey Taylor, in work conducted between 1964 and 1969, provided the theoretical basis for jet formation by describing the “Taylor cone.” Despite these advances, the field saw limited progress for several decades until its revival in 1990 by Darrell Reneker and Gregory Rutledge, who demonstrated the polymer-based fiber production and significantly expanded its application potential [7], [8]. An illustrative overview of this historical development is shown in Fig. 1.

**Fig. 1:**
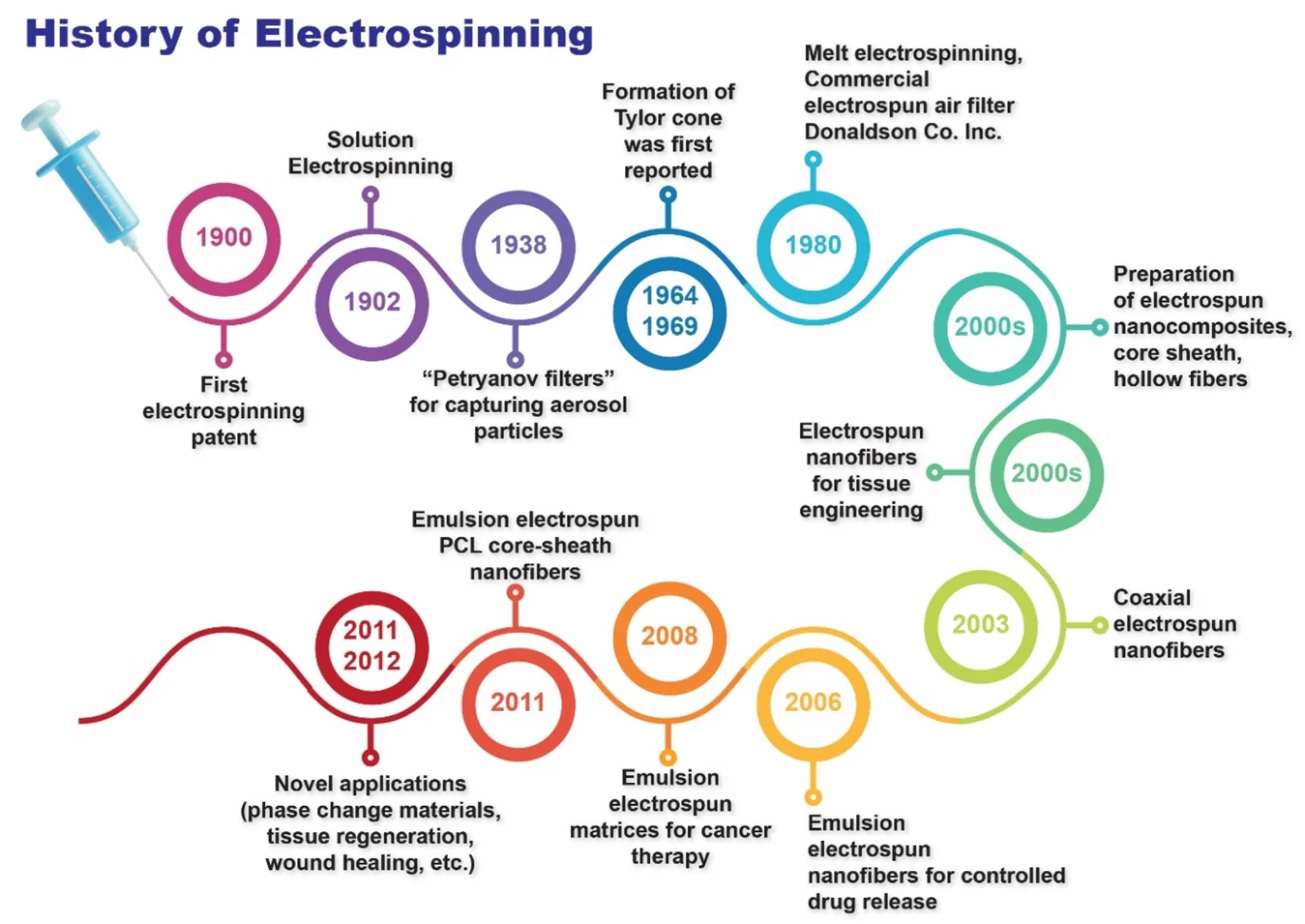
An overview of historical development and timeline of ES process.

Over time, ES has been exploited with a broad range of natural and synthetic polymers for the fabrication of NFs, including nanocomposites and core-sheath structures. Despite its strengths, ES faces several limitations. A key challenge is the reliance on large amounts of organic or hazardous solvents, especially when processing hydrophobic polymers. Additional issues include difficulties in processing very low- or high-viscosity solutions, limited throughput, and challenges in controlling fiber alignment and uniformity [9]. Common diluents like tetrahydrofuran (THF), dimethylformamide (DMF), and other benzene derivatives raise environmental and safety concerns. Their use limits the integration of sensitive biomolecules like proteins and living cells, thereby restricting their applications in TE. Moreover, residual solvents in the final fibers may impair bioactivity and trigger inflammatory responses in biological systems [10]. These constraints emphasize the need to develop greener and more biologically compatible alternatives. Water-based ES of hydrophilic polymers like PEG, PVA, and PEO offers a promising approach, while solvent blending can further improve spinnability [5], [11], [12], [13]. In contrast, hydrophobic polymers like poly (L-lactic acid) (PLLA), polycaprolactone (PCL), poly lactic-co-glycolic acid (PLGA), and PAN typically require organic solvents [5], [14], [15], [16]. Efforts are ongoing to optimize ES conditions to produce high-quality fibers suitable for commercial applications [15]. Consequently, the field has experienced rapid advancement, leading to increasingly sophisticated fiber designs and a large range of processing approaches. These developments have led to new ES systems that have significantly expanded the capabilities and impact of this technology.

A standard ES system consists of distinct components including a polymeric solution, a power supply, a spinneret, and a grounded collector [17]. The polymeric solution is delivered through a syringe and subjected to a high electric field, which induces charge accumulation at the needle tip. The resulting electrostatic forces stretch the solution into a jet pointed to the collector [18]. As the jet moves, it elongates and undergoes rapid solvent evaporation, forming solid NFs [17], [19], [20]. Fiber formation is governed by multiple interdependent parameters, including solution properties, process conditions, and environmental factors [21]. Applied voltage typically ranges from a few to 30 kV and influences jet stretching and fiber diameter, with higher voltages generally producing thinner fibers [17], [22]. However, fiber morphology is also strongly affected by viscosity, surface tension, conductivity, and solvent volatility. High viscosity resists elongation and leads to thicker fibers, while insufficient viscosity may cause droplets to form instead of fibers. Beyond viscosity, the solution's conductivity further affects jet stability and fiber morphology [17], [23], [24]. Because these parameters interact nonlinearly, achieving consistent fiber characteristics remains challenging, particularly for scale-up applications [25].

ES parameters are usually divided into three categories: solution properties, operational conditions, and environmental factors [26]. Solution-related variables like polymer concentration, conductivity, surface tension, molecular weight, viscosity, and solvent volatility determine jet stability and fiber formation. Operational parameters like applied voltage, flow rate, and the distance between the collector and tip should be adjusted based on solution-specific characteristics to ensure uniform fibers [27]. Environmental variables, particularly heat and moisture, further affect solvent evaporation and fiber conformation. For example, a stretched working distance extends the time available for jet stretching and solvent evaporation, often producing finer fibers. At the same time, inadequate volatility or low temperature can lead to incomplete drying and fiber fusion. However, excessive distance may destabilize the Taylor cone and reduce uniformity [26], [27]. Overall, ES is governed by a complex interplay of interdependent variables that are difficult to optimize independently. This nonlinear behavior poses a significant challenge for reproducibility, process standardization, and industrial scale-up [26], [27], [28].

## 2 Classifications of electrospinning techniques

The ES process is capable of producing a broad spectrum of nanofiber morphologies, including smooth, branched, core-sheath, porous, ribbon-like, and even fractal-like structures [1], [29]. Over the last twenty years, numerous ES techniques have been introduced to meet diverse application and structural requirements [29]. The growing industrial relevance of ES has driven the establishment of large-scale production systems, making both the technology and nanofibrous products increasingly commercially viable [1], [17]. As a result, a selection of procedures has been recognized to fabricate two-dimensional (2D) and 3D fibrous constructs for specific functional applications. Currently, ES systems are broadly classified into needle-based and needleless approaches [25], [30]. The first ES (needle-based) technique is the most commonly used, in which the polymer is dispensed via a syringe to form fibers. It offers accurate control of fiber structure but often suffers from clogging, particularly when processing highly viscous or particle-loaded solutions [25], [31], [32], [33]. Variants of this approach include side-by-side, blend, and multiaxial (coaxial and triaxial) ES, as shown in Fig. 2 [25]. Needleless electrospinning (NLES) eliminates the use of needles by generating fibers from liquid surfaces or rotating elements such as disks or wires. This approach reduces clogging, increases production rates, and enables the formation of complex fiber morphologies. It also allows management of critical properties such as fiber diameter, porosity, and interface chemistry [25], [34], [35], [36]. However, variations in morphology and mechanical properties are often observed due to differences in materials, solvents, and processing conditions [25], [37]. These tunable features are particularly valuable for biomedical applications, as they influence drug release behavior, targeting efficiency, and tissue compatibility. Consequently, ESNFs are progressively employed in controlled DD, targeted therapy, and TE frameworks [38].

**Fig. 2:**
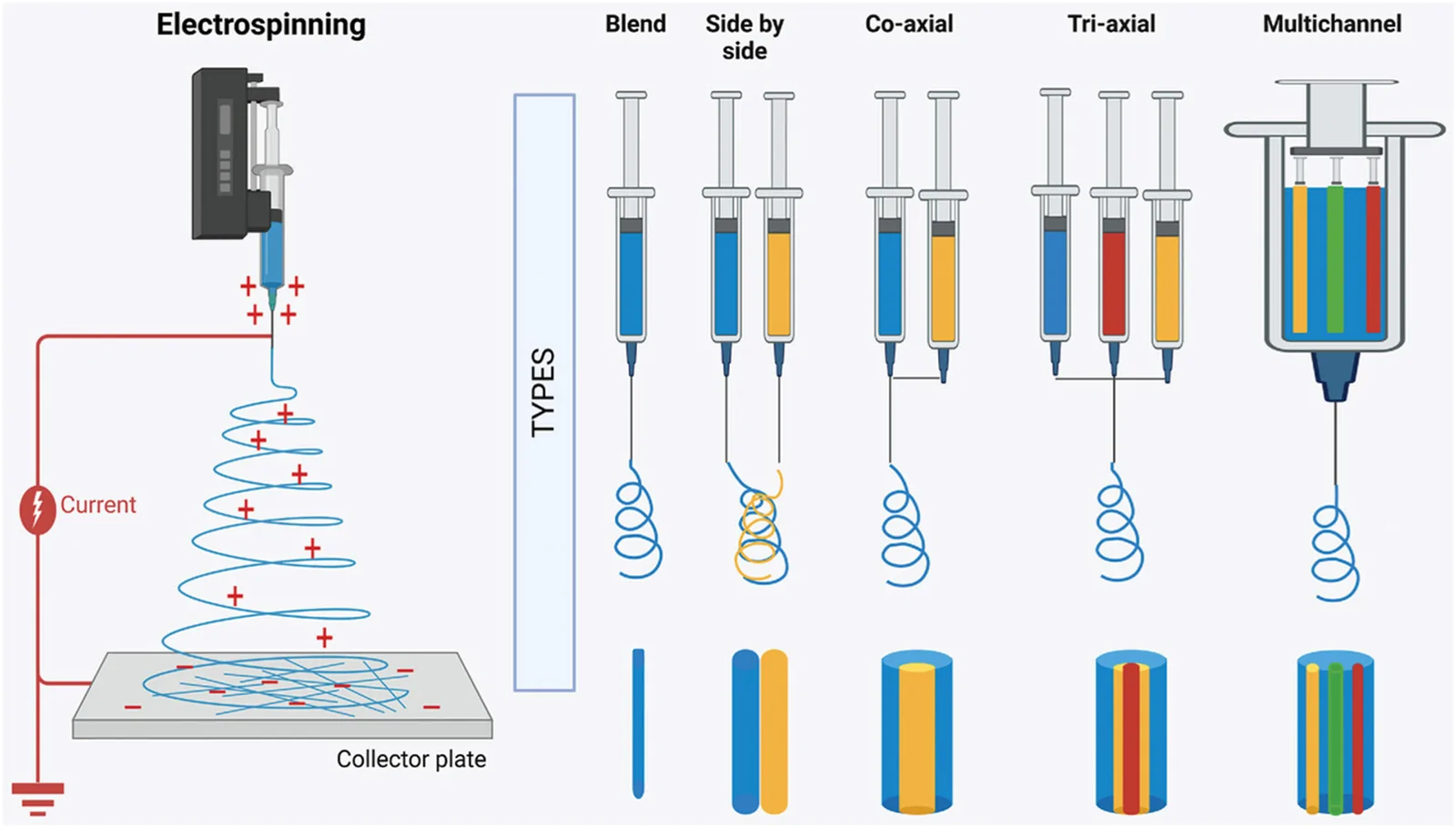
Demonstration of various ES configurations [25]. Copyright 2025. Reproduced with permission from John Wiley & Sons.

Needle-based ES has provided ample promise in DD and TE applications. ESNFs mimic the structure of the natural extracellular matrix (ECM) and work as effective scaffolds that facilitate cell adhesion, proliferation, and differentiation. They can also be functionalized with biomolecules like cytokines, growth factors, or genetic material to further enhance their regenerative capacity [8], [25]. In bone and cartilage engineering, electrospun structures provide mechanical reinforcement whilst delivering osteoinductive cues or stem cells to stimulate repair [39]. For wound healing, NFs containing antibacterial compounds or growth factors help maintain moisture and enable controlled drug release, thereby accelerating healing and reducing infection risk. Needle-based ES has also emerged as a promising platform for vaccine delivery [40]. Encapsulation of antigens or adjuvants enables sustained release and enhanced immune responses, with applications in DNA and protein vaccines where fibers act as both delivery vehicles and stabilizing matrices [41]. For infection control, ESNFs incorporating antimicrobial molecules like silver NPs, antibiotics, and antifungals have been developed for wound dressings and implant coatings, providing prolonged antimicrobial protection while supporting tissue regeneration [42]. Overall, needle-based ES offers a versatile tool for modern DD strategies and biomedical instruments. By tuning processing conditions and fiber architecture, it is possible to control drug release profiles, protect sensitive therapeutics, and enable targeted delivery [43]. Continued advances in materials and processing are expected to further expand its applications toward more effective and personalized medical solutions [25].

### 2.1 Needle based electrospinning techniques

#### 2.1.1 Blend electrospinning

Blend ES represents one of the simplest and most frequently used strategies for DD, in which both the polymer and the therapeutic compound are mixed in the same solvent solution [44]. This methodology enables the molecule of interest to be evenly distributed and integrated into the polymer matrix during fiber formation. Its effectiveness strongly depends on drug-polymer compatibility, following the “like dissolves like” principle. High compatibility promotes homogeneous drug distribution and sustained release, whereas incompatibility often triggers burst release due to rapid drug expulsion, particularly when hydrophilic drugs and hydrophobic polymers are involved [45]. In biodegradable systems, release mechanisms usually involve diffusion, polymer degradation, or a combination thereof, while in non-biodegradable systems, it is mainly diffusion-controlled [46]. Despite its simplicity, blend ES faces limitations, especially for sensitive biomolecules such as proteins, which may be denatured or damaged when exposed to organic solvents. In addition, polymers with high hydrophobicity often exhibit slow release profiles, making them less suitable for rapid delivery applications [47]. To address compatibility issues, co-solvents like dimethyl sulfoxide (DMSO) have been used to better drug dispersion and achieve more controlled release behavior [48]. Blend ES has been effectively harnessed for a large selection of therapeutics, including antibiotics [49], cytostatic agents [50], anti-inflammatory drugs [51], and antimicrobial peptides [52], [53]. However, its use for protein delivery remains restricted due to the denaturing effects of organic solvents and most commonly used polymers such as PCL, PLGA, and polyurethane (PU) require them, which can induce protein unfolding and loss of bioactivity [54].

#### 2.1.2 Side-by-side electrospinning

Side-by-side ES is a specialized technique that employs dual parallel nozzles to process two polymer solutions simultaneously, producing multicomponent NFs with distinct compartments. This approach enhances fabrication flexibility and enables the formation of complex fiber architectures with tailored properties in a single step, with applications in biomedical devices, sensors, and advanced functional materials [55]. By tuning spinneret configuration and process parameters, fibers with controlled dimensions and interfacial characteristics can be obtained [56]. In this Janus fiber configuration, two distinct solutions are co-electrospun to form adjacent but separated compartments within a single fiber [57]. A notable benefit of this system is its ability to enable biphasic drug release, where one compartment provides an initial burst release while the other ensures sustained delivery [25]. However, several challenges limit process stability. Interfacial instability between the two phases, differences in solvent evaporation rates, and phase separation can lead to morphological irregularities and altered release behavior [58]. In addition, precise coordination of fluid flow, electrostatic forces, and rheological properties is required to ensure synchronized jet formation from both nozzles [59]. These instabilities make it more difficult to achieve uniform fibers compared with blend ES, although advances in polymer compatibility and solvent selection are gradually improving reproducibility [60].

#### 2.1.3 Coaxial electrospinning

Coaxial ES is a highly versatile process for producing complex nanofibrous structures with controlled core-sheath architectures, offering enhanced structural and functional design flexibility [61]. It was introduced by Sun et al., in 2003, aiming to overcome conventional blend ES challenges by enabling the fabrication of fibers with distinct internal compartments [62]. This technique involves concurrent electrospinning of multiple polymer solutions via a coaxial spinneret consisting of concentric capillaries. The internal nozzle introduces the core solution, whereas the external nozzle supplies the polymer that forms the shell [25], [26], [62], [63], [64], [65]. During processing, the shell solution encapsulates the core at the spinneret tip, forming a composite Taylor cone under an electric field; subsequently, the solvent evaporation yields continuous fibers with well-defined core-sheath structures [26]. The final fiber properties depend strongly on the interaction between the two solutions, including viscosity, miscibility, conductivity, and interfacial tension. Stable core-sheath formation requires careful control of these parameters and maintenance of a stable Taylor cone throughout the process [26]. Typically, the shell mixture must exhibit higher viscosity than the core to ensure complete encapsulation, while flow rates must be carefully balanced to maintain structural integrity [65]. In addition, the two fluids are ideally immiscible or only partially miscible; otherwise, phase mixing, premature solidification, or structural instability may occur. Interfacial shear forces between core and shell phases also influence fiber morphology and stability [65], [66]. These factors make coaxial ES more complex than single-fluid approaches, as it requires precise optimization of viscosity ratios, flow rates, and interfacial properties. Despite this complexity, the technique is not limited to polymers and has been extended to ceramics, metal oxides, and semiconductors. Recent advances have enabled even more complex architectures, including triple-layer and multichannel fibers [67], [68], [69], [70]. As a result, coaxial ES has found broad uses in biomedicine, energy storage, and environmental fields [71], [72], [73], [74], [75], [76], [77], [78]. In biomedical applications, coaxial fibers are particularly valuable for DD and TE owing to their capacity to combine biocompatibility with mechanical stability. The core-sheath configuration enables precise regulation of drug encapsulation and release, allowing sustained and programmable delivery profiles. Compared with conventional single-jet fibers, coaxial systems offer additional advantages such as hydrophilic-hydrophobic compartmentalization, improved mechanical strength, and tunable release kinetics for bioactive molecules and NPs [26], [71], [79].

#### 2.1.4 Emulsion electrospinning

Emulsion ES is a versatile and relatively inexpensive technique for encapsulating therapeutic agents within NFs, particularly for generating core-sheath-like architectures. It has been widely used to incorporate both hydrophilic and hydrophobic bioactive compounds, resulting in fibers with distinct internal compartments [80], [81]. The setup is similar to blend ES; however, rather than employing a single homogeneous solution, a multiphase emulsion is prepared and electrospun, as depicted in Fig. 3 [81]. Typically, a water-in-oil emulsion is formed using a surfactant, which disperses an aqueous drug phase within a polymer-containing organic phase [1], [80]. The polymer solution represents the continuous phase, while the active agents are confined to the dispersed droplets. During ES, rapid solvent evaporation increases the viscosity of the organic solution, promoting migration of the aqueous droplets toward the fiber core due to viscosity gradients. Under an applied electric field, droplets align and coalesce due to electrophoresis, forming core-sheath structures [82], [83]. The physicochemical properties of the drug determined the encapsulation behavior: low-molecular-weight compounds are often dispersed within the matrix, whereas higher-molecular-weight molecules are more likely to form well-defined core-sheath morphologies [82], [84]. Despite its advantages, emulsion ES has limitations compared with coaxial ES. Emulsion stability relies on surfactants, which may alter fiber surface properties and reduce cell adhesion. In addition, interfacial tension between phases can affect the structural integrity of sensitive biomolecules, potentially leading to partial degradation during processing [64], [85]. Consequently, this technique may be less suitable for fragile biological molecules that are sensitive to interfacial stress [1], [86].

**Fig. 3:**
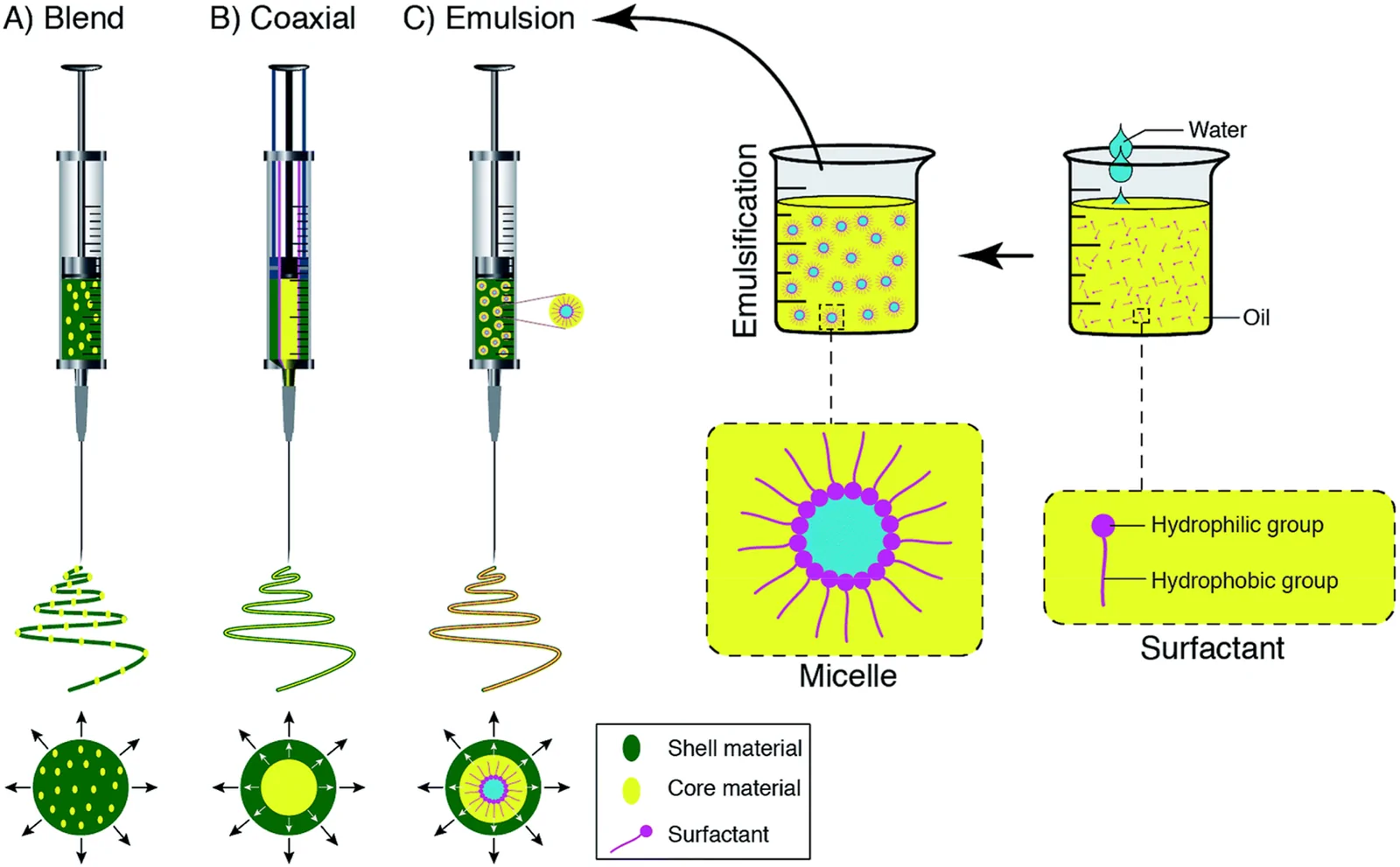
Illustration of the nozzle configurations containing a specific compound for three ES approaches: (A) blend ES, (B) coaxial ES, and (C) emulsion ES [81]. Copyright 2017. Adapted from the Royal Society of Chemistry.

#### 2.1.5 Triaxial electrospinning

Triaxial ES is a multiaxial technique that employs a specialized spinneret composed of three concentric capillaries, enabling the simultaneous processing of three distinct fluid streams. As part of the broader multiaxial ES family (including coaxial, quadraxial, and multi-nozzle systems), it allows the fabrication of complex multilayered NFs with enhanced structural and functional control [26]. A typical setup includes three syringe pumps for independent delivery of each solution, a grounded collector, a power supply, and a triaxial spinneret consisting of inner, middle, and outer channels. Under an applied electric field, the three solutions form a compound Taylor cone, resulting in fibers with clearly structured cores, intermediate, and outer layers upon solidification [26], [87], [88]. The primary advantage of triaxial ES is the ability to independently tailor the arrangement and function of each layer, enabling programmable and sequential drug release within a single fiber system [89]. This method is particularly attractive for TE applications, where mechanical support and biological functionality can be decoupled; for example, a mechanically robust synthetic polymer can serve as the core, while bioactive or cell-compatible materials are positioned in outer layers to promote cell adhesion and proliferation [90], [91]. Triaxial fibers are also well suited for the encapsulation of delicate therapeutics like growth factors, proteins, and poorly soluble drugs, as multilayer structures can protect active agents from harsh processing conditions and enable targeted or staged release, including rapid initial release followed by sustained or zero-order kinetics [92], [93]. In addition, these systems have been used to incorporate antimicrobial agents for prolonged antibacterial activity. Despite these advantages, triaxial ES is technically demanding. Careful control of solvent volatility, polymer compatibility, and molecular weight differences is essential, as premature solidification of the outer layer can destabilize fiber formation. In addition, accurate control of flow rates is required to maintain structural integrity and avert nozzle clogging, particularly when processing complex or bioactive core formulations [26], [94].

#### 2.1.6 Multiaxial electrospinning

Multiaxial ES is an advanced ES technique that employs three or more concentric nozzles (spinnerets) to simultaneously process multiple polymer solutions or fluids into a single, structurally complex nanofiber. Extending beyond coaxial (two-fluid) and triaxial (three-fluid) ES, this method enables the construction of fibers with highly precise configurations and multifunctional characteristics by combining multiple layers or compartments within a single fiber [25], [26]. In this process, polymeric solutions are delivered through nested capillaries (e.g., core-sheath-sheath configurations) and subjected to a strong electric field, producing multilayered fibers with structures such as core-double-shell systems and radial multilayer distributions [1], [25], [26]. An important advantage of multiaxial ES is its ability to incorporate different therapeutic agents into individual layers, enabling independent modulation of drug release profiles. This makes the technique particularly attractive for multidrug delivery systems requiring sequential, sustained, or stimuli-responsive release. Notably, antibacterial multiaxial fibers have demonstrated both rapid and prolonged antimicrobial activity, highlighting their promise for extended therapeutic applications [25], [95]. Despite these advantages, multiaxial ES remains technically demanding, requiring careful optimization of processing parameters, solvent systems, and polymer formulations to maintain jet stability and preserve the structural integrity of the resulting NFs [25], [96]. Fiber stability and functionality are strongly affected by factors such as inner-core solvent selection, polymer compatibility, and solvent volatility, all of which affect spinnability and interfacial integrity during fabrication [25], [97]. Consequently, careful selection of compatible solvents and spinnable fluids is essential to ensure robust fiber formation and to support the broader application of multiaxial ES in advanced DD and TE [25].

### 2.2 Needleless electrospinning techniques

Needleless electrospinning (NLES) is an innovative nanofiber fabrication procedure that differs from conventional ES by generating fibers straight from the polymer solution's surface rather than through needle-based nozzles. The earliest form of this method was reported by Simm and colleagues in 1979, who used an annular electrode as the spinneret [98]. Nevertheless, the method received little attention until the early 2000s, when Yarin and Zussman introduced a system in which a magnetic field induced surface spikes in the liquid, thereby initiating ES [99]. In 2009, Jirsak and colleagues patented a rotary-cylinder-based NLES device and later commercialized it under the name Nanospider [100]. Since then, numerous alternative NLES configurations have been developed, including systems based on air bubbles [101], conical wire spinnerets [102], flat metal plates [103], splashing spinnerets [104], rotating cones [105], container edges [106], toothed wheels [107], linear flumes [108], and shear-assisted spinnerets [109]. A distinguishing feature of NLES is that jet initiation occurs spontaneously across the polymer solution surface, independent of capillary-driven flow typical of needle-based systems [110]. As a result, spinneret design plays a prominent role in establishing jet stability, fiber morphology, and production efficiency [111]. Over the years, a diverse set of spinneret geometries has been established to improve throughput and fiber quality as shown in Fig. 4 [112].

**Fig. 4:**
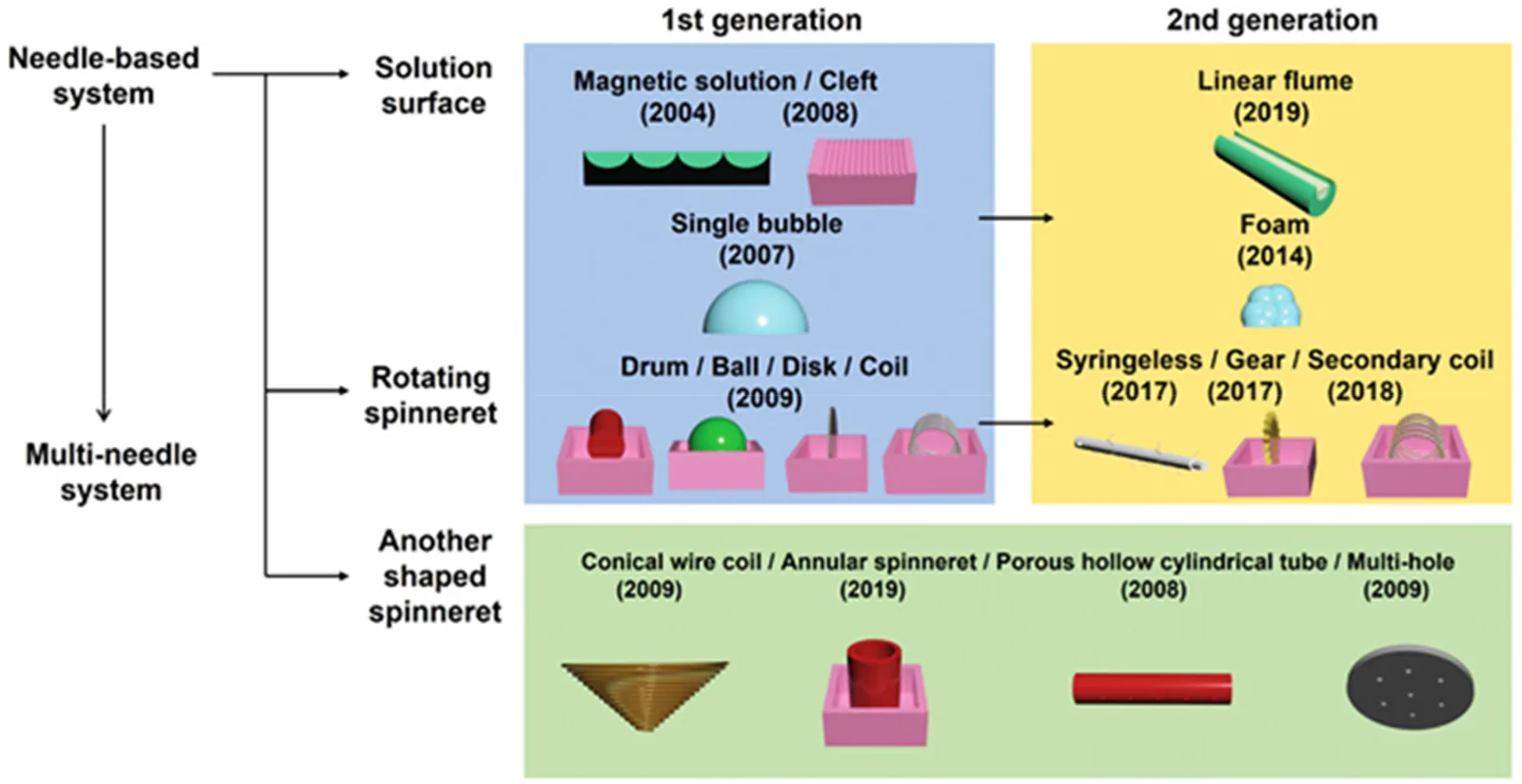
Classification of NLES techniques organized according to spinneret geometry and fiber generation mechanisms [112]. Copyright 2023. Reproduced with permission from John Wiley & Sons.

A major advantage of NLES is its ability to generate numerous polymer drops over a broad surface without relying on individual needles. Under an applied electric field, these droplets form multiple Taylor cones that emit jets toward a conductive collector, producing nonwoven fiber mats [112]. Unlike single- or few-needle systems, the concurrent formation of several jets significantly improves nanofiber production speeds [113]. The success of NLES depends strongly on the polymer solution's conductivity, which governs its interaction with the electric field and promotes stable jet formation, a requirement for continuous, uniform fibers [25], [114]. Because the technique utilizes open liquid surfaces, it is particularly attractive for large-scale production, offering simplified system design, easier maintenance, and greater flexibility in material processing [113], [114]. These features have supported its application in wound dressings, TE scaffolds, DD, biosensors, and filtration systems, where diverse polymer systems can be incorporated to produce functional NFs for biomedical and environmental uses [25], [115]. Despite these advantages, NLES still faces important challenges, including inconsistent jet formation, sensitivity to solution properties, and difficulties in controlling surface tension, all of which can contribute to variability in fiber morphology and reproducibility across batches or experimental conditions [25].

A range of NLES techniques have been developed, including corona, bubble, roller, high-speed, and alternating current (AC) ES [25]. These methods promote simultaneous multi-jet formation, significantly increasing fiber productivity compared to conventional needle-based systems. In roller ES, a rotating cylinder is partially immersed in a polymer blend solution, with an electric field applied between the roller surface and the collector. Rotation generates a thin liquid film that is subsequently stretched into NFs under the applied electric field. Although this configuration enables continuous and scalable fiber production, accurate control over fiber width and morphology remains difficult [116], [117], [118]. Bubble ES relies on gas flow to generate bubbles at the polymer surface. In the presence of a strong electric field, Taylor cones form on the bubble surface, leading to jet initiation and fiber formation. This technique enables high-throughput production and can yield hollow fiber structures; however, it is highly susceptible to solution and gas-flow parameters, which can negatively affect fiber uniformity and reproducibility [119], [120], [121]. In corona ES, a high-voltage electrode positioned close to the polymer solution induces corona discharge, generating a strong electric field that triggers Taylor cone formation and fiber discharge. The procedure is rather straightforward and easy to execute, allowing production of fibers with varying diameters, but maintaining consistent fiber alignment and uniformity remains a key limitation [122], [123]. High-speed ES employs rapidly rotating disks or similar elements to generate fibers through combined centrifugal force and shear stress. This approach can generate fibers with high aspect ratios and enhanced mechanical characteristics; however, the process needs dedicated equipment and precise adjustment of operational conditions [25], [124]. AC ES utilizes a periodically reversing electric field to induce cyclic deformation of the polymer solution surface, enabling multi-jet formation without needles. Unlike direct current systems, AC ES reduces charge accumulation on deposited fibers, resulting in more voluminous, highly porous, and 3D nonwoven structures [125], [126]. These characteristics make AC ES especially favorable for large-scale fabrication and applications that require a large interfacial surface and increased porosity. However, the oscillatory characteristic of the electric field introduces challenges to jet stability and trajectory control, making the achievement of fiber uniformity and alignment more difficult. Consequently, careful optimization of parameters such as frequency, voltage amplitude, and solution properties is essential for consistent fiber morphology [125], [126], [127].

## 3 Functionalization methods of electrospun nanofibers

Modifying or functionalizing the surface of ESNFs is a widely used strategy to enhance key surface features like wettability, surface charge, and roughness, thereby improving their performance in biomedical applications. The integration of biological molecules including nutrients, proteins, growth factors, and other bioactive or biomimetic materials into or onto nanofiber matrices, can create a favorable microenvironment that facilitates stem cell adhesion, differentiation, and proliferation. This, in turn, enhances the bioactivity of the platforms and broadens their applicability in regenerative medicine and related fields [128], [129], [130]. Surface modification strategies are generally categorized into two main approaches: (i) physical methods, such as adsorption or non-covalent functionalization, and (ii) chemical methods, including plasma treatment and graft polymerization [128], [129]. Physical modification is often preferred for its simplicity and for preserving the intrinsic structure of NFs, as it does not involve chemical reactions that may alter bulk properties [130]. This approach relies on weak, non-covalent interactions (e.g., van der Waals interactions, electrostatic forces, hydrogen bonding, and hydrophilic/hydrophobic effects) [131]. However, these interactions are typically less stable over time, which may limit long-term functionality. In contrast, chemical modification involves covalent bonding and permanent alteration of the nanofiber surface through the addition or activation of functional groups, thereby enhancing surface hydrophilicity and enabling stable biomolecule immobilization. This process is typically initiated by activating reactive groups on the nanofiber surface, such as -COOH, -NH_2_, or -OH, followed by their reaction with bioactive molecules to form stable covalent linkages [128], [132]. Because these bonds are chemically stable, functionalization achieved through this route is generally more durable than that achieved through physical adsorption. A key advantage of chemical surface modification is the creation of robust and long-lasting bioactive sites that significantly improve cell-material interactions, thereby promoting efficient cell adhesion and growth on the nanofiber scaffold [129], [133]. A schematic overview of common surface functionalization strategies for biomedical applications is presented in Fig. 5 [128].

**Fig. 5:**
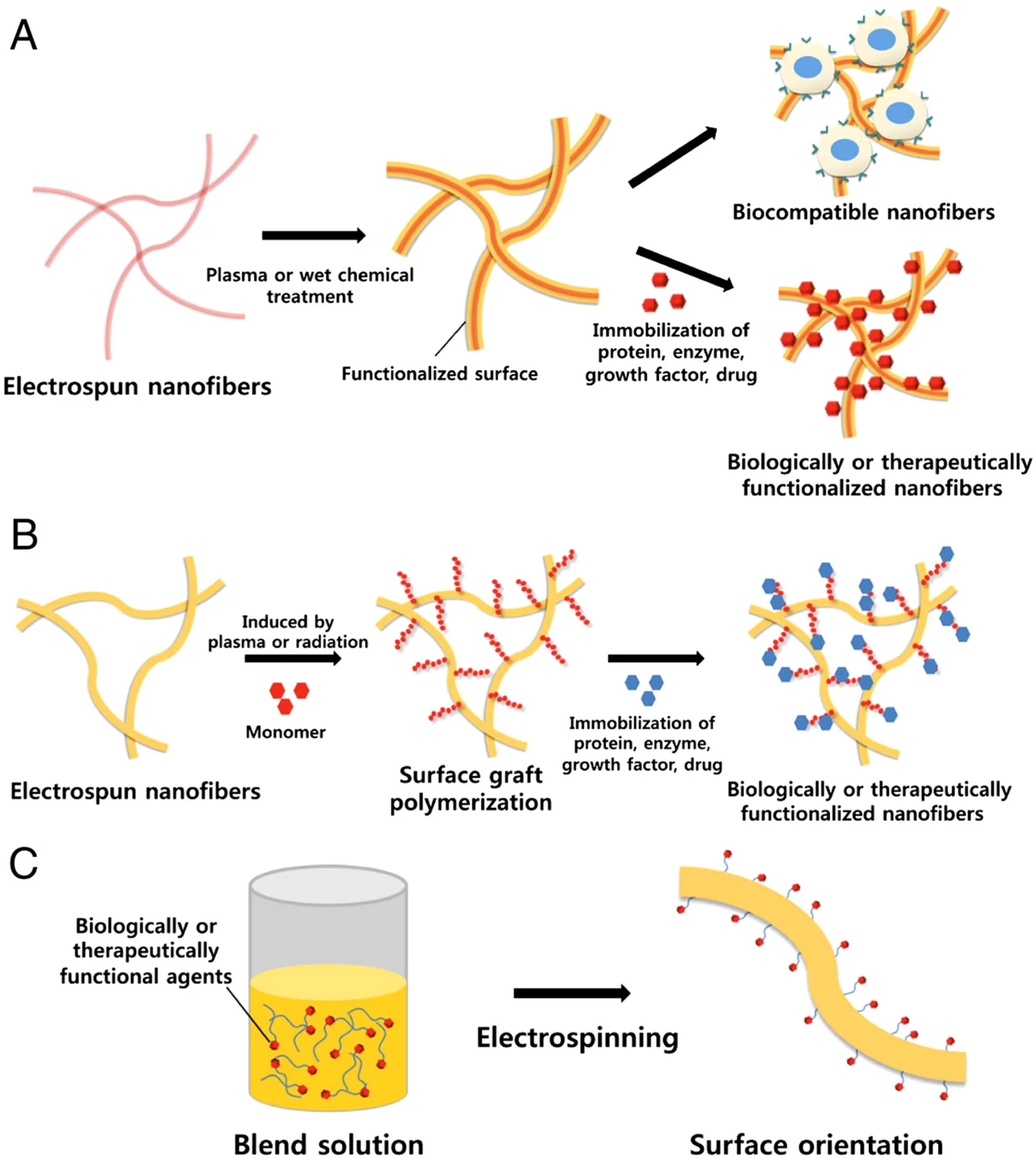
Surface modification approaches applied to ESNFs. (A) Wet chemical and plasma-based treatments; (B) Surface-initiated graft polymerization; and (C) Co-ES approaches [128]. Copyright 2009. Reproduced with permission from Elsevier.

### 3.1 Wet chemical methods

Wet-chemical treatment is a widely used functionalization strategy for ESNFs, involving chemical reactions conducted under acidic or basic conditions or in the presence of nucleophiles such as diamines. These reactions typically lead to selective cleavage of polymer chains at labile sites, such as ester bonds commonly present in polyester-based NFs [129]. Unlike purely physical modification methods, which are largely confined to the surface, wet chemical approaches can introduce hydrophilic functional groups both at and beneath the fiber surface, thereby enabling more extensive surface activation. The degree of modification depends on several parameters, including reaction time, reagent type and concentration, and the polymer's intrinsic chemical composition. Careful optimization of these conditions is therefore essential to avoid excessive degradation of the polymer backbone, which may adversely influence the mechanical durability and overall stability of the NFs structure [133], [134]. Through controlled reactions, functional groups such as amine (-NH_2_), carboxyl (-COOH), and hydroxyl (-OH) can be added onto the nanofiber surface, enhancing its chemical reactivity and suitability for further biofunctionalization. Among the most commonly employed wet chemical approaches are hydrolysis and aminolysis. The efficiency and outcomes of these processes are strongly influenced by reagent type and concentration, as well as exposure duration, all of which govern the degree of surface modification and its compatibility with biomedical applications such as DD and TE [135].

#### 3.1.1 Aminolysis

Aminolysis is a prominent surface functionalization procedure used to graft free -NH_2_ groups onto nanofiber surfaces by covalently linking diamine molecules to reactive ester groups within the polymer, enabling the formation of -NH_2_-functionalized surfaces [136]. Common diamines used for this purpose include N-aminoethyl-1,3-propanediamine, ethylenediamine, and 1,6-hexanediamine, which have been applied to both aromatic and aliphatic polyesters [137]. Successful aminolysis depends on multiple reaction parameters, including diamine concentration, solution pH, treatment duration, and reaction temperature. The process generally enhances wettability through the introduction of -COOH, -NH_2_, and -OH groups, while increased surface roughness may improve biomolecule immobilization and cellular adhesion [129]. Because of its relatively rapid reaction time, often less than 1 h, aminolysis is frequently employed as a preparatory strategy for immobilizing growth factors and other bioactive agents.

Despite these advantages, the literature presents a less consistent picture than is often implied. While many studies report improved hydrophilicity and bioactivity following aminolysis, considerable variability exists in the reported extent of amine grafting, preservation of fiber morphology, and resulting biological outcomes. These discrepancies often stem from methodological differences, including the choice of diamine, solvent system, treatment severity, and characterization technique. For example, ninhydrin assays, Attenuated Total Reflectance Fourier Transform Infrared (ATR-FTIR), X-ray photoelectron spectroscopy (XPS), and contact angle measurements are frequently used to assess functionalization efficiency. Yet these methods probe different aspects of surface modification and are not always directly comparable [129], [132]. In some cases, improved wettability has been interpreted as evidence of successful amination even when direct quantification of surface amine density was lacking, potentially overstating functionalization efficiency [135], [136], [137], [138], [139], [140], [141], [142], [143], [144].

A notable unresolved issue concerns the role of polymer crystallinity in governing aminolysis efficiency. Several studies identify crystallinity as a major barrier, particularly in PCL, where highly ordered regions and a low ester-to-alkyl ratio may restrict nucleophilic attack [129], [132], [143]. However, interpretation of these findings remains complicated because many reports treat crystallinity as a bulk property measured by Differential Scanning Calorimetry (DSC) or X-Ray Diffraction (XRD), whereas aminolysis is inherently a surface-limited reaction. As suggested by Jeznach et al. [132], ESNFs may possess a highly crystalline skin layer distinct from the fiber core, and this radial distribution may exert greater control over aminolysis than bulk crystallinity alone. Yet few studies systematically distinguish surface crystallinity from overall crystallinity, raising the possibility that conflicting reports regarding polymer responsiveness to aminolysis may reflect unaccounted structural heterogeneity rather than intrinsic differences in polymer chemistry. This methodological limitation complicates cross-study comparisons and may partly explain why some investigations report minimal modification of PCL surfaces, whereas others observe substantial amine incorporation under ostensibly similar conditions [132], [133], [135].

Conflicting observations are also evident regarding the effect of aminolysis on NF structure and mechanics. For example, Yaseri et al. [135] proposed PCL ESNFs surface modification with relatively minor morphological changes after aminolysis compared with hydrolysis, whereas Jeznach et al. [143] reported fiber fracture (Fig. 6) under harsh aminolysis conditions. In the study by Yaseri et al., the modified surfaces supported fibroblast adhesion and spreading, with cells exhibiting a spindle-shaped morphology. Enhanced cell proliferation was also reported, suggesting that aminolyzed and hydrolyzed PCL NFs may serve as promising structures for TE applications [135]. Similarly, in the work of Jeznach et al., improved cell spreading was observed for both L929 and MG63 cells even at low amine concentrations, indicating sufficient bioactivity enhancement without significant polymer degradation [143]. Other studies have intentionally exploited extensive fragmentation to generate nanocylinders or nanofibrils. These divergent outcomes suggest that the boundary between beneficial surface modification and detrimental polymer degradation remains poorly defined and likely depends strongly on polymer microstructure, solvent-mediated swelling, and reaction kinetics [144], [145]. However, these variables are not consistently controlled or reported, limiting reproducibility. Likewise, while some authors associate increased amine density with improved cell adhesion and proliferation, others report enhanced bioactivity even at low amine concentrations [143], indicating that biological responses may not correlate linearly with functional group density. These results raise questions about whether observed cellular improvements arise from surface amine density itself, secondary changes in roughness and wettability, or other confounding factors.

**Fig. 6:**
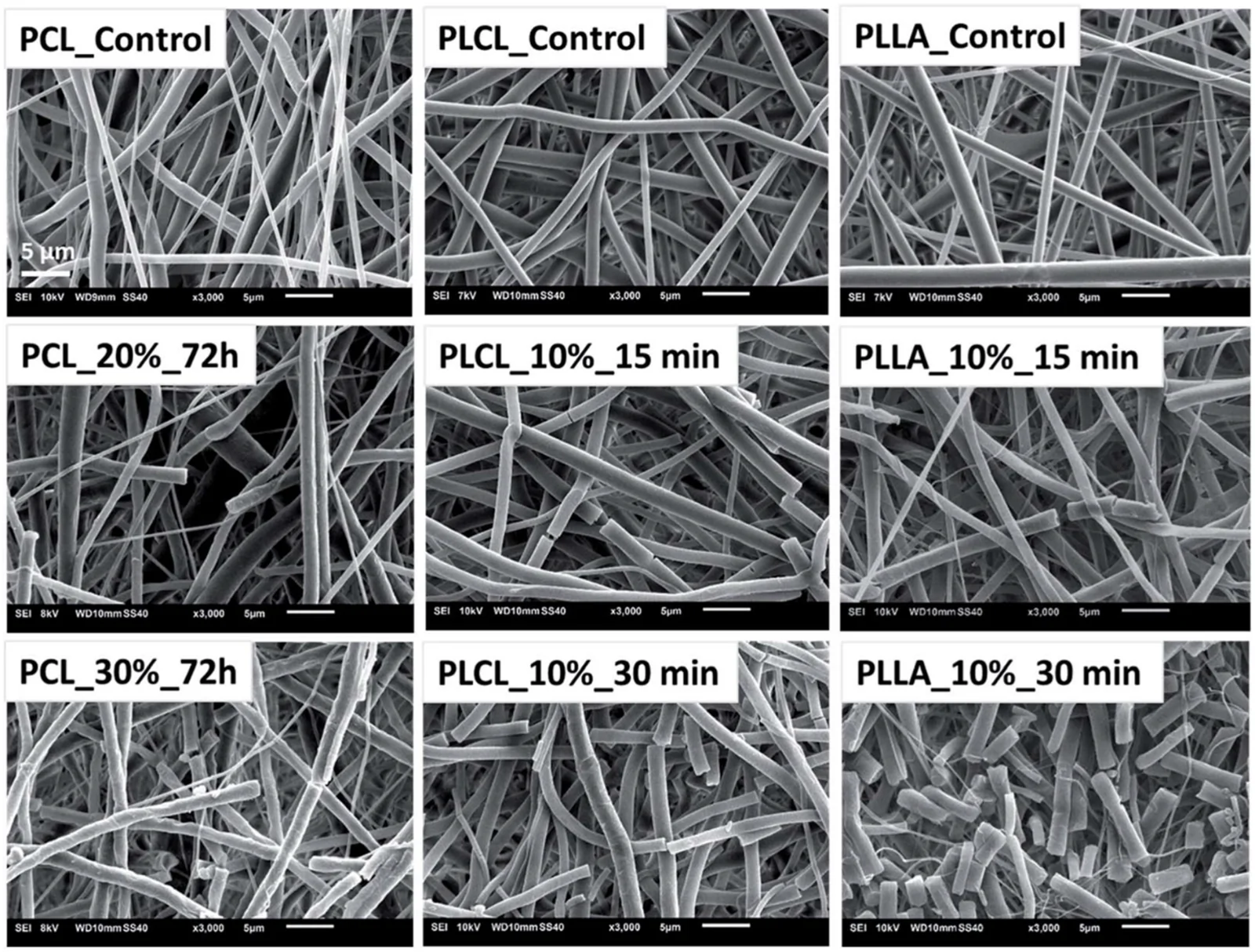
Scanning electron microscopy (SEM) micrographs depicting the fracture of fibers due to aminolysis under aggressive conditions [143]. Copyright 2022. Adapted from the Royal Society of Chemistry.

Studies involving dendrimer-mediated aminolysis and secondary functionalization strategies further illustrate both opportunities and uncertainties. Approaches involving polyamidoamine (PAMAM) and polypropylene imine dendrimers, graphene oxide deposition, or hyaluronic acid immobilization have demonstrated improved hydrophilicity, enhanced cell interactions, and even antibacterial effects in surface-functionalized ESNFs [138], [140], [141], [142], [143], [144], [145]. However, in many such studies, the contributions of aminolysis cannot be readily isolated from those of subsequently immobilized coatings or additives. As a result, improvements in biological performance are sometimes attributed broadly to aminolysis, despite likely arising from synergistic or even dominant effects of secondary modifications. More rigorous controls are needed to distinguish the role of initial amine functionalization from that of downstream bioactive modifications. Another underexamined issue concerns the long-term stability of aminolyzed surfaces. Zhu et al. [146] suggested that aminolyzed polymers may lose surface functional groups during storage at physiological temperatures or upon immersion in PBS due to chain rearrangement and burial of amines beneath the surface. Although frequently cited as a concern, this phenomenon has been investigated in only a few systems, and systematic aging studies across different polymers remain scarce. Consequently, the extent to which amine instability poses a universal barrier to translation remains uncertain.

Overall, while aminolysis remains a versatile and widely used surface modification technique, the field contains significant unresolved questions regarding reaction mechanisms, structure-reactivity relationships, reproducibility, and long-term functional stability. Future studies would benefit from standardized characterization protocols, clearer differentiation between surface and bulk crystallinity effects, and better-controlled comparisons that isolate aminolysis-specific contributions from those of subsequent functionalization steps. Addressing these methodological weaknesses is essential for resolving conflicting findings and improving the reliability and translational potential of aminolyzed nanofiber scaffolds [129], [132], [135].

#### 3.1.2 Hydrolysis

Hydrolysis-based surface modification can be broadly classified into acidic and alkaline hydrolysis**.** This technique involves cleavage of ester bonds in polyester-based materials, generating -COOH and -OH groups on the surface [133]. The reaction mechanism depends on whether acidic or basic conditions are applied. As with aminolysis, hydrolysis must be carefully controlled for temperature, pH, and reaction time, which can range from minutes to several hours depending on the material and conditions [147]. Surface hydrolysis has been widely used to enhance surface functionality of aliphatic biodegradable polyester films and scaffolds, particularly wettability. This effect arises from random cleavage of ester linkages on outer polymer chains, producing functional groups from partially decomposed, water-insoluble residues. The efficiency of hydrolysis depends strongly on reaction time and reagent concentration; in many cases, low concentrations are sufficient to accomplish surface modification, ensuring that the bulk properties or structural integrity remain unaffected [128], [137].

Acid hydrolysis proceeds via an electrophilic mechanism in which high-energy protons interact with polymer chains. This can result in carbon-centered radical formation and subsequent reactions involving unstable -OOH intermediates, producing -OH, -COOH, and ether (-R-O-R) groups [148]. Because such transformations are not easily achieved under mild conditions, acid hydrolysis typically requires strong acids, elevated temperatures, and longer reaction times [137]. Common reagents comprise hydrochloric acid (HCl), sulfuric acid (H_2_SO_4_), perchloric acid (HClO_4_), and organic acids such as lactic and acetic acid [147], [149], [150]. Several studies demonstrate its effectiveness. For example, HCl treatment of polyglycolic acid (PGA) scaffolds improved surface functionality, resulting in enhanced cardiac fibroblast adhesion and proliferation after 96 h [147]. Similarly, treatment with a perchloric acid/potassium chlorate system improved adhesion and growth of chondrocytes and fibroblasts, correlating with increased wettability [148]. However, acid hydrolysis can also introduce drawbacks; H_2_SO_4_ treatment of cellulose nanocrystals (CNCs) has been shown to reduce thermal stability and cause uncontrolled acid release during heating [150]. Due to such limitations, its application is relatively restricted.

Alkaline hydrolysis**,** in contrast, is a nucleophilic process driven by hydroxide ions (OH^−^) from bases such as sodium or potassium hydroxides (NaOH, KOH). These ions attack electrophilic sites on the polymer surface (e.g., carbonyl and ester groups), cleaving polymer chains and generating hydrophilic functional groups [151]. The extent of modification depends on hydroxide concentration and treatment conditions; higher concentrations increase surface activation but may also risk excessive degradation, including mass loss and thickness reduction [151]. Because it is easier to control and generally less damaging to bulk properties, alkaline hydrolysis is more widely used for nanofiber functionalization in biomedical applications. It is often employed as a pre-treatment step for biomolecule immobilization. For instance, NaOH-treated PLGA NFs have been successfully activated for collagen attachment using EDC/NHS chemistry as demonstrated in Fig. 7 [152]. This modification significantly improved wettability and supported enhanced cell viability in keratinocyte and fibroblast cultures over extended periods, demonstrating its suitability for skin tissue engineering (STE) [152].

**Fig. 7:**
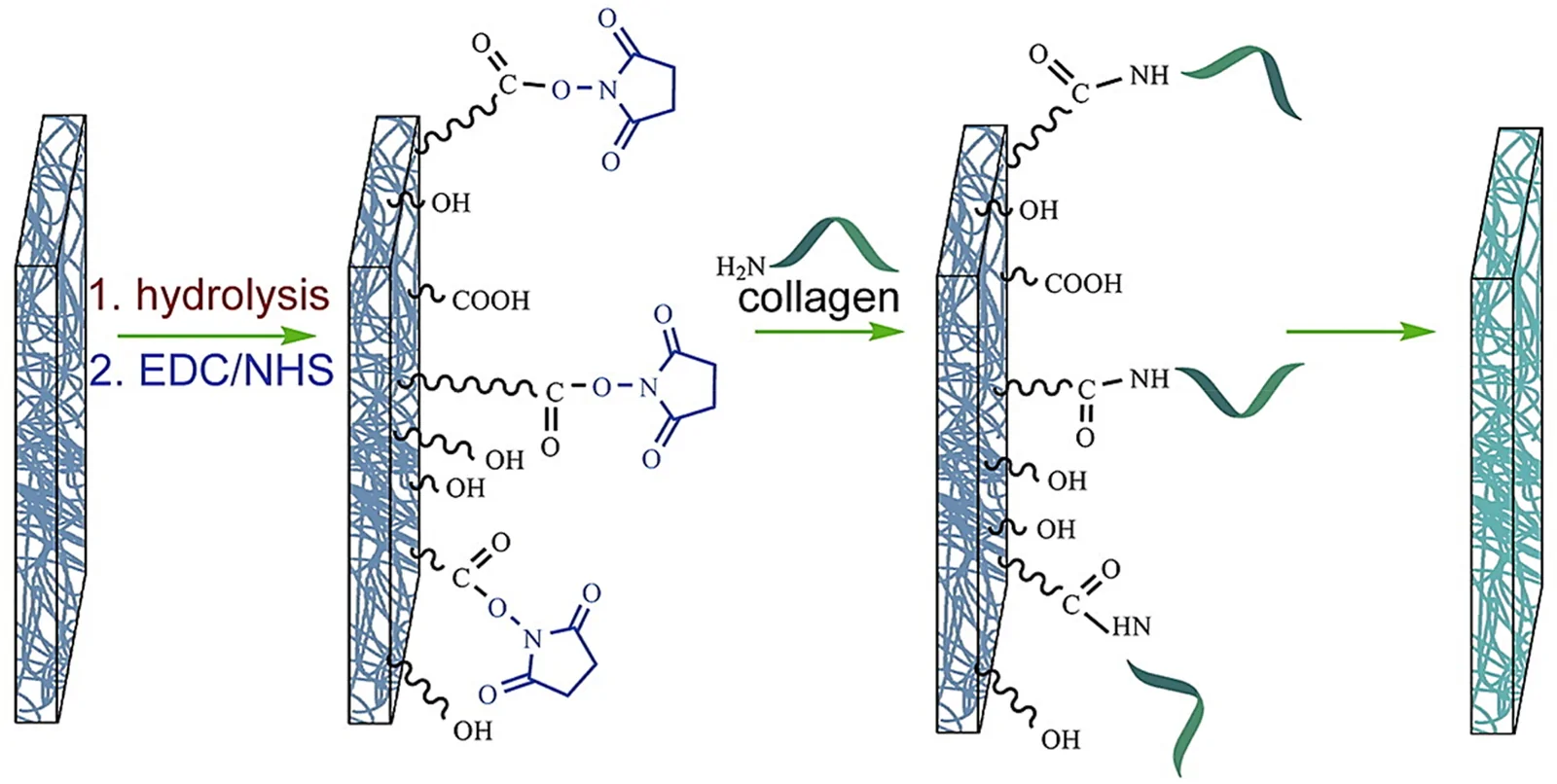
PLGA-based ESNFs surface modification via alkaline hydrolysis [152]. Copyright 2016. Reproduced with permission from Elsevier.

In another study, PCL ESNFs were treated with varying NaOH concentrations and exposure times to optimize flow behavior and enhance detection sensitivity in nucleic acid-based lateral flow assays (LFAs) [153]. The results showed that NaOH treatment had minimal effect on fiber diameter, pore size, and overall porosity. However, surface wettability improved significantly, as shown by a pronounced drop in water contact angle. This increase in hydrophilicity was linked to the formation of -OH and -COOH groups on the NF surface, with higher NaOH concentrations producing a more pronounced effect. Morphologically, hydrolyzed NFs exhibited a flatter and more compact structure compared to untreated PCL fibers. At higher NaOH concentrations, slight fiber erosion was observed, contributing to increased surface roughness. Notably, exposure to 2.0 M NaOH resulted in a substantial reduction in membrane thickness, possibly due to peeling under strongly alkaline conditions [135]. Despite these structural changes, the modified NFs demonstrated enhanced analytical performance, achieving a limit of detection (LOD) of 0.5 nM for synthetic Zika viral DNA. These findings highlight the potential of PCL NFs as a cost-effective and tunable alternative to conventional nitrocellulose membranes in LFA systems [153]. In a related work, Mobarakeh et al. functionalized PCL nanofiber membranes (NFMs) with Matrigel following alkaline hydrolysis [154]. The electrospun mats were first mixed with NaOH solution to expose reactive surface groups. Subsequent activation with 1-ethyl-3-(3-dimethylaminopropyl) carbodiimide hydrochloride (EDAC) enabled covalent linkage between the activated surface and -NH_2_ groups of Matrigel. This modification significantly enhanced hydrophilicity, as well as cellular proliferation and adhesion, indicating strong potential for TE purposes [154], [155].

#### 3.1.3 Chemical grafting

Chemical grafting is a widely used surface modification strategy where polymer chains or functional groups are covalently attached to electrospun substrates. This approach enables precise tuning of surface features by adding specific functional groups onto nanofiber surfaces. Chemical grafting is generally grouped into two main strategies: “grafting to” and “grafting from.” In the **“**grafting to” approach, pre-synthesized polymers with reactive end groups are covalently linked to functional sites on the nanofiber surface through coupling reactions. This method supports various polymerization techniques, including atom transfer radical polymerization (ATRP), controlled free-radical polymerization (CFRP), and anionic polymerization by employing various monomers like acrylonitrile, acrylates, and acrylamides [156], [157], [158], [159]. A key benefit of this strategy is the capability to fully characterize the polymer before grafting, allowing accurate control over distribution and molecular weight [160]. However, grafting density is often limited due to steric hindrance, which results in a non-uniform layer thickness and a self-limiting growth process. Additionally, thorough control of reaction conditions is necessary to avoid undesirable changes in nanofiber morphology or bulk mechanical properties [160], [161].

In contrast, the “grafting from” approach (*i.e.*, surface-initiated polymerization) enables polymer chains to grow in situ directly from reactive sites present on the substrate surface [160], [161]. This method enables higher grafting densities and more precise control over brush thickness at near-molecular resolution [161]. Typically, reactive functional groups are first added onto the nanofiber surface, and initiators are subsequently immobilized through post-polymerization modification, copolymerization, or polycondensation. Polymer chains then propagate outward from these surface-bound initiators [162], [163]. A representative example is the use of atmospheric-pressure plasma jet (APPJ) treatment to introduce reactive groups onto nanofiber surfaces. In this approach, argon plasma facilitates the deposition of 3-aminopropyltriethoxysilane (APTES), enriching the surface with amino functionalities [164]. These groups can later covalently bind to biomolecules, such as aldehyde-functionalized peptides. This strategy has been described to increase osteoblast adhesion and migration, demonstrating the effectiveness of surface-initiated grafting for biomedical applications [164]. Overall, chemical grafting provides a versatile platform for tailoring surface charge, wettability, and biocompatibility of ESNFs. A wide selection of biological molecules (e.g., proteins, antibodies, peptides, and enzymes) can be immobilized through covalent attachment [161], [165], [166]. Typically, this involves a two-step process: (i) introduction of reactive functional groups and (ii) coupling reactions such as amidation, esterification, or click chemistry [167]. Grafting techniques have proven effective for modifying diverse polymer substrates, including synthetic polymers like PET, PCL, and PLA, as well as natural polymers like cellulose, chitin, and chitosan [167], [168], [169], [170]. For example, HA grafted onto PCL ESNFs via carbodiimide chemistry resulted in improved mechanical properties, enhanced cell adhesion, and increased proliferation [171]. Similarly, poly (2-methacryloyloxyethyl phosphorylcholine) (PMPC)-grafted chitosan (CS) NFs synthesized via ATRP demonstrated superior cell attachment and uniform spreading. These improvements are attributed to synergistic interactions between functional groups on the grafted polymers and cellular components. Collectively, these findings highlight chemical grafting as a powerful and adaptable strategy for engineering nanofiber-based scaffolds with tailored functionalities for advanced biomedical applications [172].

Qin et al. [173] developed an effective strategy to modify hydrophobic PCL scaffolds by introducing an azide-terminated amphiphilic polymer, followed by conjugation with growth factor-loaded nanocapsules functionalized with dibenzocyclooctyne (DBCO). This approach utilized azide-alkyne click chemistry to enable controlled delivery of bone morphogenetic protein-2 (BMP-2). The BMP-2-containing nanocapsules were constructed via in situ polymerization around the protein core, forming a protective and degradable shell. The formulation included acrylamide (AAm) and N-(3-aminopropyl) methacrylamide (APm) in addition to the crosslinkers glycerol dimethacrylate (GDMA) and PLA-PEG-PLA dimethacrylate, which were described as biodegradable crosslinkers [173]. Following encapsulation, the nanocapsules were functionalized with DBCO-PEG-NHS, enabling bioorthogonal azide group conjugation to the PCL surface to form DBCO-n(BMP-2). Surface functionalization of the PCL scaffold was achieved via a simple one-step reaction based on non-covalent interactions between oleylamine (OA) chains and the polymer matrix resulting in the formation of an azide-presenting coating (OA-PLL-PEG-N_3_), which served as a reactive platform for subsequent click chemistry conjugation [173]. The nanocapsules were designed to degrade gradually under physiological conditions, allowing sustained BMP-2 release through hydrolysis of GDMA and PLA-PEG-PLA crosslinkers. Importantly, the encapsulated BMP-2 remained protected within the polymer shell and securely anchored to the scaffold surface, preserving its bioactivity. This surface modification strategy substantially enhanced the biocompatibility of the PCL scaffold, promoting enhanced cell proliferation and adhesion. Moreover, the sustained and localized release of BMP-2 effectively guided osteogenic differentiation of stem cells, demonstrating strong potential for bone tissue engineering (BTE) applications [173].

Although chemical grafting plays a pivotal role in nanofiber surface modification, it is associated with several limitations. One major challenge is achieving a uniform grafting density across the nanofiber surface. Variations in graft distribution can lead to inconsistent physicochemical properties and compromised performance; thus, precise control of grafting density is essential to ensure homogeneous functionalization and minimize surface defects [174]. In addition, harsh reaction conditions, such as the employment of strong oxidizing agents or high incubation temperatures, may disrupt NFs structure, thereby negatively affecting their mechanical characteristics and functionality [167]. However, careful optimization of reaction parameters, including treatment time and coupling agent concentration, has been shown to mitigate such damage and preserve nanofiber morphology [175]. Scalability and reproducibility also remain significant challenges. Small variations in reaction conditions during scale-up can lead to batch-to-batch inconsistencies, underscoring the need for process standardization and tighter control over reaction environments [167]. Furthermore, the utilization of hazardous chemicals and organic solvents raises concerns for human health and the environment, underscoring the importance of developing more sustainable and environmentally friendly grafting strategies [176]. Overall, precise tuning of grafting parameters is crucial for attaining the required surface functionality while maintaining the structural integrity and bulk properties of NFs [177], [178].

#### 3.1.4 Chemical crosslinking

Chemical crosslinking is a key strategy for the functionalization of ESNFs, forming inter-chain bonds with the polymeric chains thereby enhancing mechanical robustness, heat resistance, environmental stability, and biocompatibility [155], [179], [180]. Crosslinking can be accomplished through physical, chemical, or biological mechanism**s**, each using different agents or stimuli, including glutaraldehyde, formaldehyde, enzymes, UV irradiation, or thermal treatment [181], [182]. Physical crosslinking is based on non-covalent interactions including hydrogen bonding, van der Waals forces, hydrophobic interactions, and electrostatic attractions which are widely reported in the literature [179], [183], [184], [185]. For example, blending oppositely charged polymers can induce electrostatic crosslinking, while thermal treatment may promote conformational changes in polymer chains. In protein-based systems, heating can cause partial unfolding, exposing hydrophobic domains that facilitate intermolecular interactions. Dehydration during thermal processing further enhances chain proximity and interaction [179], [186], [187]. This approach avoids toxic chemicals but often requires precise optimization to ensure sufficient stability and durability [188]. Chemical crosslinking**,** in contrast, involves the formation of covalent bonds using reactive agents such as glutaraldehyde, formaldehyde, or genipin [179], [182], [189], [190]. This method significantly improves mechanical integrity and resistance to environmental degradation. However, residual crosslinking agents or side reactions may introduce cytotoxicity, necessitating careful post-treatment and biocompatibility evaluation, especially for biomedical applications [179], [190]. Biological crosslinking offers a safer alternative by employing naturally derived enzymes or biomolecules. Enzymes such as cellulase, transglutaminase, laccase, and β-galactosidase can catalyze bond formation under mild conditions [179], [191]. For instance, transglutaminase catalyzes the covalent linkage of lysine and glutamine residues in proteins [192], [193]. This approach enhances structural stability while promoting desirable biological responses, including improved cell adhesion, angiogenesis, and reduced inflammation [194], [195]. The selection of a crosslinking strategy depends on the polymer composition, the required material characteristics, and the intended application. Achieving a balance between functionality and biocompatibility is particularly critical in fields such as TE, DD, and wound healing.

Crosslinked ESNFs have garnered substantial attention in biomedical applications due to their improved performance [179], [196], [197], [198]. Crosslinking can reduce cytotoxicity and inflammatory responses while enhancing cell attachment and proliferation. For example, acid-oxidized xanthan gum has been used to crosslink gelatin NFs, improving their biocompatibility [199]. Similarly, cold plasma-treated gelatin NFs exhibited enhanced cell adhesion and growth [200], while enzymatic crosslinking using horseradish peroxidase (HRP) enabled efficient plasmid DNA delivery [201]. Crosslinking also facilitates the design of multifunctional scaffolds. For instance, genipin-crosslinked coaxial NFs composed of gelatin and PLA have been developed for combined anticancer therapy and bone regeneration, incorporating bioactive agents such as doxorubicin (DOX) and icariin (ICA) [202]. In another study, CS-based NFs functionalized with fucoidan and copper sulfide NPs via polyelectrolyte complexation and genipin crosslinking demonstrated enhanced stability, antibacterial activity, and bone regeneration potential [203]. Additionally, physical crosslinking of polyvinyl alcohol (PVA)/gelatin NFs using methanol produced denser, water-resistant structures that supported osteoblast adhesion and proliferation [204].

Advanced crosslinking approaches have also been explored. Radiation-induced crosslinking of PLLA NFs using triallyl isocyanurate (TAIC) and gamma irradiation yielded stable networks with good cell compatibility and no cytotoxic effects [205]. Similarly, genipin-crosslinked PCL/gelatin membranes demonstrated enhanced osteogenic differentiation in pre-osteoblast cells [206]. Hybrid CS/calcium phosphate (CaP) NFs crosslinked with vanillin and incorporating silver ions exhibited antibacterial activity, sustained ion release, and excellent compatibility with bone marrow stromal cells [207]. Collectively, these studies highlight crosslinking as a versatile and powerful approach for reinforcing the structural integrity, functional properties, and bioactivity of ESNFs. When carefully optimized, crosslinking enables the development of novel scaffolds that maintain structural integrity while delivering tailored bioactivity for a wide panel of biomedical applications.

### 3.2 Physical methods

#### 3.2.1 Plasma treatment

Plasma treatment is a widely used post-modification procedure for modulating the surface features of ESNFs, including adhesion, roughness, and wettability [155], [208]. It is considered a cost-effective, environmentally friendly, and solvent-free approach that offers advantages over conventional modification procedures, particularly in terms of energy efficiency, short processing times, and the absence of hazardous chemicals [209], [210]. In contrast to many chemical treatments, plasma modification is rapid, typically requiring only seconds to minutes to achieve the desired surface changes [129]. A key advantage of plasma treatment is its ability to selectively alter the nanofiber surface without modifying the bulk structure or generating harmful byproducts [155], [211]. By carefully selecting the plasma source and optimizing operational parameters, such as working pressure, applied voltage, and gas flow rate, it is possible to add functional groups, generate radical sites, and induce limited chain scission on the fiber surface. These modifications enhance surface reactivity, improve biocompatibility, and facilitate subsequent covalent immobilization of biomolecules [210], [211]. Fig. 8 illustrates various plasma sources used for nanofiber surface modification and the interaction of plasma gases with the fiber surface [212]. Several types of gases, including oxygen, hydrogen, argon, ammonia, nitrogen, helium, and carbon dioxide, are commonly used in plasma treatment. Adjusting the gas composition allows the incorporation of specific functional groups; for instance, oxygen or air plasma promotes the formation of oxygen-containing groups (e.g.,-COOH), while ammonia plasma introduces amine functionalities. These changes significantly increase surface energy, wettability, and roughness [155], [213], [214]. In addition to surface activation, plasma techniques can additionally be employed for thin polymer coating deposition, further enabling the attachment of proteins and other biomolecules onto nanofiber structures [215]. Despite its advantages, plasma treatment is limited by the shallow penetration depth of reactive species, which is governed by exposure time and plasma power [216]. To address this limitation, plasma-treated NFs are generally further functionalized with ECM proteins such as gelatin, collagen, and fibronectin. significantly promotes cell–material interactions and boosts biological functionality, with notable relevance to TE [213], [217], [218].

**Fig. 8:**
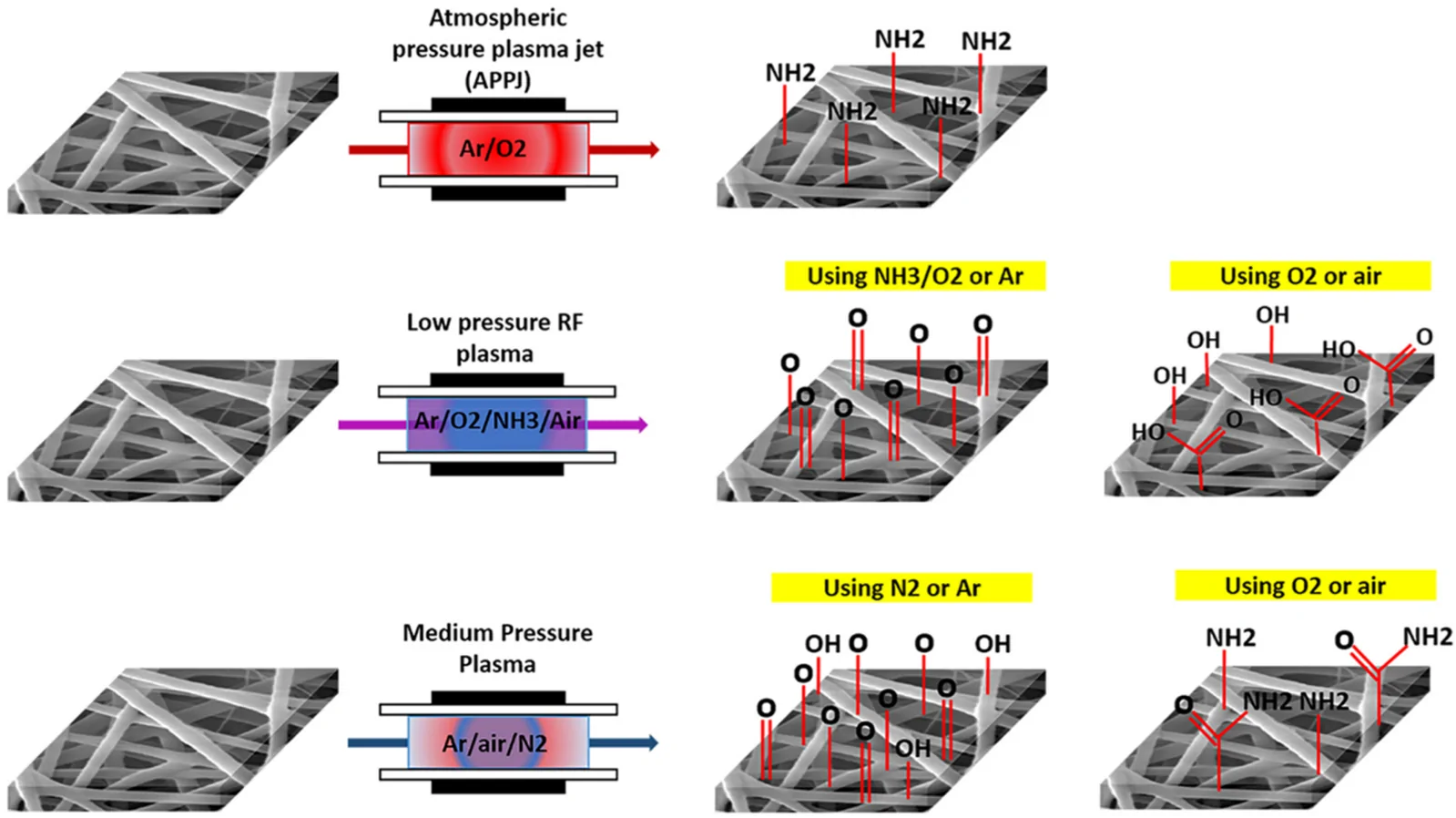
Representation of the different plasma sources and their interaction with the nanofiber surface [212]. Copyright 2022. Reproduced with permission from Elsevier.

Chen et al. [213] improved the compatibility of PLLA NFs with chondrocytes by functionalizing their surface with cationized gelatin (CG) for cartilage tissue engineering (CTE) applications. Oxygen plasma treatment was first applied to introduce -COOH onto the NF surface, creating reactive sites for subsequent covalent grafting of CG. Physicochemical characterization confirmed the successful immobilization of CG on the plasma-treated PLLA NFs. Both in vitro and in vivo studies demonstrated that chondrocytes cultured on CG-modified PLLA NFMs maintained high viability, exhibited a rounded morphology, and formed cartilage-like tissue enriched in sulfated glycosaminoglycans (sGAG) and type II collagen. These findings underscore the effectiveness of plasma-assisted functionalization in improving cell-material interactions and support the potential of CG-modified PLLA scaffolds for cartilage regeneration [213]. In a related study, Tabaei et al. [219] demonstrated the synergistic effects of coral incorporation and plasma treatment on CS/polyethylene oxide (PEO) NFs for BTE. Coral, a calcium-rich component, was integrated into electrospun CS/PEO NFs, which were subsequently treated using medium-pressure dielectric barrier discharge plasma with argon (Ar), nitrogen (N_2_), or air (Fig. 9) [219]. Plasma exposure introduced O_2_- and N_2_-based functional groups on the NF surfaces, thereby improving wettability and surface reactivity. This modification promoted osteoblast activity, while the presence of coral further supported initial cell adhesion. The biomineralization capacity of these scaffolds was evaluated by immersion in simulated body fluid (SBF). Plasma-treated CS/PEO NFs resulted in globular carbonate apatite nanocrystals formation. In contrast, coral-containing composites showed a denser and more uniform deposition of flake-like nanocrystals, resulting in a thicker mineralized layer while preserving interfibrous porosity and structural integrity. These results demonstrate that combining plasma treatment with bioactive fillers is an efficient approach to enhance the biological and physicochemical performance of nanofiber structures for bone regeneration applications [219].

**Fig. 9:**
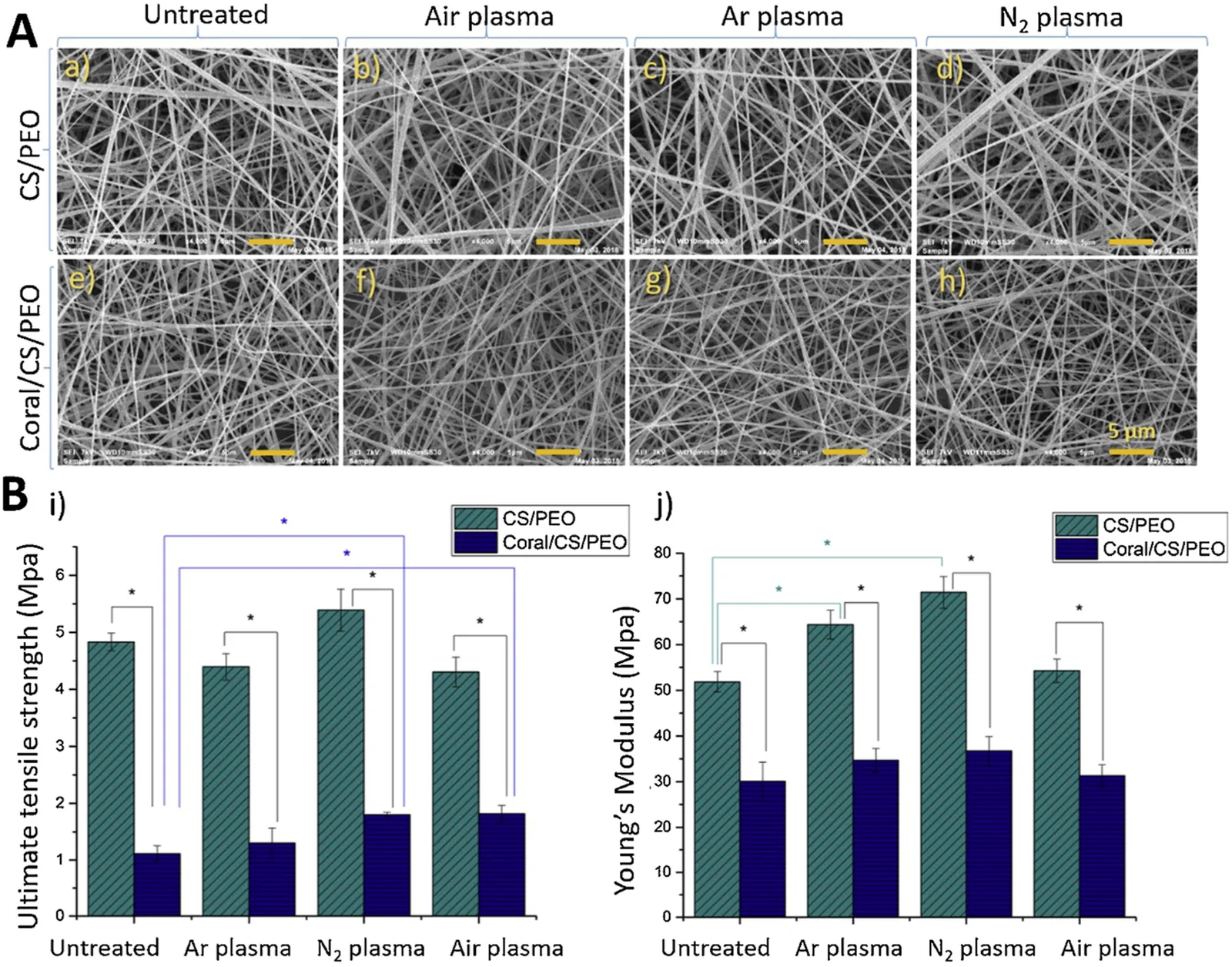
(A) SEM images of CS/PEO NFs and Coral/CS/PEO NFs before treatment (a, e) and after plasma treatment with air (b,f), Ar (c,g), and N_2_ (d,h) (scale bar = 5 μm). (B) Mechanical characteristics of the developed NFs under the same conditions. (i) Tensile strength assessment and (j) Young's modulus. Asterisks (*) indicate statistical significance (*P* < 0.05) [219]. Copyright 2021. Reproduced with permission from Elsevier.

Plasma treatment is a powerful way for altering the physicochemical features of biomaterials, thereby influencing cell-material interactions and cellular behavior [220], [221], [222]. For instance, Nandakumar et al. [220] utilized radio-frequency (RF) oxygen plasma to modify ESNFs composed of polybutylene terephthalate (PBT)/polyethylene oxide terephthalate (PEOT) copolymers. The treatment produced nanoscale surface roughness and increased oxygen content, thereby enhancing hydrophilicity. This modification also promoted greater bovine serum albumin (BSA) adsorption after 15 and 30 min of exposure. After 21 days of culture, plasma-treated nanofiber meshes supported more extensive cell growth compared to untreated controls. Gene expression analysis further revealed upregulation of bone-related glycoproteins, indicating that oxygen plasma can promote osteogenic differentiation of human mesenchymal stem cells (hMSCs) [220]. In another work, ES PCL nanofiber meshes were modified using RF plasma with argon or oxygen at varying power levels and exposure times [221]. Plasma treatment significantly improved surface hydrophobicity, as demonstrated by reduced water contact angles. Among the cell types evaluated (chondrocytes, fibroblasts, and osteoblasts), osteoblast-like cells displayed the greatest enhancement in adhesion and proliferation on treated scaffolds. Similarly, biodegradable NFs composed of PGA, PLLA, and PLGA were modified via O_2_ plasma followed by in situ acrylate attachment. This dual modification introduced -COOH groups, increasing hydrophilicity and significantly improving fibroblast adhesion and proliferation [222]. Jiang et al. [65] demonstrated the versatility of plasma treatment by combining air plasma activation with polydopamine (PDA) coating on PCL NFs. The resulting scaffolds enabled pH-responsive loading and release of DOX. Under acidic conditions, enhanced drug release led to increased cytotoxicity against cancer cells, underlining the potential of plasma-modified NFs for targeted DD applications, including gastric cancer therapy and localized treatments [65].

Plasma treatment can also be integrated with other modification strategies to enhance stem cell differentiation. Kooshki et al. [223] combined oxygen plasma treatment with lipopolysaccharide (LPS) preconditioning of mesenchymal stem cells (MSCs) on PLLA NFs. This approach improved surface hydrophilicity and cell attachment and significantly enhanced osteogenic differentiation, as confirmed by alkaline phosphatase (ALP) activity, calcium deposition, gene expression, and immunostaining analyses. In another research, Asadian et al. [224] proposed a two-step modification strategy for PCL ESNFs. Initially, plasma polymerization of acrylic acid introduced -COOH groups onto the surface. After that, post-plasma grafting of ethylenediamine via EDC/NHS coupling was performed, yielding -NH_2_-functionalized NFs as illustrated in Fig. 10 [224]. Surface characterization confirmed successful modification, including reduced negative charge and altered wettability. These changes significantly enhanced bone marrow stem cell adhesion and proliferation, which is believed to be due to the mutual effects of surface roughness, tailored chemistry, and optimized surface charge [224]. Overall, these studies demonstrate that plasma treatment (either alone or in combination with other modification techniques) offers a greatly adaptable route for enhancing the biological performance of ESNFs. Its ability to precisely tailor surface chemistry and functionality makes it particularly beneficial for regenerative medicine, TE, and targeted therapeutics.

**Fig. 10:**
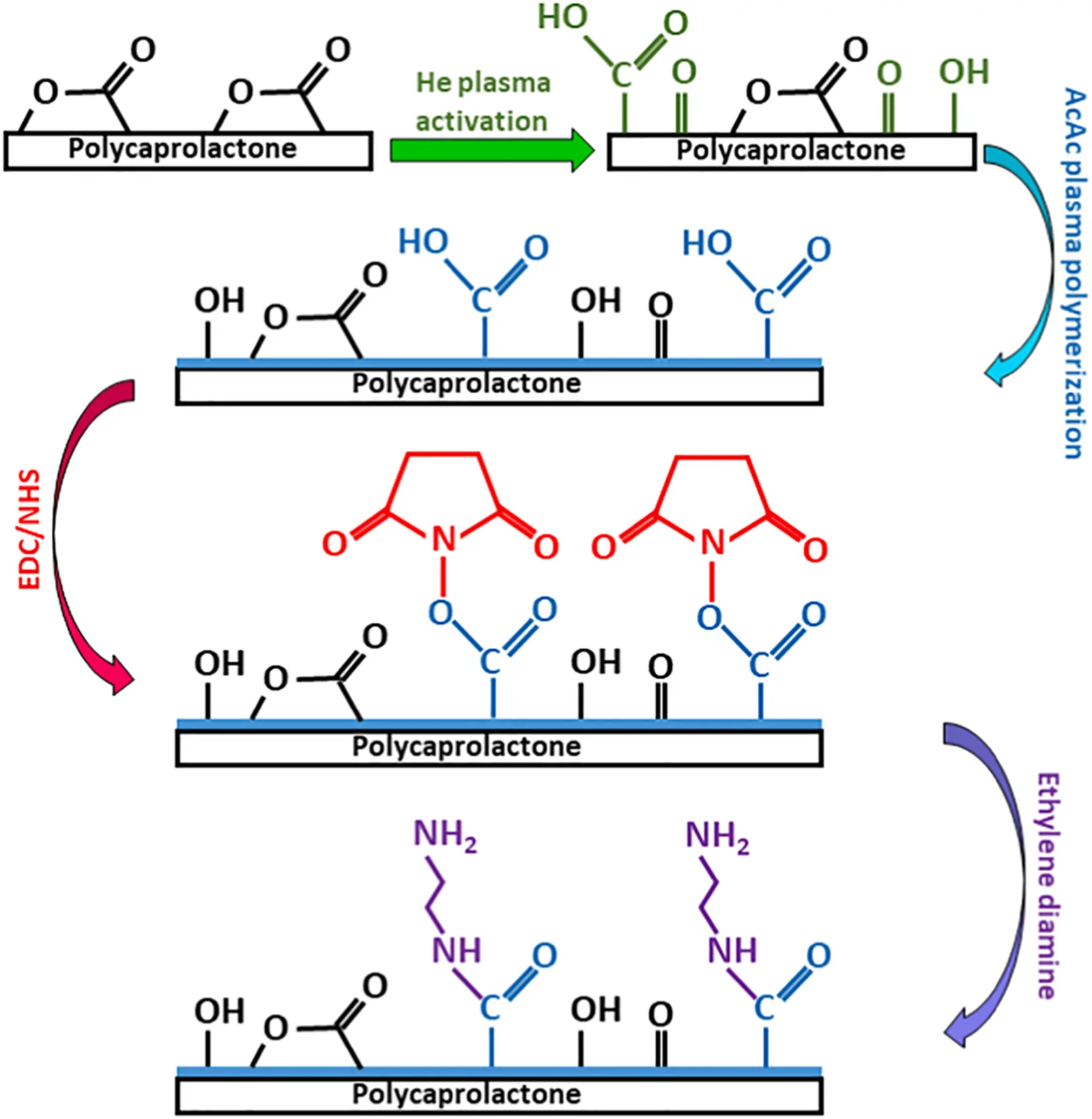
Schematic description of PCL NFs surface modification through post-plasma grafting [224]. Copyright 2021. Reproduced with permission from Elsevier.

Onak et al. [225] elaborated a surface functionalization strategy for PLGA ESNFs by conjugating biomimetic peptides rich in aspartic acid (ASP) and glutamic acid (GLU). This was achieved using EDC/NHS coupling chemistry following cold atmospheric plasma (CAP) treatment. The approach aimed to enhance osteoinductive properties and assess the ability of the modified structure to support osteogenic differentiation of human bone marrow-derived hMSCs. Comprehensive surface characterization using contact angle measurements, FTIR, XPS, and SEM, demonstrated that CAP treatment significantly improved surface hydrophilicity and increased the density of -COOH groups, enabling efficient peptide conjugation. Biological evaluations revealed that ASP-functionalized NFs (ASP-pNF) outperformed both GLU-functionalized (GLU-pNF) and untreated CAP NFs. Specifically, ASP-pNF scaffolds exhibited higher ALP activity, increased calcium mineral deposition, and upregulated expression of key osteogenic biomarkers such as osteocalcin (OC), osteopontin (OP), and collagen type I (Col-I). These findings underline the strong potential of ASP-conjugated NFs for biomimetic scaffold design in bone regeneration applications [225]. Furthermore, multiple studies [226], [227], [228], [229] have consistently demonstrated that plasma-based surface treatments significantly improve cell adhesion and proliferation on ESNFs. Overall, plasma treatment represents a versatile and tunable strategy for introducing functional groups onto nanofiber surfaces, enabling their optimization for various biomedical applications [208], [212], [213], [214], [215], [216], [217], [221], [230].

#### 3.2.2 Ultraviolet treatment

Ultraviolet (UV) treatment is one of the simplest and most affordable approaches for surface modification of ESNFs, as it eliminates the need for chemical reagents or vacuum systems [231]. UV irradiation alters surface chemistry by inducing polymer chain scission and generating functional groups, while largely preserving nanofiber morphology and alignment [232], [233]. Additionally, UV exposure produces surface-bound free radicals that act as reactive sites for subsequent modifications, including grafting, crosslinking, and covalent immobilization of biomolecules [234], [235]. These modifications enhance surface hydrophilicity, tailor chemical functionality, and significantly improve cell adhesion and proliferation [236], [237]. Moreover, UV-induced crosslinking can be applied to both hydrophilic and hydrophobic polymeric NFs to improve water resistance and structural stability, resulting in insoluble polymer networks suitable for long-term biomedical applications [238]. Tort et al. [239] studied the effect of UV exposure time on nanofiber-based wound dressings. They reported that 2 h of irradiation achieved effective sterilization without compromising fiber morphology, wettability, mechanical properties, or bioadhesion. Khosravi et al. [240] demonstrated the covalent immobilization of silk fibroin (SF) onto PCL NFs using UV-ozone activation, followed by acrylic acid grafting to introduce -COOH groups. In vitro studies with NIH 3T3 fibroblasts proved enhanced cytocompatibility, cell adhesion, and proliferation compared to untreated scaffolds. In a related approach, UV photolithography was employed to fabricate microporous electrospun scaffolds, in which patterned UV exposure generated ∼100 μm pores that facilitated directional muscle cell migration, underscoring the significant potential of UV-based patterning for 3D TE [240]. Similarly, Asadi et al. [241] utilized UV treatment to immobilize enzymes on PAN/PVA NFs, preserving structural integrity while enabling controlled enzyme release. Andrews et al. [242] compared UV-ozone and ethylene oxide sterilization of Tecoflex® SG-80A PU NFs, finding that UV-ozone treatment reduced surface roughness via photo-oxidative effects and improved cell coverage through favorable topographical changes. Mohsen et al. [243] developed UV-treated PVA/CS NFs for wound healing, demonstrating broad-spectrum antibacterial activity, high cell viability (96.9% at 2000 mg/mL), and complete wound closure within 72 h. Collectively, these studies establish UV treatment as a safe, efficient, and versatile strategy for tailoring the surface properties of ESNFs, enabling their application across a wide span of biomedical fields.

#### 3.2.3 Simple physical adsorption

Physical adsorption is one of the most straightforward, cost-effective, and non-destructive strategies for functionalizing the surface of ESNFs. This method relies on non-covalent interactions to enable molecular attachment onto nanofiber surfaces [244]. A significant advantage of this method is its capability to introduce functional moieties without altering the bulk properties or structural integrity of the NFs [167]. Typically, the process involves spraying or immersing NFs in biomolecule-containing solutions, allowing spontaneous adsorption driven by intermolecular interactions [129]. Physical adsorption is applicable to various electrospinnable polymers such as PLLA, PLGA, PCL, PVP, PVA, and PEG [245], [246], [247], [248]. Commonly adsorbed biomolecules include peptides, proteins, enzymes, polysaccharides, growth factors, antibacterial agents, and vitamins [128], [249], [250], [251]. This strategy enhances cellular recognition of surface cues, promotes cell adhesion, and improves overall biocompatibility without compromising scaffold architecture [129], [252]. Furthermore, it enables rapid immobilization of bioactive molecules and helps preserve their bioactivity by avoiding harsh processing conditions such as organic solvents, elevated temperatures, or high-voltage conditions during ES [247], [253].

Casper et al. [245] incorporated low-molecular-weight heparin (LMWH) conjugated with polyethylene glycol (PEG-LMWH) into PEO/PLGA NFs to enhance their bioactivity for tissue regeneration and DD. The modification preserved fiber morphology while improving binding affinity for fibroblast growth factors. Similarly, Patel et al. [246] combined surface adsorption of ECM proteins and growth factors with fiber alignment to mimic native tissue architecture. Their results showed that aligned NFs significantly enhanced neurite outgrowth, while biochemical functionalization further amplified this effect, underscoring the synergistic role of physical and biochemical cues. Shitole et al. [247] demonstrated the functionalization of core-sheath ESNFs composed of a poly (vinyl pyrrolidone)-iodine (PVP-I) core and a PCL shell using Poly-L-lysine as a surface modifier. The modified NFs exhibited significantly enhanced protein adsorption, with higher affinity for BSA and fibrinogen than pristine PCL. Cytocompatibility studies confirmed non-toxicity at an optimized PVP-I concentration (3.0 wt%), while poly-L-lysine-functionalized NFs showed enhanced cell viability after 7 days of culture. Additionally, the scaffolds demonstrated strong antimicrobial activity against both *S. aureus* and *E. coli*, highlighting their potential as multifunctional wound dressings that promote early-stage healing and prevent infection.

Chen et al. [254] functionalized silica/PVP composite NFs with laminin, a key ECM matrix protein. The modified scaffolds exhibited excellent biocompatibility, biodegradability, and structural stability for up to 18 days. Notably, neurite extension was markedly greater on covalently functionalized fibers as opposed to both unmodified and physically adsorbed laminin-coated fibers, indicating that while adsorption is simple, covalent strategies may provide stronger and more stable biofunctional effects in certain applications. Ye et al. [255] reported a facile adsorption-based modification of thermoplastic polyurethane (TPU) NFs using cellulose nanofibrils under ultrasonication. This treatment resulted in uniform surface coating, enhanced hydrophilicity, and improved mechanical properties. Importantly, the modified NFs significantly promoted adhesion and proliferation of human umbilical vein endothelial cells (HUVECs), demonstrating their suitability for vascular tissue engineering (VTE).

In DD applications, Kim et al. [256] encapsulated the hydrophilic antibiotic cefoxitin sodium (Mefoxin®) onto PLGA NFs. While initial drug release was rapid, the incorporation of PEG-b-PLA enabled sustained release over seven days, with antimicrobial activity exceeding 90% inhibition of *Staphylococcus aureus*. Similarly, Bolgen et al. [257] developed PCL NFMs loaded with Biteral®, which significantly reduced postoperative adhesions and supported tissue healing in vivo. In a related work, Xiao et al. [258] engineered a 3D PCL NF scaffold through sequential surface hydrolysis, biomimetic mineralization, and SF coating as demonstrated in Fig. 11. This process generated a -COOH -rich, bone-mimicking architecture that supported directional cell growth and enhanced proliferation. In vivo studies further confirmed improved tissue organization and accelerated bone regeneration [258]. Overall, physical adsorption represents a resourceful and useful surface modification strategy for ESNFs. Despite its simplicity and preservation of biomolecule activity, its relatively weak interaction strength compared to covalent methods may limit long-term stability, highlighting the need to tailor the approach to specific biomedical applications.

**Fig. 11:**
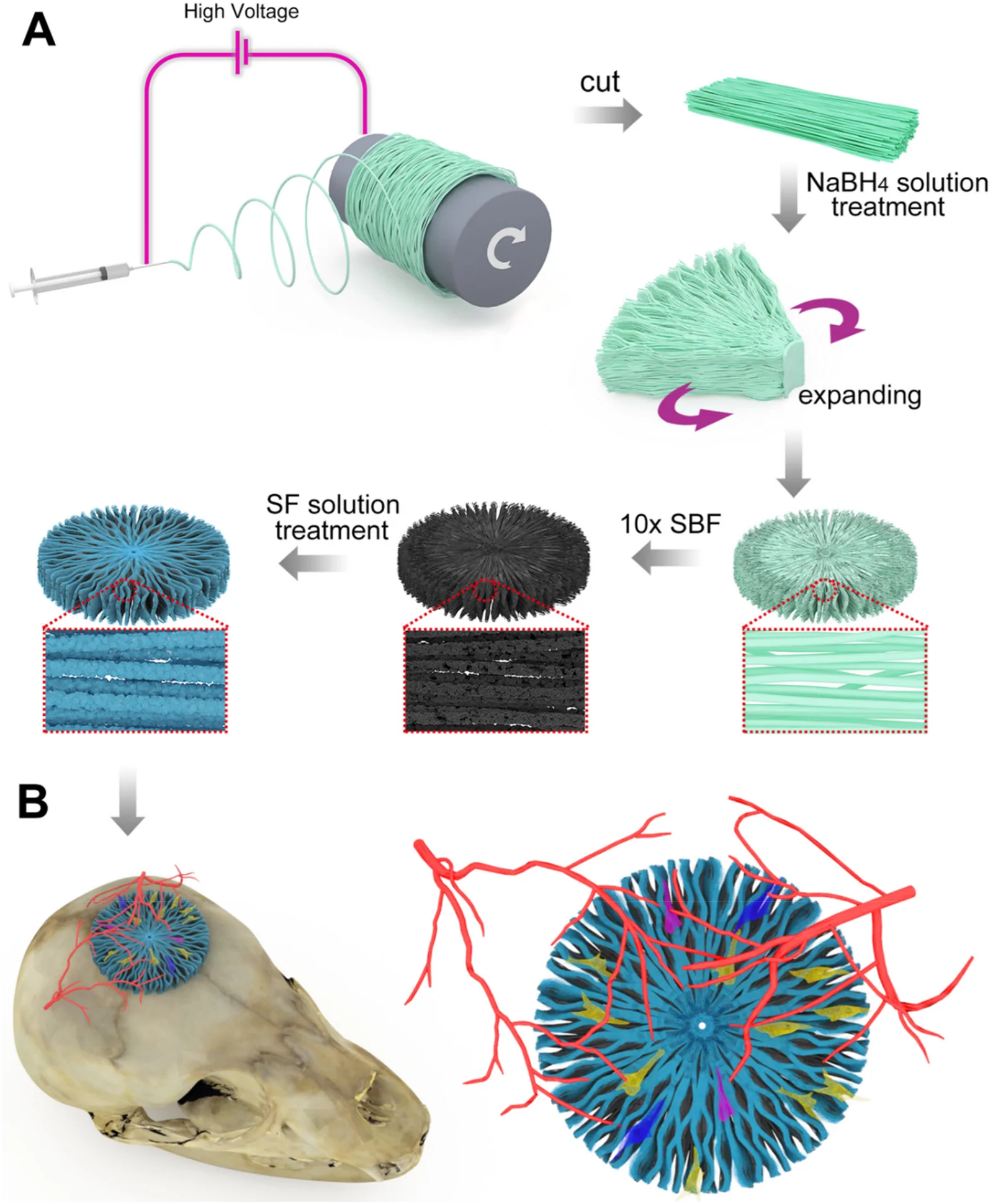
(A) Graphic demonstration of the 3D radially aligned NF fabrication process. (B) In vivo assessment using a rat cranial defect model, demonstrating the application of the scaffold in promoting bone remediation [258]. Copyright 2021. Reproduced with Permission from Elsevier.

#### 3.2.4 Layer-by-layer deposition

Layer-by-layer (LBL) assembly is a highly adaptable surface modification procedure that enables precise control over film thickness, spanning the nanometer-to-micrometer scale [259], [260], [261], [262], [263]. The method relies on the sequential attachment of negatively charged species to a substrate, resulting in a well-ordered multilayered coatings or freestanding films. Electrostatic attraction is the main driving force; however, additional interactions such as covalent and non-covalent bonding, metal-ligand coordination, and specific biomolecular recognition can also contribute to film assembly [264], [265], [266]. Between successive deposition steps, intermediate rinsing (and typically drying) was performed to remove loosely bound species and prevent cross-contamination, thereby preserving layer integrity. The total number of layers can be simply tuned by adjusting the amount of deposition cycles, while film features like composition, thickness, morphology, and porosity can be precisely adjusted by controlling solution parameters (e.g., concentration, pH, ionic strength) and processing conditions (e.g., temperature and deposition time) [267].

LBL assembly is widely regarded as an affordable, environmentally friendly technique that does not require harsh organic solvents or high-temperature processing. It offers exceptional tunability in terms of film architecture, porosity, and mass loading, and is compatible with various substrates and biomolecules due to its mild processing conditions [128], [268], [269]. In addition, its scalability and ability to incorporate multiple functional components render it highly appropriate for creating responsive, multifunctional materials. A rich selection of building blocks, including natural and synthetic polymers, proteins, lipids, DNA, NPs, and supramolecular complexes, can be integrated using this approach, allowing the design of innovative functional coatings for various application fields, like biomedicine, catalysis, energy storage, optics, and filtration [270]. Several LBL deposition strategies have been developed, including dip-coating (immersion), spin-assisted deposition, spray-assisted assembly, electromagnetic-assisted assembly, and microfluidic-based assembly [270]. Each technique offers distinct advantages and constraints regarding deposition speed, uniformity, structural control, and scalability, making method selection critical for application-specific design [271].

In biomedical engineering, LBL assembly has materialized as a powerful strategy for functionalizing electrospun NF membranes without significantly altering their intrinsic porosity or morphology. Its ability to uniformly coat complex 3D fibrous structures has enabled broad applications in TE, bone regeneration, controlled DD, wound healing, and biosensing [272], [273], [274], [275], [276], [277], [278]. For example, Ma et al. functionalized SF ESNFs using LBL deposition of CS and PDA, resulting in enhanced antibacterial activity and improved cytocompatibility [279]. Similarly, Zhou et al. constructed multifunctional antibacterial NFMs by combining ES and LBL deposition of quaternized chitin (QC) and tannic acid (TA), thereby achieving improved wettability, antibacterial performance, antioxidant activity, and hemocompatibility for wound-healing applications [280].

LBL assembly has also been intensively applied in BTE through biomimetic mineralization strategies. For example, Zheng et al. developed apatite-coated PCL NFs using LBL assembly of collagen and chondroitin sulfate followed by mineralization, resulting in enhanced osteogenic differentiation and in vivo bone formation. The fabrication procedure for biofunctional bone scaffolds using the LBL approach is depicted in Fig. 12 [281]. Similarly, Wang et al. constructed a tissue-engineered periosteum by integrating PCL, nano-hydroxyapatite (nHAp), and collagen with bone marrow-derived stem cells, promoting efficient osseointegration and reducing fibrotic encapsulation [282]. Further advancing multifunctional scaffold design, Cheng et al. developed core-sheath nanofibrous mats with spatially controlled delivery of BMP2 and CTGF using LBL functionalization, achieving temporally regulated growth factor release and enhanced bone regeneration outcomes [272]. In another study, PLLA NFs functionalized with CS, heparin, and graphene oxide via LBL assembly exhibited increased surface roughness, hydrophilicity, mechanical strength, and improved endothelial cell compatibility, while also reducing inflammatory responses [283].

**Fig. 12:**
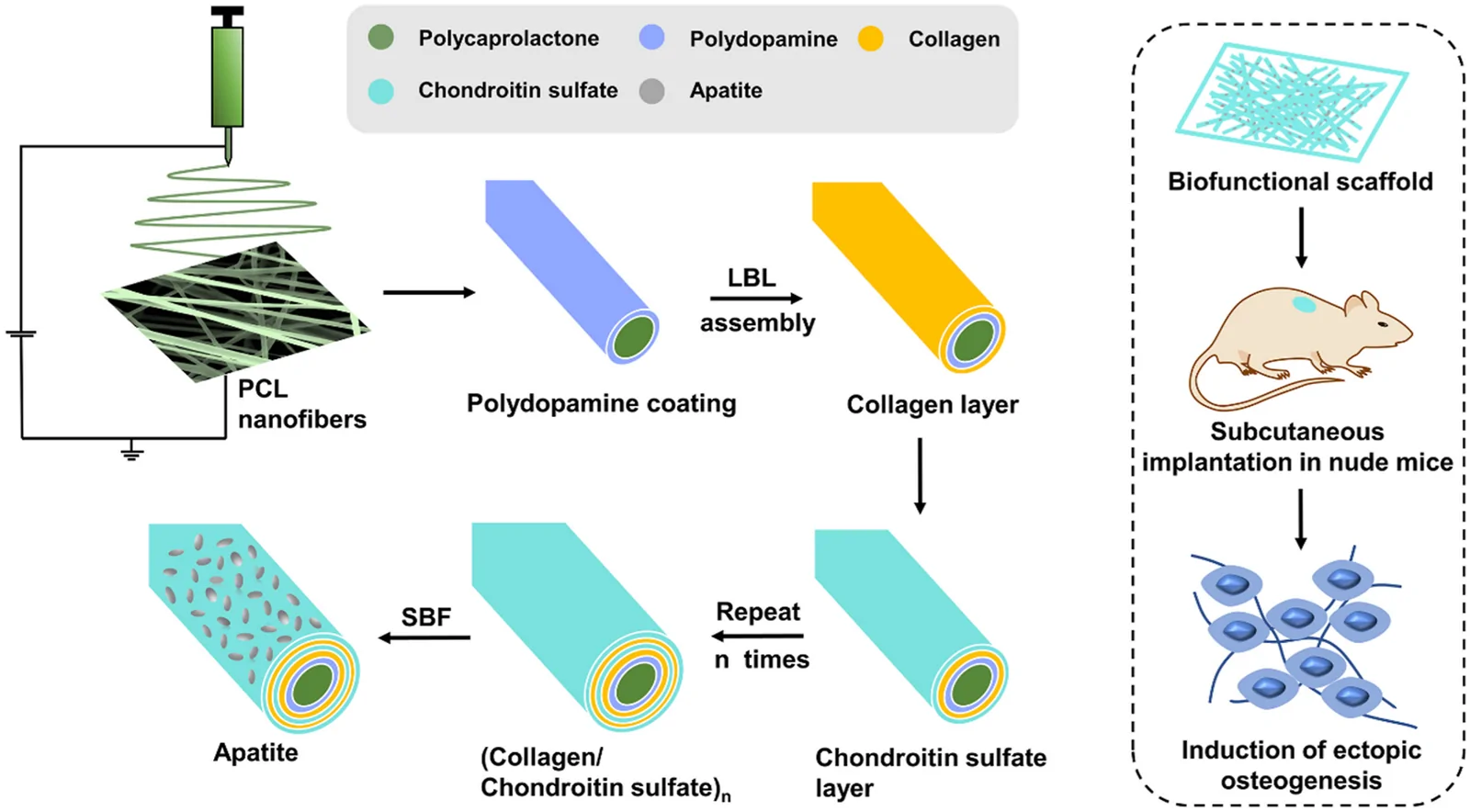
Fabrication process for biofunctional bone scaffolds: The strategy involves LBL self-assembly of bioactive molecules onto electrospun nanofibrous membranes, followed by biomimetic mineralization via apatite deposition to enhance osteoconductivity and promote bone tissue regeneration [281]. Copyright 2021. Reproduced with permission from Elsevier.

The aforementioned discussion of the different functionalization strategies for ESNFs emphasizes that no single strategy is universally superior; rather, each involves a balance among biological advantages, material performance, process complexity, and translational feasibility. Table 1 provides a concise comparative analysis of the major functionalization strategies discussed earlier in this manuscript, highlighting their key advantages, limitations, and most suitable biomedical applications. From a translational perspective, the most promising functionalization strategies should therefore be selected not solely on the basis of their ability to enhance biological performance, but also on whether the resulting improvements provide a meaningful therapeutic advantage that justifies the additional material, processing, manufacturing, and regulatory complexity.

**Table 1:** Comparative overview of functionalization strategies for ESNFs, highlighting functionalization outcomes, key strengths, major limitations/trade-offs, suitable biomedical applications and translation potential.

Functionalization strategy	Functionalization outcome	Key strengths	Major limitations/trade-offs	Most suitable biomedical applications	Translational potential/considerations	Ref.
**Aminolysis**	Introduces amine groups and other reactive sites onto the fiber surface	Simple and relatively rapid; increases surface reactivity; facilitates covalent immobilization of biomolecules	Harsh reaction conditions or excessive treatment may alter fiber morphology, molecular weight, mechanical properties, and degradation behavior; residual reagents must be thoroughly removed	Tissue engineering, wound healing, drug delivery, peptide/protein immobilization	**Moderate:** useful for surface functionalization, but control of reaction conditions, residual reagents, and reproducibility is important for scale-up	[34], [128], [129], [130], [133], [135], [140], [141], [142], [143]
**Hydrolysis**	Generates hydrophilic and reactive groups on polymer surfaces	Improves surface wettability and cell–material interactions; relatively straightforward; facilitates subsequent functionalization	Excessive hydrolysis may cause surface erosion, accelerated degradation, and reduced mechanical integrity	Tissue engineering, wound healing, drug delivery	**Moderate–High:** relatively simple processing and limited additional components; excessive treatment must be controlled to preserve mechanical and degradation properties	[34], [128], [129], [137], [151], [154], [155]
**Chemical grafting**	Covalent attachment of polymers, biomolecules, or functional groups to the fiber surface	Stable functionalization; enables tailored surface chemistry; reduced desorption compared with physical adsorption	Requires additional chemical reagents and processing; possible cytotoxicity from residual reagents; grafting density and uniformity can be difficult to control	Drug delivery, tissue engineering, biosensing, targeted delivery, antimicrobial scaffolds	**Moderate:** strong functional stability, but reagent selection, grafting uniformity, residual chemicals, and process complexity may complicate regulatory translation	[96], [129], [161], [166], [167], [168], [173], [174], [175], [235]
**Chemical crosslinking**	Forms covalent bonds between polymer chains and/or immobilizes functional molecules	Enhances mechanical and aqueous stability; improves retention of incorporated bioactive agents	Crosslinkers may be cytotoxic; residual reagents and degradation products are concerns; excessive crosslinking may reduce flexibility, porosity, and degradation rate	Wound healing, tissue engineering, protein/peptide delivery	**Moderate:** attractive where aqueous/mechanical stability is essential, but biocompatible crosslinkers and stringent residual-reagent control are critical	[34], [128], [129], [179], [180], [181], [182], [193], [194], [201], [207]
**Plasma treatment**	Activates the fiber surface and introduces polar/reactive functional groups	Rapid, solvent-free, and primarily surface-specific; improves wettability and provides reactive sites with limited bulk modification	Requires specialized equipment; strongly dependent on treatment parameters; surface effects may undergo aging; excessive treatment can damage fibers	Tissue engineering, wound healing, biosensing, cell-adhesive surfaces	**High–Moderate:** solvent-free and surface-specific, with relatively limited chemical burden; process uniformity and surface aging require consideration during scale-up	[34], [129], [200], [212], [213], [214], [215], [216], [217], [218], [219], [220], [221], [230]
**UV treatment**	Induces surface activation/oxidation and facilitates subsequent functionalization	Simple and relatively accessible; low chemical consumption; modifies surface chemistry without extensive wet processing	Limited penetration; excessive UV exposure may cause polymer degradation, embrittlement, and changes in mechanical properties	Tissue engineering, wound healing, antimicrobial surfaces, biomolecule immobilization	**Moderate–High:** accessible and relatively simple, but exposure control and potential polymer degradation must be carefully managed	[34], [231], [232], [233], [239], [240], [241], [242]
**Simple physical adsorption**	Non-covalent attachment/loading of functional molecules onto the fiber surface	Very simple, mild, and inexpensive; suitable for sensitive biomolecules; minimal chemical processing	Weak attachment; desorption and burst release may occur; limited control over molecular orientation and long-term stability	Drug delivery, growth-factor delivery, wound healing, protein immobilization	**High for selected applications:** minimal processing and chemical burden favor translation, but limited retention and long-term stability may restrict applications requiring durable functionalization	[34], [129], [167], [244], [245], [246], [247], [248], [254], [255]
**LBL deposition**	Sequential assembly of interacting/charged polymers or biomaterials into multilayer coatings	Precise control over coating composition and thickness; enables incorporation of multiple functional agents; highly versatile	Multiple processing steps increase manufacturing complexity; possible coating instability or delamination; scale-up can be challenging	Controlled drug delivery, antimicrobial coatings, tissue engineering, multifunctional scaffolds	**Moderate:** excellent control over multifunctionality, but the number of processing steps, coating uniformity, stability, and scale-up remain important barriers	[34], [129], [259], [260], [262], [267], [268], [272], [273], [274], [275], [276], [277], [278]

## 4 Biomedical applications of functionalized nanofibers

Nanofiber technology has rapidly emerged as a key focus in biomedical research, offering promising solutions to major clinical challenges, including wound and burn care, osteoporosis treatment, organ regeneration, and the management of various diseases [284], [285], [286]. ESNFs are particularly attractive due to their exceptionally high surface area relative to bulk materials, which improves the adhesion of cells, proteins, and therapeutic agents. Additionally, these NFs can be assembled into complex 3D architectures, enabling their use in diverse biomedical applications [284], [285], [286], [287], [288]. The large interfacial surface of NFs contributes to improved cellular interactions, enhanced drug loading volume, and tunable drug release profiles. Furthermore, optimization of ES parameters, such as polymer type, solution properties, and fiber alignment, allows accurate modulation of cell differentiation and DD behavior. Consequently, the combination of high surface area, mechanical elasticity, and adaptable surface chemistry makes ESNFs highly valuable in biomedical applications [284], [285], [286], [287], [288], [289], [290], [291]. Importantly, the properties of ESNFs can be further tailored through surface functionalization using various chemical groups and bioactive molecules. Such modifications significantly influence both physicochemical and biological characteristics. For instance, surface treatments can alter wettability, thermal features (including melting and glass transition temperatures), and overall stability of the NFs [43], [285], [289]. Simultaneously, biological properties such as biocompatibility, cell adhesion, and cell proliferation can be markedly enhanced. A wide range of materials (polymers, metal and magnetic nanoparticles (MNPs), carbon-based nanomaterials, functional groups, proteins, and peptides) have been employed for nanofiber surface modification [43], [285], [289]. These innovations have led to extensive integration of functionalized ESNFs across domains such as regenerative medicine, TE, wound healing, targeted DD, biosensing, and cell imaging. Overall, surface-functionalized ESNFs represent a powerful tool for next-generation biomedical technologies. The following sections will discuss their applications in greater detail across various biomedical domains.

### 4.1 Tissue engineering and regenerative medicine

TE and regenerative medicine sit at the intersection of biology, clinical medicine, and materials science, with the primary goal of restoring or replacing damaged tissues and organs. Given the structural intricacy of the human body and the interdependence of its tissues, biomaterials should be carefully designed to ensure mechanical integrity, biocompatibility, and chemical compatibility with the physiological microenvironment. In this context, NFs have emerged as a critical class of biomaterials due to their structural resemblance to the ECM, which enables dynamic cell-material interactions and supports tissue regeneration [292], [293], [294]. For instance, Zheng et al. [295] developed functionalizable nanostructured scaffolds by combining PCL with 4-dibenzocyclooctynol via chemical polymerization. This strategy enabled the synthesis of adaptable arrangements suitable for various TE purposes. Their design leveraged the interplay between sol and gel states, emphasizing the importance of rheological transitions from injectable sol phases to mechanically stable gel networks. Similarly, Doench et al. [296] engineered a nanostructured system based on nanofibrillated cellulose for nucleus pulposus regeneration. In vivo studies using porcine and rabbit spinal models demonstrated that injectable cellulose nanofibril/CS formulations improved biomechanical performance of intervertebral discs, highlighting their potential in spinal tissue repair.

A crucial aspect of successful tissue regeneration is the promotion of angiogenesis, particularly in hard-tissue repair such as bone regeneration. Kuttappan et al. [297] demonstrated that ESNF membranes can serve as carriers for the localized and controlled delivery of key signaling molecules like vascular endothelial growth factor (VEGF), fibroblast growth factor (FGF), and BMP-2. These biomolecules perform complementary roles: VEGF stimulates vascularization in MSCs, BMP-2 promotes osteogenic differentiation [298], and basic FGF (bFGF) enhances endothelial cell migration and proliferation, particularly in HUVECs [299]. The controlled and sequential release of these factors from NF-based systems was shown to substantially accelerate the formation of vascularized bone tissue, a major objective in advanced regenerative therapies.

Cell-material interactions are central to scaffold performance and directly influence cellular behavior. Cho et al. [145] investigated the metabolic outcome of cells cultured on different NF systems (aNFs, HA0@NFs, and HA5@NFs) using WST-1 assays. While aNFs initially supported higher cell viability during the first three days, a decline was observed by day 5, likely due to cell overcrowding and the scaffold's structural instability as depicted in Fig. 13a [145]. In contrast, HA-modified NFs, particularly HA5@NFs, maintained superior cell viability, attributed to HA's stabilizing effect. DNA quantification further confirmed enhanced cell proliferation on HA-functionalized NFs, with HA5@NFs showing the highest proliferation rates throughout the culture period (Fig. 13b) [145]. In addition to proliferation, HA functionalization significantly influenced cell morphology as depicted in Fig. 13c [145]. NIH 3T3 fibroblasts cultured on HA-modified NFs exhibited a more elongated morphology with enhanced actin filament organization, whereas cells on unmodified NFs displayed a rounded shape with weaker cytoskeletal development [145]. These findings highlight the importance of surface-mediated cell adhesion, particularly for anchorage-dependent cells such as fibroblasts. Enhanced ECM protein adsorption on HA-functionalized surfaces further enhances cell adhesion and proliferation [300]. These results are consistent with previous studies demonstrating that HA incorporation promotes cytoskeletal organization and strengthens cell-material interactions, underscoring the critical role of ECM-mimetic cues in TE applications [301].

**Fig. 13:**
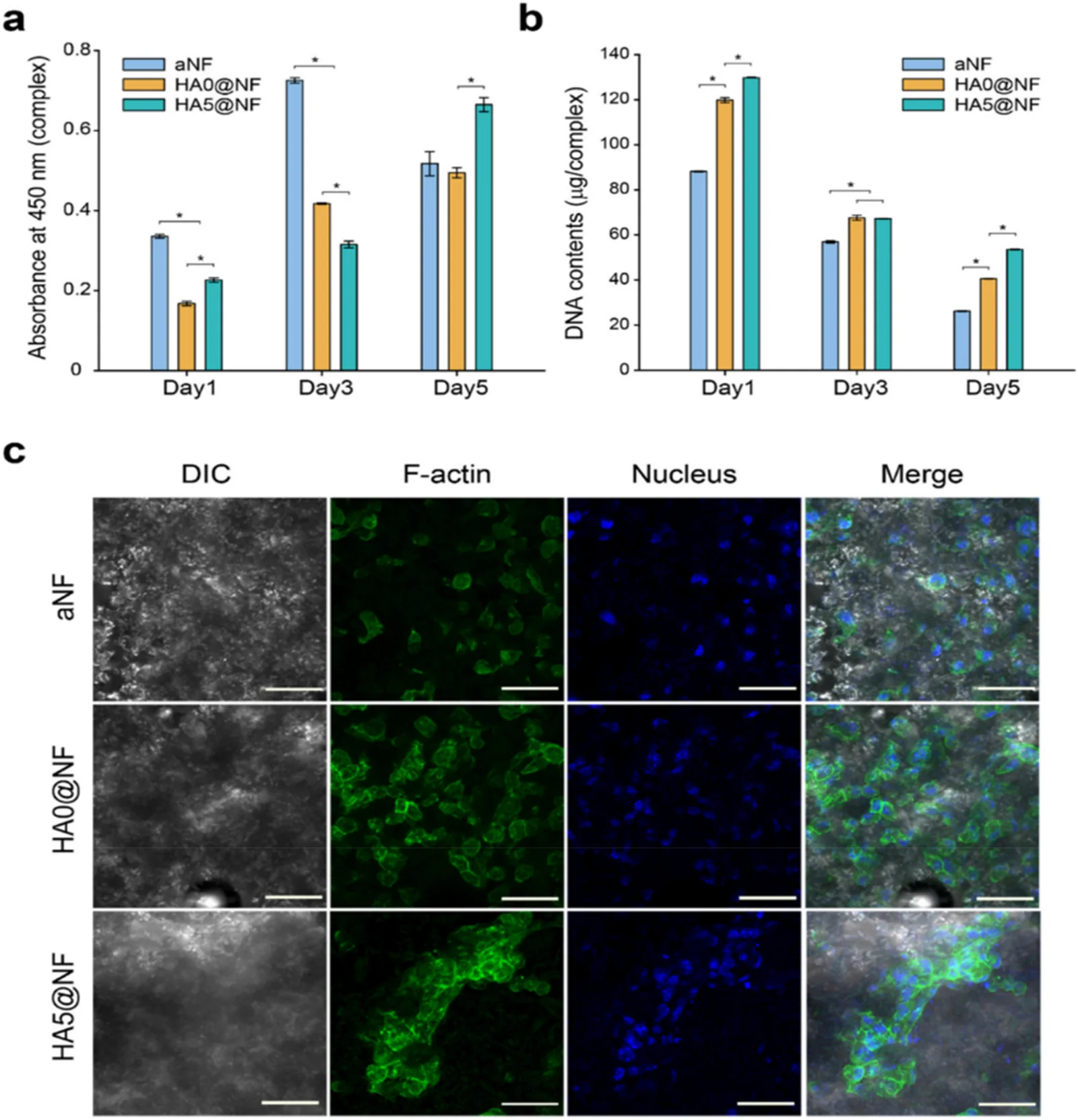
Proliferation and DNA content of NIH3T3 cells cultured within cell/nanofiber (NF) Formulations. (a) Cell proliferation assessment via WST-1 assay for treatments of 1, 3, and 5 days. (b) DNA content quantified using the PicoGreen assay. (c) Confocal laser scanning microscopy (CLSM) photographs of cells combined with NF, HA0@NF, and HA5@NF after 72 h of cultivation. F-actin was marked with Alexa Fluor 488 Phalloidin and cell nuclei was marked with 4′,6-diamidino-2-phenylindole (DAPI). Scale bar depicts 50 μm. Statistical significance is showcased by asterisks (*p < 0.05) [145]. Copyright 2022. Reproduced with permission from the American Chemical Society.

Nanofibrous scaffolds can be specifically engineered for targeted clinical applications, including ocular wound healing. Yan et al. [42] designed a PLA electrospun scaffold functionalized with silver nanoparticle/cellulose nanofiber (AgNPs/CNF) hybrids to create a multifunctional system with enhanced antibacterial and cell-supportive properties. The fabrication process of PLA-based electrospun fiber mats (EFMs) incorporating CNF and AgNPs is illustrated in Fig. 14 [42]. This study represents an early effort to design ocular bandages with intrinsic antimicrobial activity, potentially reducing the need for repeated topical antibiotic administration. In this system, cellulose NFs (CNFs) served as anchoring platforms for silver NPs at very low loading levels, simultaneously improving scaffold hydrophilicity and imparting antibacterial functionality. In vitro co-culture studies demonstrated excellent biocompatibility with ocular epithelial cells, with CNF-coated scaffolds significantly enhancing cell proliferation. The scaffolds exhibited strong antimicrobial activity, achieving over 95% inhibition against *E. coli* and *S. aureus*, as well as notable antifungal activity against *Fusarium* species. Although silver NPs are often associated with cytotoxicity concerns [302], [303], the modified scaffolds (PLA-1 and PLA-2) maintained normal cell morphology and proliferation comparable to pure PLA, indicating that the AgNP concentrations used were within a safe range. Live/dead staining assays (Fig. 15a1-a4 and 15b1-b4) revealed minimal cell death, further confirming the non-toxic nature of the scaffolds [42].

**Fig. 14:**
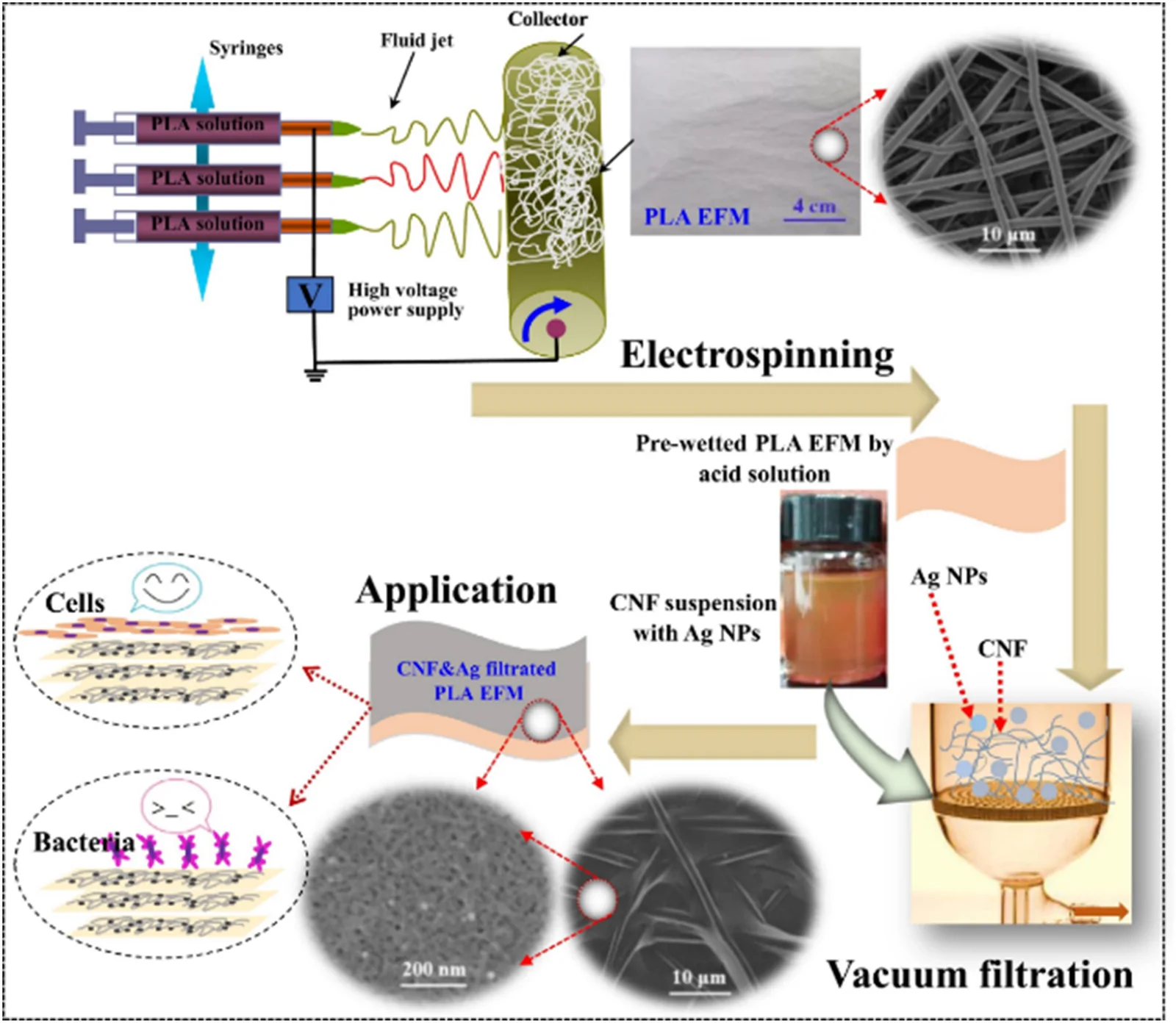
Schematic showing the ES process and physicochemical characterization of PLA-CNF-AgNP EFMs [42]. Copyright 2020. Reproduced with permission from Elsevier.

Effective ocular bandages must also promote rapid wound healing by facilitating cell movement and proliferation [304]. Consistent with this requirement, BrdU-based proliferation assays (Fig. 15c1-c4 and 15d1-d4) showed a higher proportion of proliferating cells on PLA-1 and PLA-2 scaffolds compared to tissue culture polystyrene (TCPS) and non-modified PLA. Both conjunctival epithelial cells (CjECs) and corneal epithelial cells (CECs) exhibited the highest viability on the modified scaffolds, confirming their aptitude to support cell growth. Enhanced cell viability over 48 and 72 h further highlighted the beneficial effect of CNF incorporation on scaffold cytocompatibility. This improvement was primarily attributed to enhanced surface hydrophilicity, as demonstrated by a reduced water contact angle, which created a more beneficial environment for cell adhesion and proliferation. Additionally, CNF incorporation contributed to collagen-like fibrillar features within the scaffold, which may further influence cellular responses [305]. Overall, the combined mechanical and biological enhancements of these PLA-based nanofibrous scaffolds underscore their strong potential as ocular wound-healing materials. The incorporation of CNF promotes epithelial regeneration, while AgNPs provide effective antimicrobial protection, making these scaffolds promising candidates for advanced ocular therapies [42].

**Fig. 15:**
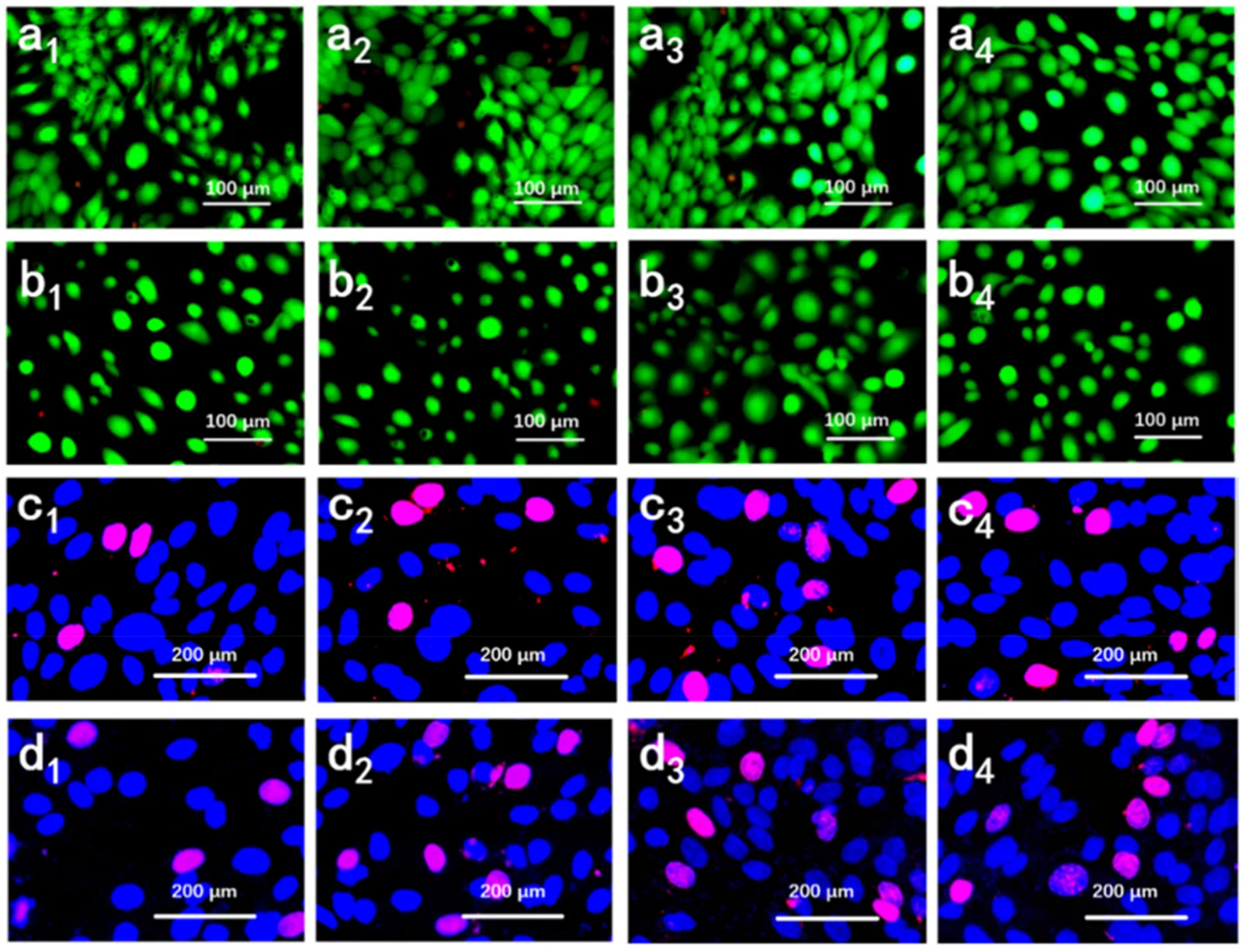
Assessment of cell viability and proliferation for CjCEs and CECs cultured on (a1-d1) TCPS and PLA-based EFMs: (a2-a4) PLA, (b2-b4) PLA-1, and (c2-c4) PLA-2. Representative live/dead staining images reveal viable cells in green and non-viable cells in red for conjunctival (a1-a4) and corneal (b1-b4) epithelial cells grown on the different EFMs [42]. Copyright 2020. Reproduced with permission from Elsevier.

In the pursuit of multifunctional scaffolds for bone repair, Cheng et al. [272] developed core-sheath structured SF/PCL/PVA NF constructs using coaxial ES combined with LBL assembly, as shown in Fig. 16. In this design, BMP-2 (10 μg/mL) was integrated into the nanofiber core. In contrast, connective tissue growth factor (CTGF, 10 μg/mL) was immobilized on the surface, enabling a double DD construct with temporally controlled release profiles. Both in vitro and in vivo studies demonstrated that the dual-functioning system successfully promoted osteogenesis and angiogenesis. A sequential release behavior was observed: BMP-2 exhibited a sustained, nearly linear release over 30 days, while CTGF showed a relatively faster release over approximately 40 days. When implanted into cranial defects of mice, the BMP-2/CTGF-loaded SF/PCL (1:5)/PVA-LBL20 scaffolds achieved extensively enhanced bone formation (∼43%) as opposed to structures delivering BMP-2 alone. After eight weeks, the dual-delivery system maintained the highest bone volume and area, along with increased expression of angiogenic indicators such as VEGF and CD31. These findings highlight the effectiveness of time-controlled release enabled by core-sheath NF architectures in coordinating osteogenic differentiation and vascularization, thereby accelerating bone regeneration and microvascular development [272]. In a complementary strategy, Oliveira et al. [306] developed injectable composite scaffolds based on organically modified glass (ormoglass) particles enriched with calcium and phosphorus (CaO: P2O5:Na2O: TiO_2_ molar ratio of 44.5:44.5:6:5). These particles were synthesized via a partially hydrolyzed alkoxide sol-gel process, subsequently coated with PLA fibers, and uniformly dispersed in a 40% (w/v) hydroxypropyl methylcellulose (HPMC) hydrogel matrix. Upon implantation into the femoral condyles of Wistar rats, the scaffolds promoted both angiogenesis and bone regeneration within 3-6 weeks. This outcome was primarily attributed to the controlled release of Ca^2+^, which plays a critical role in bone remodeling and vascularization [306].

**Fig. 16:**
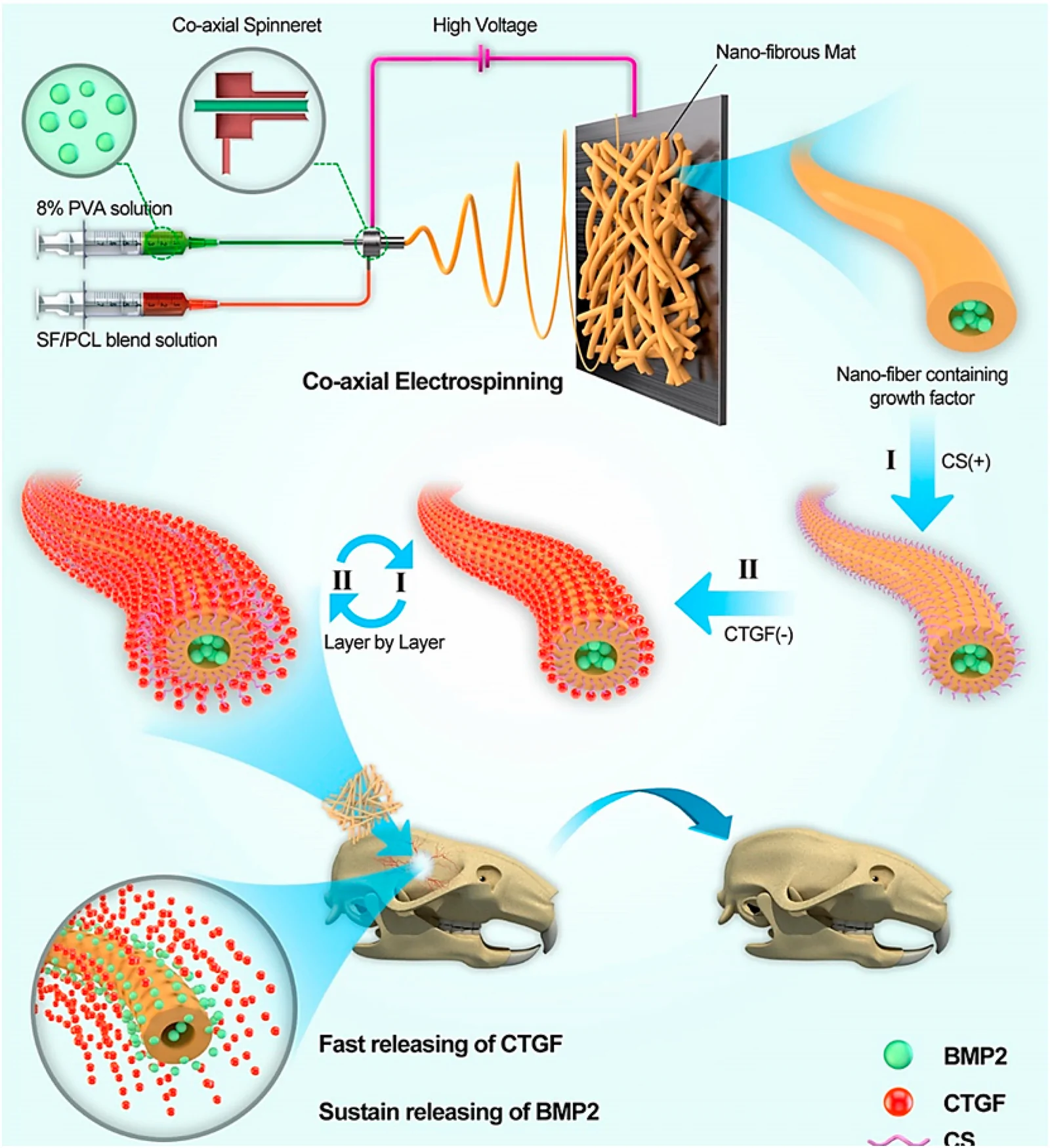
Depiction of coaxial (SF/PCL 1:5)/PVA-LBL20 ESNFs encapsulating BMP-2 and CTGF for BTE. By combining coaxial ES and LBL assembly, BMP-2 was integrated into the fiber core, while CTGF was immobilized on the fiber surface. The sustained BMP-2 release and the transient CTGF release enable stage-specific biological activity during bone healing, supporting coordinated tissue regeneration [272]. Copyright 2019. Reproduced with permission from the American Chemical Society.

Bone, a dense connective tissue, plays a fundamental role in sustaining structural integrity and supporting vital organs [307]. However, traumatic injuries, infections, and pathological conditions often result in bone defects that are difficult to repair using conventional grafting techniques [308]. Consequently, BTE has become a multidisciplinary field that combines biomaterials, cells, and bioactive signaling molecules to restore bone function more effectively than traditional grafts or implants [309]. Among the various biomaterial strategies, nanofibrous scaffolds and membranes functionalized with bioactive agents have shown significant promise. Their structural similarity to the native ECM offers an ideal microenvironment for cell adhesion, proliferation, and differentiation [310], [311]. In particular, 2D NFMs are designed not merely to fill bone defects but to guide and regulate new bone tissue formation in a controlled manner [293]. Beyond bone regeneration, nanofibrous systems have also demonstrated considerable potential in VTE. Jia et al. [312] investigated tissue-engineered blood vessels (TEBVs) fabricated from collagen and chondroitin sulfate-collagen (ChS-COL) NFs, which were implanted into unilateral carotid arteries of New Zealand white rabbits (Fig. 17A-B). Of the six animals used in the study, one animal died due to postoperative complications. At the same time, the remaining grafts were explanted after 10 days for evaluation of early endothelialization and thrombosis. SEM analysis revealed that collagen-based TEBVs exhibited extensive blood clot formation, indicating significant thrombosis (Fig. 17C) [312]. Histological assessments, including hematoxylin-eosin and Masson's trichrome staining, further confirmed lumen occlusion in these grafts, characterized by dense platelet aggregation and the absence of a functional endothelial layer (Fig. 17D-E) [312]. In contrast, ChS-COL nanofiber-based grafts displayed a smooth, thrombus-free lumen. They were covered by a uniform monolayer of endothelial-like cells expressing PECAM and ICAM markers, confirming successful endothelialization (Fig. 17F) [312]. These results emphasize a critical role of surface composition and biofunctionalization in modulating blood compatibility and cellular responses, underscoring the significant potential of nanofibrous scaffolds in both BTE and VTE.

**Fig. 17:**
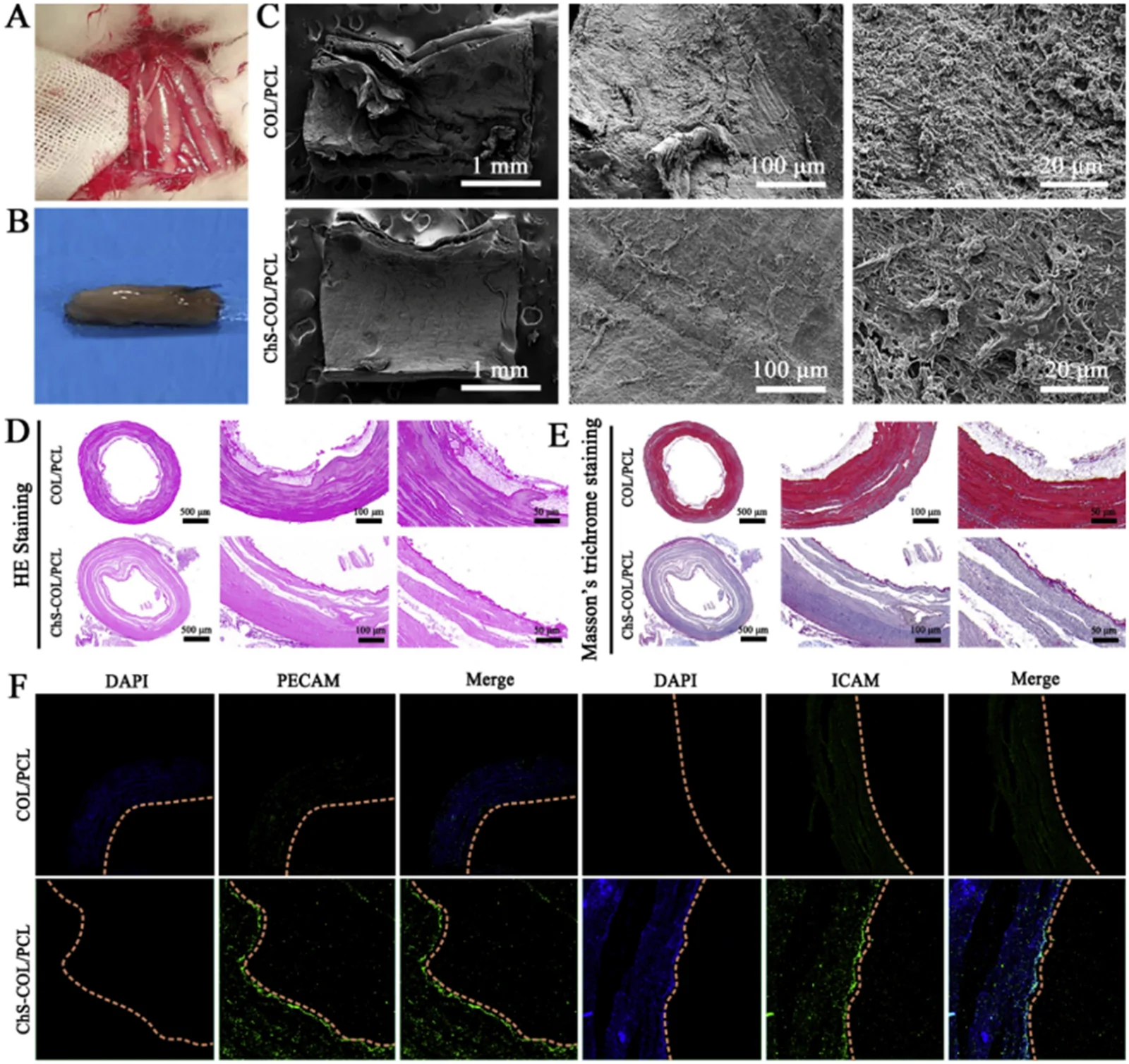
TEBV implantation, histological staining, and endothelial marker expression [312]. Copyright 2021. Reproduced with permission from Elsevier.

Electrospun SF/Poly(L-lactide-co-ε-caprolactone) (SF/PLCL) scaffolds have demonstrated significant potential in promoting bone tissue regeneration. When cultured with human adipose-derived stem cells (hADSCs), SF/PLCL (50:50) scaffolds exhibited an optimal tensile strength of about 6 MPa and an enhanced ALP activity (∼150 absorbance units), compared to ∼80 for pure PLCL, indicating improved osteogenic differentiation [313]. Similarly, Zhu et al. [314] incorporated adenoviral vector-encoded BMP-2 into electrospun PLGA NFs with fine diameters (∼140 ± 40 nm), which supported bone marrow stromal cell adhesion and proliferation. After 14 days, the expression of key osteogenic markers, including collagen type I, Runx2, and ALP, was markedly increased. In another study, Dhand et al. [315] developed collagen-based ES scaffolds incorporating calcium and catecholamines, resulting in mechanically robust constructs (Young's modulus ∼675.8 ± 81 MPa) that closely simulate the configuration of natural bone. These scaffolds significantly enhanced osteoblastic differentiation, with ALP activity per cell reaching 0.12 on day 14 compared to 0.075 for the tissue culture plastic (TCP) control. Karaman et al. [316] further demonstrated that MSCs cultured on PLGA NFs functionalized with glutamate-rich peptides retained osteoblast-like morphology under osteogenic conditions, confirming enhanced differentiation. Osteogenic differentiation is a multistep process involving cell adhesion, proliferation, ECM deposition, and mineralization. While synthetic scaffolds are typically bioinert, they can be rendered osteoinductive by incorporating ECM-mimetic peptide motifs that activate relevant cellular signaling pathways [317]. The evaluation of osteogenesis commonly relies on markers such as ALP activity, Ca^2+^ accumulation, and osteogenic protein expression [318], [319]. Consistent with this, immunofluorescence analyses after 28 days of culture revealed that hMSCs grown on ASP-functionalized NFs exhibited significantly higher expression of collagen type I (COL-I), OP, and OC compared to those cultured on GLU-functionalized or unmodified NFs [225]. These findings suggest that while both ASP and GLU peptides promote osteogenic differentiation, ASP exerts a more pronounced inductive effect.

Beyond regeneration, nanofibrous systems are also being investigated for the treatment of bone-related cancers such as osteosarcoma (OS), where surgical resection often results in large, difficult-to-repair defects. Conventional allogeneic grafts are associated with risks including infection, immune rejection, and disease transmission (e.g., HIV, hepatitis) [320], [321]. To address these challenges, current strategies focus on integrating therapeutic agents into biodegradable polymeric systems, such as hydrogels, microspheres, NPs, and electrospun fibers, to create multifunctional implants [322]. Electrospun membranes are particularly attractive due to their increased interfacial area, drug-loading capacity, and ECM-mimicking structure. The development of coaxial ES has enabled the fabrication of core-sheath NFs capable of dual or sequential drug release. For example, He et al. [323] engineered a core-sheath membrane with naringenin-loaded PVP in the core and metronidazole-loaded PLGA in the shell, achieving both rapid and sustained drug release. However, single-drug systems often fall short in addressing the multifaceted requirements of cancer therapy [324], [325]. To overcome this limitation, Cao et al. [326] elaborated a multifunctional coaxial nanofibrous membrane co-loaded with DOX, nHAp, and ICA, a flavonoid known to promote osteogenesis. In this system, DOX and nHAp were incorporated into the shell, while ICA was embedded in the core, enabling sequential release to achieve combined antitumor and bone regenerative effects.

Building on this concept, Liu et al. [202] demonstrated that such composite membranes possess sufficient flexibility to conform to irregular bone defects, making them proper for guided bone regeneration (GBR) applications (Fig. 18). Their biological evaluations revealed strong cytotoxic effects against MG-63 OS cells, particularly in higher DOX-loading formulations (2% and 4% DHGIP), as evidenced by extensive cell death (Fig. 19A). The two-stage release mechanism (i) initial diffusion of DOX followed by (ii) gradual release from nHAp ensured sustained antitumor activity. Importantly, the same scaffolds supported the viability and proliferation of MC3T3-E1 pre-osteoblasts, with relative growth rates exceeding 90% in most formulations and surpassing 95% in lower DOX-content samples after 168 h. Morphological analyses using SEM and fluorescence imaging confirmed healthy, elongated cell morphology and strong adhesion to the nanofibrous matrix (Fig. 19B-C). Overall, these findings demonstrate that multifunctional electrospun nanofibrous scaffolds can simultaneously inhibit tumor growth and promote bone regeneration, offering a promising strategy for GBR following OS resection [202].

**Fig. 18:**
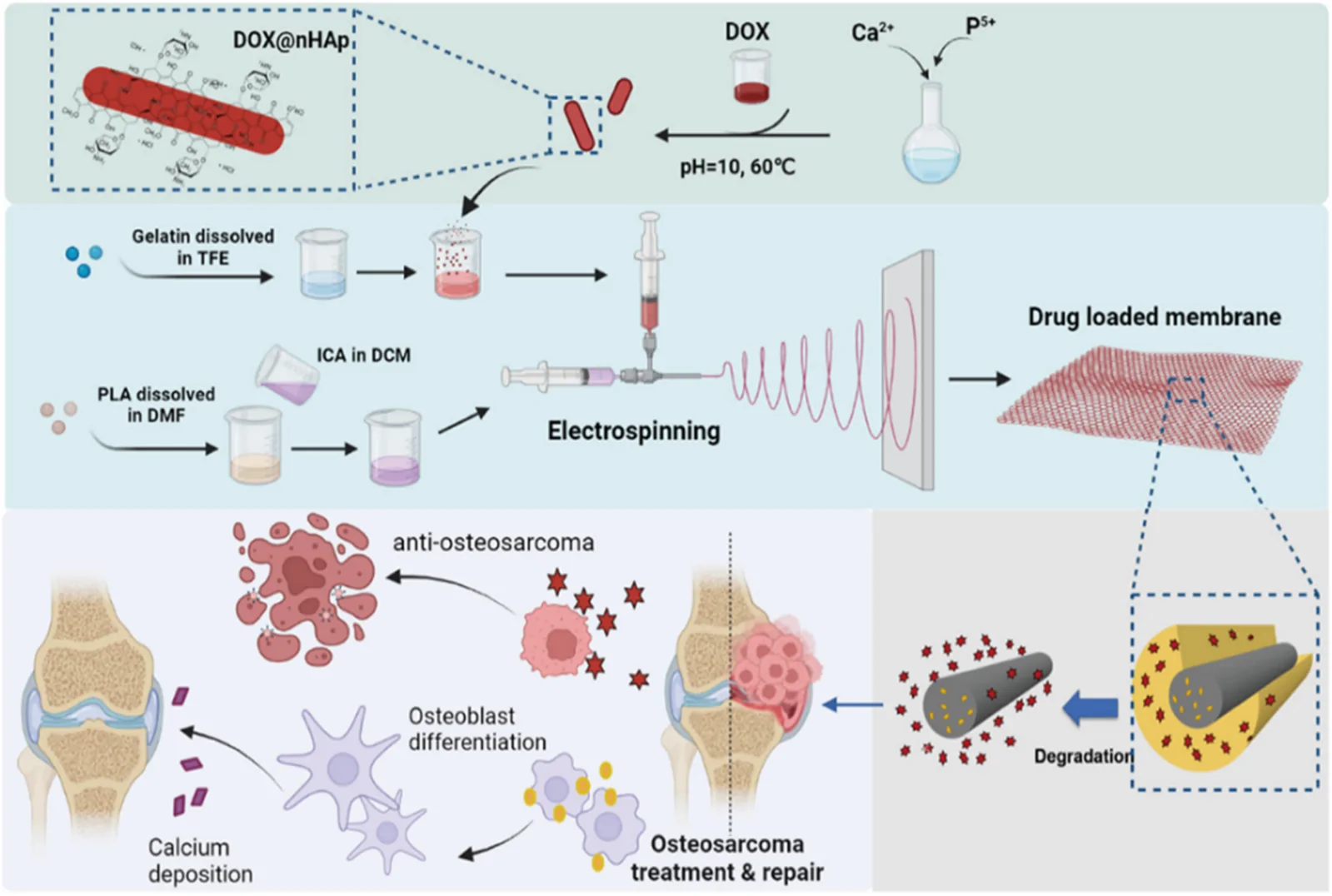
Schematic of OS-resection defect and coaxial dual-drug fiber design [202]. Copyright 2023. Reproduced with permission from Elsevier.

**Fig. 19:**
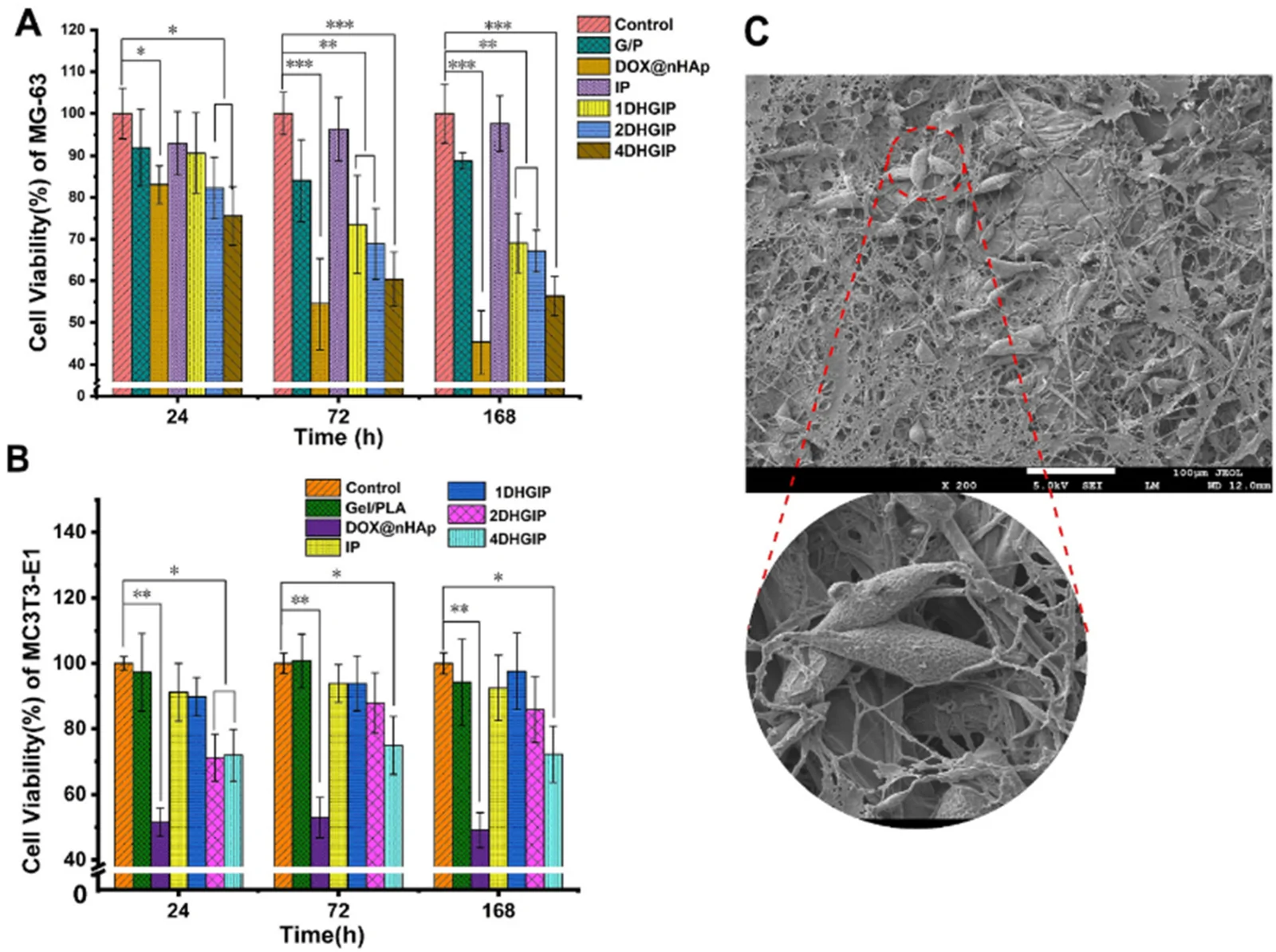
Viability assessment (MG-63 and MC3T3-E1), fluorescence microscopy, and SEM of cell adhesion [202]. Copyright 2023. Reproduced with permission from Elsevier.

Building on the incorporation of bone-mimetic inorganic phases, Gao et al. [327] co-electrospun nHAp platelets into PCL NFs, resulting in enhanced osteoconductivity and mechanical stiffness. These composite structures promoted osteogenic differentiation of hMSCs in vitro and significantly improved in vivo bone formation. Similarly, hydroxyapatite (HA)/poly (butylene adipate-co-terephthalate) NFs were shown to support the differentiation of hADSCs while also improving mechanical performance, with increases in elastic modulus (12.7%), stress at break (25.7%), and elongation at break (52%) [328]. Ko et al. [329] further advanced this strategy by functionalizing electrospun SF scaffolds with HA distributed throughout both the inner and outer regions, thereby markedly accelerating mineralized tissue formation in a critical-sized calvarial defect model. In another approach, CaP deposition onto hydroxyethyl cellulose/PVA (HEC/PVA) NFs significantly improved osteoconductivity, increasing the elastic modulus from 355 ± 10 MPa to 412 ± 12 MPa [330]. Additionally, magnetically functionalized organic-inorganic hybrid NFs have emerged as promising multifunctional scaffolds, offering enhanced osteogenic potential and antimicrobial properties, thereby broadening their applicability at implantation sites [285].

Beyond BTE, nanofibrous scaffolds have also shown promise in cartilage regeneration. In one study [213], cartilage-oriented constructs were evaluated histologically to assess chondrocyte morphology, cell distribution, and ECM formation. After 14 days, PLLA NFMs predominantly exhibited spindle-shaped, fibroblast-like cells, whereas chondroitin sulfate-grafted PLLA (CG-PLLA) NFMs displayed round or oval cells characteristic of chondrocytes. Alcian blue and Safranin O staining confirmed the existence of sulfated proteoglycan-rich ECM in both systems; however, CG-PLLA NFMs showed more intense and uniform staining, indicating higher glycosaminoglycan (GAG) accumulation and the formation of lacunae-like structures resembling native hyaline cartilage. Further in vivo analysis compared unseeded control CG-PLLA NFMs with cell-seeded constructs using histological staining methods, including hematoxylin and eosin (H&E), Alcian blue, and Safranin O (Fig. 20a–g) [213]. Control samples showed minimal tissue infiltration and only a slight inflammatory response, suggesting limited host interaction. In contrast, by week 4, cell-seeded constructs exhibited rounded cells maintaining a chondrocyte phenotype, along with abundant pericellular GAG deposition, as evidenced by Alcian blue and Safranin O staining. Immunohistochemical investigation further confirmed cartilage-specific matrix formation, with positive staining for type II collagen observed throughout the CG-PLLA scaffolds (Fig. 20h) [213]. Overall, these discoveries reiterate the versatility of ESNFs structure in both BTE and CTE, where the incorporation of inorganic phases and biofunctional modifications can significantly enhance mechanical performance, cellular response, and tissue-specific regeneration.

**Fig. 20:**
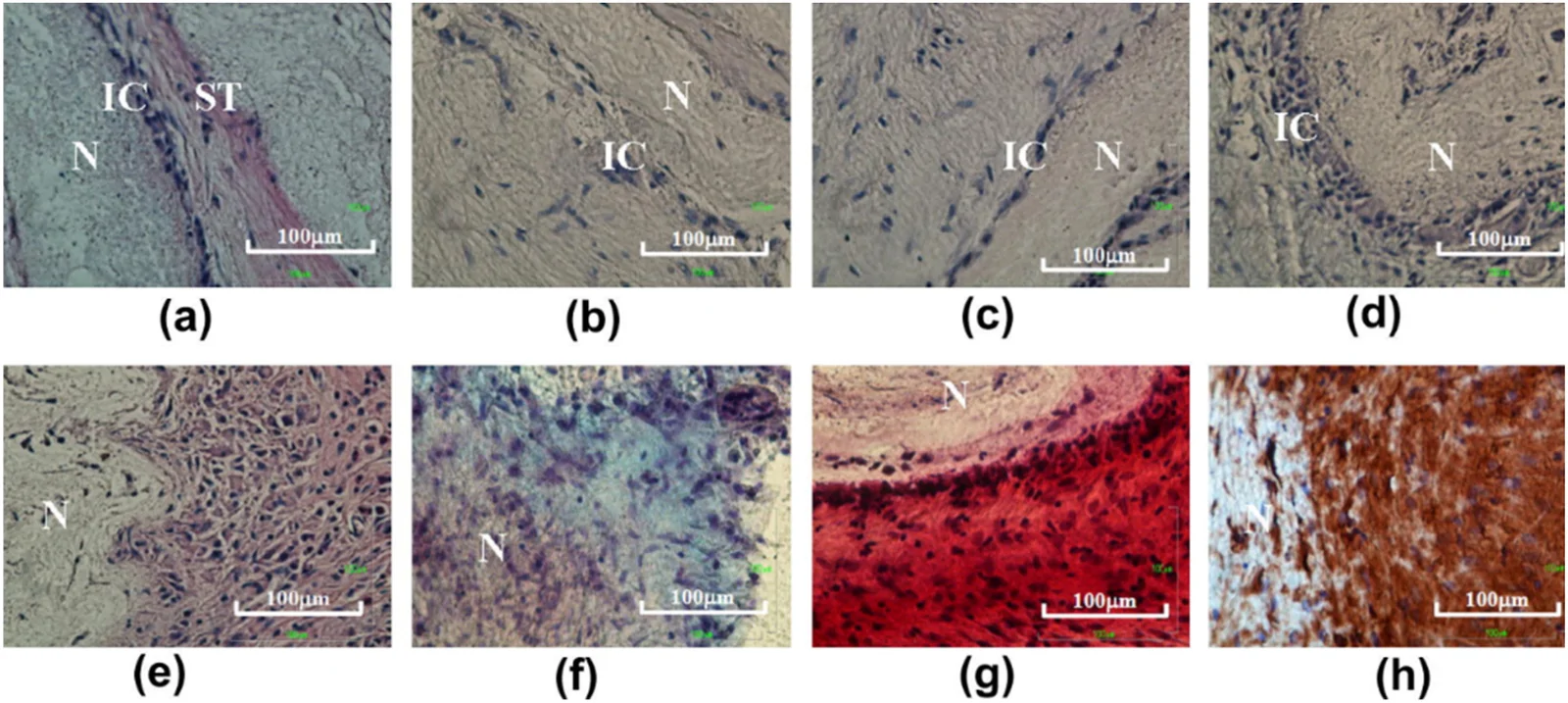
Four-week post-implant histology showing collagen II staining [213]. Copyright 2011. Reproduced with permission from Elsevier.

From a broader regenerative medicine perspective, replacing allografts or xenografts requires biomaterials that do more than simply avoid adverse host responses; they must actively regulate cell-material interactions in a manner that recapitulates the native ECM, while maintaining appropriate biodegradation kinetics, mechanical integrity, and mass transport properties. ES has therefore emerged as a key fabrication strategy, enabling precise control over fiber diameter, alignment, and porosity to mimic the fibrillar architecture of ECM across a range of tissues, including bone, skin, and nerve [331], [332]. However, many synthetic polymer-based scaffolds inherently lack bioactive surface cues [333], necessitating extensive surface modification to introduce cell-recognizable ligands and biomolecules [128]. The primary objective of such modifications is to promote integrin-mediated adhesion through defined biochemical motifs, thereby enhancing the survival, proliferation, and function of both transplanted and infiltrating cells [334]. For example, dextran grafted biodegradable PCL NFs were studied for TE applications [335]. Gelatin, a commonly used bioactive coating, was also applied to PCL NFs to improve endothelial cell spreading and proliferation, enhance cell orientation, and support the development of new materials for blood vessel engineering [336], [337]. Another example involves the use of mussel-inspired PDA coatings to convert hydrophobic nanofibrous surfaces into highly cell-adhesive substrates [338]. The authors experimentally validating the finding that a conformal PDA coating on 3D PCL nanofibrous meshes significantly enhances endothelial cell adhesion (Fig. 21A). In their approach, PCL NFs were fabricated via ES and subsequently immersed in an aqueous dopamine (DA) solution, where in situ oxidative polymerization led to the formation of a uniform PDA layer. Morphological analysis revealed that all scaffolds retained a non-woven nanofibrous architecture with a highly porous structure suitable for nutrient transport and ECM mimicry [338]. SEM imaging confirmed that fiber diameters were comparable across the various treatment groups, indicating that macroscopic fiber diameter alone is insufficient to confirm successful PDA deposition (Fig. 21B) [338]. Nevertheless, further analysis divulged that the PDA layer formed a uniform coating with an approximate width of ∼40 nm after 16 h of incubation, consistent with previously reported values [339], [340].

**Fig. 21:**
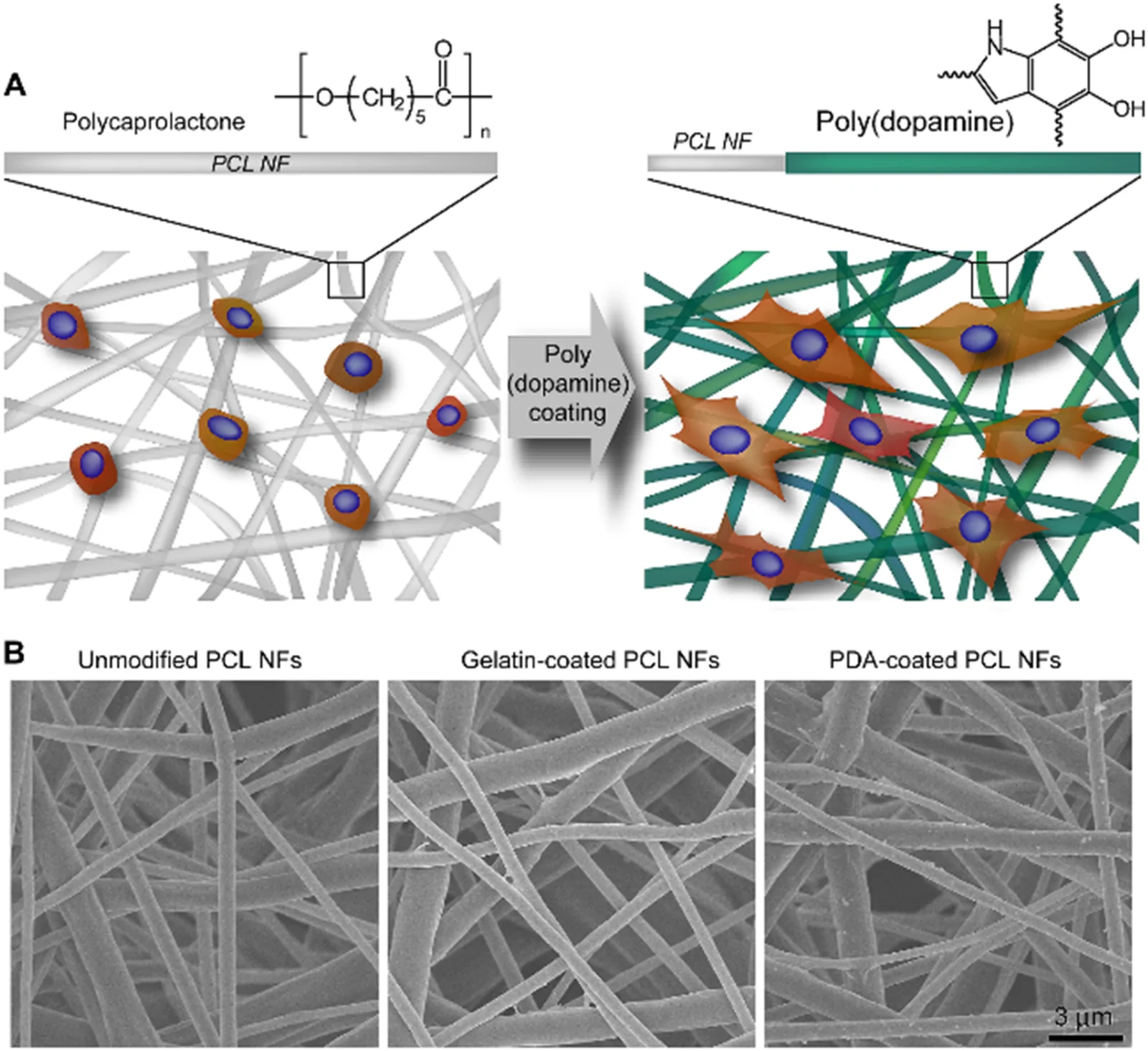
(A) Scheme showing cell attachment on PCL NFs coated with PDA. (B) SEM scans [338]. Copyright 2010. Reproduced with permission from Elsevier.

Mammalian cells typically undergo a sequential adhesion process involving cytoskeletal organization, substrate attachment, and cell spreading [296]. To visualize these events in HUVECs cultured on unmodified and surface-modified PCL NFs, rhodamine-phalloidin staining was employed to label filamentous actin [338]. Analysis of cytoskeletal organization and cell morphology revealed that HUVECs on pristine PCL NFs remained predominantly spherical or weakly elongated, indicating limited adhesion and restricted spreading (Fig. 22A) [338]. In contrast, PDA-coated PCL NFs supported a well-spread, fibroblast-like morphology characterized by extensive cytoskeletal organization. Gelatin-coated fibers exhibited an intermediate response, with a heterogeneous population containing both spread and rounded cells. This difference is probably due to the distinct interfacial chemistries of the coatings. In the case of PDA, spontaneous oxidative polymerization generates a bioactive surface capable of covalently interacting with serum proteins via Schiff-base formation and Michael-type addition reactions involving amine and thiol groups [288]. These adsorbed proteins retain their native conformation and biological activity, thereby providing stable and functionally active adhesion sites for cells [297]. By contrast, gelatin attachment to PCL is predominantly driven by weak physical adsorption, resulting in reduced stability and lower surface density of bioactive ligands, ultimately limiting sustained cell adhesion and spreading [298]. Immunofluorescence analysis further supported these observations. The endothelial junctional marker PECAM-1 was primarily localized at cell-cell interfaces in PDA- and gelatin-coated samples, indicating enhanced intercellular connectivity. In contrast, in unmodified PCL, it was largely restricted to isolated cells with poor junction formation (Fig. 22B) [338]. Consistent trends were observed on non-adherent control substrates. In the absence of surface modification, HUVECs displayed poor attachment and a diffuse cytoskeletal structure lacking well-defined stress fibers (Fig. 23A) [338]. Although gelatin coating modestly improved cell spreading, substantial heterogeneity in morphology remained across different substrates. In contrast, PDA modification promoted uniform adhesion and robust stress fiber formation across all underlying materials, indicating strong, substrate-independent bioactivity. Metabolic activity assays further corroborated these morphological findings. MTT analysis demonstrated that PDA treatment significantly enhanced HUVEC viability, increasing metabolic activity by approximately 7.5-fold on PDMS, 8.8-fold on SR, and 2.7-fold on PE compared to unmodified controls, whereas gelatin produced only marginal improvements (Fig. 23B) [338]. Overall, these results demonstrate that PDA serves as a versatile, broadly effective surface modification strategy applicable to both flat films and 3D nanofibrous scaffolds, significantly enhancing endothelial adhesion and functionality in VTE applications [338].

**Fig. 22:**
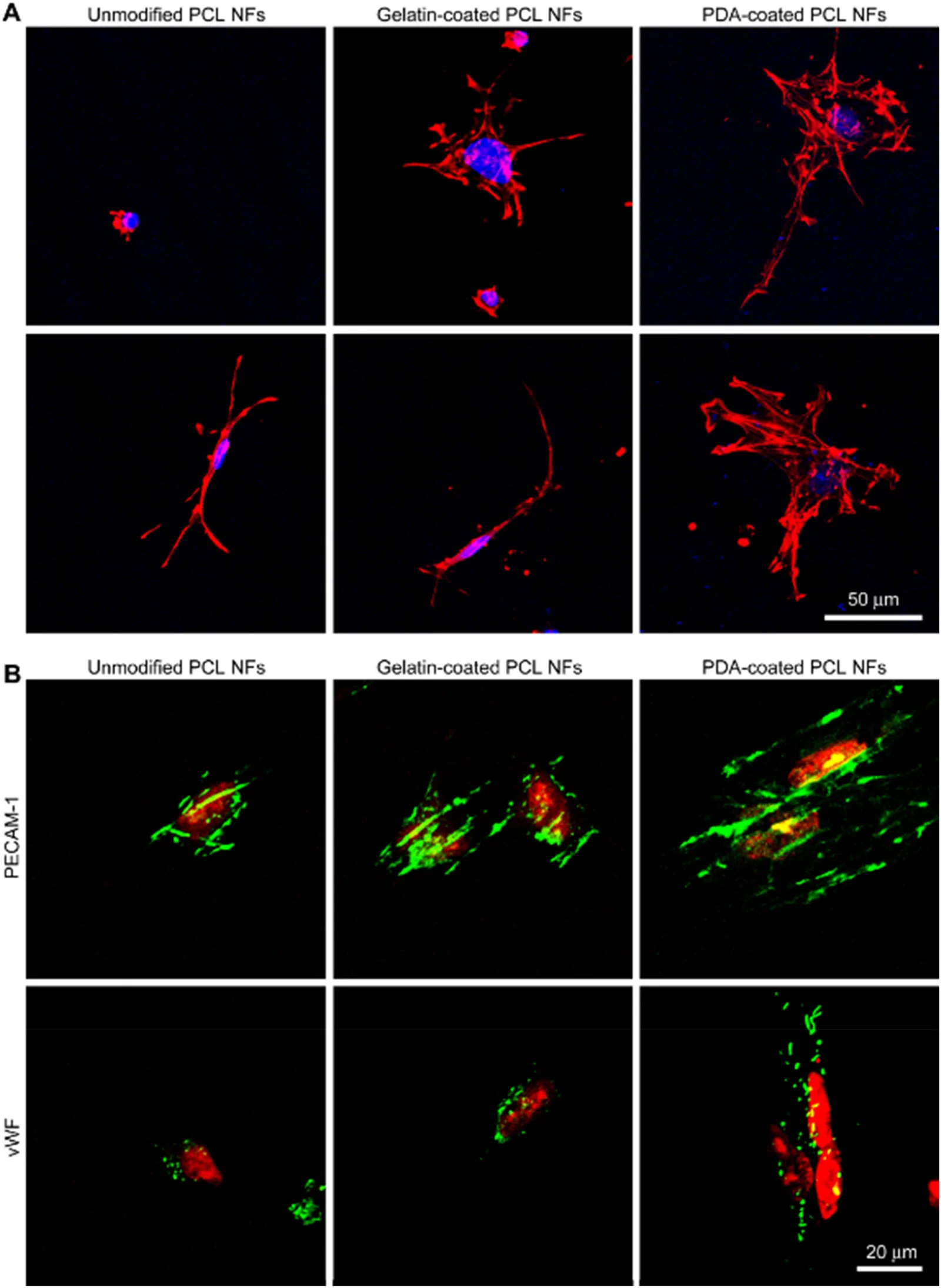
Cytoskeleton formation and endothelial marker expression (PECAM-1, vWF) [338]. Copyright 2010. Reproduced with permission from Elsevier.

**Fig. 23:**
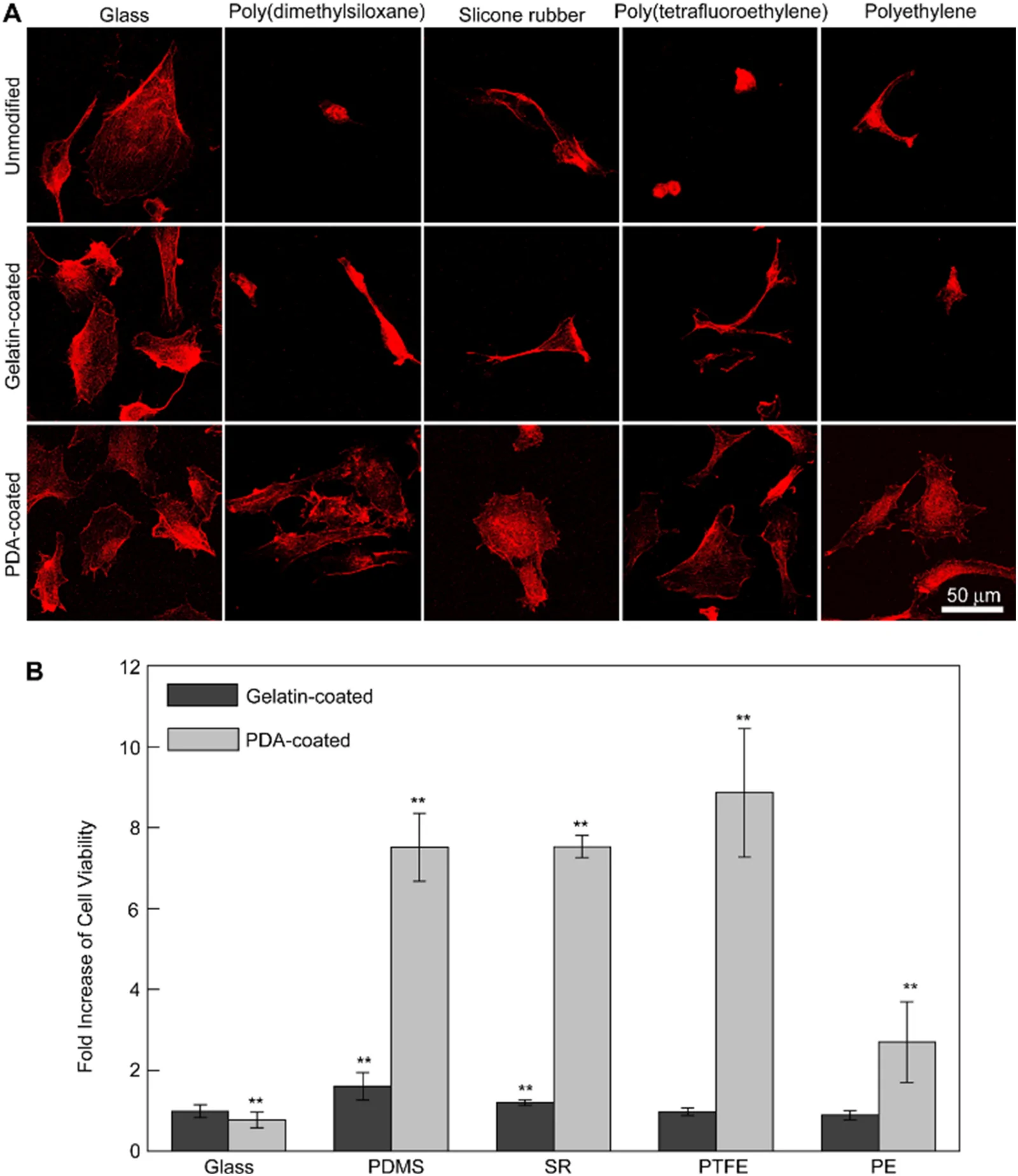
Cytoskeletal formation and cell-viability fold-increase across substrates [338]. Copyright 2010. Reproduced with permission from Elsevier.

A wide range of cell types benefit from nanofibrous meshes, including keratinocytes, hepatocytes, MSCs, and embryonic stem cells, largely due to their high interface-to-volume proportion and ECM-mimetic topography [341]. In liver TE, for example, acrylic acid has been photo-grafted onto thermoplastic NFs to introduce -COOH functionalities, which are subsequently conjugated to galactopyranoside. The resulting galactosylated, highly porous poly (ε-caprolactone-co-ethyl ethylene phosphate) (PCLEEP) scaffolds supported rat hepatocyte adhesion in vitro and promoted spheroid formation with enhanced hepatic function [339]. Fiber architecture has also been shown to strongly influence cellular behavior. Electrospun meshes fabricated in both random and aligned configurations and subsequently coated with collagen supported human coronary artery endothelial cell proliferation along the direction of the underlying fibers, while maintaining characteristic endothelial morphology independent of alignment [341]. In cartilage bioreactor systems, nanofibrous grids have enabled long-term stem cell expansion and chondrogenic differentiation over 42 days, resulting in elevated expression of key cartilage-specific ECM components like aggrecan and type II collagen [341]. More generally, surface immobilization of adhesive ligands, including collagen, fibronectin, and RGD peptides, leverages the high-aspect-ratio nanofibrous architecture to increase ligand density relative to conventional flat substrates, thereby enhancing initial cell adhesion and accelerating proliferation [341].

Electrospun meshes have also been extensively explored for epithelial TE. Through glutamate-mediated crosslinking, fibronectin was successfully grafted onto PLLC NFs without altering fiber morphology. Although a slight reduction in tensile properties was observed due to mild polyester aminolysis, SEM imaging, immunohistological analysis, and protein quantification confirmed significantly enhanced proliferation and adhesion of porcine esophageal epithelial cells. Besides the structural support, ESNFs have served as efficient platforms for drug and gene delivery, benefiting from rapid fluid uptake at mucosal surfaces driven by nanoscale porosity and strong mucoadhesive properties. Collagen functionalization further enhances stem cell attachment and proliferation, with dose-dependent increases in neural stem cell growth and differentiation attributed to improved initial adhesion and sustained viability on the nanofibrous interface [225]. In parallel, hybrid electrospun systems composed of biodegradable polymers, PLGA beads, and PLLA have been used to expand mouse MSCs, although translating high in vitro cell yields into stable in vivo engraftment remains a key challenge [342].

Chemically modified CNFs provide an additional class of bioactive scaffolds. For example; 2,2,6,6-tetramethylpiperidine 1-oxyl (TEMPO)-oxidized CNFs (TOCNFs), which contain C6-COOH groups, support adhesion and proliferation of mouse fibroblasts and myoblasts even in the absence of protein coatings, highlighting the role of charge-mediated cell-material interactions [343]. Further surface phosphorylation of CNFs has been demonstrated to directly induce osteogenic differentiation and matrix mineralization in osteoblast-like cells without the need for exogenous growth factors or collagen supplementation [343]. These findings emphasize that nanoscale crystallinity and surface chemistry are key determinants of cellular response at the biomaterial interface. In the context of xeno-free and serum-free culture systems, recent studies have explored ECM-mimetic alternatives such as polyuronates and sulfated glycosaminoglycan (sGAG)-like materials to replace animal-derived collagen [343], [344]. Combining TOCNFs with polyuronate motifs and newly developed sulfated CNFs (S-CNFs), which mimic GAG sulfate groups, has enabled adhesion and proliferation of primary human MSCs under serum-free conditions. A graphic overview of this strategy is shown in Fig. 24 [344]. Importantly, stem cell multipotency was preserved across multiple CNF-based substrates (TOCNF, TS11, and S-CNF0.64). Immortalized hMSCs expanded for 3 passages retained their differentiation potential, as confirmed by effective induction into osteogenic, adipogenic, and chondrogenic lineages. As shown in Fig. 25 [344], chondrogenic induction produced spheroidal cell aggregates within 24 h that stained positively with Alcian Blue after cryosectioning, whereas non-induced controls remained flattened with minimal staining. Adipogenic differentiation resulted in intracellular lipid droplet formation observed by Oil Red O staining, in contrast to undifferentiated spindle-shaped cells. Overall, these results demonstrate that surface-engineered CNFs can sustain stem cell growth and preserve multipotency under serum-free conditions, highlighting their strong potential for clinically relevant, xeno-free regenerative medicine applications [344].

**Fig. 24:**
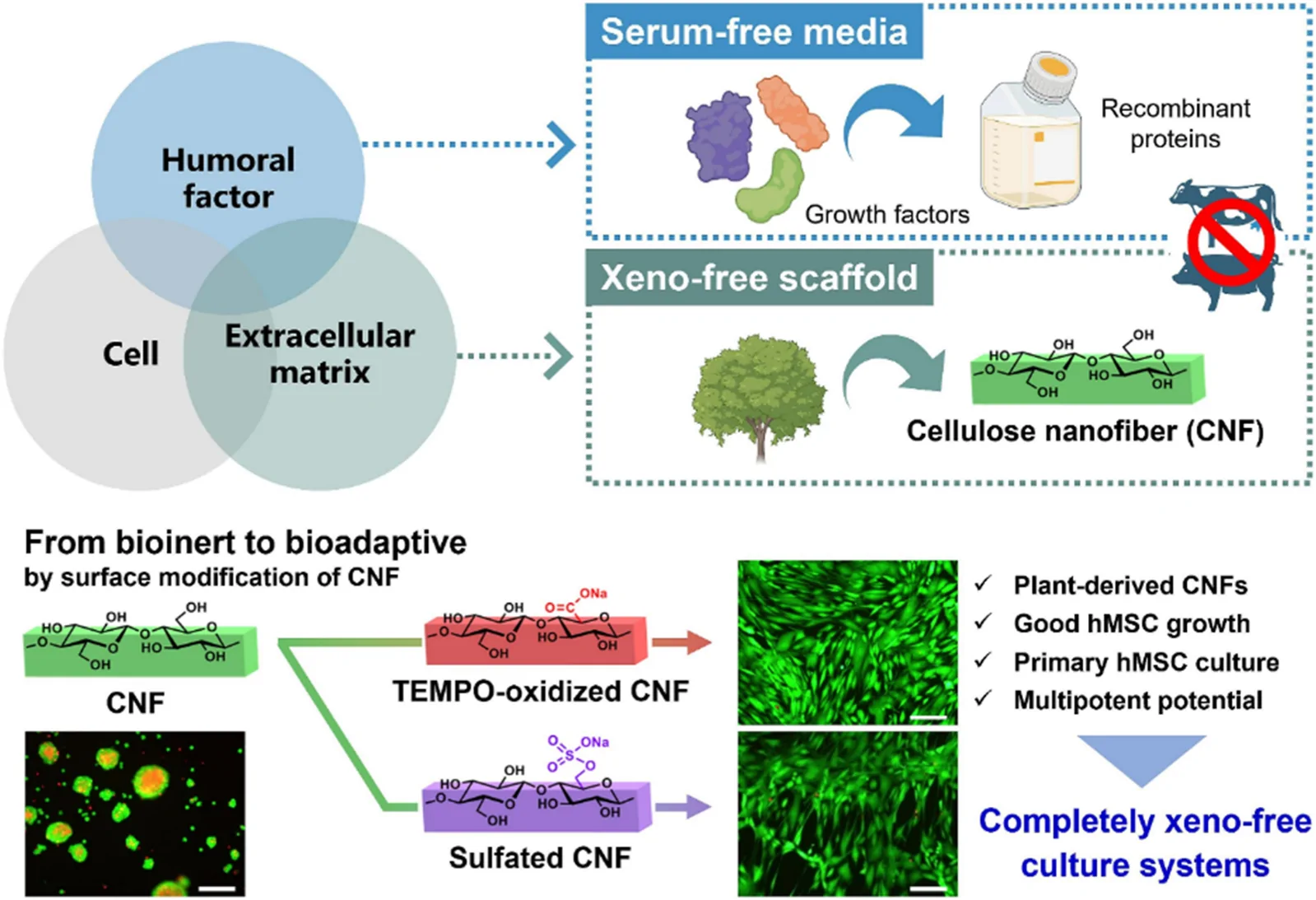
Schematic of TOCNF/S-CNF scaffolds enabling serum-free hMSC culture [344]. Copyright 2024. Reproduced with permission from Elsevier.

**Fig. 25:**
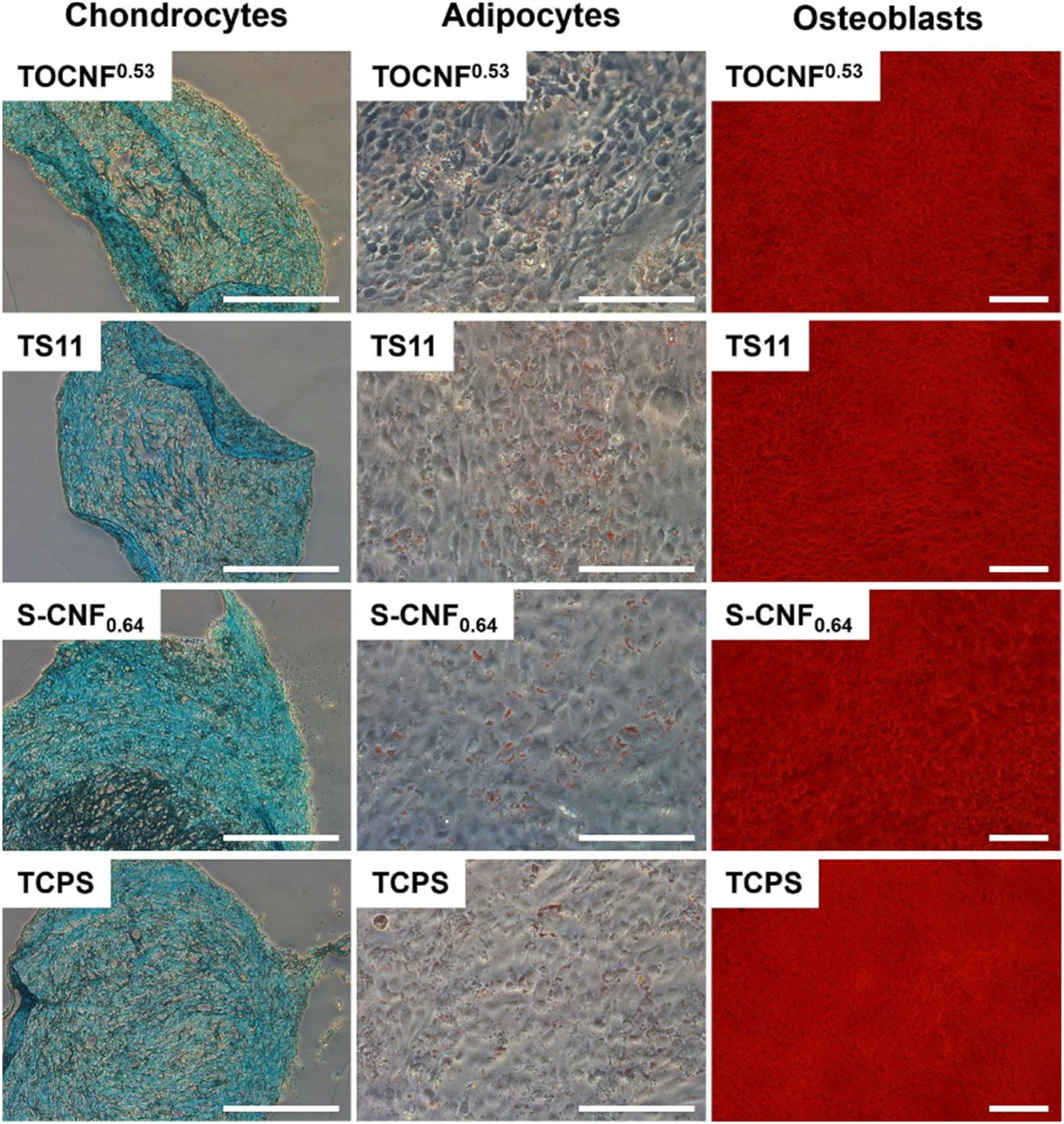
Optical micrographs of hMSC differentiation into chondrocytes, adipocytes, and osteoblasts on CNF substrates [344]. Copyright 2024. Reproduced with permission from Elsevier.

For soft-tissue repair, nanofiber-hydrogel composite systems have demonstrated particular promise in skin regeneration, where mechanical demands are lower than in load-bearing tissues. Liu et al. [345] established an alginate-based hydrogel reinforced with PCL NFs as an implantable skin substitute. The system was further functionalized with an amino-terminated hyperbranched polyamide to impart antimicrobial activity. The resulting composite exhibited high tensile strength, favorable water uptake, and appropriate water vapor permeability, leading to effective performance in an in vivo skin defect model. Similarly, protein-based photocrosslinked hydrogels incorporating NF dispersions have been reported as efficient wound dressings [346]. In this approach, gelatin and decellularized dermal matrix were chemically modified to form gelatin methacryloyl (GelMA) and methacrylated decellularized dermal extracellular matrix (ddECMMA), respectively. PCL NF suspensions and thioglycolic acid-modified chitosan (TCS) were incorporated into the precursor solutions, followed by photocrosslinking to generate four hydrogel systems: GelMA, GTP4, DP, and DTP4. These hydrogels exhibited favorable physicochemical properties, excellent biocompatibility, and negligible cytotoxicity. In a full-thickness skin defect model in Sprague-Dawley rats, all hydrogel-treated groups showed significantly enhanced wound healing compared to untreated controls. Histological analyses using hematoxylin and eosin (H&E) and Masson's trichrome staining further confirmed improved tissue regeneration, particularly in hydrogels containing both PCL NFs and TCS (GTP4 and DTP4). Among these, the GTP4 formulation demonstrated the most pronounced regenerative effect, highlighting its strong potential for advanced STE applications [346]. Overall, nanofiber reinforcement provides a practical strategy for hydrogel-based wound dressings by improving mechanical stability without requiring excessive crosslinking density. In addition, the embedded nanofibrous network introduces interfacial porosity, enhancing mass transport and providing structural cues that support cell adhesion, migration, and alignment [346]. Beyond skin applications, nanofiber-hydrogel composites incorporating HAp or HAp-like structures have shown strong potential in bone regeneration due to the osteoconductive nature of CaP phases [308], [347]. For instance, Wang et al. [348] fabricated high-aspect-ratio HANFs and integrated them into GelMA hydrogels to form m-HANFs/GelMA composites. Subsequent biomineralization in SBF induced the formation of spherulitic HA structures resembling native bone mineral, which significantly enhanced the proliferation and osteogenic maturation of MC3T3-E1 cells [348].

### 4.2 Drug delivery

The literature increasingly highlights drug-eluting nanofiber layers integrated into implants and stents, enabling medical devices to function as localized DD platforms. In a representative study, a urethral stent was wrapped with an ESNF membrane designed for sustained nitrofurantoin delivery, a commonly used antibiotic for urinary tract infections [349]. Drug-free NFs were first fabricated, followed by deposition of drug powder onto the membrane surface, after which the assembly was stretched over the stent to form a multilayer stent-drug-nanofiber construct. In vitro release studies conducted in PBS buffer (0.1 M PBS, pH 6.8; 50 mL) demonstrated near-zero-order release kinetics over 28 days, with cumulative drug release of about 82.8 ± 1.75%. Importantly, in vivo results confirmed the system's ability to deliver prolonged, localized antimicrobials and to improve infection control [349]. Surface-modified, prefabricated nanofibrous meshes are particularly advantageous for drug immobilization on high-surface-area interfaces. Given their exceptionally high interface-to-mass ratio, these meshes can achieve higher drug loading than conventional carriers. They can be engineered for rapid, localized “on-demand” release, making them especially suitable for post-operative antimicrobial prophylaxis [257]. More advanced hierarchical strategies further expand their functionality. For example, drug-loaded NPs can be adsorbed onto nanofiber surfaces to generate composite delivery systems capable of multi-phase or sequential release profiles that are difficult to achieve using homogeneous fiber matrices alone [350], [351]. Three commonly employed physical loading strategies for ESNF surfaces are summarized in Fig. 26 [128]. For sensitive molecules such as proteins and nucleic acids, surface immobilization offers a key advantage over bulk encapsulation, as it avoids exposure to harsh ES conditions and the solubility limitations of high-molecular-weight biomolecules in organic solvents. Consequently, biologically active macromolecules can be selectively or non-selectively immobilized on engineered nanofiber surfaces, enabling localized and sustained release while preserving their functional integrity [352].

**Fig. 26:**
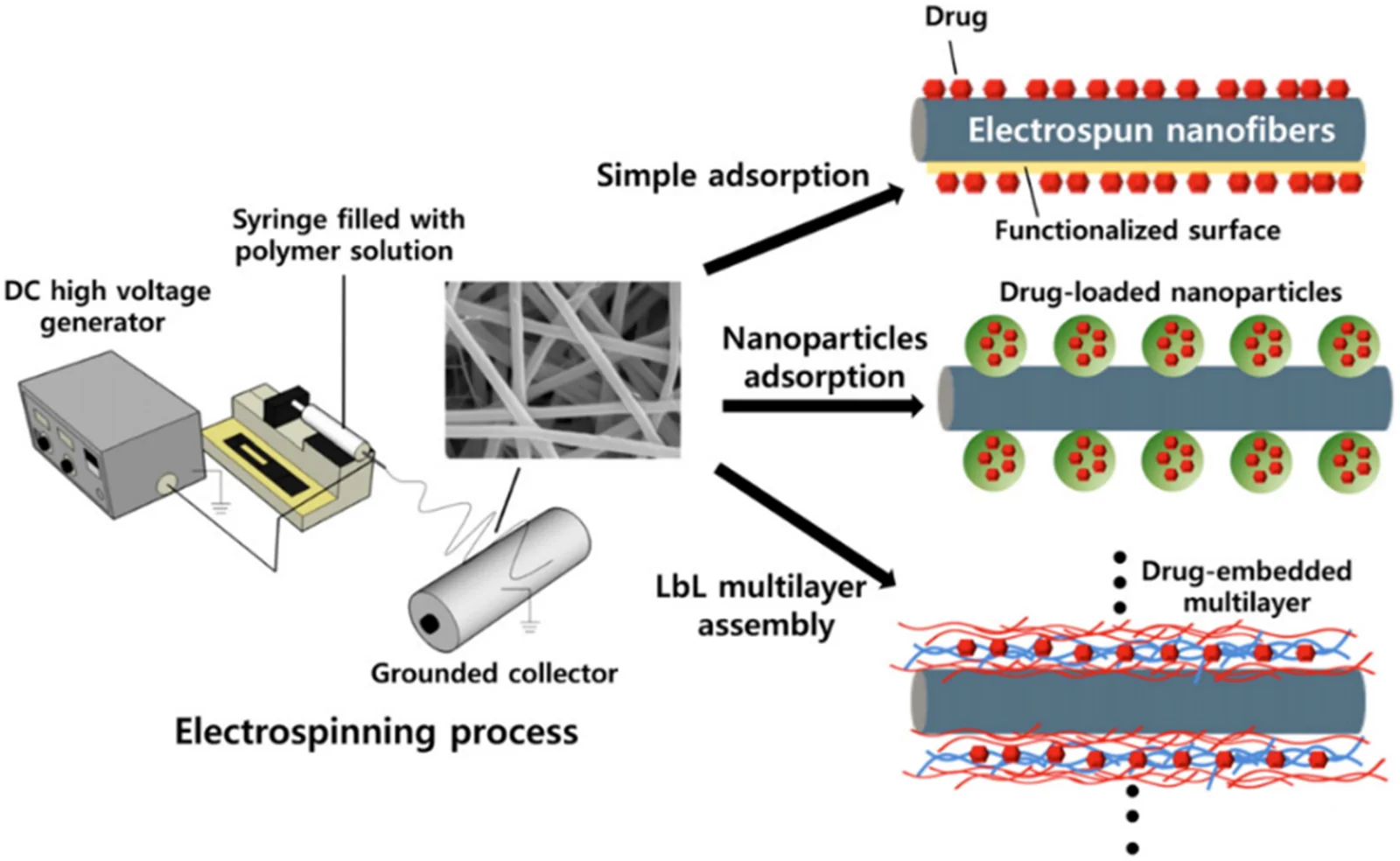
Three physical routes to load drugs on ESNF surfaces [128]. Copyright 2009. Reproduced with permission from Elsevier.

Nanofiber-coated implants have demonstrated strong potential for localized and sustained DD in ophthalmic applications. For instance, Guerra et al. [353] developed an intravitreal ESNF system co-delivering bevacizumab (50 μg) and dexamethasone (300 μg) from a biodegradable implant. In this system, PLGA degradation primarily governed dexamethasone release, which initiated around day 7 and peaked between days 10-15, whereas bevacizumab exhibited a sustained release profile reaching approximately 88% cumulative release by day 48 [353]. The dual-DD platform was well tolerated when administered intravitreally, highlighting its potential for long-term ocular therapy. More broadly, nanofiber processing parameters can be systematically tuned to regulate drug loading and release kinetics. Kersani et al. [352], for example, proposed a stent-based strategy for preventing post-angioplasty restenosis using simvastatin-loaded electrospun coatings. Dynamic flow studies showed that coating time (10, 30, and 60 min) had minimal influence on burst release for CS-only NFs; however, when CS was combined with cyclodextrin, coating duration significantly modulated release behavior. The time required to reach 90% drug release increased from approximately 15 min to 120 min and 300 min with increasing coating times, respectively, demonstrating the strong influence of processing conditions on drug release kinetics [352]. While short-term in vivo studies confirm the therapeutic efficacy of nanofiber systems for up to 14 days [353], potential systemic drug exposure at higher doses remains a concern [354], underscoring the importance of careful dose optimization. In addition to process-controlled release, pH-responsive nanofiber systems provide an additional mechanism for spatiotemporal control. PLA/CS@ZIF-8 (PLA/CS@ZIF-8) nanofibrous film layers have been shown to exhibit increasing drug loading capacity (DLA) and encapsulation efficiency (EE) with higher 2-methylimidazole (2-MIM) concentrations, attributed to enhanced ZIF-8 crystal growth on the nanofiber surface [355]. For camptothecin (CPT) and artemisia polysaccharide (APS), DLA/EE values reached approximately 32%/91% and 36%/93%, respectively. In vitro release experiments showed distinct release profiles for the two drugs. APS, being hydrophilic, exhibited rapid diffusion, with more than 50% released within 8 h, whereas CPT showed a slower release of approximately 30% over the same period (Fig. 27a and b) [355]. This behavior is consistent with the higher aqueous solubility and diffusion rate of hydrophilic compounds compared to hydrophobic molecules [356], [357], [358]. Release profiles gradually plateaued at approximately 90% for APS and 50% for CPT as diffusion became rate-limiting. Environmental pH further modulated release behavior (Fig. 27c and d) [355]. At physiological pH (7.4), both drugs exhibited sustained release, whereas under acidic conditions (pH 5.5), cumulative release increased to approximately 75% within 10 h. This acceleration is attributed to acid-induced degradation of the ZIF-8 framework into zinc ions and organic ligands [359]. Despite this enhancement, CPT continued to exhibit slower release than APS, reaching only ∼50% under acidic conditions due to diffusion limitations associated with its hydrophobic nature. Overall, these findings demonstrate that PLA/CS@ZIF-8 NFMs function as versatile pH-responsive DD systems, making them particularly suitable for targeted delivery in acidic tumor microenvironments [355].

**Fig. 27:**
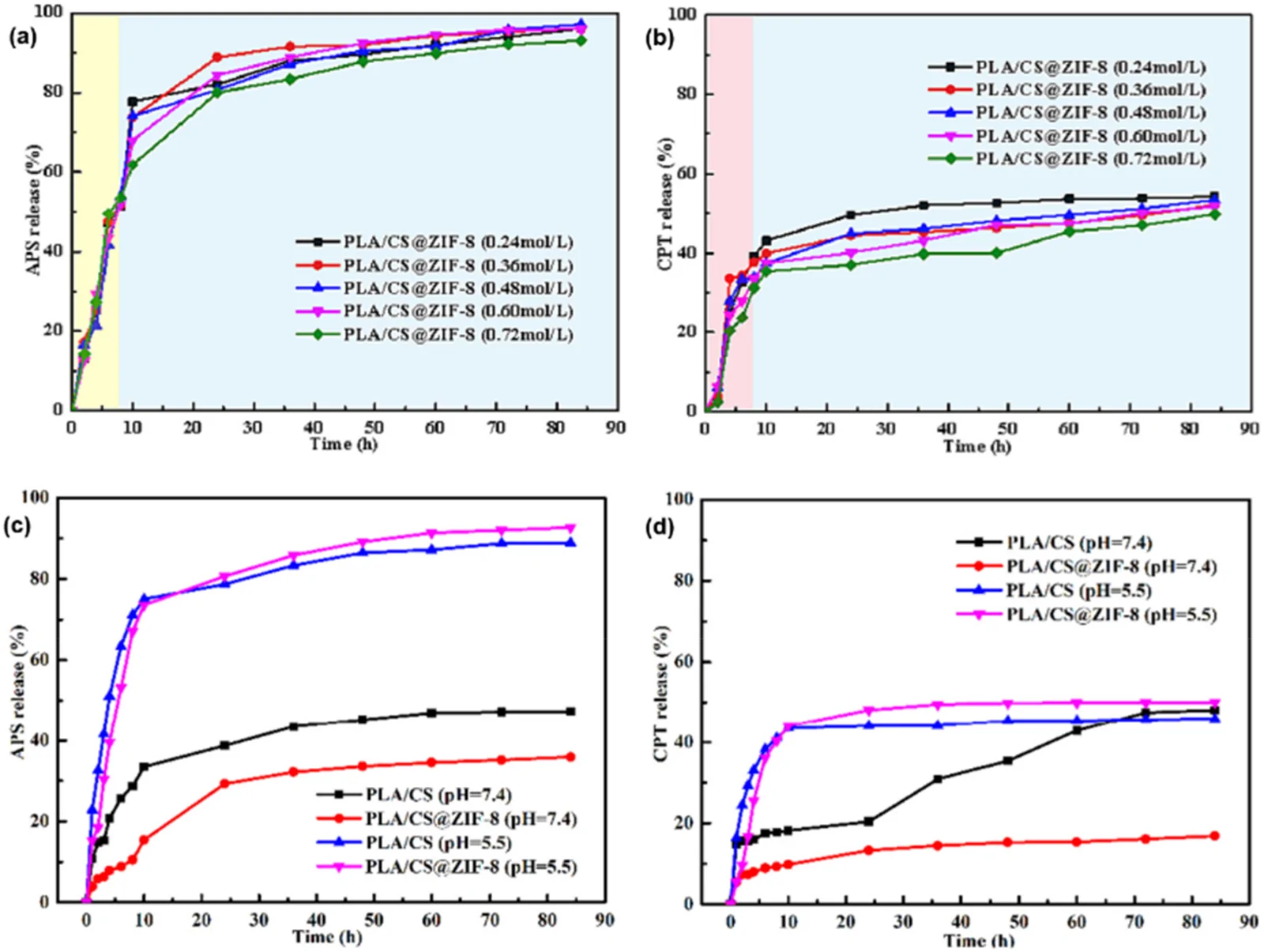
Cumulative drug release profiles from PLA/CS@ZIF-8 NF membranes prepared under varying precursor concentrations, shown for (a) APS and (b) CPT. Panels (c) and (d) illustrate the pH-dependent release behavior of APS and CPT, respectively, comparing PLA/CS membranes with PLA/CS@ZIF-8 NF systems [355]. Copyright 2024. Reproduced with permission from Elsevier.

For applications requiring rapid and localized drug administration, fast-dissolving electrospun systems offer significant advantages. Eudragit-based vancomycin-loaded NFs (E-VAN) have been shown to provide sustained antibiotic release for at least 7 days, with an initial faster release phase attributed to increased hydrophilicity and improved wetting behavior [360]. Such characteristics make these systems suitable for loco-regional therapies, including intravaginal DD [361], intraoral anti-inflammatory treatment [362], anticancer applications [356], and rapid infection control in wound environments [363]. Sustained and tunable drug release from Eudragit matrices can be further achieved through polymer blending or crosslinking strategies [364]. For example, blending E-VAN with the cationic polymer EE100 (25:75, E-VAN1) resulted in pH-responsive release behavior, with the quickest release observed at pH 5.5. In contrast, blending with the ionizable polymer ES100 (25:75, E-VAN2) produced an inverted pH response, showing minimal release at pH 5.2 and accelerated release at pH 8.8, reflecting the solubility profile of ES100 above neutral pH [360]. These tunable behaviors have important practical implications. Specifically, E-VAN2 formulations can function as “smart” wound dressings that enhance drug release in alkaline conditions typically associated with infected wounds, while maintaining reduced release under mildly acidic conditions characteristic of healthy skin [360]. A ternary blend system (E-VAN/ES100/ERL100; 25:40:35) showed intermediate behavior, with relatively faster release at acidic pH (pH5.2) and more sustained release at physiological pH (pH7.4) compared to E-VAN2. Formulations with higher ES100 content (E-VAN4 and E-VAN5) enabled more refined control of release near physiological pH. Overall, these differences are primarily governed by electrostatic interactions between vancomycin and the polymer matrices (EE100-ES100 and VAN-ES100 interactions). Importantly, vancomycin stability remained high throughout the release period, with approximately 96.9%, 97.1%, and 81.5% of the drug remaining after 7 days at pH 5.2, 7.4, and 8.8, respectively [360].

Beyond polymer blending systems, hierarchical “nano-in-nano” architectures further expand control over DD. As illustrated in Fig. 28 [365], capsaicin-loaded alginate NPs (CAP ALG NPs) were embedded within PCL-CS ESNFs to create a composite delivery platform designed to stabilize the drug, regulate release kinetics, and enhance anticancer efficacy. Within alginate NPs, CAP achieved an exceptionally high encapsulation reaching 98.7%, primarily driven by hydrogen bonding between CAP functional groups and the -OH and charged moieties of alginate, Tween 80, and the cationic gemini surfactant, consistent with previously reported high encapsulation efficiencies in alginate-based systems [365]. In vitro release assessment demonstrated a biphasic release pattern. An initial burst phase within approximately 30 min released ∼20% of CAP from free NPs and ∼35% from nanoparticle-loaded NFs, attributed to surface-associated drug on high-area interfaces. Subsequently, free CAP ALG NPs released nearly 100% of their payload within 120 h (5 days), whereas nanoparticle-embedded NFs extended release to over 500 h (approximately 21 days). These results clearly demonstrate that integrating NPs within electrospun fiber matrices provides a powerful strategy for substantially prolonging and modulating drug release profiles [365].

**Fig. 28:**
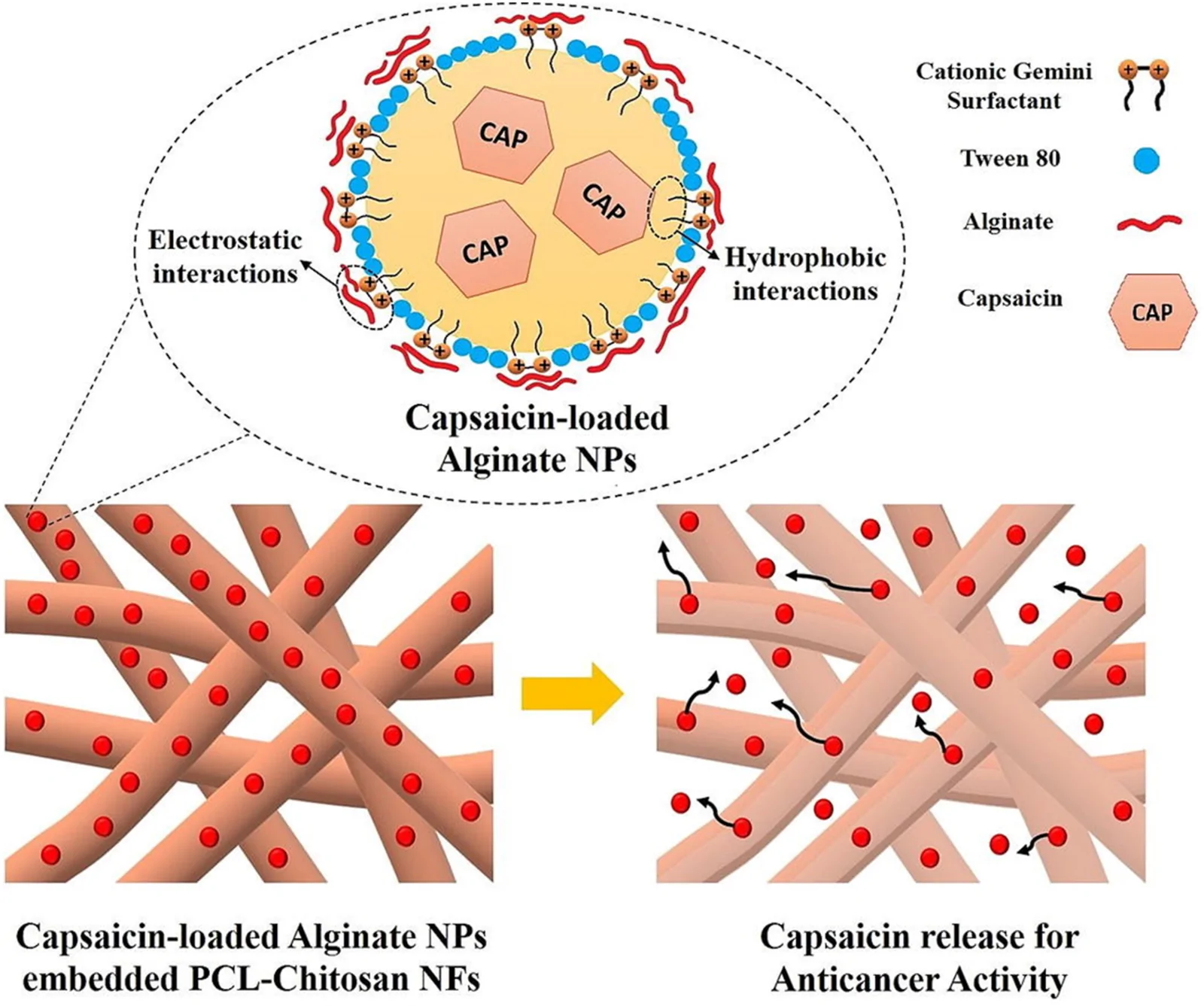
Schematic representation of the nano-in-nano DD platform, illustrating CAP-ALG NPs embedded within PCL-CS NFs [365]. Copyright 2023. Reproduced with permission from Elsevier.

Hydrogels have been widely explored as DD matrices due to their high water content, biocompatibility, and adjustable physicochemical properties. However, drug release from conventional hydrogels is primarily diffusion-controlled, making precise regulation of release kinetics difficult and limiting their suitability for long-term therapeutic delivery. To overcome these limitations, incorporating NFs into hydrogel systems has emerged as a promising approach to modulate drug transport and enhance release performance [366]. NFs can modify the mechanical properties of the hydrogel network, alter diffusional pathways, and simultaneously function as independent drug reservoirs. This dual functionality enables orthogonal engineering of both hydrogel and fiber components, thereby facilitating synergistic control over mechanical behavior and therapeutic release.

Several studies have demonstrated the versatility of nanofiber-hydrogel composite systems for coordinated DD. For example, Nakielski et al. proposed a thermoresponsive poly(NIPAM) hydrogel containing gold nanorods (AuNRs) and embedded drug-loaded PLA NFs [367]. Upon near-infrared irradiation, the plasmonic resonance of the AuNRs generated localized heating, increasing the temperature to approximately 42 °C and inducing hydrogel deswelling. The resulting water expulsion, combined with mild thermal stimulation, accelerated drug release from the NFs. In another study, Liguori et al. prepared a gelatin hydrogel reinforced with PEO/PBT NFs, in which diclofenac was encapsulated within the fibers and chlorotetracycline within the hydrogel matrix [368]. Drug release behavior was strongly influenced by laminate architecture, with double-layer constructs containing an additional hydrogel layer exhibiting more gradual release profiles than monolayer systems. Similarly, Zare et al. designed multilayer laminates composed of alginate hydrogel sheets, PLA nanofiber mats, and PLGA NPs loaded with kartogenin [369]. In this system, the increased diffusion pathway length and nanoparticle encapsulation synergistically contributed to prolonged and sustained drug release.

NF-based systems have also been studied for wound-healing applications, where DD is combined with structural and biological support. β-cyclodextrin-grafted CS/PVA) (β-CD-g-CS/PVA) NFs loaded with indomethacin represent one such example [370]. CS contributes intrinsic hemostatic activity comparable to collagen-, fibrinogen-, and thrombin-based materials while also promoting cellular attachment. In these systems, indomethacin was dispersed separately within CS and β-CD-g-CS matrices before ES. Both preparations showed a distinct biphasic release profile involving a preliminary fast phase followed by a prolonged near-linear release phase, a behavior generally observed in blend-electrospun systems [370], [371]. The initial fast phase, typically observed within the first day at physiological pH and temperature, was attributed to molecules localized near the fiber surface during fabrication and storage. The rapid diffusion of these molecules into the release medium was driven by thermodynamic partitioning and their high aqueous solubility [370]. The subsequent sustained-release phase was primarily governed by matrix swelling and degradation. Native CS fibers degraded slowly, resulting in an approximately linear release profile. In contrast, β-CD-g-CS systems demonstrated swelling-mediated release that accelerated as degradation progressed [370]. The incorporation of β-cyclodextrin increased hydrophilicity, swelling capacity, and drug-loading efficiency, resulting in faster and more complete drug release. Indomethacin release from β-CD-g-CS fibers reached equilibrium after approximately 125 h and continued gradually until nearly 300 h, indicating sustained diffusion from deeper regions of the hydrated matrix. The close correspondence between swelling and release profiles suggested that swelling dominated transport at early stages, whereas diffusion-controlled release became more significant over time. Notably, CS fibers released only approximately 20% of the encapsulated drug over 12 days, whereas β-CD-containing systems achieved nearly complete release, facilitated by cyclodextrin cavities that facilitate drug transport [370]. Compared with CS/PVA fibers, β-CD-g-CS/PVA fibers also exhibited faster initial release because of enhanced drug dispersion, increased hydrophilicity, and improved solubility. In contrast, bead formation within CS/PVA fibers reduced the fraction of surface-accessible drug [370]. Overall, these systems demonstrated prolonged local DD behavior, which may be further improved through the integration of biomolecules like nerve growth factors.

Surface modification strategies have additionally been employed to optimize nanofiber-mediated drug release. In a recent study, CAP treatment was applied to quercetin (QU)-loaded PCL NFs to enhance their DD performance [372]. CAP exposure for 0.5, 1, and 3 min induced morphological alterations in NFs, resulting in changes in fiber diameter and surface characteristics. These modifications significantly influenced release kinetics and loading efficacy. The cumulative release of QU increased from 29 ± 1.8% in untreated PCL/QU fibers to 56 ± 2.2% following 0.5 min CAP treatment, while longer treatments of 1 and 3 min reduced release to 48 ± 4.2% and 44 ± 3.9%, respectively, as shown in Fig. 29 [372]. Similarly, loading efficiencies improved following short-duration CAP exposure, reaching a maximum of 88 ± 4.4% after 0.5 min treatment. These findings indicate that moderate CAP treatment enhances drug diffusion and loading capacity, whereas prolonged exposure may induce surface densification or structural rearrangement that impedes molecular transport [372], [373]. Collectively, these studies emphasize the significant potential of nanofiber-based composite and surface-engineered systems for achieving tunable, sustained, and multifunctional DD.

**Fig. 29:**
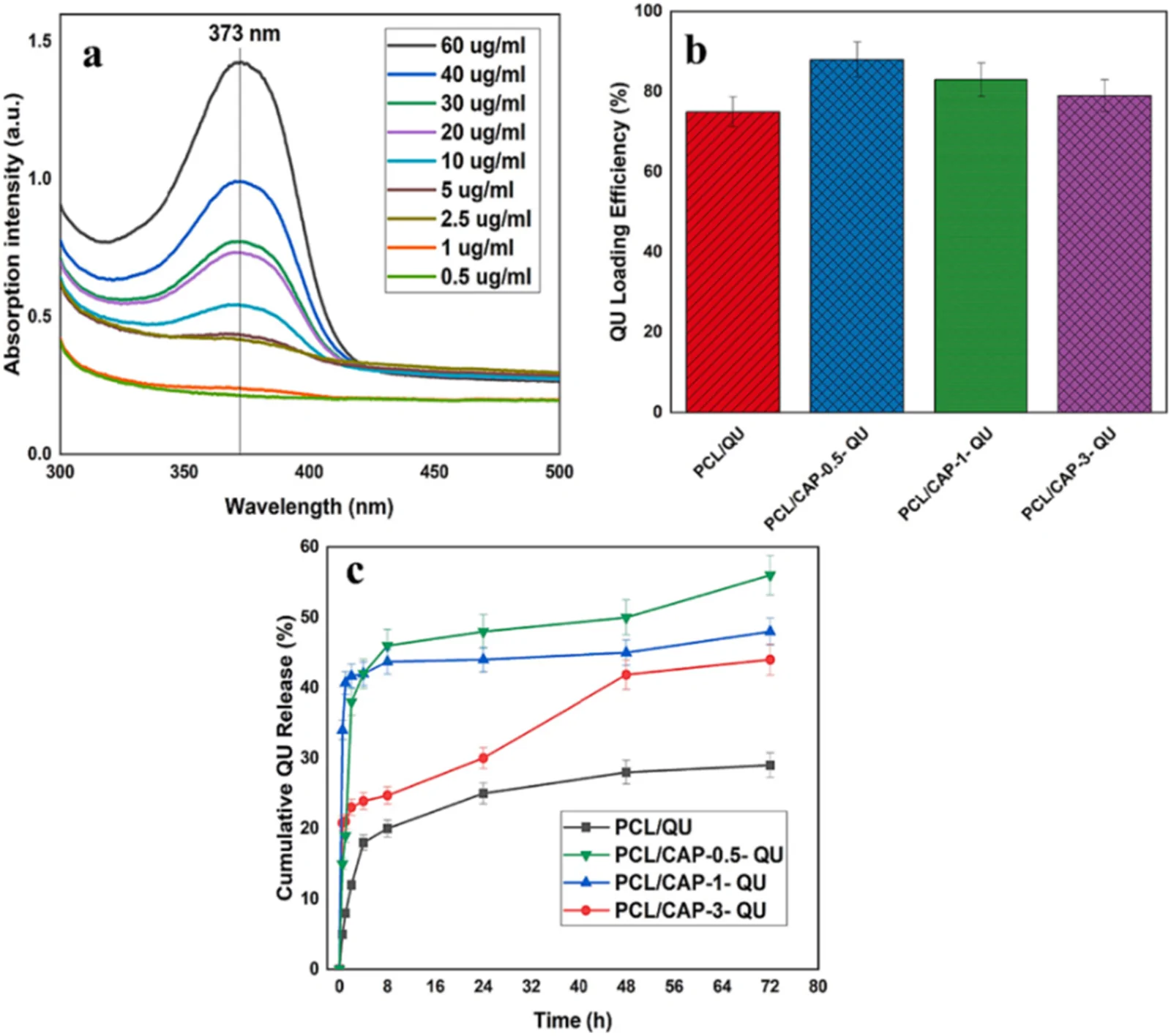
Drug release profile of ESNFs [372]. Copyright 2024. Reproduced with permission from Elsevier.

Collectively, these studies highlight the magnitude of controlling the release kinetics of NF-based DD systems to achieve effective therapeutic outcomes. Drug release must provide appropriate concentrations over clinically relevant durations [374]. Recent advances summarized in Table 2 continue to leverage the many advantages of ESNFs, including their high surface area-to-volume ratio, interconnected porosity, ECM-like structure, flexible processing, and ability to incorporate diverse therapeutics such as peptides, proteins, antibodies, and small molecules [373], [375], [376], [377], [378], [379], [380]. ES remains one of the most versatile NF fabrication techniques for DD systems. Increasing commercial availability and the development of advanced ES variants have helped overcome limitations of conventional systems, as summarized in Table 3 [381], [382], [383], [384], [385].

**Table 2:** Representative examples of release kinetics in ESNF-based DD systems.

Release mechanism class	Drug	Polymer/fiber architecture	Fabrication features influencing release	Dominant release behavior	Key mechanistic insight	Ref.
Anomalous (non-Fickian) diffusion	Caffeine	Poly(N-vinylcaprolactam-co-acrylic acid) NFs	Swelling-responsive copolymer network	Korsmeyer-Peppas (non-Fickian)	Polymer swelling contributes equally to diffusion and relaxation processes	[373]
Fickian diffusion (Higuchi/first-order dominated)	Tetrahydro-curcumin	PCL/PEG composite fibers	Blending with hydrophilic PEG increases water uptake	Higuchi + first-order diffusion	Hydrophilic phase accelerates diffusion-controlled release (R^2^ = 0.975)	[375]
Fickian diffusion (early-time burst + sustained diffusion)	Melatonin	PCL monoaxial fibers	Single-layer architecture; surface drug fraction present	First-order diffusion	Initial burst due to surface drug, followed by diffusion-limited transport	[376]
Zero-order-like sustained release (multi-mechanism balance)	Ferulic acid	Cellulose acetate (CA) triaxial fibers	Multi-layer architecture (triaxial structure)	Combined zero-order + Korsmeyer-Peppas	Controlled architecture balances diffusion and erosion to approximate zero-order kinetics	[377]
Diffusion-to-erosion transition (pH-responsive systems)	Diclofenac sodium	Eudragit® L 100-55 monoaxial fibers	pH-dependent polymer solubility and drug loading level	Mixed diffusion-erosion (Fickian → erosion-controlled)	Release mechanism shifts with pH and loading, indicating structure-dependent transport regime	[378]
Anomalous diffusion with swelling-controlled contribution	Azithromycin	PCL/CS dual-jet co-electrospun fibers	core-sheath -like phase separation; hydrophilic CS domain	Korsmeyer-Peppas (non-Fickian)	Multistage release: desorption → swelling-mediated diffusion → degradation	[379]
Diffusion-erosion coupled release	Cisplatin	PEO/PLA composite fibers	Biodegradable matrix with moderate hydrophilicity	Diffusion + matrix erosion	Rapid early release (61.7% in 6 h) due to combined diffusion and polymer degradation	[380]

**Table 3:** Common ES configurations for bioactive compound delivery.

Electrospinning technique	Bioactive compound	Polymer system	Solvent(s)	Outcome/performance	Ref.
**Consecutive ES**	Curcumin	Gelatin/ethyl cellulose	Acetate/water/ethanol	Thermally stable fibers showing sustained 96 h release and preserved antioxidant capacity.	[381]
**Free-surface ES**	Phycocyanin (*Spirulina sp.*)	PEO	Acetate/water	Controlled protein release with retained antioxidant activity.	[382]
**Uniaxial + coaxial ES**	Sour cherry extract (*Prunus cerasus L.*)	Gelatin/lactalbumin	Acetate	Improved bioaccessibility and retention of phenolic compounds.	[383]
**Emulsion ES**	Catechins	PLGA	Water/chloroform	Controlled release with enhanced antioxidative activity and accelerated skin-wound healing.	[384]
**Single-nozzle ES**	Curcumin	CS/gelatin/PCL	Acetic acid/HFIP/PBS	Hydrophilic, wettable, degradable fibers suitable for wound dressings; moderate mechanical strength.	[385]

### 4.3 Wound healing and wound dressing

Skin is the largest organ of the human body and functions as the primary barrier against pathogens, chemical agents, physical trauma, and environmental contaminants [386]. In addition to its defensive role, the skin contributes to vitamin D synthesis, thermoregulation, moisture retention, and sensory perception [387]. Structurally, the skin is composed of three major layers: the epidermis, dermis, and hypodermis. The outermost layer is the epidermis, which prevents transepidermal water loss, maintains internal homeostasis, and protects against external insults [388]. Keratinocytes comprise approximately 80% of epidermal cells, alongside melanocytes, Merkel cells, and tissue-resident immune cells [389]. Under the epidermis lies the dermis, a collagen- and elastin-rich connective tissue layer that provides mechanical strength, elasticity, and nutritional support [390], [391]. The dermis is subdivided into a superficial papillary layer, containing loosely organized collagen fibers, and a deeper reticular layer, characterized by dense collagen bundles aligned parallel to the skin surface. Although less cellular than the epidermis, the dermis contains fibroblasts, fibrocytes, mast cells, dermal dendritic cells, macrophages, innate lymphoid cells, and extensive vascular, lymphatic, and neural networks. The innermost hypodermis primarily consists of adipose tissue arranged in lobules parted by fibrous septa containing blood vessels and nerves. This layer functions as an energy reservoir, thermal insulator, and mechanical cushion [391].

Cutaneous wound healing is a highly coordinated biological process involving numerous cell types, ECM components, and signaling mediators. Regardless of injury etiology, wound healing is generally separated into four temporally overlapping phases: hemostasis, inflammation, proliferation, and remodeling, as illustrated in Fig. 30 [390]. The hemostatic phase begins immediately after injury and is characterized by vasoconstriction and platelet activation at sites of vascular damage. Von Willebrand factor mediates platelet adhesion to exposed subendothelial collagen through interactions with platelet glycoprotein Ib/IX/V receptors, triggering platelet activation and degranulation. Simultaneously, tissue factor-mediated thrombin generation promotes fibrin formation, while activated factor XIII stabilizes the fibrin network through covalent crosslinking. The resulting platelet-fibrin clot not only limits blood loss but also provides a provisional matrix enriched with growth factors and complement proteins that support subsequent cellular recruitment and immune activation [392].

**Fig. 30:**
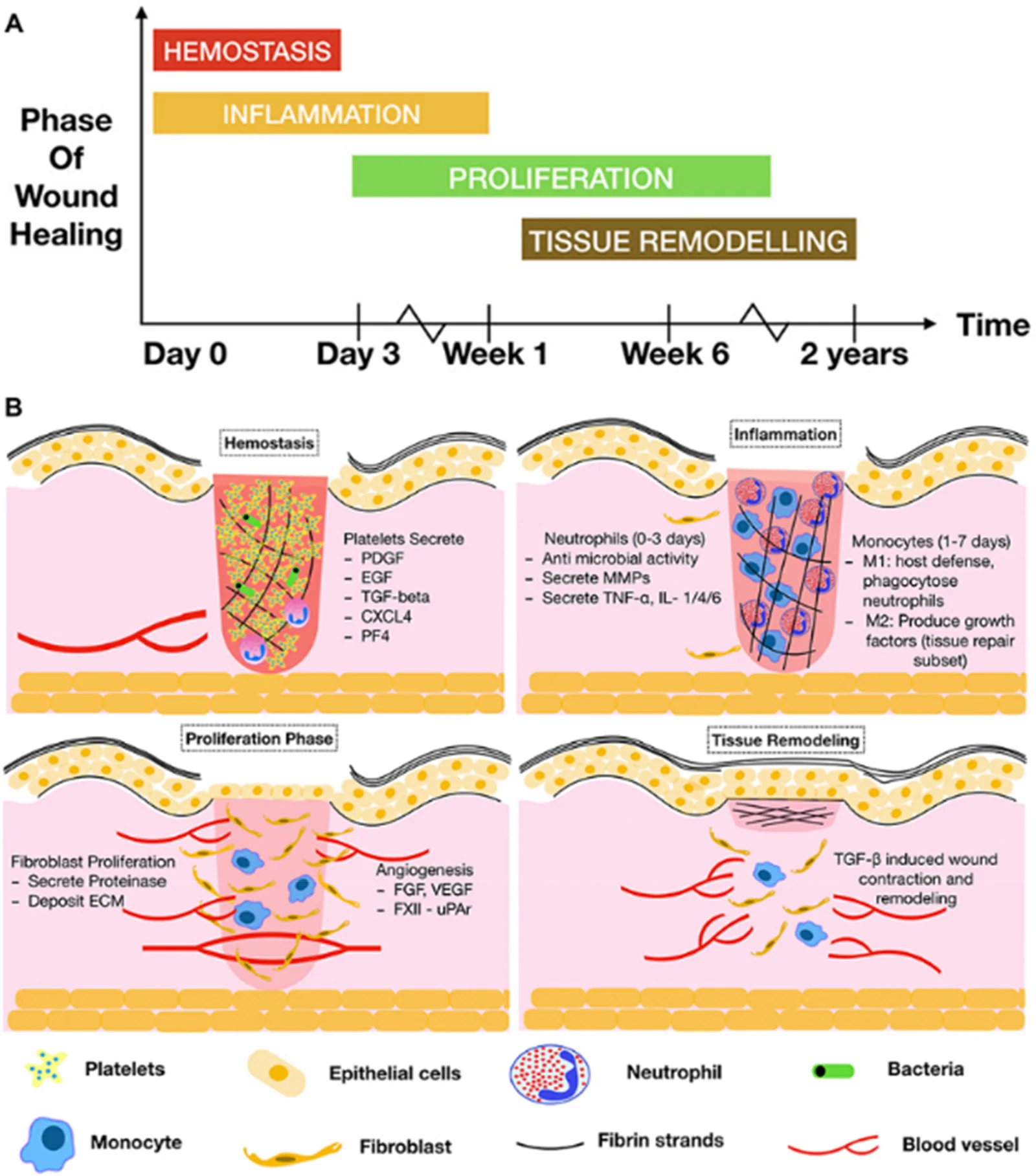
Temporal overlap and cellular contributions across the various stages of wound healing. This includes four major steps: (i) hemostasis, (ii) inflammation, (iii) proliferation, and (iv) remodeling [390]. Copyright 2019. Reproduced with permission from Elsevier.

The inflammatory phase overlaps with hemostasis and functions to eliminate pathogens and remove damaged tissue. Neutrophils are amongst the first recruited immune cells to the damaged site, where they perform phagocytosis, release antimicrobial peptides and proteolytic enzymes, generate reactive oxygen species (ROS), and form neutrophil extracellular traps [393]. As inflammation progresses, macrophages become the dominant cell population. These cells exhibit phenotypic plasticity and are often broadly categorized into pro-inflammatory M1 and anti-inflammatory/pro-regenerative M2 phenotypes [394]. M1 macrophages (CD86^+^) contribute to pathogen clearance in response to stimuli such as LPS and interferon-γ [395], [396], whereas M2 macrophages (CD206+) secrete anti-inflammatory cytokines, including IL-10, along with growth factors that promote angiogenesis, fibroblast activation, and re-epithelialization [397]. Proper macrophage recruitment and phenotypic transition are critical for successful healing; as impaired macrophage function is associated with delayed tissue repair [398].

The proliferative phase is characterized by active tissue reconstruction. Keratinocytes at the wound margins migrate and proliferate to restore epidermal continuity, while fibroblasts infiltrate the wound bed and synthesize a provisional ECM. Cytokines and growth factors transform fibroblasts into α-smooth muscle actin (α-SMA)-expressing myofibroblasts, which mediate wound contraction. Concurrently, angiogenesis restores vascular supply by forming new capillaries from endothelial cells. Epidermal stem cells originating from basal epidermal layers and hair follicle niches further contribute to tissue regeneration. Collectively, re-epithelialization, neovascularization, matrix deposition, and wound contraction re-establish tissue integrity during this stage [399], [400].

The final remodeling phase may continue for months or even years following injury. During this period, granulation tissue is gradually reorganized into mature scar tissue through continuous matrix synthesis, degradation, and remodeling. Type III collagen deposited during earlier phases is progressively replaced by mechanically stronger type I collagen, leading to increased tensile strength [401], [402]. However, healed skin rarely regains the structural organization of native tissue. Scar tissue typically lacks appendages such as hair follicles and sweat glands, exhibits reduced elastin content, and displays collagen fibers aligned in parallel rather than the basket-weave architecture of normal skin. Consequently, the tensile strength of healed tissue generally reaches only 50-80% of that of uninjured skin [403], [404]. Myofibroblasts remain important regulators during this stage by controlling collagen fibrillogenesis through ECM-associated molecules such as decorin. Prolonged inflammation, particularly in burn injuries, can further delay remodeling and contribute to excessive scar formation [403]. Overall, wound healing is a complex, immensely controlled process that functions through coordinated interactions among immune cells, stromal cells, signaling molecules, and ECM components. A deeper understanding of these processes is critical for developing advanced biomaterials and regenerative therapies to improve tissue repair outcomes [405], [406], [407], [408].

Recently, a rapid and practical approach for generating aggregation-induced emission luminogen (AIEgen)-based nanofibrous dressings directly at the wound site has been reported for the treatment of postsurgical and trauma-related infections [409]. Using a handheld ES device, N, N-diphenyl-4-(7-(pyridin-4-yl) benzo[c] [1,2,5] thiadiazol-4-yl) aniline functionalized with 1-bromoethane (TBP)-loaded PCL NFs were deposited in situ onto skin wounds as shown in Fig. 31, forming a conformal and adhesive dressing that adapts to irregular wound geometries while providing immediate protection. The portable system integrates a miniaturized high-voltage source and syringe pump, enabling on-demand fabrication of personalized dressings without conventional laboratory equipment [409]. TBP, the incorporated AIEgen, exhibits a strong antibacterial effect against Gram-positive bacteria, including methicillin-resistant *S. aureus* (MRSA), without inducing resistance. Photophysical analysis confirmed its aggregation-induced emission behavior, with enhanced fluorescence observed at higher toluene fractions due to aggregate formation. When embedded in PCL NFs, TBP exhibited loading-dependent fluorescence and efficient ROS generation under visible light irradiation, indicating potent photodynamic antibacterial activity [409]. The resulting NFs were uniform and defect-free, and cytocompatibility tests using HUVEC and NIH 3T3 cells confirmed good biocompatibility. In vitro antibacterial experiments demonstrated significant reductions in the viability of *S. aureus* and MRSA, accompanied by membrane disruption. In vivo, in situ-deposited AIE nanofibrous dressings hastened wound healing, reduced inflammation, and promoted tissue regeneration compared with control groups [409]. Overall, this work highlights handheld ES of AIEgen-loaded NFs as a rapid, customizable, and effective strategy for managing infected wounds.

**Fig. 31:**
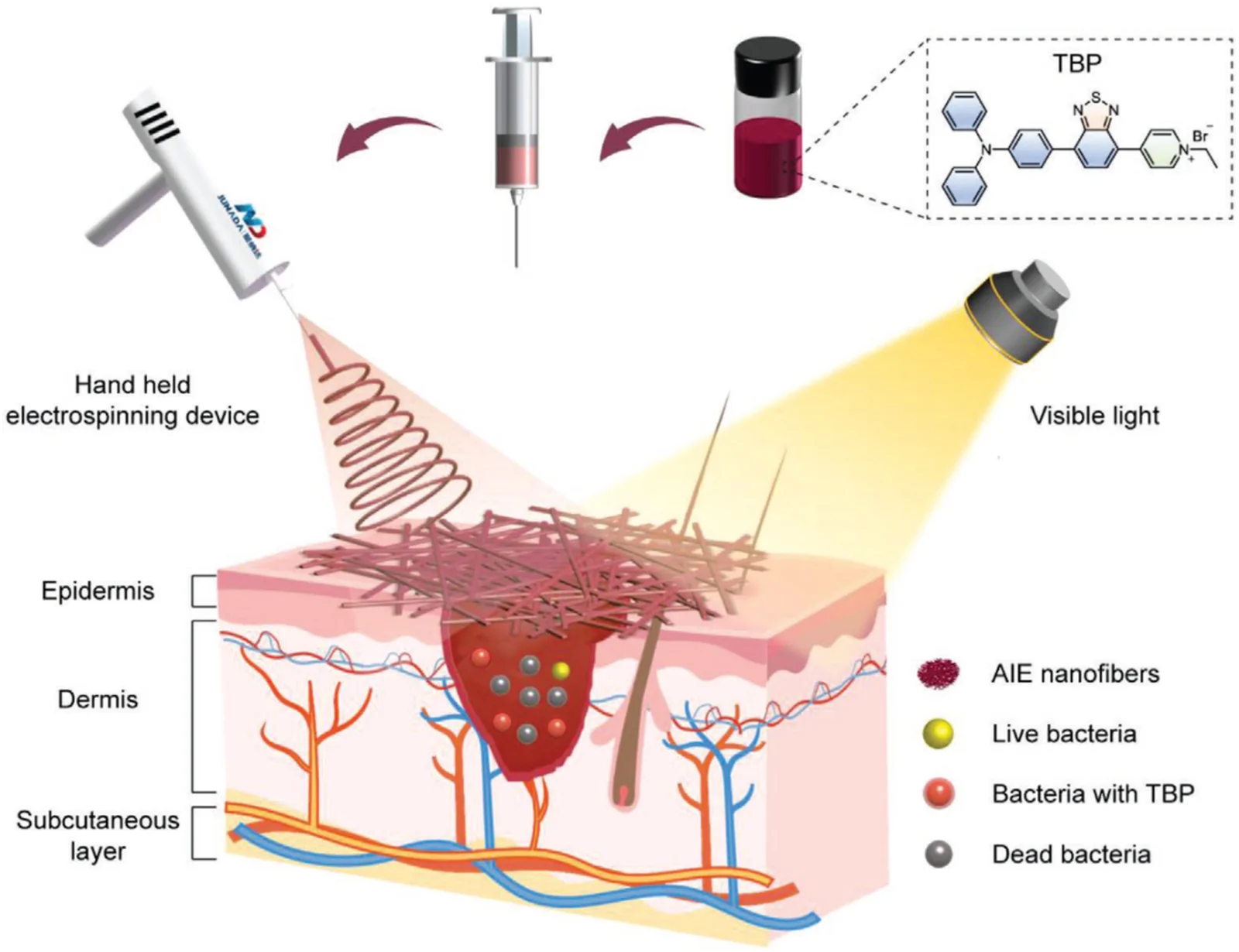
Illustration of the ES procedure for fabricating the AIEgen-incorporated antibacterial dressing [409]. Copyright 2024. Reproduced with permission from John Wiley & Sons.

In parallel, TA has been widely explored as a supramolecular cross-linker for assembling LBL block copolymer micelle (BCM)/TA films with swelling-deswelling behavior capable of regulating small-molecule release [410]. TA is particularly attractive due to its intrinsic pro-healing, antibacterial, and anti-inflammatory properties, including suppression of TNF-α, a key cytokine elevated in chronic wound environments [411], [412]. These characteristics make TA-based systems highly promising for wound healing applications. For instance, Albright et al. [413] developed BCM/TA coatings on PCL/collagen (PCL/Coll) NFs to create bioactive surfaces that enhance fibroblast adhesion, proliferation, and spreading, thereby accelerating wound closure. The BCM/TA layer can also be loaded with antibiotics, enabling infection control governed by swelling-dependent release kinetics. As illustrated in Fig. 32 [413], the system is modular and can be combined with TGF-β1-loaded PCL/Coll fibers to achieve antimicrobial properties and enhanced tissue regeneration simultaneously. In vitro wound-closure assays using normal human dermal fibroblasts (NHDFs) further confirmed the efficacy of this approach. Bare PCL/Coll NFs showed limited migration, with approximately 20% and 50% closure at 24 and 72 h, respectively. In contrast, BCM/TA-coated NFs achieved ∼60% and ∼80% closure over the same time points. This improvement was attributed to the synergistic effects of BCM/TA-induced surface topography and increased cell attachment, which together promoted fibroblast migration and proliferation, key processes in effective wound healing [413].

**Fig. 32:**
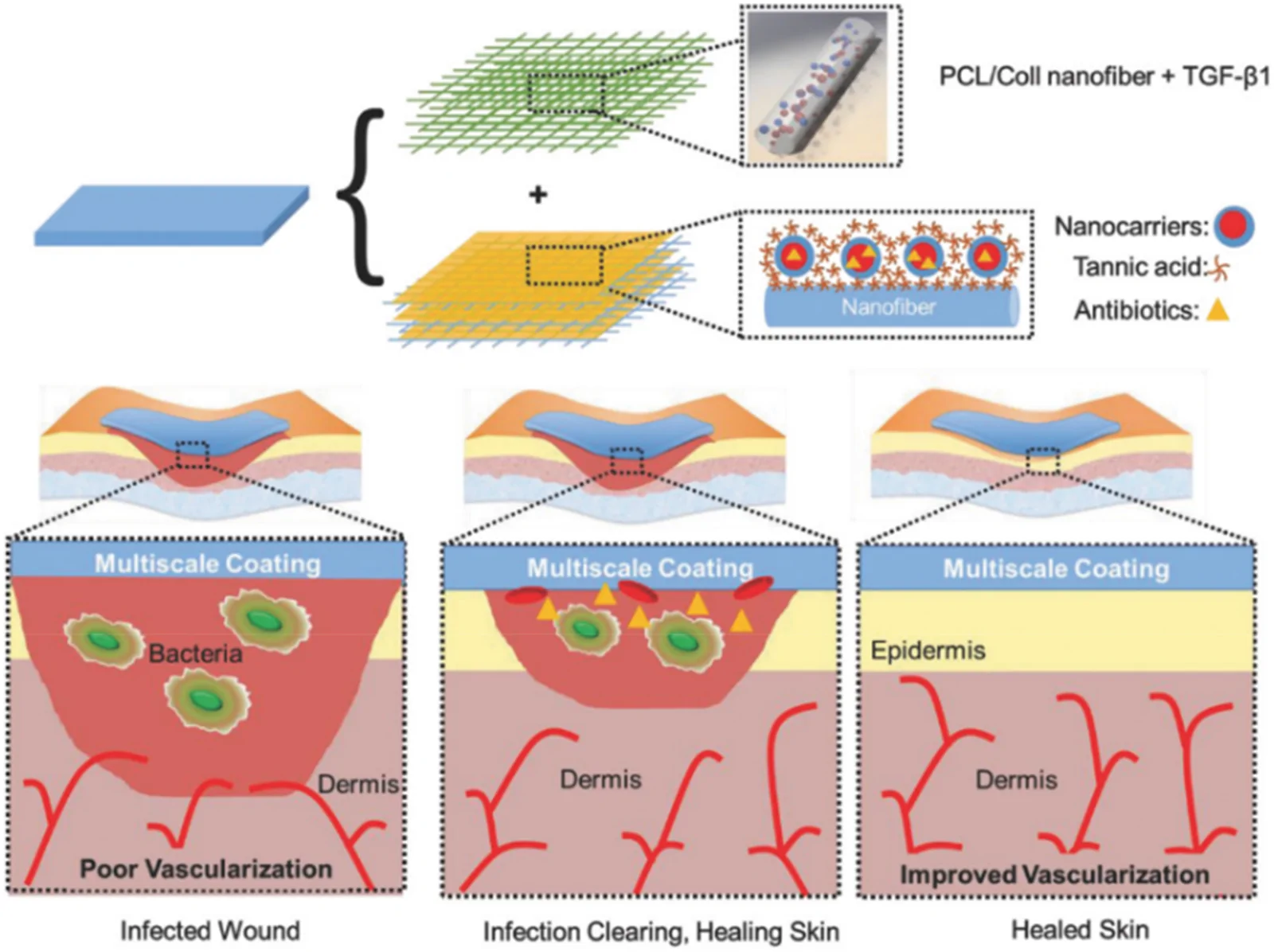
Modular PCL/Coll platform: BCM/TA micellar coatings for antibiotic delivery plus TGF-β1-loaded fibers for myofibroblast induction [413]. Copyright 2018. Reproduced with permission from John Wiley & Sons.

Electrospun dressings have been extensively explored as wound management tools, as their nanofibrous architecture provides both mechanical support and interconnected transport pathways that facilitate cell infiltration and signaling [414]. However, even with standard care, early-stage infection and dysregulated inflammation can impair healing, driving the engineering of multifunctional dressings that combine physical support with localized delivery of anti-infective and anti-inflammatory agents [415]. Various approaches have been explored, including antibiotic-loaded fibers [416], silver nanoparticle-based composites with broad antimicrobial activity [417], [418], and gold (Au) NPs functionalized with small molecules like 6-aminopenicillanic acid for combating multidrug-resistant bacteria [419]. In vivo studies have shown that PCL-gelatin NFs decorated with surface-modified Au NPs significantly reduce bacterial load and improve healing outcomes in infected wound models [419]. In addition, local delivery of non-steroidal anti-inflammatory drugs (e.g., naproxen, ibuprofen, and ketoprofen) has been incorporated into NFs to modulate excessive inflammation [420].

In a related study, the in vivo efficacy of coaxial nanofiber dressings was assessed in a diabetic wound model [421]. Macroscopic analysis showed progressive wound closure in all groups, with both coaxial blank PVP/PLA and AS/PVP/PLA functional dressings outperforming conventional medical gauze (Fig. 33a). From day 6 onward, the AS/PVP/PLA group exhibited the fastest healing, accompanied by minimal edema, erythema, and inflammation, whereas the gauze group showed mild exudation and irritation. Quantitative analysis confirmed this trend: on day 3, wound closure rates were 10.84 ± 1.80% (gauze), 14.27 ± 0.96% (blank PVP/PLA), and 18.76 ± 1.82% (AS/PVP/PLA) (Fig. 33b), with significantly higher healing rates observed in the functional dressing group at subsequent time points. By day 14, the AS/PVP/PLA group achieved 96.54 ± 0.29% wound closure [421]. Histological analysis further supported these findings. H&E staining on day 7 revealed inflammation, localized hemorrhage, and limited collagen deposition across all groups. By day 14, the gauze-treated wounds demonstrated incomplete re-epithelialization and sparse collagen production, whereas the blank group exhibited partial closure but persistent hemorrhage and reduced collagen density. Nevertheless, the AS/PVP/PLA group demonstrated a fully re-formed epidermis, a markedly thicker granulation layer, and substantially enhanced collagen deposition. Masson's trichrome staining confirmed denser and more organized collagen fibers in the functional dressing group, along with increased fibroblast presence and alignment (Fig. 34) [421]. Collectively, these findings demonstrate that coaxial nanofiber dressings significantly accelerate wound closure and improve tissue regeneration in diabetic wounds.

**Fig. 33:**
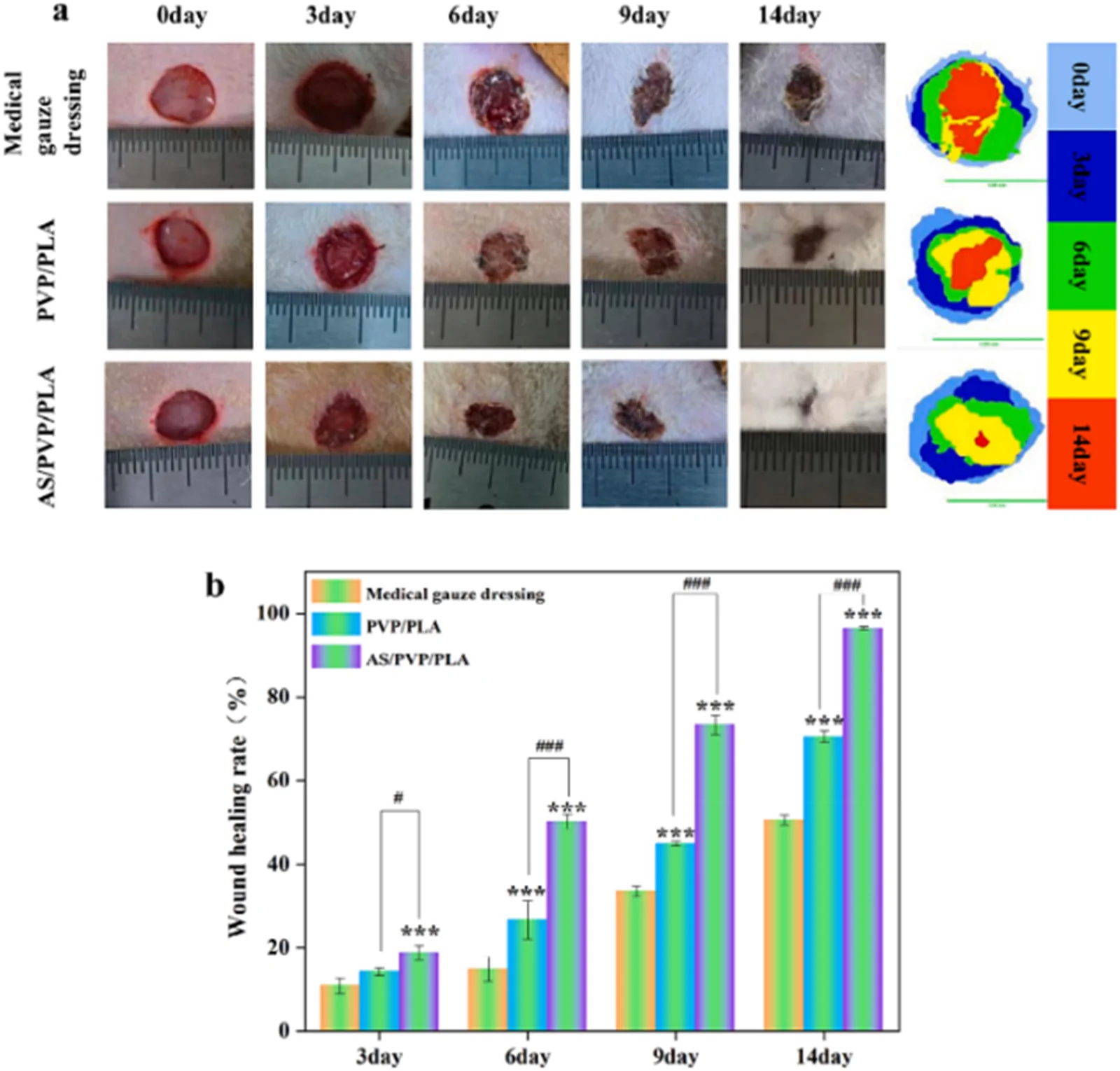
Evaluation of wound healing progression. (a) Photographs illustrating macroscopic wound closure at sequential time points, accompanied by a schematic depiction of the healing process. (b) Quantitative assessment of wound closure percentages over time (n = 3). Statistical comparisons are made against medical gauze dressings (*P < 0.05, **P < 0.01, ***P < 0.001) and against PVP/PLA membranes (#P < 0.05, ##P < 0.01, ###P < 0.001) [421]. Copyright 2024. Reproduced with permission from Elsevier.

**Fig. 34:**
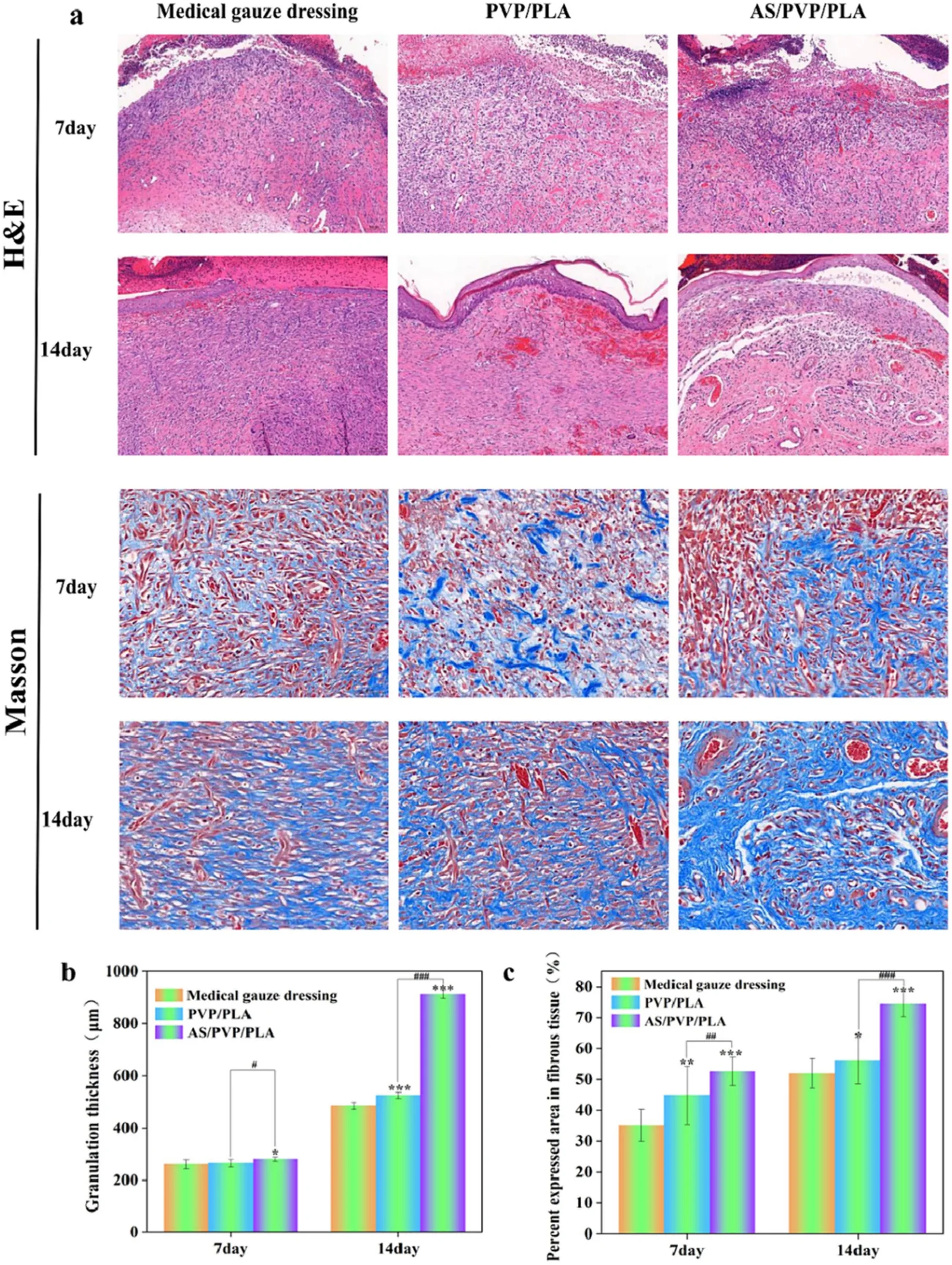
Histological examination of wound tissue sections at various time points. (a) Images of H&E and Masson's trichrome staining from each treatment group (scale bar = 100 μm). (b) Quantitative assessment of granulation tissue thickness across wound groups (n = 3). (c) Quantitative evaluation of collagen deposition area in different wound tissues (n = 3). Statistical differences are denoted as follows: relative to medical gauze (*P < 0.05, **P < 0.01, ***P < 0.001) and relative to PVP/PLA (#P < 0.05, ##P < 0.01, ###P < 0.001) [421]. Copyright 2024. Reproduced with permission from Elsevier.

Chen et al. [422] prepared a curcumin-loaded sandwich-like NF membrane (CSNM) using sequential ES, as depicted in Fig. 35, for hemostasis, antibacterial activity, and accelerated wound healing. Both in vitro and in vivo hemostatic assays demonstrated that CSNM exhibited excellent blood-clotting performance. DPPH-based evaluation of the antioxidant activity showed that CSNM possessed strong free radical scavenging activity. In addition, antibacterial testing using the disk diffusion method confirmed its effective antimicrobial properties. In a rat dorsal skin defect model, CSNM significantly promoted collagen deposition, granulation tissue formation, and epithelial tissue remodeling. Immunohistochemical analysis further revealed that CSNM enhanced early expression of CD31 and TGF-β, thereby facilitating angiogenesis and accelerating wound closure [422]. In another work, the therapeutic effect of SMGT on diabetic wounds was evaluated using a streptozotocin-induced diabetic rat model [423]. Progressive hyperglycemia was observed, with blood glucose levels reaching approximately 28 mM by week six (Fig. 36a). Wound regions were photographed on a specific schedule (0, 3, 6, 9, and 12 days), and contraction rates were quantified from the images (Fig. 36b). As expected, severe hyperglycemia disrupted skin histoarchitecture and significantly delayed wound closure, whereas treatment with SMGT-NFM accelerated healing and improved tissue regeneration (Fig. 36c and d) [423]. The expression of α-smooth muscle actin (α-SMA), a marker of myofibroblast activity and neovascular maturation, was assessed by immunohistochemistry. Because timely activation and subsequent resolution of myofibroblasts are critical to preventing fibrosis, α-SMA expression provides important insight into wound remodeling. In the high-glucose (HG) group, SMGT combined with NFM restored more normal cutaneous architecture and promoted appropriate spatial localization of α-SMA within regenerated tissue, indicating improved wound maturation and controlled repair [423].

**Fig. 35:**
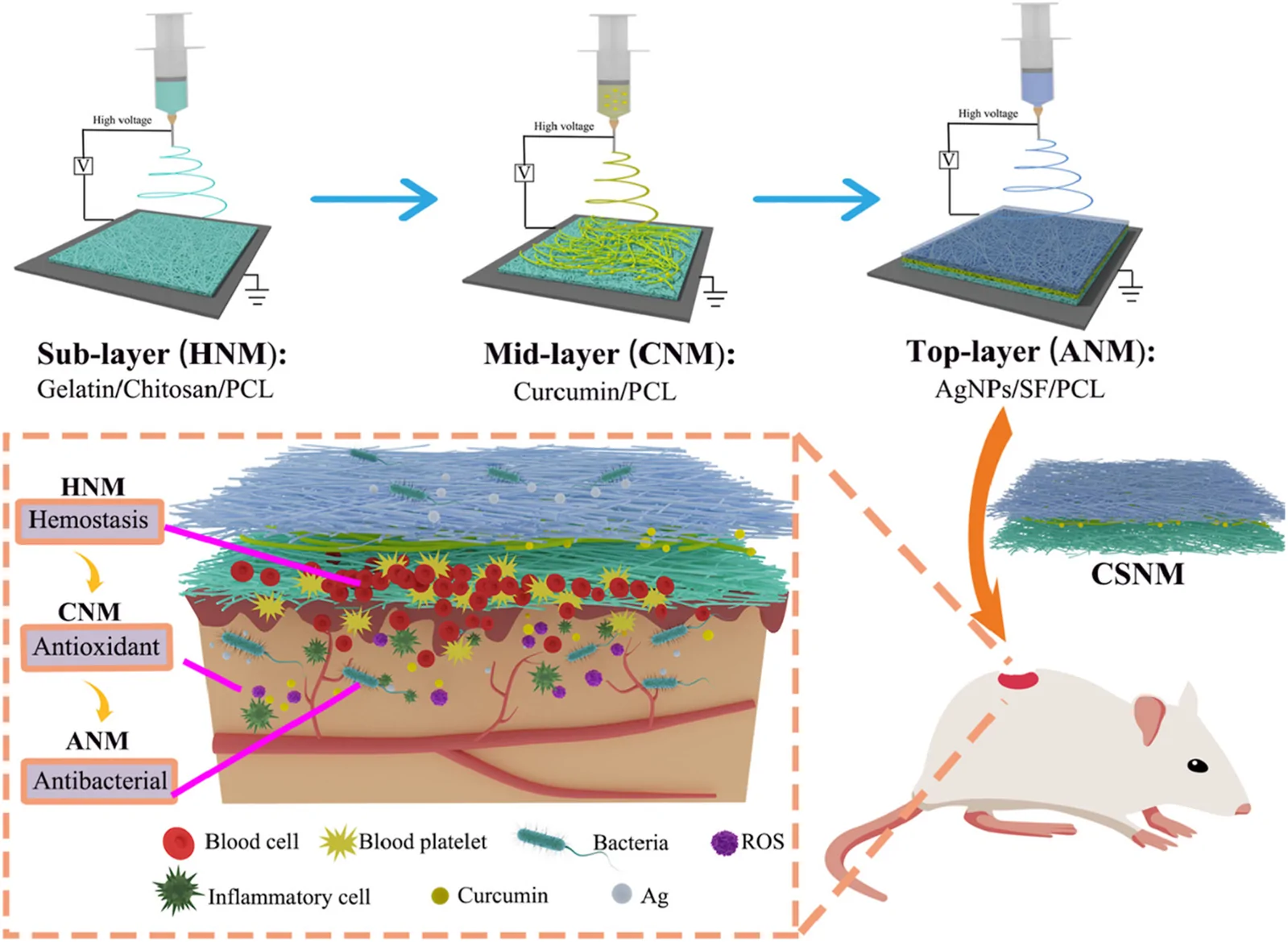
Schematic illustration of CSNM preparation using sequential ES [422]. Copyright 2021. Reproduced with permission from Elsevier.

**Fig. 36:**
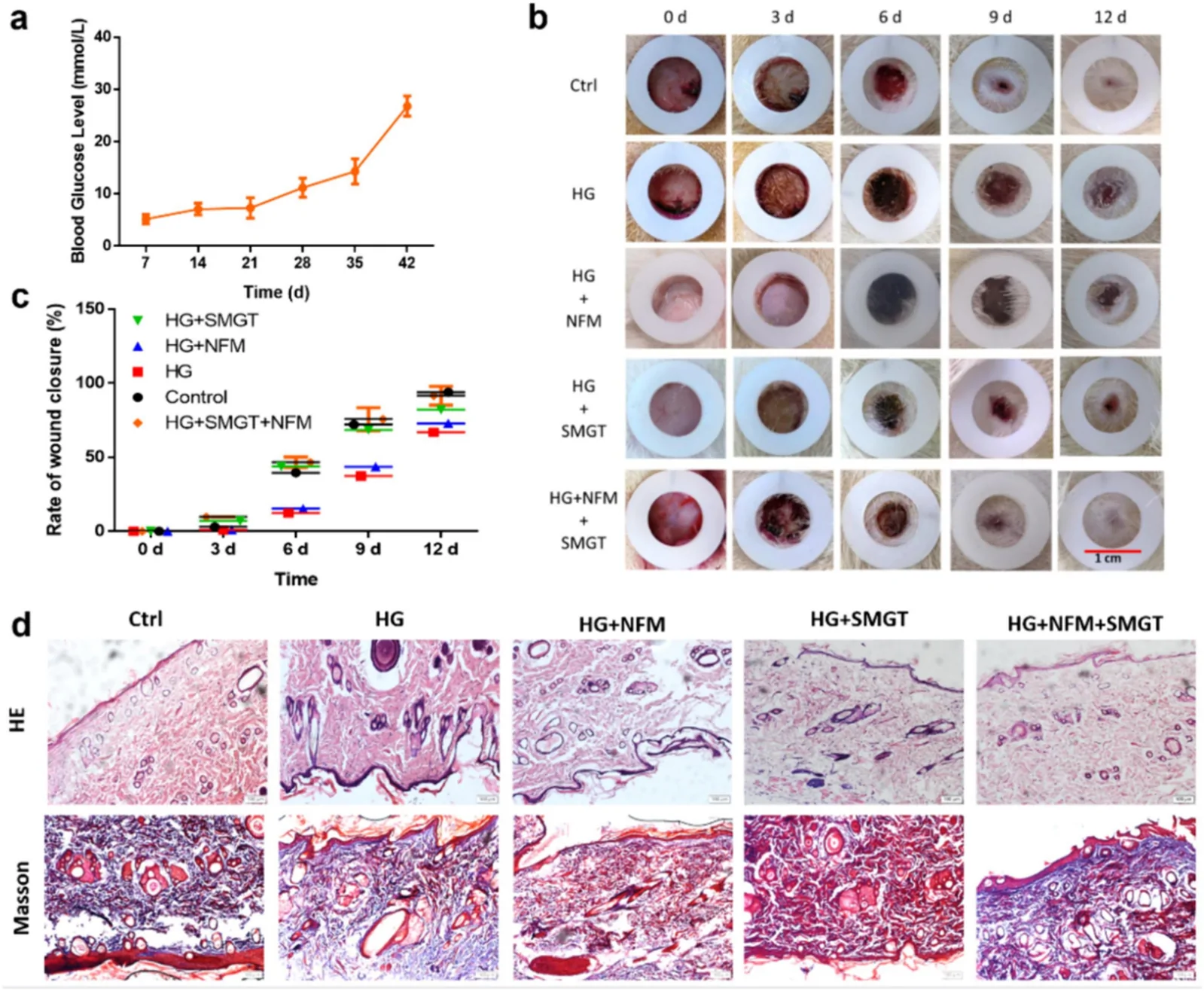
SMGT enhances wound healing in a diabetic rat model. (a) Sequential changes in blood glucose levels in diabetic rats over time. (b) Representative photographs illustrating wound closure under different treatment regimens (scale bar = 1 cm). (c) Quantitative evaluation of wound closure rates, calculated from four randomly chosen fields using Image-Pro Plus software. (d) Histological assessment of skin tissue, including H&E staining (top) and Masson's trichrome staining (bottom). Experimental groups include Ctrl (healthy rats), HG (untreated hyperglycemic rats), HG + NFM (hyperglycemic rats treated with nanofibers lacking semaglutide), HG + SMGT (hyperglycemic rats treated with semaglutide), and HG + NFM + SMGT (hyperglycemic rats treated with semaglutide-loaded NFa). Illustrative images were obtained from six random fields. Data are expressed as mean ± standard deviation (n ≥ 3). ****P < 0.001 [423]. Copyright 2024. Reproduced with permission from Elsevier.

Hyaluronic acid, a major component of the ECM, plays a key role throughout all phases of the wound-healing process. Throughout the hemostatic and inflammatory phases, it contributes to provisional matrix formation that stabilizes the wound and supports early fibrin-based scaffold formation [424]. In the earliest and mid-stages of healing, hyaluronic acid facilitates leukocyte and fibroblast infiltration, enhances clearance of necrotic tissue, and suppresses excessive inflammatory signaling by modulating macrophage phenotype and TLR4 activity [425]. In the proliferative phase, it promotes keratinocyte and fibroblast proliferation and stimulates ECM synthesis, thereby increasing collagen and elastin deposition to support granulation tissue formation and angiogenesis [426]. During the remodeling phase, hyaluronic acid regulates matrix turnover by interacting with TGF-β and matrix metalloproteinases, contributing to balanced collagen organization rather than directly inducing myofibroblast differentiation [427]. Despite these beneficial roles, native hyaluronic acid exhibits weak tissue adhesion, limited antimicrobial activity, and insufficient intrinsic regenerative capacity, which has motivated the development of hyaluronic acid -based composite dressings and cross-linked hydrogels to enhance its stability and bioactivity [428].

DA, a catecholamine neurotransmitter involved in vasodilation, insulin regulation, and immune modulation, offers complementary advantages. Its catechol structure enhances tissue adhesion, improves antioxidant capacity, and increases biocompatibility [429]. To overcome the limitations of hyaluronic acid, DA was grafted onto hyaluronic acid, followed by amidation with gelatin (GEL) after acetic acid removal, enabling the fabrication of DA-modified hyaluronic acid (OHDA)/GEL-based nanofiber dressings [430]. This strategy produced multifunctional scaffolds with antimicrobial, anti-inflammatory, and pro-healing characteristics for diabetic wound management, as shown in Fig. 37 [430]. In the diabetic wound environment characterized by hyperglycemia, high bacterial load, and elevated levels of inflammatory mediators, untreated wounds initially exhibited larger areas with delayed contraction [430]. By day 7, wounds treated with OHDA/GEL or cordycepin (COR)/OHDA/GEL NF membranes displayed wound contraction, whereas control wounds remained inflamed with visible exudate. After two weeks, the control group showed only limited healing, while both treatment groups exhibited substantial closure, with COR/OHDA/GEL performing best. By day 21, large unhealed regions persisted in the control group, whereas complete wound closure was noted in the COR/OHDA/GEL group. The histological analysis further supported these findings. In the control group, a thin epidermis, sparse collagen deposition, and delayed granulation and re-epithelialization indicated impaired fibroblast activity and matrix formation. In contrast, both OHDA/GEL and COR/OHDA/GEL groups showed well-developed granulation tissue, increased and well-organized collagen fibers, complete re-epithelialization, and partial regeneration of skin appendages, including hair follicles. The COR/OHDA/GEL group demonstrated the most advanced tissue remodeling, indicating superior collagen organization and more effective functional skin regeneration [430].

**Fig. 37:**
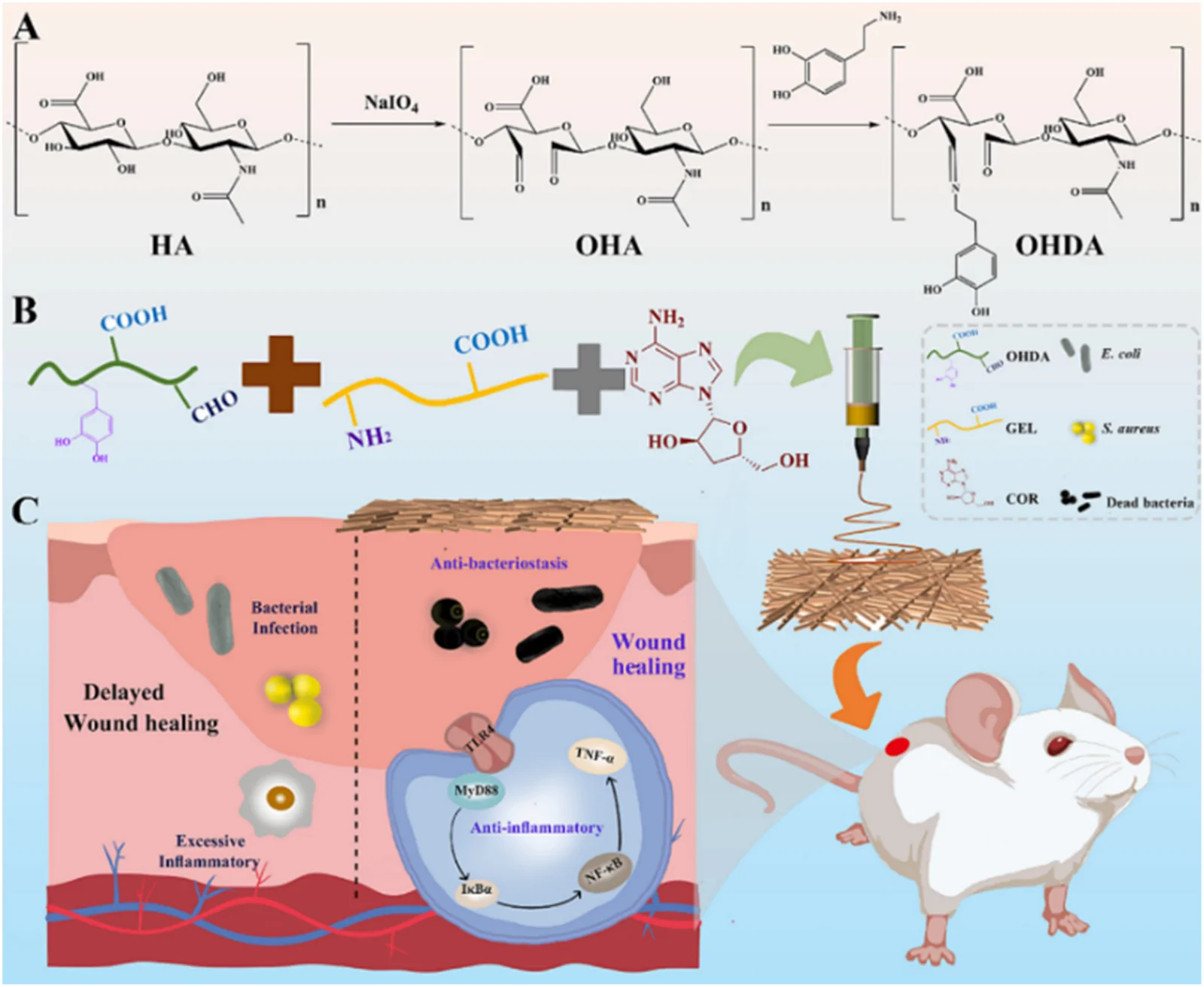
(A) Hyaluronic acid oxidation and OHDA preparation; (B) COR/OHDA/GEL nanofiber membrane preparation; (C) Wound healing mechanism of COR/OHDA/GEL NF wound dressing for diabetic mice [430]. Copyright 2024. Reproduced with permission from Elsevier.

Despite their advantages, many electrospun membranes are dense 2D structures with limited pore size, poor interconnectivity, and low fluid absorption capacity, which can restrict 3D cell infiltration and biofluid transport [431]. Gas-foaming has been employed to convert these structures into 3D nanofiber sponges and to incorporate functional agents such as TA, curcumin, and Ag-metal-organic frameworks (MOFs), thereby enhancing porosity, water uptake, and ECM-like behavior [431], [432]. Owing to their greater structural similarity to native tissue, 3D sponges better support cell adhesion, proliferation, and migration than conventional 2D mats [433]. To address MRSA-infected wounds, a layered electrospun 3D nanofiber sponge (3D-ZnMOF) was developed from CS, pullulan, and citric acid, with in situ incorporation of ZnMOF (Fig. 38) [434]. An MRSA-infection wound model in rats was used to compare gauze (control) with three nanofiber-based dressings. Wound images recorded on days 0, 3, and 9 demonstrated progressive contraction and infection status (Fig. 39A-C) [434]. On day 3, wound closure rates were 21.7% (control), 37.9% (2D-T/D/P/C), 47.4% (3D-T/D/P/C), and 53.8% (3D-ZnMOF). By day 9, closure increased to 82.46 ± 5.28% (control), 92.99 ± 1.25% (2D-T/D/P/C), 94.7% (3D-T/D/P/C), and 97.4% (3D-ZnMOF), indicating that all nanofiber dressings significantly improved healing, with 3D-ZnMOF performing best. Bacterial load analysis from wound exudates cultured on agar showed abundant colonies in the control and 3D-T/D/P/C groups on day 3, fewer colonies in the 2D-T/D/P/C group, and the lowest bacterial burden in the 3D-ZnMOF group, consistent with in vitro antibacterial performance (Fig. 39B) [434]. Histological evaluation further supported these findings. H&E staining at day 9 revealed minimal inflammatory infiltration in the 3D-ZnMOF group, whereas numerous neutrophils persisted in the control and, to a lesser extent, in the 2D- and 3D-T/D/P/C groups (Fig. 39D, E) [434]. Evidence of neovascularization was observed in all groups (blue arrows), while newly formed mature vessels and hair follicles were more evident in the 3D-T/D/P/C and 3D-ZnMOF groups (green arrows). Masson's trichrome staining demonstrated the highest collagen deposition and most organized fiber alignment in the 3D-ZnMOF group, followed by 3D-T/D/P/C and 2D-T/D/P/C, with the lowest levels in the control group. Quantitative analysis of collagen coverage on day 9 showed values of 51.6% (control), 57.3% (2D-T/D/P/C), 64.3% (3D-T/D/P/C), and 70.1% (3D-ZnMOF) (Fig. 39 F, G) [434]. Overall, nanofiber-based dressings enhanced wound healing and collagen regeneration, with the 3D-ZnMOF system exhibiting the most pronounced therapeutic effect, whereas commercial gauze led to more pronounced scarring. Moreover, rapid hemostasis remains a critical clinical challenge [434].

**Fig. 38:**
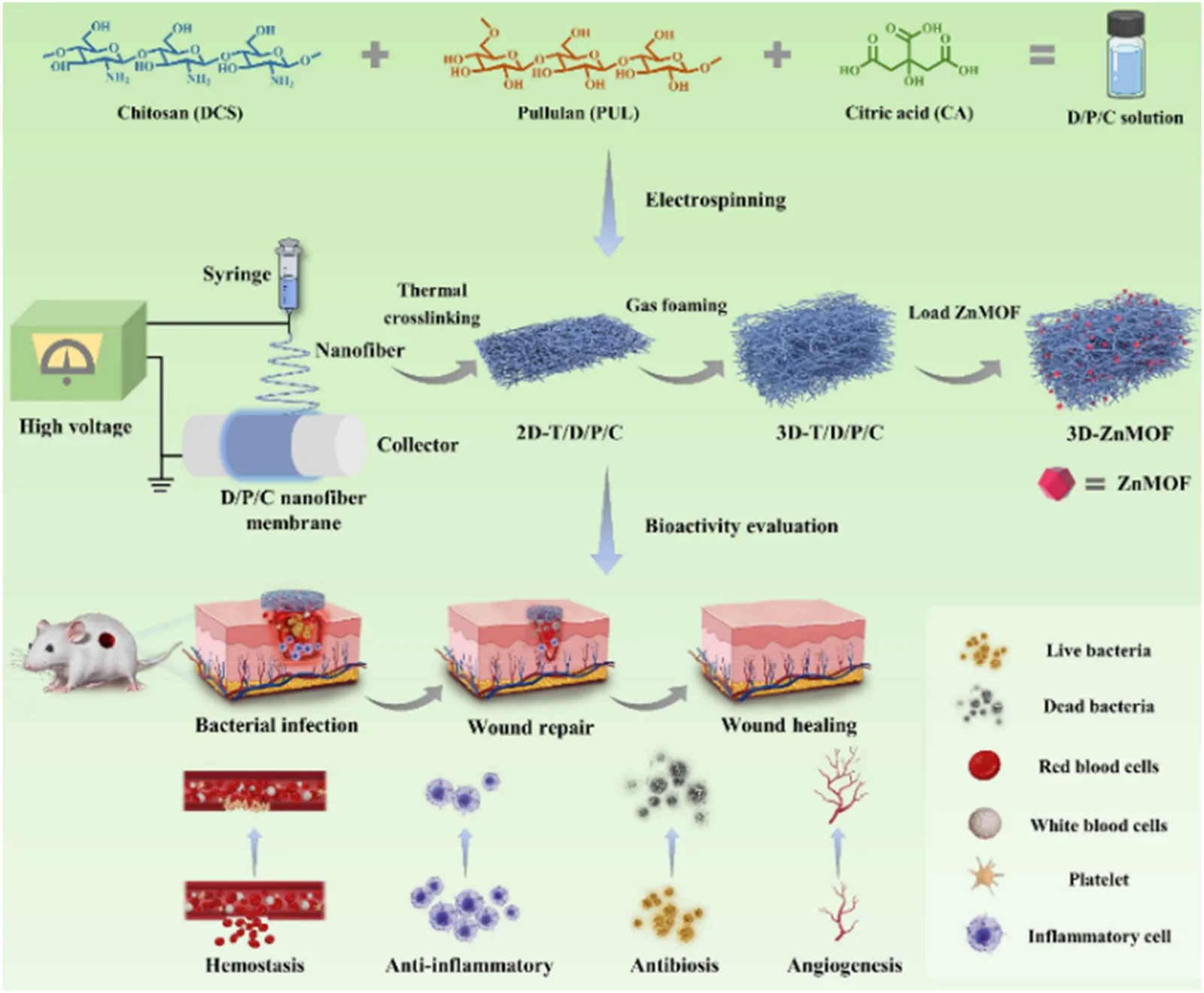
Technique of preparation and evaluation of 3D-ZnMOF nanofiber sponge activity [434]. Copyright 2024. Reproduced with permission from Elsevier.

**Fig. 39:**
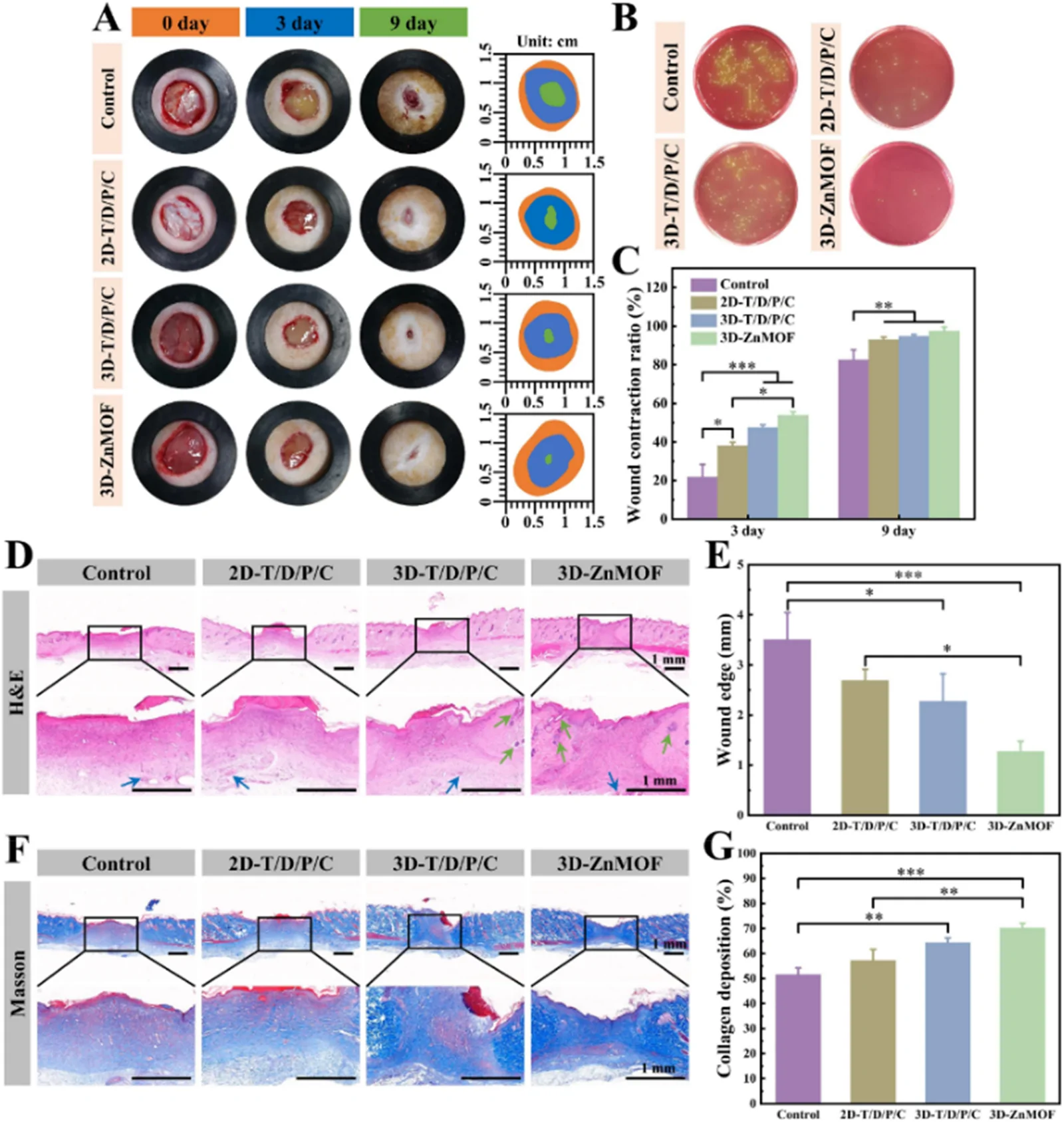
MRSA-infected wounds exhibit accelerated healing when covered with 2D/3D nanofiber dressings. (A) A diagram of the wound region and typical images of MRSA-infected wounds at days 0, 3, and 9 post-treatments. (B) Agar plate images showing MRSA colonies derived from tissue rinses on day 3. (C) Comparative wound contraction rates between control and nanofiber-treated groups over time. (D) Histological views of regenerated skin via H&E staining. (E) Quantification of wound edge lengths across groups. (F) Masson trichrome staining of regenerated skin. (G) Collagen coverage measurements at wound sites [434]. Copyright 2024. Reproduced with permission from Elsevier.

Rathinavel et al. studied the in vivo wound-healing functionality of PVA NFs integrated with amine-modified mesoporous silica (NH_2_-SBA-15) and curcumin [435]. Using Albino Wistar rats, they compared electrospun PVA/NH_2_-SBA-15 (5.0 wt%) and PVA/NH_2_-SBA-15 (5.0 wt%)/curcumin NFs over days 0, 4, 10, 14, and 18. Their earlier work had also evaluated PVA, saline controls, and SBA-15-loaded PVA/curcumin systems. In the early stage (day 4), wound closure was limited; by day 10, noticeable improvement in healing was observed. At day 14, curcumin-loaded NFs exhibited pronounced tissue regeneration. By day 18, the PVA/NH_2_-SBA-15/curcumin group achieved nearly complete wound closure (∼98% reduction in wound area) along with healthy hair regrowth, outperforming the curcumin-free NH_2_-SBA-15/PVA system. No evidence of inflammation, infection, or allergic response was detected, confirming good biocompatibility and suitability for skin applications. The synergistic effect of PVA, NH_2_-modified SBA-15, and curcumin contributed to accelerated healing. SBA-15 provided a high-surface-area, porous structure with strong drug-loading capacity, serving as an efficient curcumin carrier. At the same time, surface amine groups enhanced bioactivity and supported cellular processes involved in tissue repair [435]. To further evaluate macroscopic healing, Liao et al. analyzed wound images collected on days 3, 7, and 14 after dressing removal (Fig. 40A) [436]. Image analysis was used to quantify residual wound area relative to baseline, enabling comparison between nanofiber membrane (NFM) treatments and gauze controls. In diabetic wounds, PCH and PC NFs promoted faster healing than PLGA and gauze, with PCH showing the best performance, consistent with the superior efficacy of curcumin-based NFMs observed in vitro. By day 3, wound areas were already reduced in PCH (58.3%) and PC (58.1%) compared with gauze (66.2%) (Fig. 40B) [436]. On day 7, all NFM-treated groups significantly outperformed the control, with PCH beginning to show clear superiority over PLGA. By day 14, the curcumin-heparin composite (PCH) achieved the most rapid closure, reflecting synergistic anti-inflammatory and pro-angiogenic effects. Final wound-area reductions were lowest with PCH (13.9%) and highest with gauze (37.1%), followed by PC (20.5%) and PLGA (27.4%). Overall, these results confirm that dual-component NFMs significantly enhance wound contraction and accelerate tissue regeneration, particularly in diabetic wound environments [436].

**Fig. 40:**
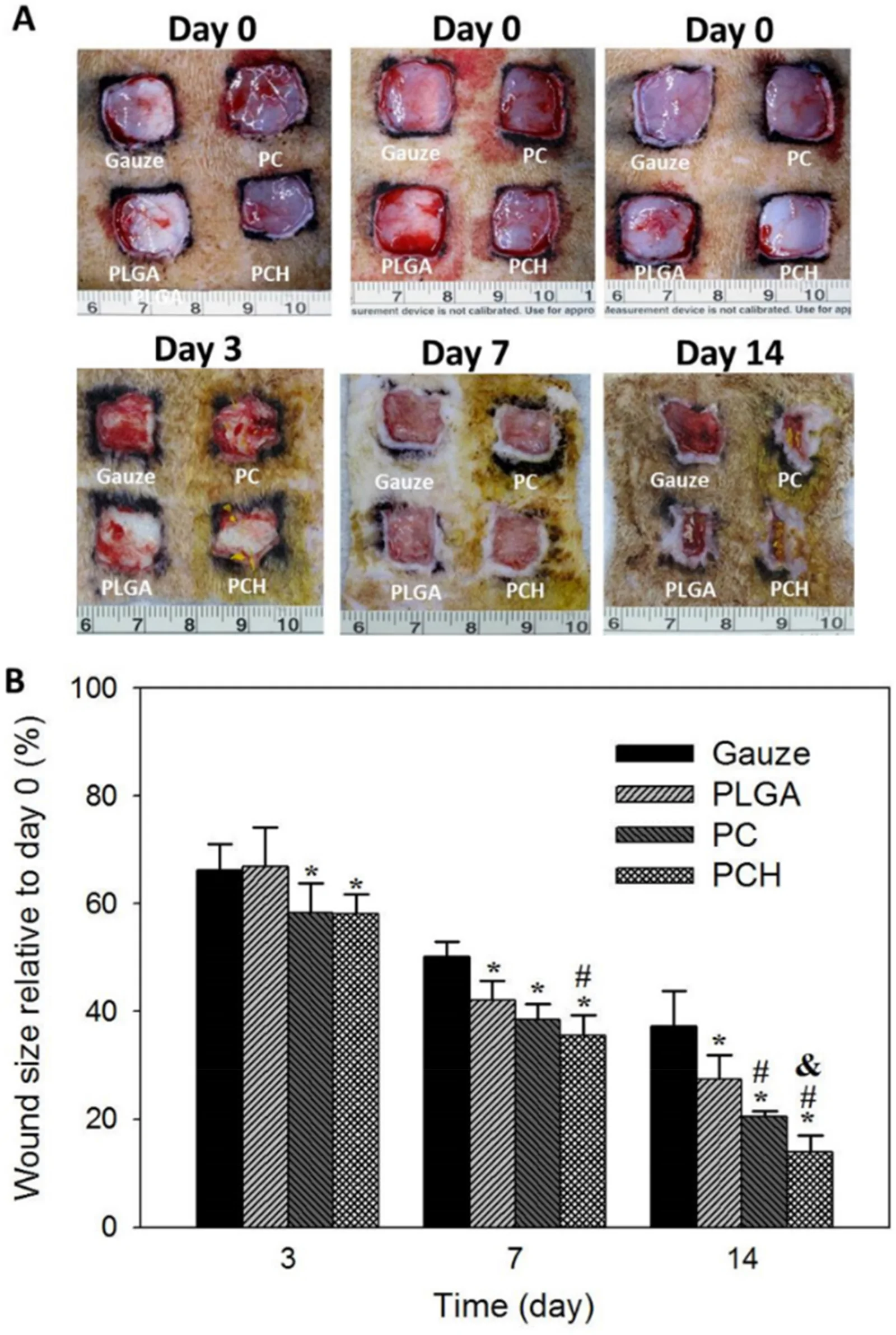
PLGA vs PC vs PCH comparison in diabetic rats; PCH superiority by day 14 [436]. Copyright 2021. Reproduced with permission from Elsevier.

### 4.4 Antibacterial agent

ESNFs are a highly versatile tool for antibacterial applications given their large interfacial surface area, adjustable porosity, and easily modifiable surface chemistry, which enable the efficient incorporation of diverse antimicrobial agents [437], [438]. CS, a derivative of chitin, is one of the most extensively studied natural polysaccharides owing to its intrinsic antimicrobial activity and biocompatibility. However, its poor solubility and limited stability in aqueous media can restrict processing and practical applications [439]. Similarly, cellulose and its derivatives represent another important class of biopolymers for antimicrobial NFs. Native cellulose is difficult to electrospin because of its low solubility; therefore, CA, which is soluble in solvents such as DMF and acetone, is commonly used instead [438]. To overcome limitations in spinnability and functionality, CS and related biopolymers are often blended with synthetic polymers, NPs, or biomolecules to improve physical features and introduce multiple antibacterial mechanisms [437], [440]. Current strategies increasingly focus on functionalizing NFs with metallic NPs (e.g., ZnO, TiO_2_), antibiotics, natural antimicrobial agents, engineered nanostructures, or multilayer coatings. These systems typically act through contact killing, oxidative stress generation, and controlled drug release [441]. Such modular designs enable precise tuning of antimicrobial activity, release kinetics, and cytocompatibility for biomedical and environmental applications [442].

Evaluation of antibacterial performance in ESNFs is commonly performed using standardized assays, including direct-contact, dynamic-contact, and agar-diffusion methods, which simulate different bacterial-material interaction scenarios. The most frequently used bacterial strains are *S. aureus* (Gram-positive) and *K. pneumoniae* or *E. coli* (Gram-negative), with *E. coli* often preferred due to its lower biosafety requirements and wide availability [443], [444]. These assays are primarily conducted in vitro using bacterial cultures in agar or liquid media to assess inhibition zones, colony reduction, or minimum inhibitory concentration (MIC). However, in vivo evaluations remain limited, especially in biomedical applications such as wound dressings, sutures, implants, and filtration systems, where physiological complexity and immune responses significantly influence performance [445]. In this context, the following section highlights representative functionalization strategies and their antibacterial performance, focusing on material integration approaches, synergistic nanostructures, and both in vitro and in vivo studies relevant to wound healing and antimicrobial barrier systems.

Antimicrobial functionality typically requires incorporation of active agents such as metallic NPs or photosensitizers [446]. Huang et al. reported electrospun PVP/CNC/Ag NFs with tunable morphology and strong antibacterial activity [447]. The addition of CNCs and silver NPs (AgNPs) increased solution conductivity, resulting in uniform, thinner fibers. The PVP/CNC/AgNO_3_ NFs exhibited clear inhibition zones against both *E. coli* and *S. aureus*, whereas CNC-only mats showed no antibacterial activity (Fig. 41) [447]. This effect was attributed to Ag^+^ release and nanoparticle interactions with bacterial membranes, leading to disrupted permeability and impaired respiration. Similarly, He et al. developed AgNP-loaded feather keratin (FK)/PVA/PEO NFs with strong antibacterial performance mediated by silver-induced membrane disruption [448]. Increasing AgNP content (0.6-3%) progressively enhanced antimicrobial activity against both *E. coli* and *S. aureus*, whereas Ag-free pads showed no inhibition zones. At optimal loading, the NFs produced clear inhibition halos, with larger zones observed for *E. coli* than for *S. aureus*, suggesting higher sensitivity of Gram-negative bacteria [448].

**Fig. 41:**
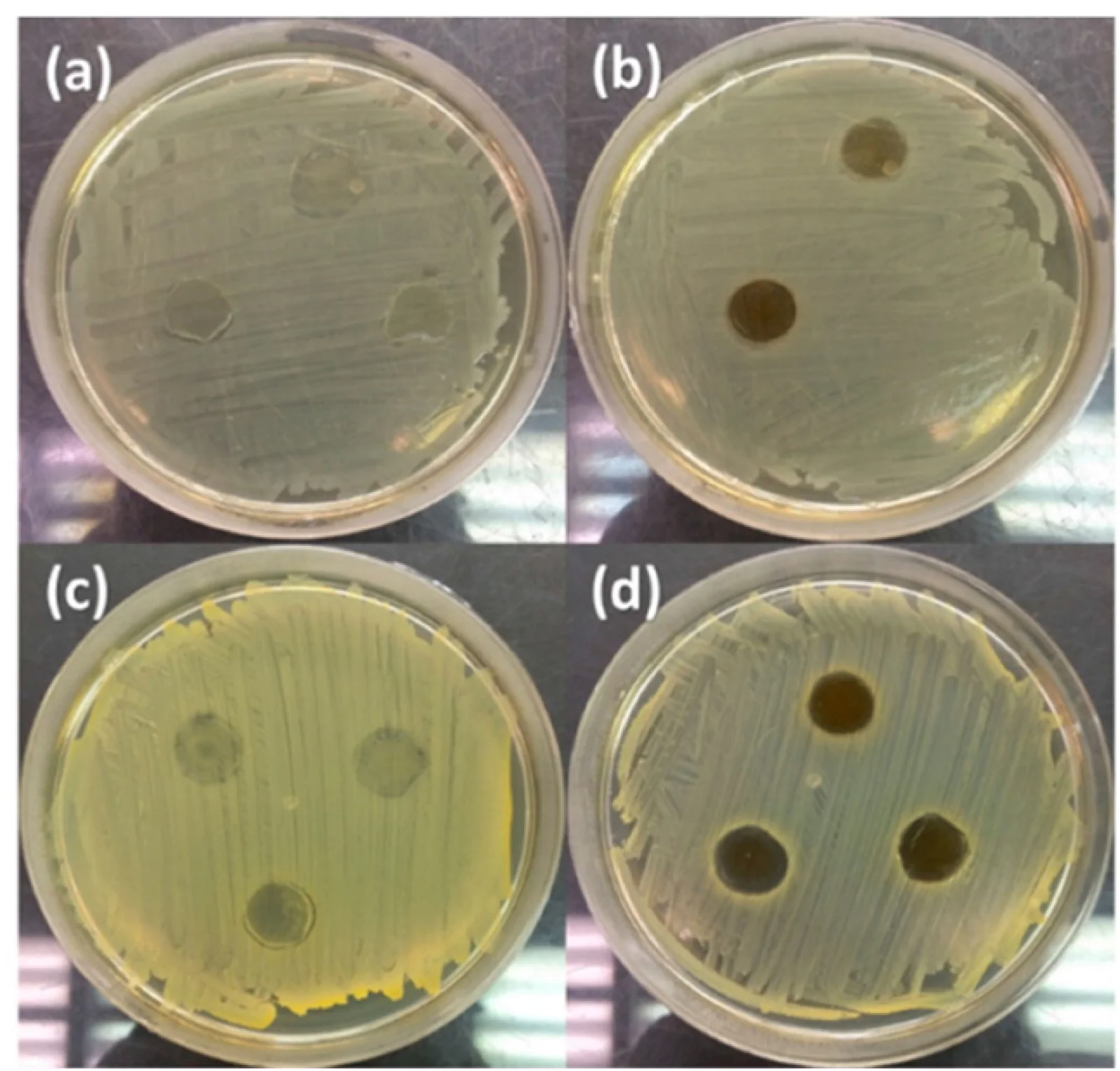
Images illustrating the inhibitory effects of PVP/CNC on *E. coli* (a) and *S. aureus* (c), alongside the effects of PVP/CNC-4%/AgNO_3_-0.34% on *E. coli* (b) and *S. aureus* (d) [447]. Copyright 2016. Adapted from MDPI.

Beyond AgNPs, a range of metal-based NPs, including zinc, titanium, copper, cobalt, and hybrid systems such as Zn/Ti, have been explored for antibacterial applications via contact-killing mechanisms and ROS-mediated activity similar to silver-based systems [449], [450], [451], [452]. For example, Kenawy et al. [450] developed ZnO-loaded PVA/SA NFs exhibiting strong antimicrobial performance, particularly when combined with glycine. Their optimized formulation (AS 3.3: 10% PVA/1.5% SA/10% CA/0.75% ZnO/0.5% glycine) produced the largest inhibition zones against multiple multidrug-resistant pathogens, reaching 23.15 ± 2.34 mm for *Bacillus cereus* and 22.22 ± 2.65 mm for *Staphylococcus aureus*. Gram-negative strains also showed significant susceptibility, with *E. coli* exhibiting inhibition zones up to 17.45 ± 3.21 mm. Glycine-containing NFs consistently outperformed control formulations, and biofilm assays confirmed inhibition rates exceeding 97% for Gram-positive bacteria. In addition, time-kill kinetics demonstrated nearly complete eradication of *B. cereus* and *S. paratyphi* within 48 h, highlighting the synergistic antibacterial impact of ZnO NPs and glycine [450]. Similarly, Devi et al. revealed the potent antibacterial action of metal nanoparticle-doped polyacrylonitrile (PAN) NFMs [452]. ZnO-based (PZCR) and Cu-based (PCCR) NFs, both functionalized with CS and the thiourea-derived antimicrobial compound R1, achieved up to 95% reduction in bacterial growth against *E. coli* and *S. aureus*. The zones of inhibition reached 12-13 mm for *E. coli* and 20-22 mm for *S. aureus*, indicating particularly strong activity against Gram-positive bacteria. The enhanced antimicrobial performance is attributed to the combined effects of (i) electrostatic membrane disruption by cationic CS, (ii) intrinsic antibacterial activity of R1, and (iii) ROS generation induced by ZnO and Cu NPs [452]. In another work, PLGA/CuO hybrid nanofiber scaffolds were engineered to impart strong antimicrobial activity to otherwise inert PLGA mats [453]. While pristine PLGA produced no inhibition zones, CuO-loaded NFs generated clear bactericidal halos against both *S. aureus* and *E. coli*. Optical density measurements further confirmed pronounced growth suppression, with *E. coli* showing the greatest reduction in turbidity over 24 h. The antibacterial activity was attributed to sustained Cu^2+^ ion release, which reached 1.6 ppm by day 10 and correlated with the inhibition trend. These results demonstrate that CuO incorporation effectively transforms PLGA NFs into potent antimicrobial scaffolds suitable for wound care applications [453].

Cobalt-based fibers have also demonstrated strong antimicrobial performance, as reported for cobalt-MOF/PVA-PVP-based NFs [454]. These MOF-loaded fibers inhibited all seven tested pathogens, with MIC values ranging from 8 to 64 μg/mL, including *Salmonella enterica* (8 μg/mL), *Mycobacterium tuberculosis* (16 μg/mL), and *E. coli* (32 μg/mL). Their bactericidal activity was similarly strong, with MBC values as low as 16 μg/mL for *S. enterica* and *P. aeruginosa*. The NFs achieved complete bacterial clearance against *E. coli*, outperforming both pristine PVA-PVP fibers and amikacin, which was ineffective against *Legionella* and *Giardia*. This broad-spectrum efficacy was attributed to the high specific surface area (2415 m^2^/g), which enhances microbial interaction with cobalt MOF active sites [454]. Titanium-based NFs, particularly electrospun PVP/TiO_2_ systems, have also shown strong antibacterial performance [455]. Across all tested concentrations (0.5, 0.75, and 1.0 mg/mL), TiO_2_ NFs inhibited both Gram-positive and Gram-negative strains, including *E. coli*, *Pseudomonas aeruginosa*, *Salmonella typhimurium*, *Bacillus subtilis*, and *Staphylococcus aureus*. The highest activity was observed at 1.0 mg/mL, where *S. aureus* exhibited an inhibition zone of 21 mm, outperforming both TiO_2_ NPs and PVP-only controls, which showed no inhibition. This enhanced efficacy is attributed to the higher surface area of TiO_2_ NFs, which promotes stronger bacterial membrane disruption and more efficient interaction with bacterial cell walls [455].

In another study, functionalized electrospun polyamide NFMs were developed as antibacterial microfiltration media, achieving high pathogen removal in water treatment applications [456]. Among the tested biocides, poly[(dimethylimino)(2-hydroxy-1,3-propanediyl) chloride] (WSCP) at 5 wt% showed the strongest performance, removing 5.2 log_10_ colony forming units (CFU)/100 mL of hospital wastewater in short-term tests. Long-term filtration maintained high disinfection efficiency, achieving 5.6 log_10_ removal for *Staphylococcus aureus* and 4.0 log_10_ for *Escherichia coli*, demonstrating greater efficacy against Gram-positive bacteria. WSCP-functionalized NFs consistently outperformed silver, bronopol, 2,2-dibromo-3-nitrilopropionamide (DBNPA), and 2-(thiocyanomethylthio)benzothiazole (TCMTB). Although approximately 10% of WSCP was leached during operation, the membranes retained over 90% of the active agent and maintained full antibacterial performance [456]. In a related study, Nageh et al. prepared ZnO-loaded polyvinylidenefluoride (PVDF) NFs that exhibited strong antiviral activity, transforming otherwise inert PVDF into a functional antiviral platform [457]. Increasing ZnO content from 2.0% to 5.0% significantly enhanced activity; at 5% loading, ZnO/PVDF NFs completely suppressed human adenovirus type-5 (ADV-5), with viral copies reduced to undetectable levels as quantified by real-time polymerase chain reaction (RT-qPCR) in both adsorption and virucidal assays. Pristine PVDF showed no antiviral activity, while 2% ZnO/PVDF was effective only in the virucidal mode. All nanofiber formulations remained non-toxic to Vero cells (70-80% viability), confirming that antiviral effects originated from ZnO rather than cytotoxicity. The activity was attributed to Zn^2+^-mediated viral neutralization, membrane interactions, and oxidative stress mechanisms [457].

Boron nitride (BN) has also been explored in antimicrobial NFs, as demonstrated by Doğan et al., who incorporated hydroxylated BN (mBN) into PVA/PAA electrospun mats [458]. While pristine PVA/PAA exhibited limited antibacterial activity, mBN addition significantly improved bacterial reduction, achieving 68.8% inhibition against *E. coli* and 75.1% against *S. aureus* after 24 h of dynamic contact. Antibacterial activity increased with BN loading, with Gram-positive *S. aureus* showing higher susceptibility than *E. coli*. This difference is attributed to variations in cell wall structure, hydrophilicity, and aggregation behavior. The antimicrobial mechanism is associated with BN-induced oxidative stress, membrane disruption, and electrostatic interactions with bacterial cell walls [459], [460].

LBL micellar coatings provide a modular strategy for antibiotic loading and controlled release from fibrous scaffolds [461]. For example; BCM/TA coatings on PCL/Coll NFs were evaluated against *S. aureus* using zone-of-inhibition (ZOI) assays as shown in Fig. 42 [413]. Before testing, both bare and BCM/TA-coated fibers were UV-sterilized and loaded with clindamycin hydrochloride (1.0 mg/mL, pH 7.5) at 4 °C for 20 h. The MIC of clindamycin was 0.25 μg/mL, while BCM/TA-coated fibers produced larger ZOIs than bare fibers (32 ± 6 mm vs. 23 ± 9 mm) and absorbed approximately ten times more antibiotic by mass. Notably, clindamycin-loaded BCM/TA coatings retained antibacterial activity after 20 weeks of storage at 5 °C, with ZOIs comparable to those of freshly prepared samples. In liquid culture, these fibers reduced bacterial CFU by approximately two orders of magnitude within 5 h, similar to free clindamycin [413].

**Fig. 42:**
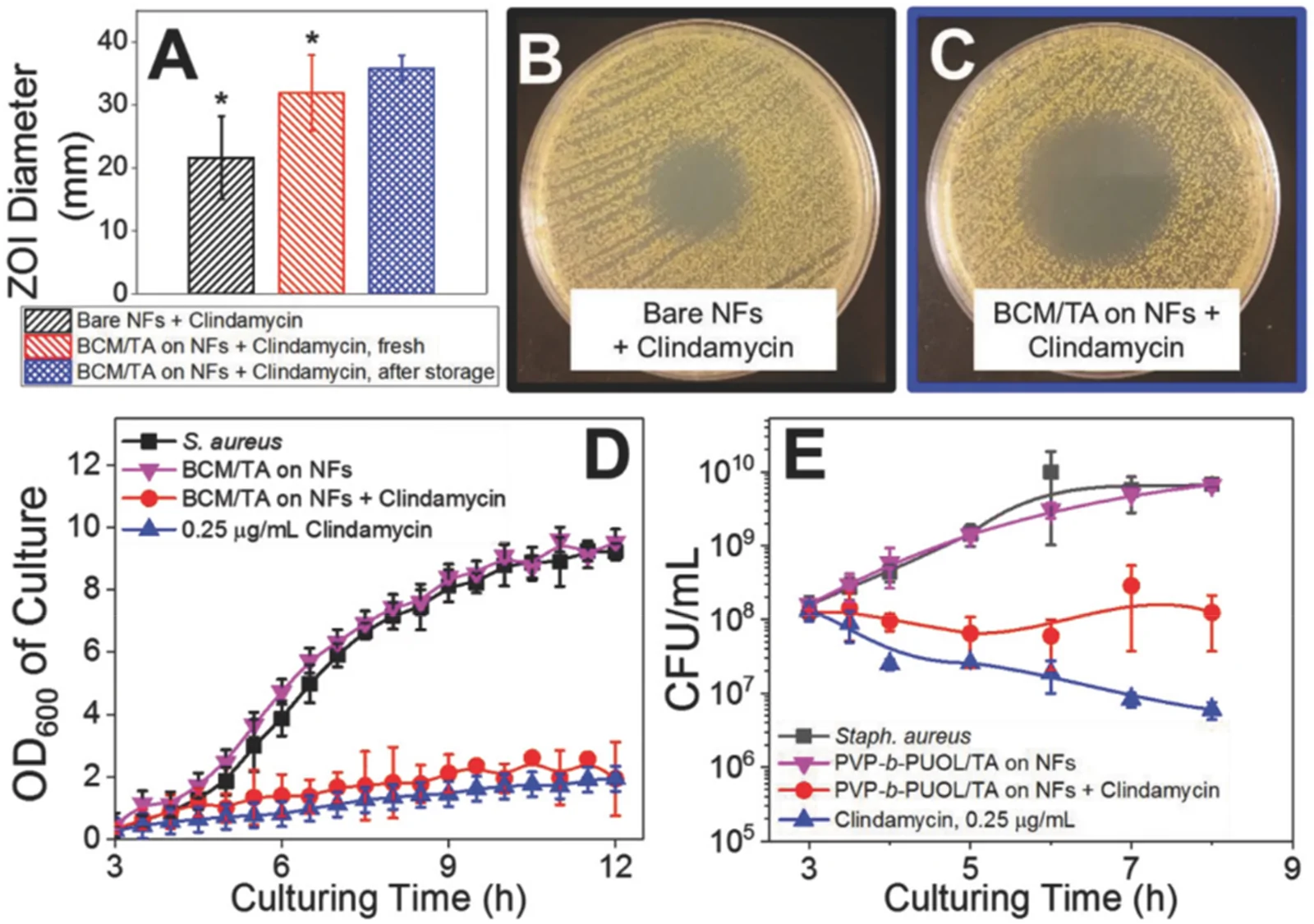
BCM/TA coatings on PCL/Coll NFs inhibit *S. aureus* growth in tryptic soy broth (TSB) solution and on agar plates. (B, C) Images of ZOIs for bare and BCM/TA-coated NFs loaded with clindamycin, including samples freshly prepared (red) and stored at 5 °C for 20 weeks (blue). (D, E) Clindamycin-loaded BCM/TA coatings markedly reduce bacterial culture optical density and colony-forming units over time [413]. Copyright 2018. Reproduced with permission from John Wiley & Sons.

Antimicrobial screening is essential in diabetic wounds because patients with diabetes are at increased risk of infection. Wang et al. [430] demonstrated that COR/OHDA/GEL NFMs provide strong antimicrobial activity against both *E. coli* and *S. aureus*. In vitro assays showed that COR-loaded NFs achieved 71.2% inhibition of *E. coli* and 95.6% inhibition of *S. aureus*, outperforming the OHDA/GEL control membranes. As illustrated in Fig. 43A and 43B, bacterial colonies were markedly reduced around COR/OHDA/GEL samples, confirming their enhanced bacteriostatic effect. The differential activity between the two species is attributed to Gram-positive bacteria's greater sensitivity to COR. Overall, these findings indicate that COR/OHDA/GEL NFs can effectively suppress early-stage infection in diabetic wounds, creating a cleaner microenvironment that supports subsequent healing [430]. Puigmal et al. evaluated fucoidan (FUC)-loaded PVA/CS NFs as antibacterial scaffolds for skin tissue applications and demonstrated that fucoidan significantly enhanced antimicrobial performance [462]. While PVA and PVA/CS control mats produced only modest inhibition zones (7-8 mm), the incorporation of fucoidan increased antimicrobial effect against *E. coli* and *S. aureus*, with the 30 mg FUC formulation reaching up to 10 mm for *E. coli* and 9 mm for *S. aureus*. The inhibition areas increased progressively with higher fucoidan loading, confirming a dose-dependent effect. This improvement was attributed to the underlying antimicrobial activity of fucoidan, which synergizes with CS to disrupt bacterial membranes and inhibit growth. Overall, fucoidan-enriched NFs exhibited the strongest antibacterial response among all tested formulations, highlighting their suitability for infection-prone wound environments [462]. Another approach involved the development of chitosan-thiourea/PVA (CST/PVA) NFs incorporating Ag NPs and coated with polyhexamethylene biguanidine (Ag/PHMBG), leading to a highly effective antibacterial wound-healing scaffold. The optimized formulation containing 3.0% Ag/PHMBG (PCT3) achieved 95% inhibition of *S. aureus* and *P. aeruginosa* within three days, significantly outperforming CST/PVA controls. As illustrated in Fig. 44, time-kill assays showed a sharp and sustained reduction in bacterial optical density over 72 h, confirming continuous bactericidal activity [463]. This strong antimicrobial effect is attributed to the synergistic action of thiourea-modified CS, which enhances electrostatic interactions with bacterial membranes, and PHMBG-stabilized Ag NPs, which provide controlled silver ion release [463].

**Fig. 43:**
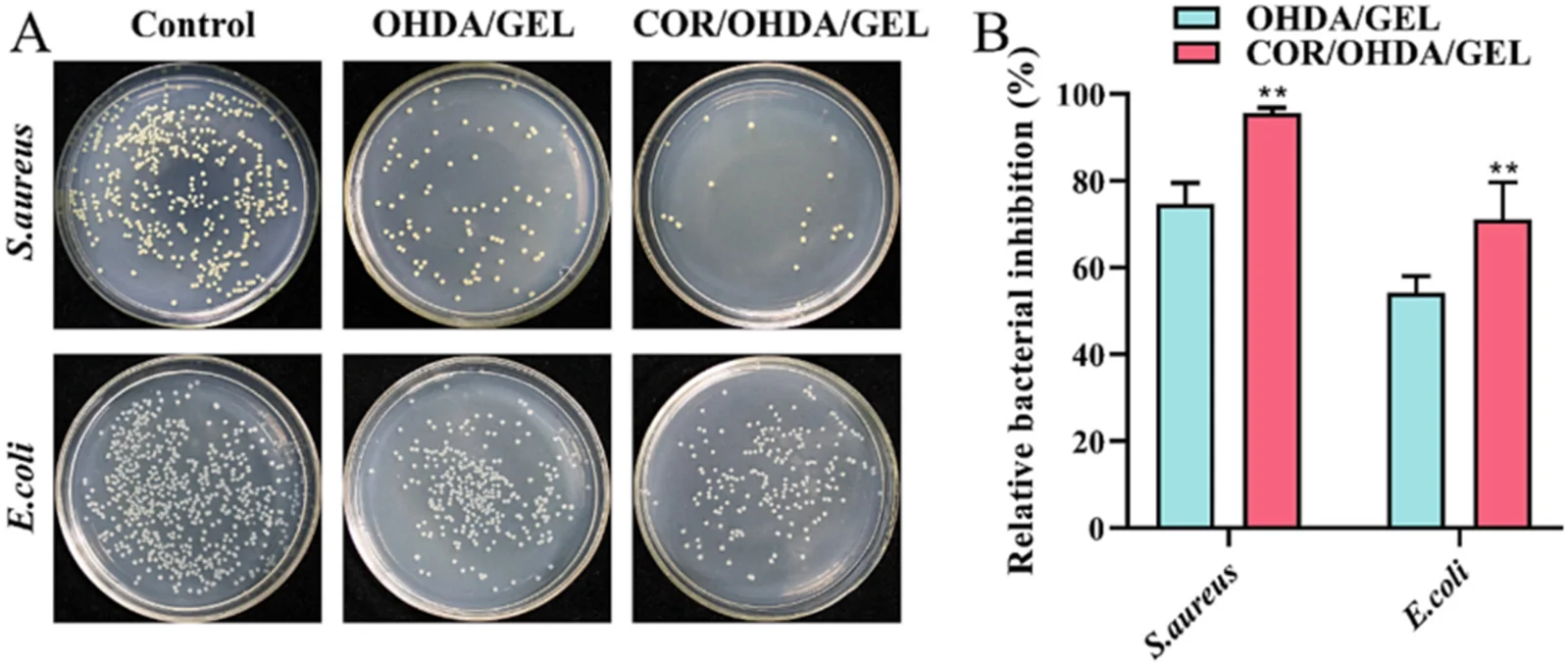
(A) OHDA/GEL nanofiber membrane and COR/OHDA/GEL nanofiber membrane photos (B) relative bacterial inhibition of *S. aureus* and *E. coli*[430]. Copyright 2024. Reproduced with permission from Elsevier.

**Fig. 44:**
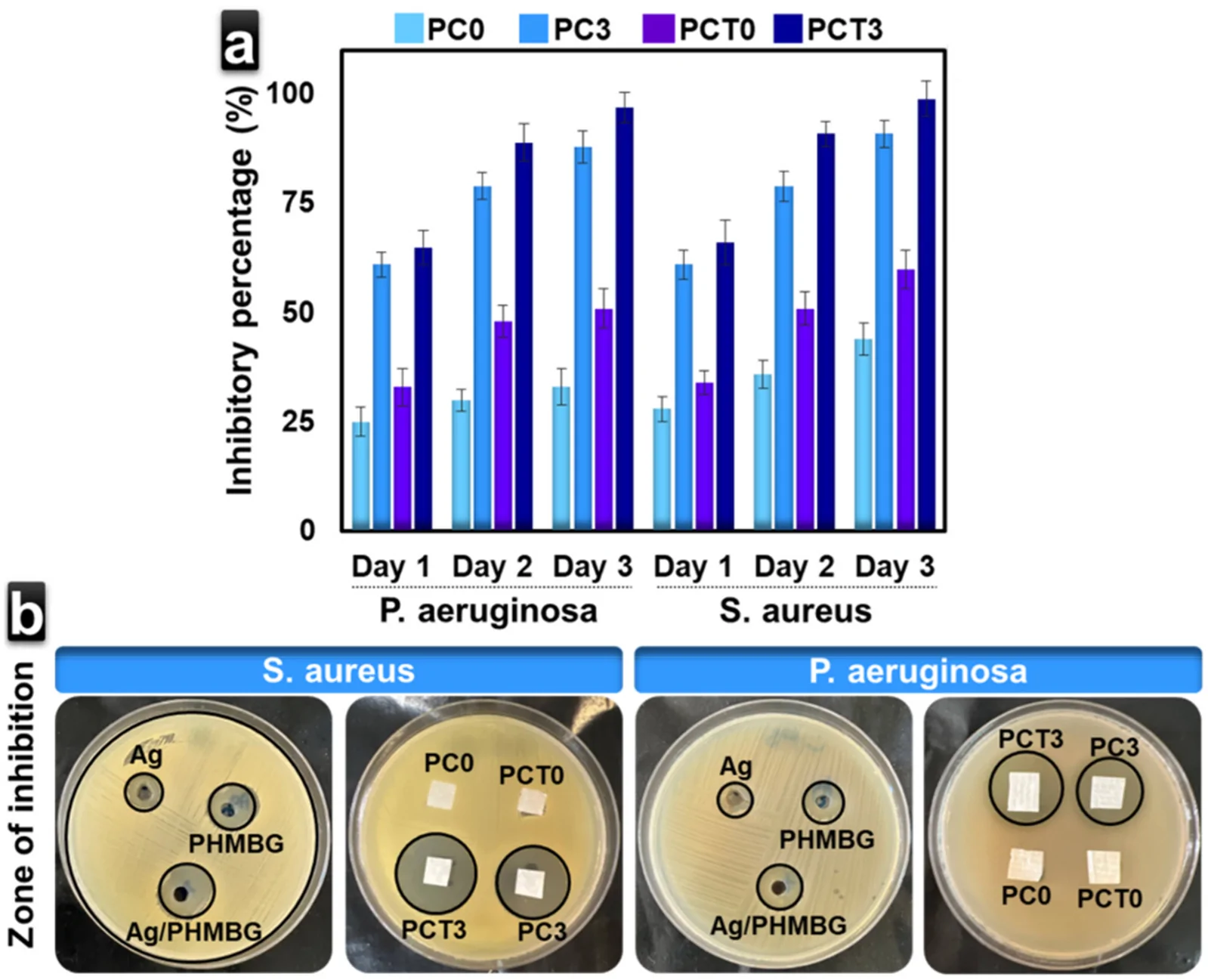
(a) Time-management techniques, (b) zone inhibition techniques, and the antibacterial activity of various samples [463]. Copyright 2025. Reproduced with permission from Elsevier.

Building on these antibacterial nanofiber strategies, Soo et al. demonstrated that silver-doped titanium dioxide (Ag-TiO_2_) NFs can deliver exceptionally strong antibacterial activity, far surpassing that of intrinsic TiO_2_ [464]. Their optimized formulation containing 2.0% Ag achieved the highest bactericidal performance, achieving a 5.92 log CFU reduction against *Salmonella Albany* and a 1.38 Log CFU reduction against *S. aureus* after only 60 min of contact. Ag-TiO_2_ consistently outperformed pure TiO_2_ across multiple Gram-negative strains, with log reductions exceeding 4-5 logs for several *E. coli* pathotypes. The study attributed this enhancement to the synergistic interaction between Ag NPs and photocatalytic TiO_2_, which increases ROS generation and suppresses electron-hole recombination, thereby intensifying membrane damage and bacterial inactivation [464]. Extending this antimicrobial nanofiber framework, CA membranes loaded with metal-doped silica nanohybrids were developed and exhibited exceptionally broad-spectrum antimicrobial activity [465]. Among all formulations, the trimetallic Ag-Si + Cu-Si + Co-Si nanohybrid membrane produced the largest inhibition zones, outperforming all monometallic counterparts against *S. aureus*, MRSA, *S. pneumoniae*, *K. pneumoniae*, *E. coli*, *P. aeruginosa*, and multiple fungal strains. The inhibition zones were consistently wider for the trimetallic system, confirming a strong synergistic effect. This enhanced activity was attributed to visible-light-activated ROS generation, facilitated by the lowest band gap energy, along with the combined bactericidal effects of Ag, Cu, and Co [465]. Overall, ES combined with diverse biocidal agents provides a highly flexible platform for antimicrobial NFs. In addition to the studies discussed above, many other reports in the literature have explored similar systems, some of which are listed in Table 4.

**Table 4:** Representative electrospun antibacterial NFs and incorporation strategies.

Electrospun polymer	Antibacterial Agent (s)	Incorporation method	Ref.
PLGA	Cefoxitin	Mixing (blend)	[256]
PLA, PEVA, PLA/PCL, PEUU/PLGA	Tetracycline	Mixing (blend)	[466]
coPLA, coPLA/PEG, PU	Ciprofloxacin	Mixing (blend)	[467]
PLAGA	Cefazolin	Mixing (blend)	[468]
PMMA/nylon	Ampicillin	Core-sheath (coaxial)	[469]
CA	Chlorhexidine	Mixing (blend)	[470]
PAN, PLA, PLA/PEG	QACs	Mixing (blend)	[471]
PLA, PCL, PAN, PVA, PEO	Ag NPs	In situ synthesis	[472]
PVA/CS	Ag NPs	NP dispersion	[473]
PU, PVA, SF	ZnO, TiO_2_	NP dispersion	[474]
PLA, PVA	CS derivatives	Blending	[475]
PET, PCL, PEO	CS	Blending	[476]

### 4.5 Cancer diagnostics and therapy

Compared to a uniform film, an ESNF network offers a dramatically higher surface-to-volume ratio, allowing extensive interaction with solutes and tumor cells and thereby improving drug loading and interaction efficiency [477]. Drugs can be embedded within or onto NFs for localized, sustained delivery, allowing chemotherapeutic agents to act directly at the tumor site while minimizing general toxicity [478]. This spatial control over drug release is particularly beneficial in solid tumors and in sensitive regions such as the brain. ESNFs fabricated from synthetic, natural, or hybrid polymers have therefore been widely applied in cancer therapy, 3D tumor modeling, and diagnostics. For instance, they serve as delivery vehicles for small molecules, genes, or stimuli-responsive agents in tumor-targeted therapy [479] and as scaffolds to recreate tumor microenvironments for high-throughput drug screening and disease modeling [480]. Nanofiber meshes have also been functionalized for cancer diagnostics, including the detection and identification of circulating tumor cells (CTCs) and the recognition of tumor biomarkers, thereby playing a key role in cancer research and diagnosis as tools for therapeutic applications [481], [482].

Zhang et al. examined how fibrillar ECM cues regulate cell migration by patterning polystyrene fibers into linear, curved, looped, and grid-like arrays (3 μm diameter) using continuous ES with reduced power [483]. Cells adjusted their shapes and migration routes according to local microfibrillar geometries; however, the global pattern had little effect on mean migration speed. A behavioral shift emerged when feature dimensions approached the cell's minor-axis limit, suggesting that the short axis of the cell body constrains rerouting along a given fiber track [483]. Estabridis et al. cultured U251 glioblastoma cells on suspended NFs (approximately 400 nm) to compare 1D and 2D migration. Cells adhered to single, dual, or orthogonal fibers; migration was faster on parallel fibers than on single fibers, and 1D guidance resulted in more persistent and rapid movement than 2D surfaces [484]. In another study extending capture-based approaches, Ma et al. reported a 3D fibrous network that demonstrated a “trap” effect for the efficient isolation and capture of cancer cells [485]. Compared with an anti-epithelial-cell adhesion molecule (EpCAM)-functionalized smooth polystyrene control that captured fewer than 40% of MCF-7 cells, the 3D micro- and nanofibrous interfaces captured 67-82% of MCF-7 cells within 30 min. They selectively enriched EpCAM-positive cell lines (MCF-7, PC3) over EpCAM-negative lines (Daudi, Jurkat, HeLa), attributed to the elastic 3D network topology [485].

Drug-loaded nanocarriers such as mesoporous silica NPs, carbon nanotubes (CNTs), and polymeric micelles have been co-electrospun into fibers to enhance local therapy, improve retention, enhance tumor accumulation, and sustain drug release [486], [487], [488], [489]. Local recurrence following cancer surgery is still an important clinical issue. To enhance antitumor efficacy while maintaining acceptable toxicity for the prevention of local recurrence, Zhang et al. [490] demonstrated a dual-drug-loaded multilayered fiber mat approach, in which dichloroacetic acid (DCA) and oxaliplatin were co-spun into specific layers of the fabricated scaffold. The oxaliplatin-loaded fiber layer was sandwiched between a base film layer and two outer fiber layers, yielding multilayered mats with dual-release capability and synergistic anticancer activity. DCA, with its relatively low toxicity, promoted apoptosis in cancer cells through metabolic modulation, whereas oxaliplatin, at low concentrations, eliminated remaining cells. When applied at the resection margin in a murine cervical carcinoma model, these dual-drug multilayered mats demonstrated superior anti-recurrence efficacy and reduced systemic toxicity over 30 days compared with single-layer counterparts. Such time-programmed DD highlights the potential of multilayered nanofiber scaffolds for localized post-surgical cancer treatment [490].

Another investigation reported a novel strategy for developing simultaneously photo-cleavable and activatable (SPCA) prodrug-backboned block copolymer micelles using a Pt(IV)-azide complex. These Pt(IV)-azide complexes functioned as anticancer prodrugs, stimuli-responsive groups, and co-monomers, enabling the construction of a new class of macromolecular prodrugs and drug carriers for mono- or multi-drug cancer therapy [491]. Building on these findings, Zhang et al. exhibited an implantable nanofiber system loaded with a prodrug micelle/small-molecule for improved localized therapy [492]. In this approach, amphiphilic Pt(IV) prodrug-backbone micelles ((PEG_2_k-Pt(IV))_n_) were synthesized via copper-free click chemistry and co-electrospun with DCA into PVA NFs to achieve localized combination therapy, as illustrated in Fig. 45 [492]. In U14 tumor-bearing mice, the combination micelle/DCA fiber system suppressed tumor growth more effectively than single-agent fiber systems. Also, it outperformed systemic (intravenous) administration of micelles plus DCA, particularly beyond day 7, consistent with sustained high local drug exposure and gradual cellular uptake of micelles. Body weight increased steadily in all fiber-treated groups, whereas intravenous treatment initially decreased, followed by recovery. Survival was improved relative to controls, and histological analysis revealed extensive tumor necrosis with pyknotic or absent nuclei in the combination-fiber group (Fig. 46) [492]. Furthermore, histological assessment revealed that all major organs, including the heart, liver, spleen, lungs, and kidneys, remained healthy without visible inflammation or lesions after treatment, as shown in Fig. 47A [492]. The excellent biocompatibility of PVA has been demonstrated in previous research. Although PVA is not readily degradable in vivo, the fibrous matrix can gradually dissolve in body fluids and subsequently removed. At the same time, systemic toxicity was markedly decreased by local fiber delivery. The potential adverse effects in mice were further evaluated using biochemical blood markers estimation. The results shown in Fig. 47B indicated that kidney and liver functions remained normal compared with the control group. These findings suggest that the M/DCA-fiber system may serve as an implantable tool for effective therapy of advanced tumors while reducing possible adverse effects [492].

**Fig. 45:**
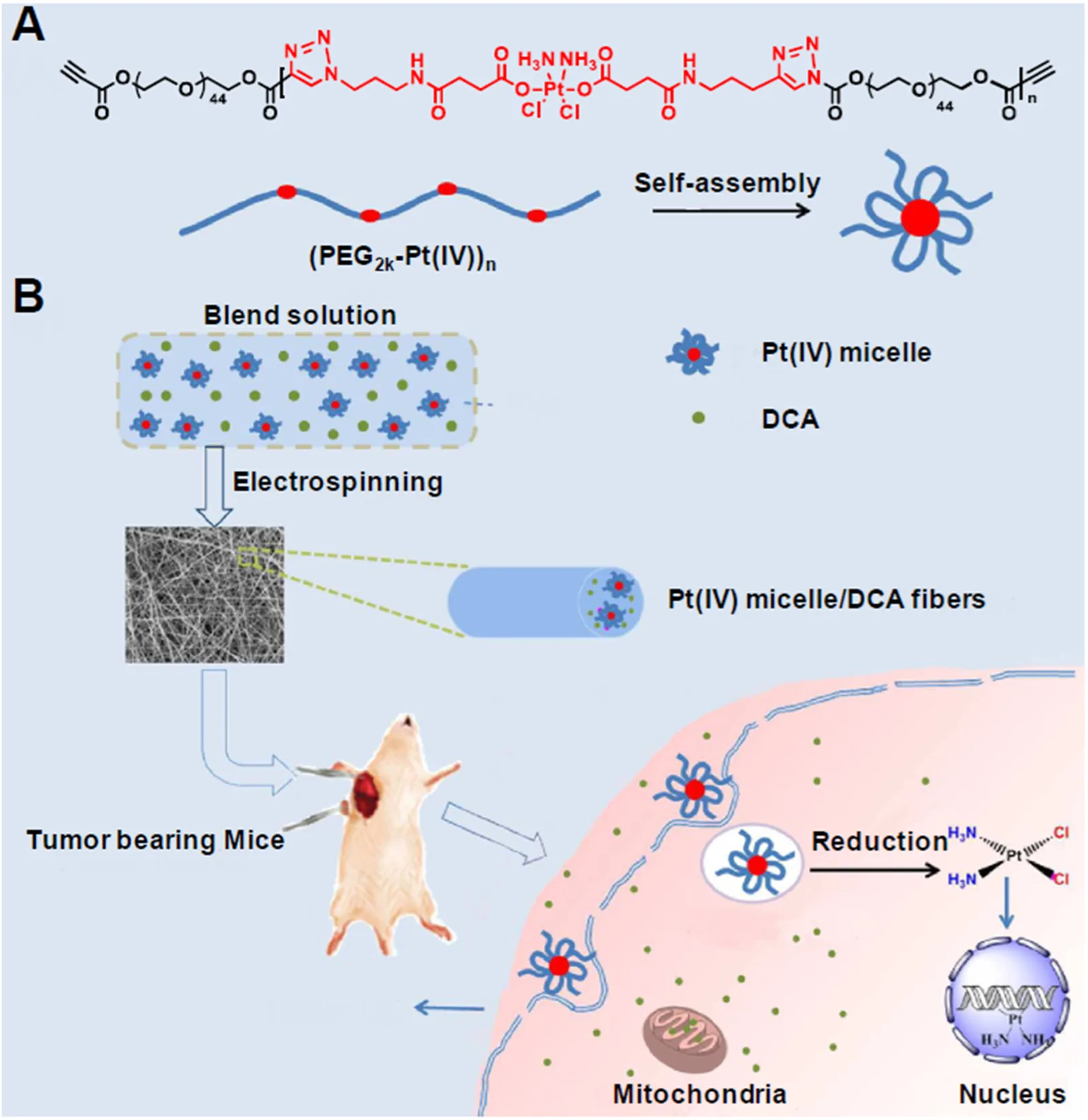
Schematic illustration of the Pt 9Iv) prodrug-backbone micelles (A) structure and self-assembly process and (B) fabrication of m/DCA-fibers for local combination therapy [492]. Copyright 2017. Reproduced with permission from the Royal Society of Chemistry.

**Fig. 46:**
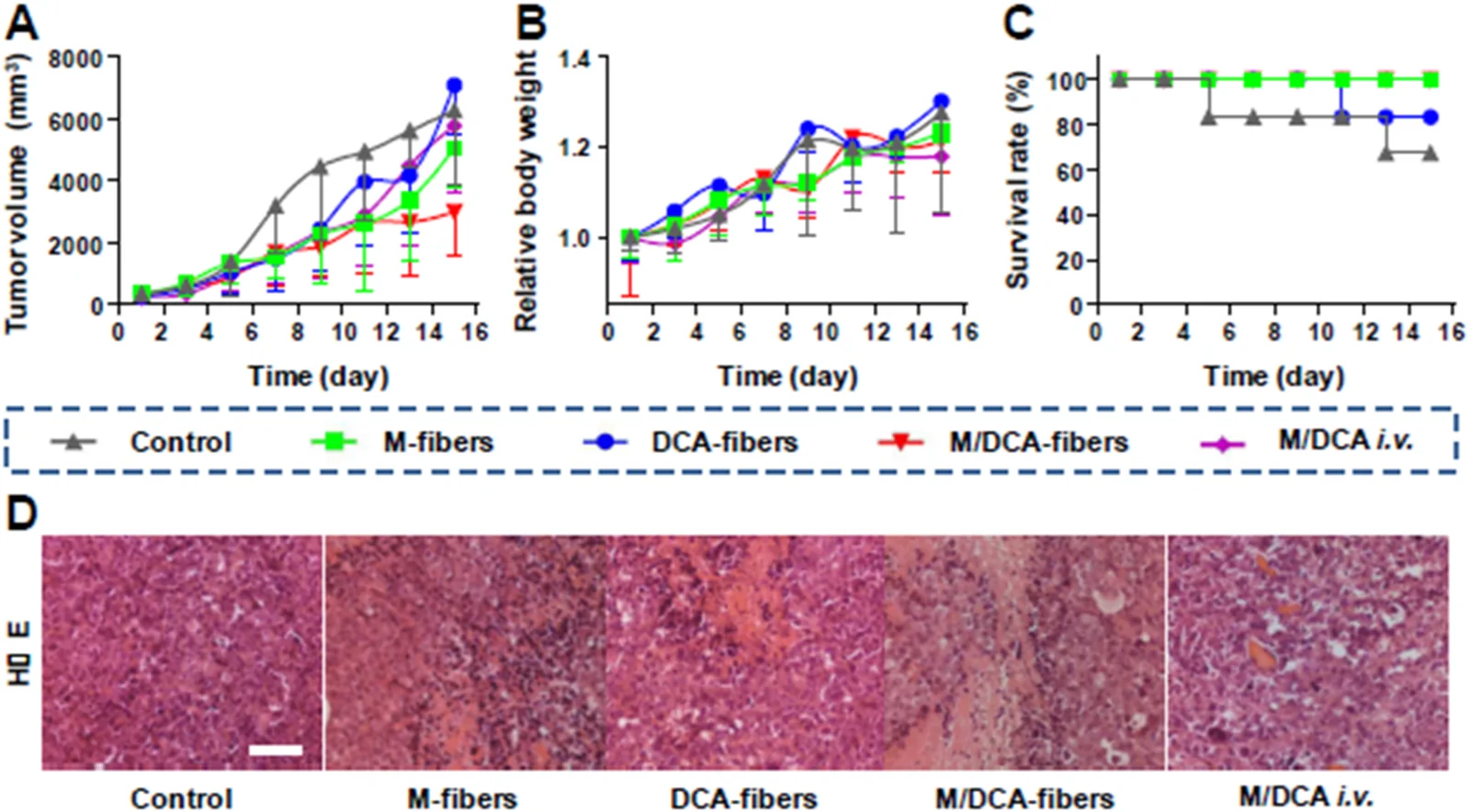
U14 tumor-bearing mices (A) tumor volume and (B) post-treatment change in relative body weight. (C) Tumor-bearing mices survival rate following various treatments, and (D) Histologic evaluation of tumors using H&E staining in mice. Scale bar = 50 μm [492]. Copyright 2017. Reproduced with permission from The Royal Society of Chemistry.

**Fig. 47:**
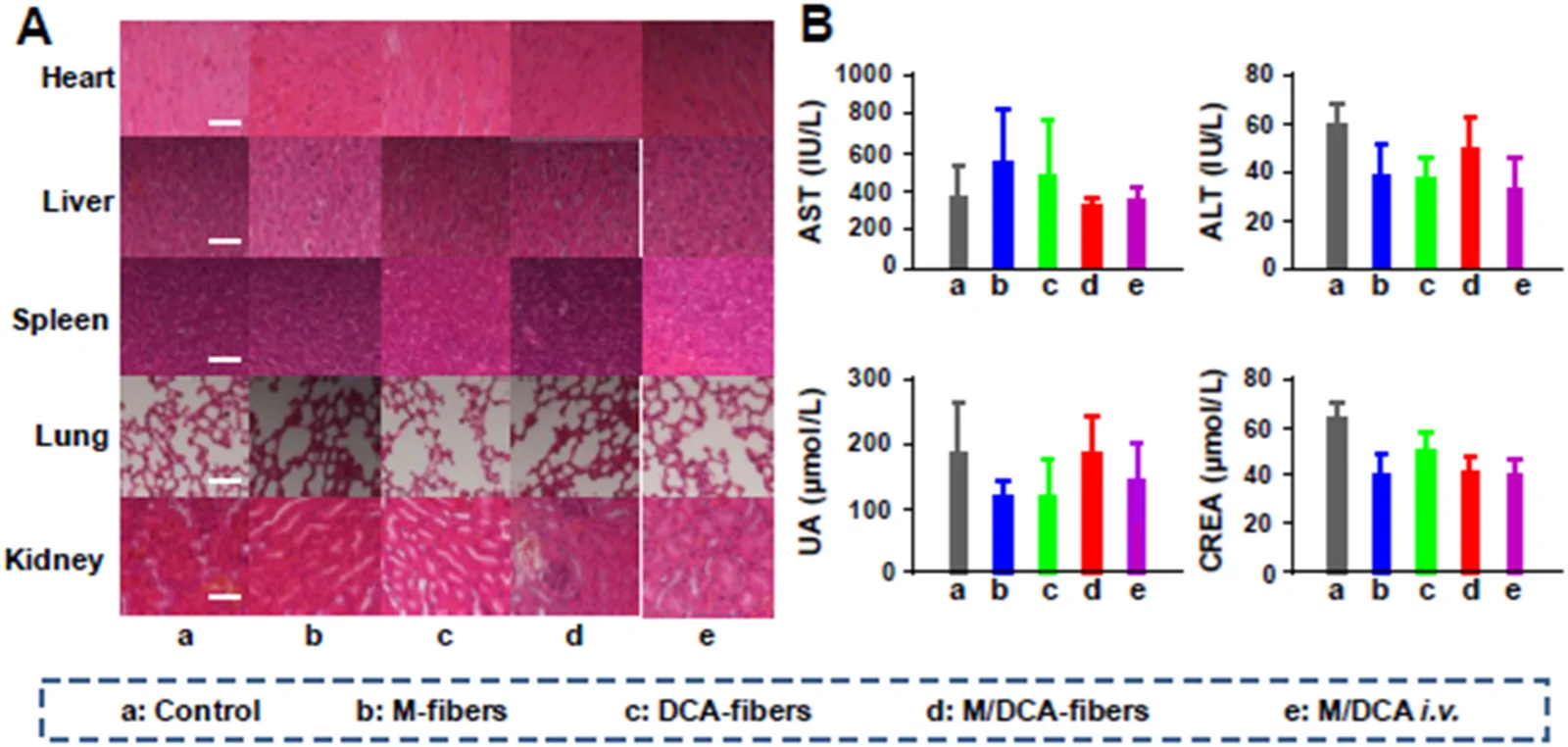
Systemic toxicity evaluation of nanofiber materials on mice. (A) H&E histologic images of various organs. (B) blood markers assessment after 15 days treatment. Scale bar = 50 μm [492]. Copyright 2017. Reproduced with permission from The Royal Society of Chemistry.

NFs can also enable selective detention and slow release of CTCs. For example, Wang et al. grafted thermoresponsive poly(N-isopropylacrylamide) (PNIPAAm) brushes onto CS NFs by ATRP and later introduced a poly (methacrylic acid) linker to anchor a DNA aptamer-based capture agent [493]. At low temperature, the PNIPAAm chains extended, rendering the surface hydrophilic and facilitating the release of non-target white blood cells (WBCs), thereby increasing purity. In spiked human whole blood (10-1000 MCF-7 cells/mL), the aptamer-PNIPAAm-modified CS fibers efficiently captured cancer cells while minimizing WBC carryover [493]. In another study, SF/gelatin (SF/GL) NFs functionalized with Pd and Pt NPs exhibited cytotoxic effects against A549 lung cancer cells, with IC_50_ values of 32.8 μg/mL (SF/GL), 30.3 μg/mL (SF/GL/Pd), 30.2 μg/mL (SF/GL/Pt), and 28.8 μg/mL (SF/GL/Pd-Pt), with the bimetallic system showing the strongest activity [494]. The proposed mechanism involves close contact between NFs and cells, followed by gradual ion release and ROS generation, resulting in damage to cellular membranes and nucleic acids, in agreement with previous reports [495], [496], [497]. Additionally, quantitative real-time PCR (qRT-PCR) analysis showed downregulation of Hsp90 after SF/GL/Pd-Pt exposure (−0.70 at 100 μg/mL), consistent with the literature linking reduced Hsp90 expression to favorable cancer prognosis [494], [498].

Magnetic hyperthermia can sensitize tumors to chemotherapy [499]; however, free MNPs raise safety concerns in vivo, including DNA damage and mitochondrial dysfunction [500], [501]. Embedding MNPs within NFs provides a more controllable and implantable platform while preserving their nanoscale functionality. For example, Kim et al. [502] demonstrated a smart hyperthermia nanofiber system that integrates both heat generation and controlled drug release. In this design, MNPs and DOX were incorporated into a chemically crosslinked, thermoresponsive poly (N-isopropylacrylamide-co-N-hydroxymethyl acrylamide) [poly(NIPAAm-co-HMAAm)] nanofiber matrix. When an alternating magnetic field (AMF) is applied (“on” state), the MNPs generate localized heat, inducing collapse of the polymer network and triggering an on-demand burst release of DOX. When the AMF is turned off, the system returns to its swollen state, thereby suppressing further drug release. This mechanism was further validated in vitro, where human melanoma (COLO 679) cells exhibited minimal cytotoxicity in the absence of AMF, confirming stable drug retention within the fibers [502]. Upon AMF exposure, a pronounced decrease in cell viability was observed. MNP-only fibers reduced viability by approximately 20% via hyperthermia alone, whereas DOX/MNP-loaded fibers achieved a greater reduction of about 30%, demonstrating a synergistic effect between thermal ablation and chemotherapeutic release [502]. Overall, ESNFs offer multiple convergent advantages in oncology: ECM-mimetic guidance for migration studies and CTC capture, high-capacity localized drug depots enabling synergistic combination therapies and prodrug systems, and implantable hyperthermia platforms that couple thermal effects with stimulus-triggered drug release. Moving forward, improved in vivo pharmacokinetic characterization at tumor margins, standardized reporting of drug loading and release parameters, and harmonized cytotoxicity assessment frameworks will be crucial to hasten the clinical translation of these nanofiber-based therapeutic strategies [502].

Another study reported that DOX released from DOX/MNP-loaded NFs induces apoptosis through synergy with magnetic hyperthermia. In contrast, short exposure to an AMF produced only a mild anticancer effect when hyperthermia was applied alone [503]. Notably, although the cumulative amount of DOX released from DOX/MNP-NFs was comparable to that of free DOX, the combined modality resulted in higher cell death than chemotherapy alone. In contrast, DOX/MNP-NFs without AMF exposure exhibited no intrinsic cytotoxicity. These findings support a trigger-on-demand mechanism that is superior to conventional systemic drug administration [503]. In other studies, apoptosis staging was confirmed using annexin V (early apoptosis) and terminal deoxynucleotidyl transferase-mediated dUTP nick end labelling (TUNEL) (late apoptosis) assays [502], [504], [505]. MNP-only NFs showed annexin V positivity without TUNEL staining, consistent with membrane perturbation as the primary effect under brief AMF exposure. In contrast, DOX/MNP-NFs and free DOX induced both annexin V and TUNEL signals by day 5 (but not day 4), indicating progression to late-stage apoptosis [502].

Beyond therapeutic applications, nanofiber-enabled CTC capture systems have demonstrated strong translational potential. For example, a microfluidic platform containing an electrospun interface functionalized with a melanoma-specific anti-CD146 coating was developed for the efficient capturing and separation of circulating melanoma cells (CMCs) [506]. In this study, a PLGA nanofiber-containing NanoVelcro chip (Fig. 48a and b) was designed to achieve high-efficiency CMC capture while allowing highly precise isolation of single CMCs immobilized on the nanosubstrate without contamination from WBCs. A custom-built ES system was used to deposit PLGA NFs with a thickness of 130.6 ± 62.7 nm (Fig. 48c) over a laser microdissection (LMD) slide, thereby generating transparent PN-NanoVelcro substrates. These substrates were subsequently functionalized with streptavidin-conjugated melanoma-specific anti-CD146 antibodies. To facilitate covalent streptavidin attachment, N-hydroxysuccinimide (NHS) chemistry was employed to activate the -COOH groups present on the PLGA nanofiber surface (Fig. 48d). The PN-NanoVelcro chip consisted of a transparent PN-NanoVelcro substrate overlapped with a polydimethylsiloxane (PDMS) chaotic mixer (Fig. 48e), both assembled within a plastic container and secured with four screws. A 22 cm-long microchannel with herringbone microfeatures was fabricated within the PDMS mixer to enhance cell-substrate interactions by increasing the frequency of cell-capture surface contact. Importantly, integrating PLGA NFs with the LMD slide enabled precise single-cell isolation (Fig. 48f). For performance optimization and validation, PN-NanoVelcro chips were tested with suspensions of the M229 human melanoma cell line, prepared at 100 cells mL^−1^ in Dulbecco's Modified Eagle Medium (DMEM) which served as a model system. The effect of different ES deposition times on CMC capture efficiency was evaluated at a flow rate of 1 mL h^−1^, and the optimized deposition time was 180 min, yielding an approximate capture efficiency of 87% (Fig. 49a). Furthermore, the use of PLGA NFMs (3 μm) and application of a reduced flow rate of 0.5 mL h^−1^ further enhanced cell capture efficiency (Fig. 49b). The distribution of captured cells along the 22 cm channel was also investigated, revealing that approximately 68% were localized within the initial 8 cm, indicating that the explored length was sufficient for effective cell capture (Fig. 49c). To further evaluate device performance, four control experiments were conducted in parallel using PN-NanoVelcro chips without anti-CD146 coating, PN-NanoVelcro chips lacking herringbone microfeatures, anti-CD146-coated PLGA films without nanostructures, and anti-CD146-coated silicon nanowire (SiNW) substrates. The comparative results demonstrated that the capture antibody, chaotic microfluidic mixer, and nanostructured substrate each contributed significantly to the enhanced cell capture efficiency observed in the PN-NanoVelcro system (Fig. 49d). The dynamic detection range of the chips was subsequently evaluated using artificial CMC samples (DiO-stained M229 cells spiked into either healthy blood or DMEM medium) at concentrations ranging from 10 to 1000 cells mL^−1^. The data showed that the device possessed sufficient sensitivity and performance for clinically relevant samples, which typically contain only a few to several hundred CMCs per milliliter (Fig. 49e). Finally, the specificity and broader applicability of the developed chips for capturing CD146-positive melanoma cells were assessed using three cell lines known to express the antigen (M229, M229R, and M202), two CD146-negative cancer cell lines (Jurkat leukemia and PC3 prostate cancer cells), and freshly isolated human WBCs. The findings confirmed that the PN-NanoVelcro chips selectively captured melanoma cells with high specificity (Fig. 49f). In contrast, nonspecific binding of other cell types was effectively minimized through subsequent immunocytochemistry (ICC) and fluorescence-guided LMD analysis [506].

**Fig. 48:**
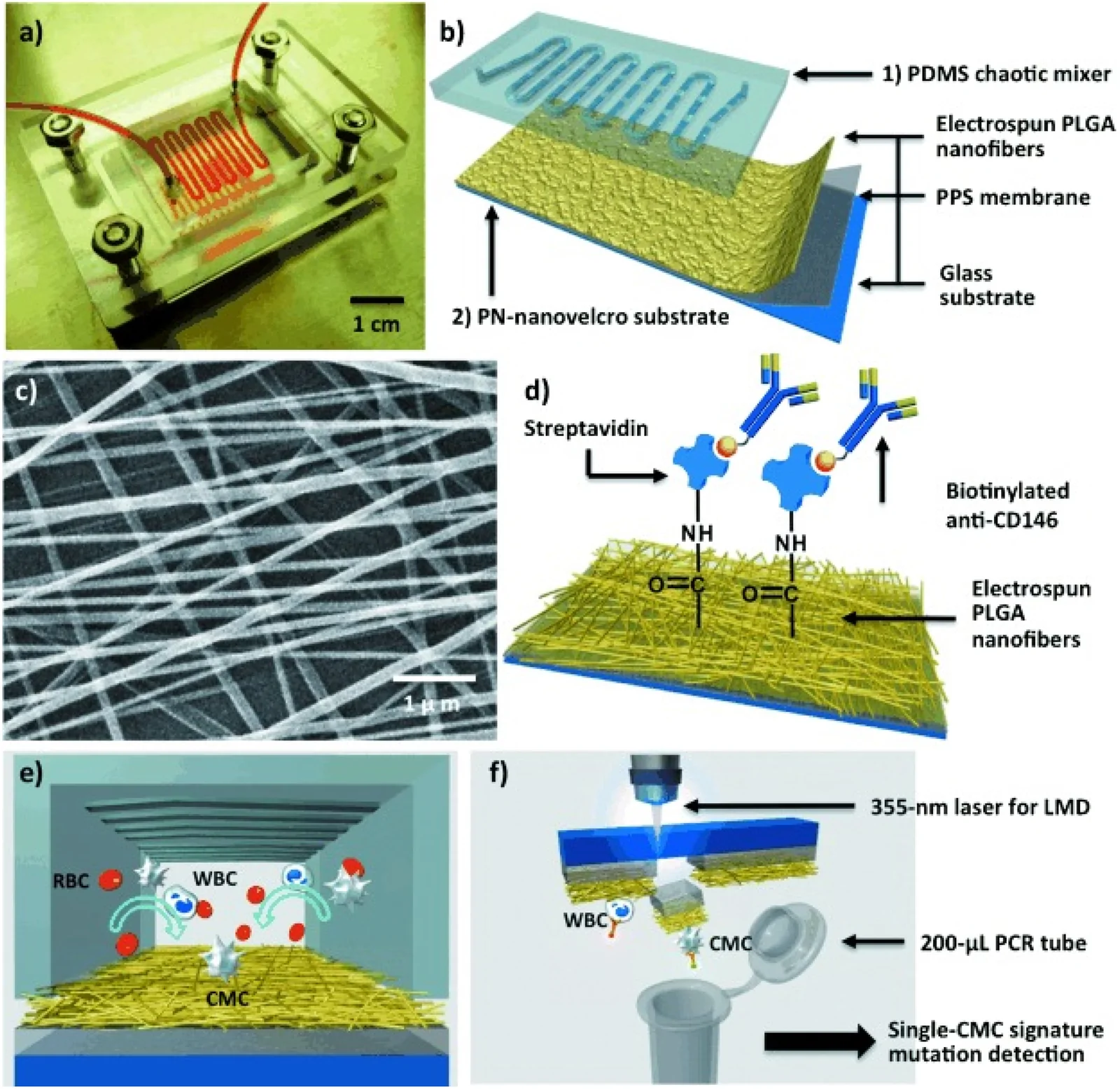
(a) Digital photo of the PN-NanoVelcro chip designed for capturing and isolating individual CMCs. (b) Illustration of the chip composition, showing (1) Coated PDMS chaotic mixer and (2) A transparent PN-NanoVelcro substrate. (c) SEM micrograph of PLGA ESNFs deposited on the PN-NanoVelcro substrate. (d) Surface functionalization strategy using NHS chemistry to covalently immobilize streptavidin for subsequent conjugation of biotinylated anti-CD146. (e) Pictogram showing the capture mechanism of the PN-NanoVelcro CMC chip. (f) LMD-based method for the isolation of individual CMCs [506]. Copyright 2018. Reproduced with permission from John Wiley & Sons.

**Fig. 49:**
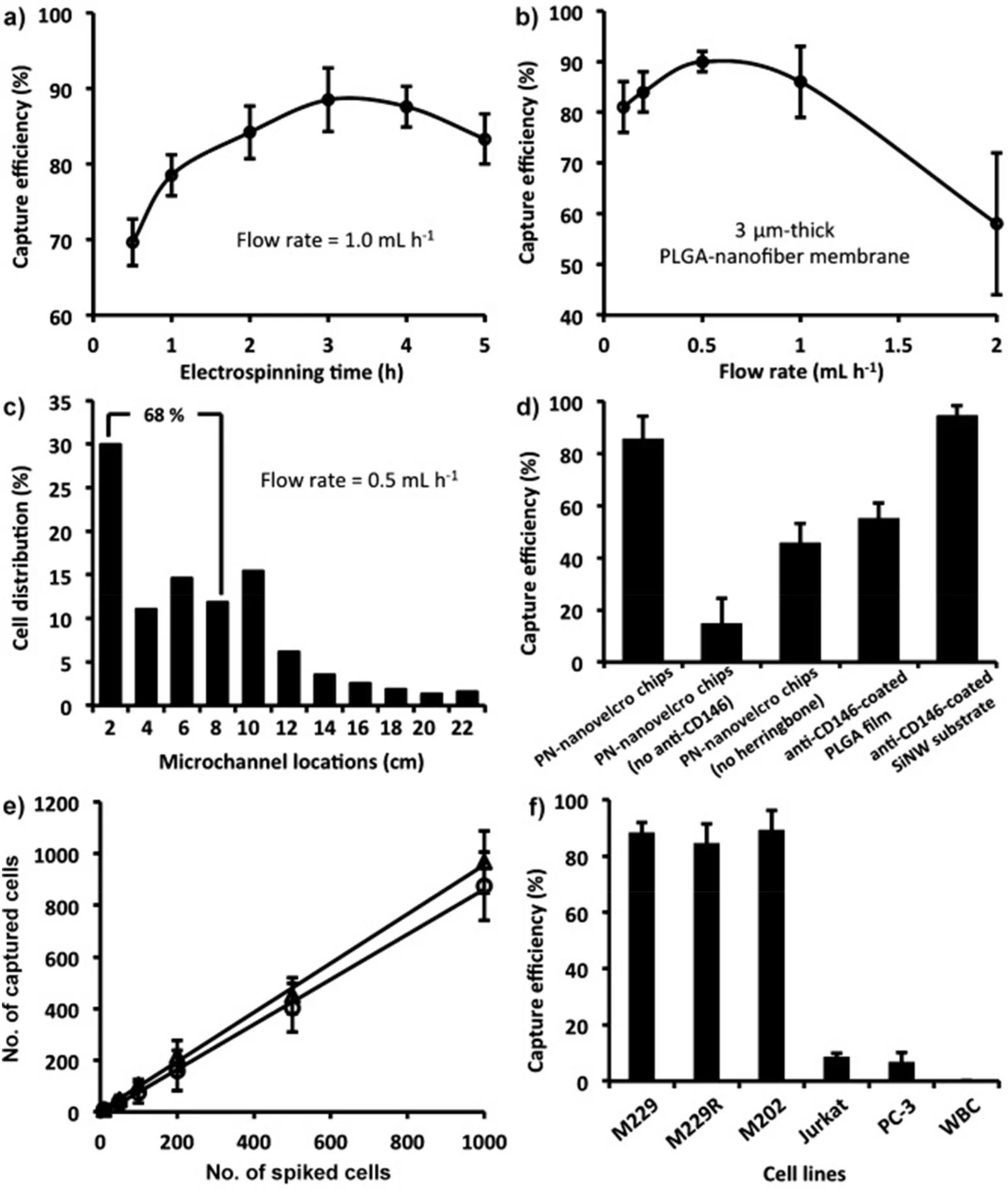
Optimization and validation of PN-NanoVelcro chips using M229 melanoma cells (error bars represent standard deviations, *n* ≥ 3). (a) Capture efficiency as a function of ES deposition time. (b) Capture efficiency measured at different flow rates using PN-NanoVelcro chips fabricated with the optimal ES time of 3 h. (c) Distribution of captured cells along the microchannel. (d) Assessment of cell capture efficiency between anti-CD146-functionalized chips and four control systems. (e) Capture efficacy as a function of cell number in DMEM medium (△) and whole blood (○). (f) Capture efficiency for different cell types, including melanoma cells (M229, M229R, and M202), T lymphocytes (Jurkat), prostate cancer cells (PC3), and WBCs [506]. Copyright 2018. Reproduced with permission from John Wiley & Sons.

Core-sheath and multilayer designs provide additional control over drug localization and leakage. For example, Yang et al. demonstrated an implantable active-targeting micelle-in-nanofiber system for effective and safe cancer therapy, as shown in Fig. 50 [507]. First, folate (FA)-conjugated PCL-PEG (FA-PCL-PEG) copolymers were used to incorporate DOX via self-assembly into active-targeting micelles (FM). Folate ligands can specifically bind to folic acid receptors, which are overexpressed on many solid tumors. These micelles were subsequently inserted into the core of core-sheath NFs via coaxial ES, in which the inner phase consisted of an aqueous PVA solution containing micelles and the outer phase consisted of a gelatin solution [507]. In vitro release studies showed that free FM exhibited a fast ∼60% release within the initial 12 h, followed by a cumulative ∼75% release at 48 h and ∼98% at 288 h. In contrast, FM-loaded NFs showed a reduced initial burst and a slower cumulative release, reaching ∼40% at 48 h and just over 80% at 288 h [507]. The faster release from free micelles was attributed to their limited thermodynamic stability in aqueous environments [508]. In vivo fluorescence imaging (4T1 tumor model) at 6, 24, and 48 h showed weak tumor signals in all groups receiving 1.0 mg DOX/kg, consistent with rapid micelle clearance and the lower dose compared with 5.0 mg/kg used in previous studies [509], [510]. In contrast, implantable DOX-nanofiber, M-nanofiber, and FM-nanofiber groups exhibited strong and sustained tumor-localized fluorescence over time (Fig. 51a) [507]. Following implantation near tumors, micelles initially remained stable due to immobilization within the nanofiber matrix, preventing rapid systemic clearance. As the NFs gradually degraded in vivo, embedded micelles were slowly released and accumulated at tumor sites through interstitial passage, including diffusion through the ECM and entry into circulation via lymphatic pathways, followed by enhanced permeability and retention (EPR)-mediated accumulation and cellular uptake via endocytosis [507]. Among implantable systems, FM-nanofiber and M-nanofiber groups showed stronger tumor-associated fluorescence than DOX-nanofiber alone, indicating that micelle incorporation, particularly folate-targeted micelles, enhanced tumor accumulation. Ex vivo imaging at 48 h further confirmed higher tumor fluorescence in the FM-nanofiber group than in the DOX-nanofiber group, indicating improved carrier-mediated delivery and receptor-assisted internalization. Quantification of DOX levels by fluorescence spectroscopy (Fig. 51b) showed that, at 1, 6, 24, and 48 h post-administration, tumor accumulation in the FM and FM-nanofiber groups was significantly higher than in the free DOX group. This was attributed to the PEGylated micellar shell, which reduces recognition by the reticuloendothelial system (RES) [511], the EPR effect in tumor vasculature [512], and folate-mediated active targeting [513]. Finally, DOX accumulation in normal tissues was primarily observed in the heart, lung, liver, and kidney. Importantly, the FM-nanofiber showed decreased drug accumulation in these organs compared with free DOX and FM, confirming that this implantable system effectively limits off-target distribution and reduces systemic toxicity [507].

**Fig. 50:**
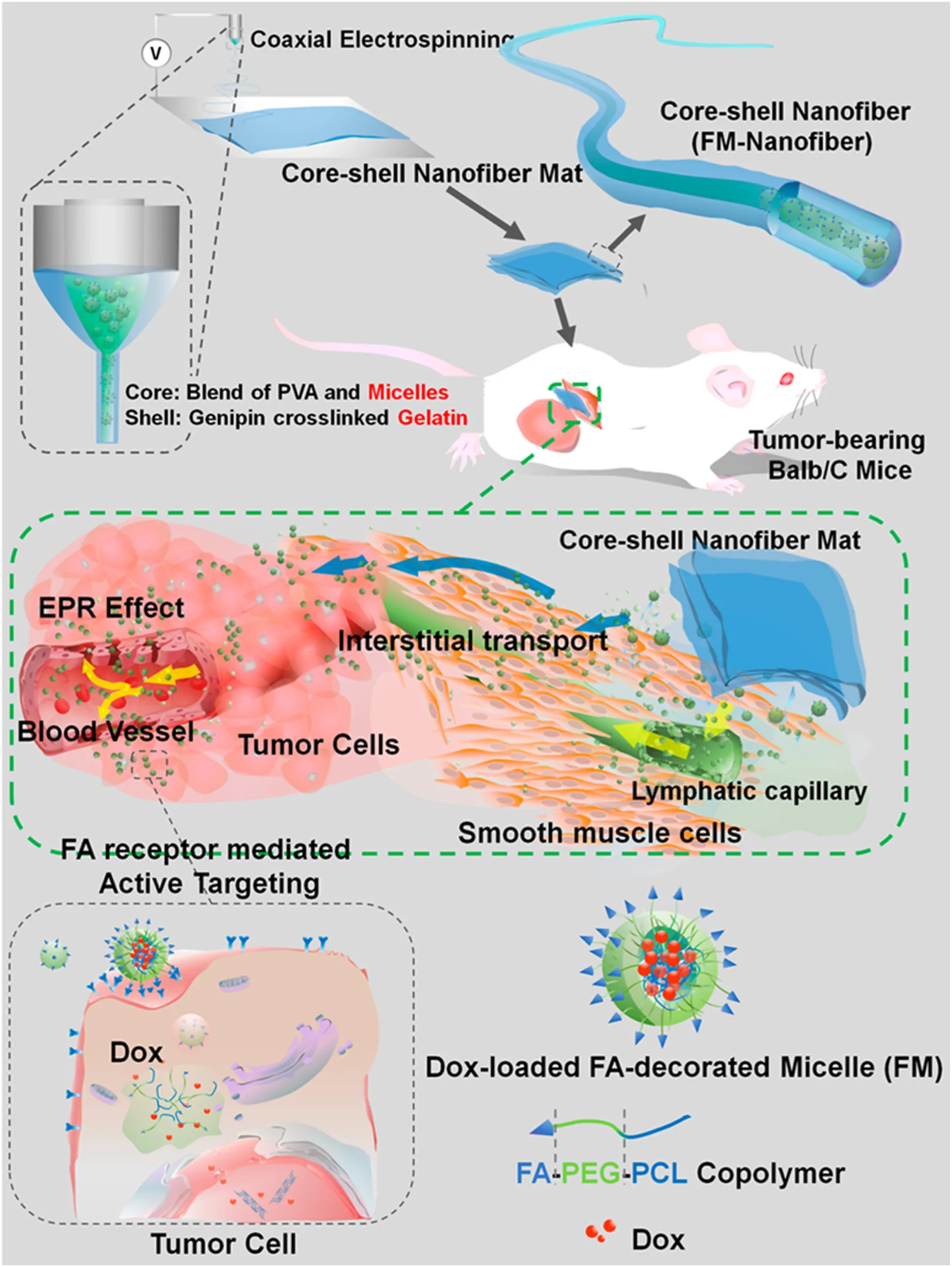
Pictorial elucidation of the fabrication of the implantable active-targeting FM-nanofiber and the delivery route of DOX-loaded micelles from nanofiber matrix to tumor tissues and finally to tumor cells [507]. Copyright 2015. Reproduced with permission from the American Chemical Society.

**Fig. 51:**
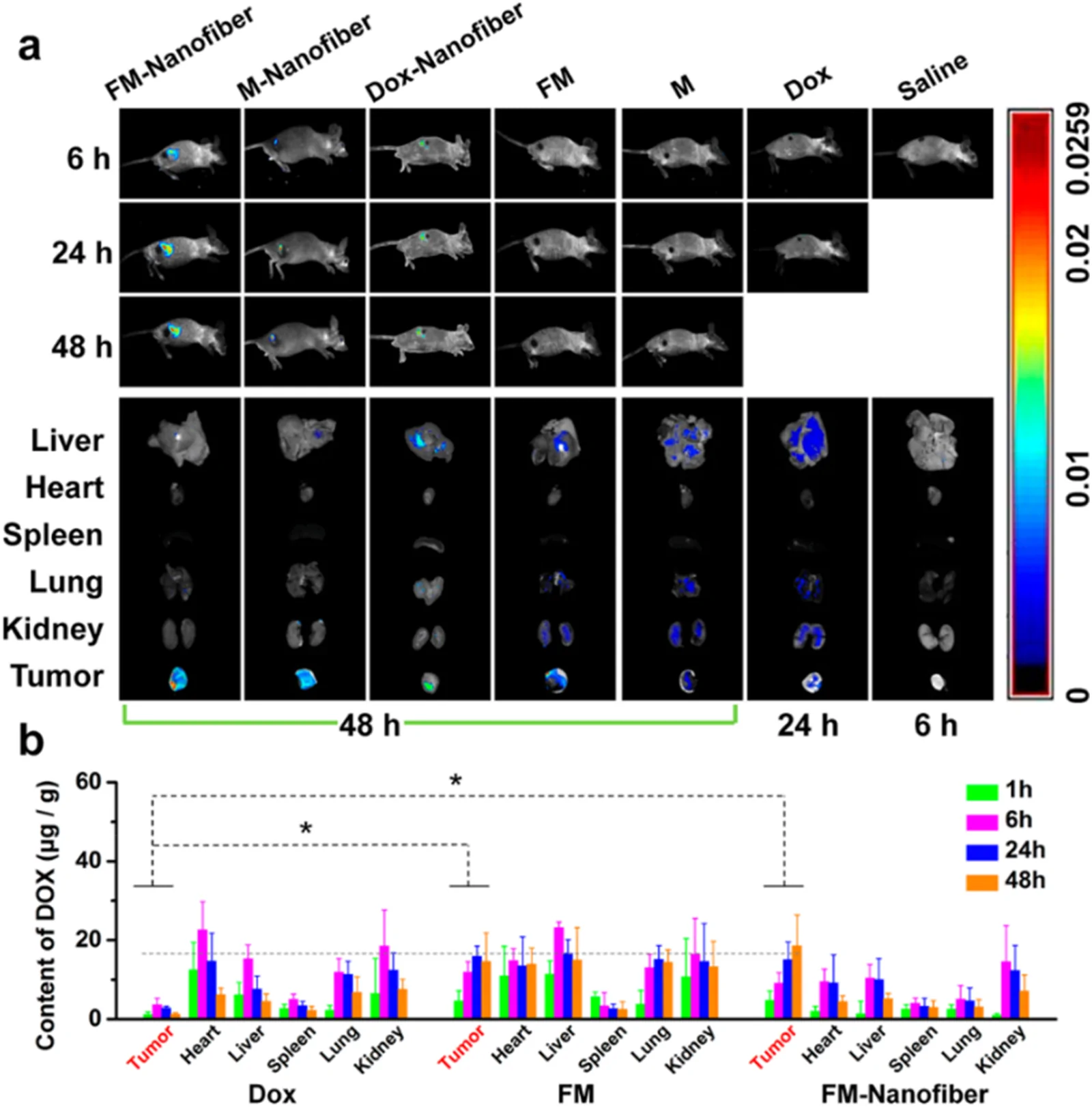
(a) In vivo and ex vivo fluorescence images of DOX showing its biodistribution in nude mice bearing 4T1 murine breast tumors following implantation of FM-Nanofiber and M-Nanofiber systems or intravenous injection of Dox, M, FM, and saline controls (dose: 1 mg Dox kg^−1^ body weight) at different time points after treatment. (b) Quantitative biodistribution of DOX in major tissues collected from mice at different time points following implantation or injection (dose: 2 mg DOX kg^−1^ body weight). Statistical significance is indicated by *P* < 0.05 [507]. Copyright 2015. Reproduced with permission from the American Chemical Society.

Another study demonstrated that integrating DOX-loaded vanadium MOFs (DOX@V-MOFs) into CS/PVA ESNF membranes can yield a unique multifunctional and stimuli-responsive DD platform [514]. The resulting membrane exhibited a dual response to pH and heat cues, substantially enhancing content release under simulated tumor environment (pH5) and high temperatures (37 °C −42 °C). Drug release profiles were consistent with zero-order, Higuchi, and Korsmeyer-Peppas models, suggesting that diffusion-driven mechanisms dominanted release behavior. The DOX@V-MOF membranes also demonstrated strong cytotoxic effect against several cancer cell lines, including liver cancer (HepG-2) and breast cancer (MCF-7), as well as notable antioxidant and antimicrobial activities against *S. aureus*, *E. coli*, and *C. albicans*. Optimization using a Box-Behnken design further uncovered that temperature, pH, and time were important factors influencing drug release dynamics [514]. Babadi et al. [515] fabricated piperine (PIP)-loaded electrospun PCL NFs as an implantable anticancer controlled-release platform for postsurgical breast cancer treatment and also investigated the effect of collagen (Coll) integration into the structure. The fabricated NFs were evaluated for shape, drug content, hydrophilicity, release behavior, mechanical features, cytotoxicity, and the underlying anticancer mechanisms in two breast cancer cell lines (MCF-7 and 4T1). The inclusion of collagen decreased the hydrophobicity of the PCL matrices, while the formulation containing 25% collagen exhibited favorable mechanical properties [515]. An in vivo antitumor study was conducted in 4T1 tumor-bearing mice, and among the tested formulations, the PIP-PCL75-Coll25 nanofiber was identified as the optimal combination because of its stable PIP release, suitable mechanical properties, and enhanced cancer toxicity. Fig. 52A illustrates the surgical procedure, during which all mice survived successfully. Photographs of tumors collected from the different experimental groups are also presented in Fig. 52B. Both surgical procedures and treatments were tolerated by animal as demonstrated by the minimal fluctuations in body weight (Fig. 52C) [515]. Efficacy results showed that tumors in both the control and PCL75-Coll25 groups grew similarly, reaching approximately 4000 mm^3^, corresponding to nearly a 10-fold increase over baseline size, as shown in Fig. 52D. Additionally, implanting the PIP-PCL75-Coll25 membrane suppressed tumor growth during the first 9 days, maintaining tumor volumes at approximately 700 mm^3^, comparable to those with PIP suspension treatment. Thereafter, the PIP-PCL75-Coll25 nanofiber exhibited significantly greater tumor inhibition, with a final tumor volume of approximately 1280 mm^3^ compared to approximately 2550 mm^3^ in the PIP suspension group. This corresponded to tumor volume increases of roughly 3-fold and 7-fold relative to the initial tumor size, respectively. Overall, the in vivo antitumor efficacy study confirmed the superiority of the PIP-PCL75-Coll25 NF compared to other groups. In contrast to the restricted PIP alone effect, these findings highlight the importance of sustained, controlled drug release to maintain appropriate drug concentrations at the target site to promote tumor growth inhibition [515].

**Fig. 52:**
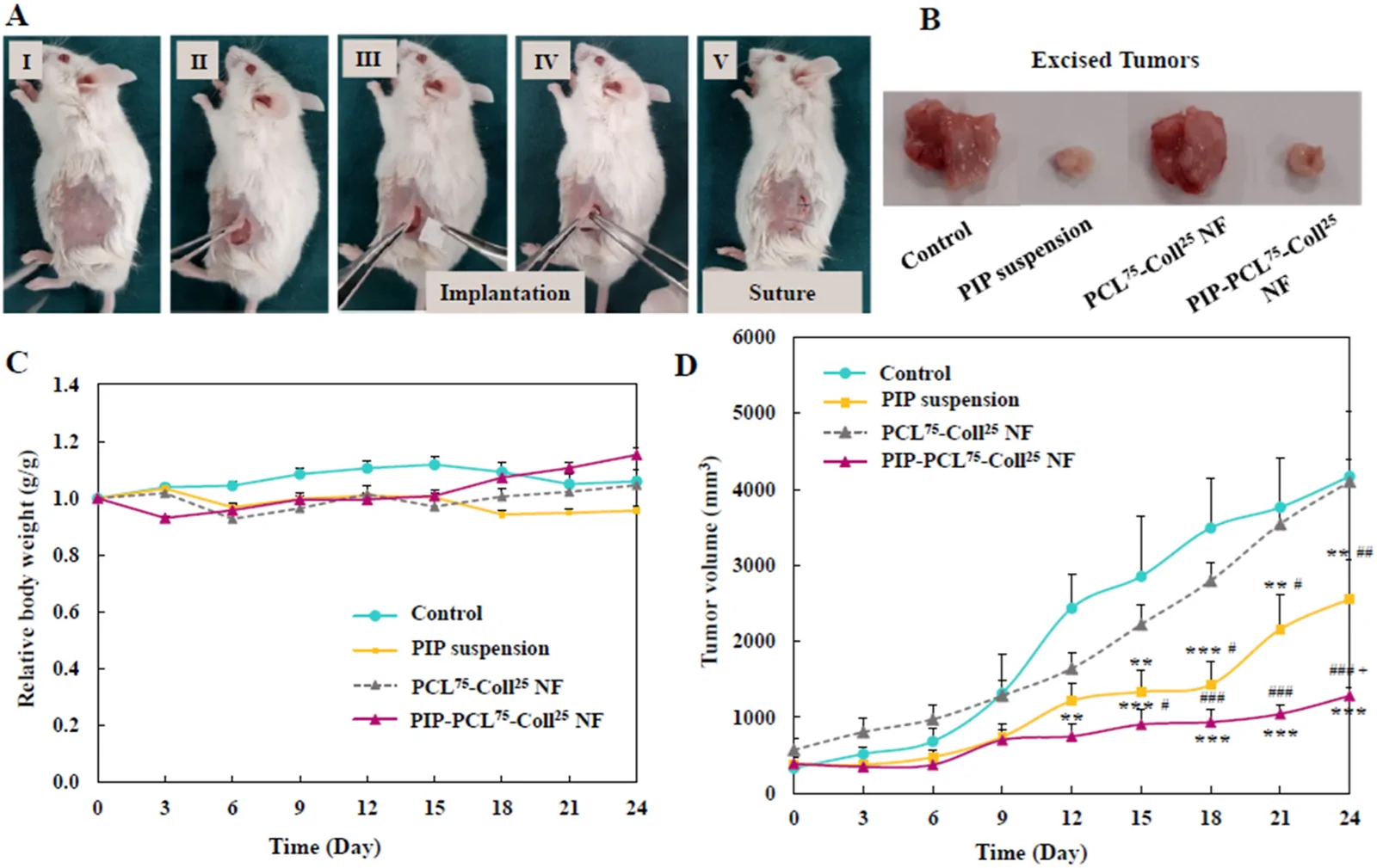
In vivo therapeutic efficacy evaluation in breast tumor-bearing mice. (A) Schematic illustration of the surgical procedure and implantation of the nanofiber formulations. (B) Representative photographs of excised tumors collected from the different treatment groups. (C) Body weight profiles of mice during the treatment period. (D) Tumor growth curves of untreated and treated groups over the 24-day study period. Data are presented as mean ± SEM. Statistical significance is indicated as follows: *** and ###, *P* < 0.001; ** and ##, *P* < 0.01 versus the control group and blank nanofiber group, respectively; #, *P* < 0.05 versus the blank nanofiber group; +, *P* < 0.05 versus the PIP suspension group [515]. Copyright 2022. Reproduced with permission from Elsevier.

In another study, Serio et al. developed biocompatible ECM-like NFs composed of PVA/gum arabic (GA) blend for in vitro testing [516]. For this, green-synthesized GA-stabilized AuNPs (GA-AuNPs) were uniformly adsorbed on blended PVA/GA ECM-like NFs and characterized using CLSM, SEM, and high-angle annular dark-field scanning transmission electron microscopy (HAADF-STEM) (Fig. 53A-C) [516]. CLSM detected intense Rhodamine B isothiocyanate (RBTIC) fluorescence. GA-AuNPs were observed as bright dots on the NFs surface (arrows - Fig. 53B and C), distributed homogeneously along the fibers, corroborating the NFs functionalization with GA-AuNPs. Confocal microscopy confirmed that GA-AuNPs adsorbed onto nanofibers were internalized by B16-F10 melanoma cells within 4 h, with cytoplasmic localization validated by actin/nuclei overlays (Fig. 53D-F) [516]. Prior work established that GA-stabilized AuNPs selectively impair melanoma cell viability and proliferation without affecting non-tumoral cells [517]. At sub-toxic concentrations, GA-AuNPs also reduced metastatic parameters such as invasion and colony formation [517]. As a proof of concept, GA-AuNP-functionalized NFs were applied to evaluate mitochondrial metabolism associated with cell viability (Fig. 54A, B) [516]. Melanoma cells cultured on PVA/GA ECM-like NFs containing 2.44, 24.4, or 244 mg/L GA-AuNPs displayed similar viability patterns to controls, with statistical significance detected only in CHL-1 cells (Fig. 54C) [516]. Meanwhile, BALB/3T3 fibroblasts showed no cytotoxicity or viability changes (Fig. 54D) [516], corroborating earlier findings [517]. These results support advancing GA-AuNPs-decorated ECM-mimetic fibers for melanoma, including migration assays and in vivo validation.

**Fig. 53:**
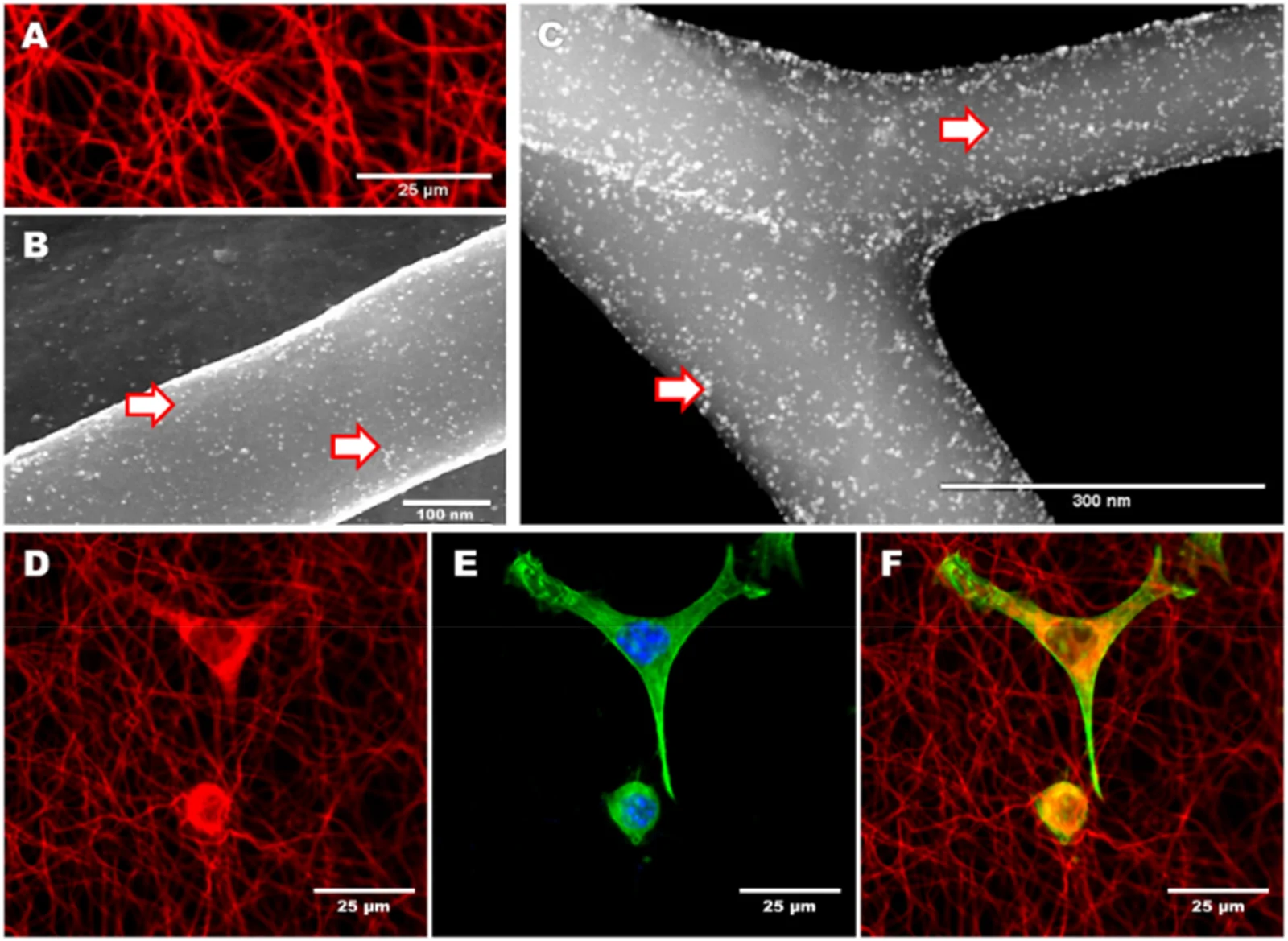
PVA/GA ECM-like NFs Blend functionalized with GA-AuNPs. RBTIC@GA-AuNPs-functionalized NFs were visualized using CLSM (A), SEM (B), and HAADF-STEM (C), with arrows indicating adsorbed GA-AuNPs. Cells cultured on these NFs were imaged by CLSM (D-F): red signals show RBTIC@GA-AuNPs on NFs (A, D) and within cells (D), blue indicates nuclei stained with DAPI (E), and green marks actin filaments stained with phalloidin in the overlay (F) [516]. Copyright 2021. Reproduced with permission from Elsevier.

**Fig. 54:**
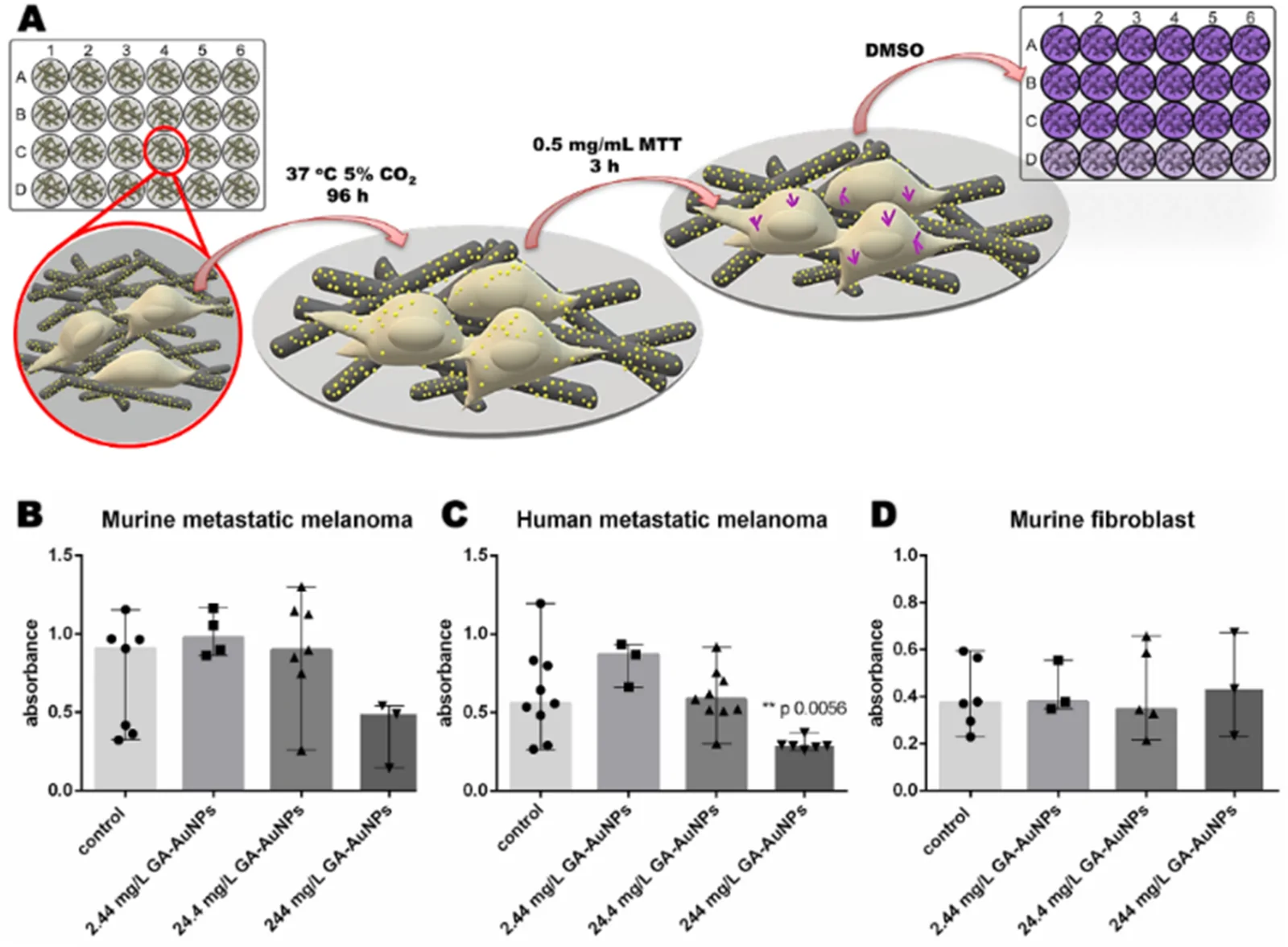
Bioactivity of GA-AuNP-functionalized blended PVA/GA ECM-like NFs was evaluated using the MTT mitochondrial metabolism assay (A). Cell viability was assessed after 96 h of culture on functionalized NFs using human melanoma (CHL-1) (C), murine melanoma (B16-F10) (B), and murine fibroblast (BALB/3T3) cells (D) [516]. Copyright 2021. Reproduced with permission from Elsevier.

In recent work, PCL nanofibers biofunctionalized with levan and egg lecithin and loaded with tanshinone IIA (Tan@Lev/EL/PCL-NF) were produced using a factorial design-guided water-in-oil emulsion ES approach [518]. The developed composite NFs exhibited a uniform shape with a mean diameter of 365.5 ± 46.2 nm, along with promising hydrophilicity and tensile strength. Specifically, Tan@Lev/EL/PCL-NF demonstrated a sustained drug-release profile, achieving complete drug release within 5 days, and providing approximately 50% skin deposition of tanshinone IIA. The anticancer effect was explored in vitro, where significant cytotoxicity of the developed NFs against squamous cell carcinoma (SCC) cells was observed, while maintaining good cytocompatibility with fibroblasts. Moreover, Tan@Lev/EL/PCL-NF exhibited pronounced apoptotic activity characterized by evident nuclear fragmentation, G2/M phase cell-cycle arrest, and strong antimigratory effects [518]. The in vivo antitumor efficacy of the composite NFs was further evaluated in tumor-bearing mice, in which the composite significantly inhibited tumor progression and reduced relative tumor weight compared with the other treatment groups. Histomorphometric analysis further confirmed the enhanced anticancer potential of the developed NFs [518]. To determine the effect of the developed nanofibers on tumor progression, investigators monitored the tumor growth as a percentage over the study duration and analyzed relative tumor weight normalized to body weight. In the untreated positive control group, tumor volume increased dramatically over the study duration (Fig. 55A), reaching a tumor growth rate of 475.02 ± 35.64% after three weeks (Fig. 55B), along with a relative tumor weight of 5.06 ± 0.006% (Fig. 55C) [518]. In contrast, the NF-treated group showed a significant reduction (p ≤ 0.05) in tumor progression after three weeks, reaching nearly a two-fold reduction compared to the control. Furthermore, this group had the lowest tumor weight (1.25 ± 0.18%) among all treatment groups. Although the remaining treatment groups also reduced tumor progression, their effects were less pronounced than those observed for Tan@Lev/EL/PCL-NF [518].

**Fig. 55:**
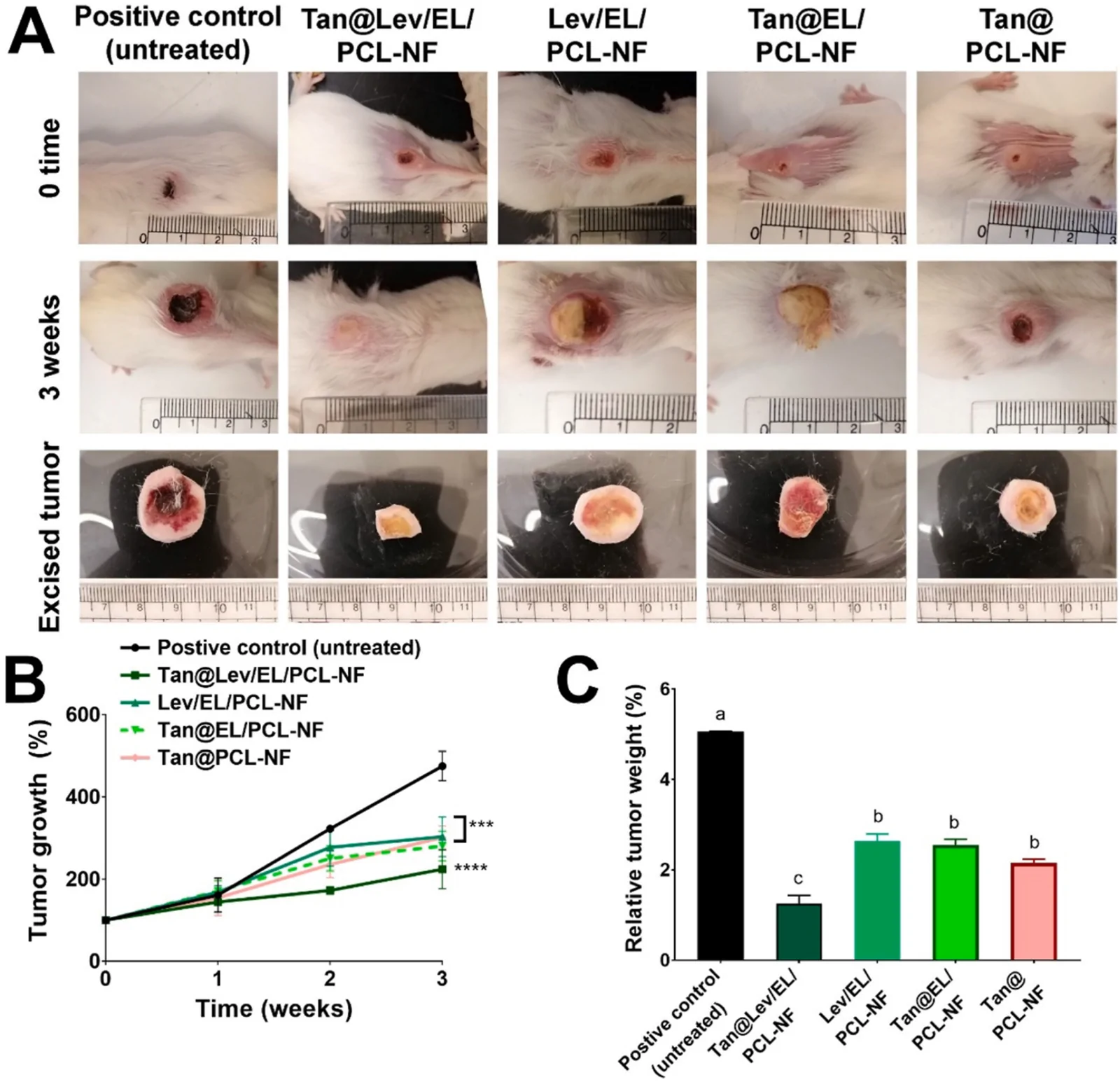
In vivo evaluation of skin tumor growth in different treatment groups. (A-C) Images representing skin tumors at baseline (0 weeks), 3 weeks after treatment, and excised tumors at the end of the study (A); percentage tumor growth at different post-induction time points (1, 2, and 3 weeks) (B); and percentage relative tumor weight following animal sacrifice (C). Data are presented as mean ± SD (*n* = 5). Columns labeled with different letters indicate statistically considerable differences (*a* > *b* > *c*, *p* ≤ 0.05). Statistical significance relative to the control group is indicated as ***P ≤ 0.001 and ****P ≤ 0.0001 [518]. Copyright 2023. Reproduced with Permission from Elsevier.

To further elucidate the anticancer mechanisms of the developed NFs, the expression levels of VEGF, B-cell lymphoma 2 (BCL2), and nuclear factor kappa B (NF-κB) proteins were analyzed in excised skin tumors collected three weeks after application. VEGF is an important mediator of angiogenesis and plays key roles in tumor growth and cancer progression [519]. VEGF was significantly downregulated in all tested groups by approximately 1.5-fold relative to the untreated control group (Fig. 56A), indicating the antiangiogenic activity of the NFs. Similarly, NF-κB expression, which is closely associated with inflammatory signaling during malignant tumor progression, was markedly reduced in all treated groups (Fig. 56B), confirming the anti-inflammatory effects of the developed formulations [520]. This finding corroborates previous studies demonstrating the anti-inflammatory properties of both tanshinone IIA [521] and levan [522], which may act synergistically to attenuate inflammation in the tumor microenvironment. In addition, the expression of the antiapoptotic protein BCL2 was significantly reduced by nearly two-fold in the Tan@Lev/EL/PCL-NF and Tan@EL/PCL-NF groups (Fig. 56C), supporting the proapoptotic activity of these formulations. Collectively, these findings suggest that the potent anticancer activity of Tan@Lev/EL/PCL-NF results from the combined effects of proapoptotic, antiangiogenic, and anti-inflammatory mechanisms [518]. Further mechanistic investigation at the gene expression level was conducted using qRT-PCR to evaluate apoptotic caspase-3, the apoptosis-regulating gene BAX, and the proliferation-associated marker MKi67. The developed NF formulation significantly upregulated caspase-3 and BAX expression while markedly suppressing MKi67 expression, with fold differences of 34.3 ± 2.6, 14 ± 1.2, and 6.9 ± 1.6, respectively, compared with control group (Fig. 56D-F). Similar trends were observed for the remaining treatment groups, although to a lesser extent. These findings further confirmed the strong proapoptotic and antiproliferative activities of the developed composite NFs [518]. Table 5 gives an overview of ESNFs utilized in cancer diagnostics and therapy. All these studies have demonstrated suitable in vitro release profiles from drug-loaded ESNFs; however, translating these results into clinical practice remains elusive. The in vivo tumor microenvironment is highly diverse, and various factors, including abnormal vasculature, elevated interstitial fluid pressure, hypoxia, enzymatic activity, and immune-cell interactions can influence it. All these factors can substantially affect the drug's distribution and therapeutic efficacy of the drug. Thus, future studies should compare in vitro release data with in vivo pharmacokinetic and pharmacodynamic results to better anticipate clinical performance.

**Fig. 56:**
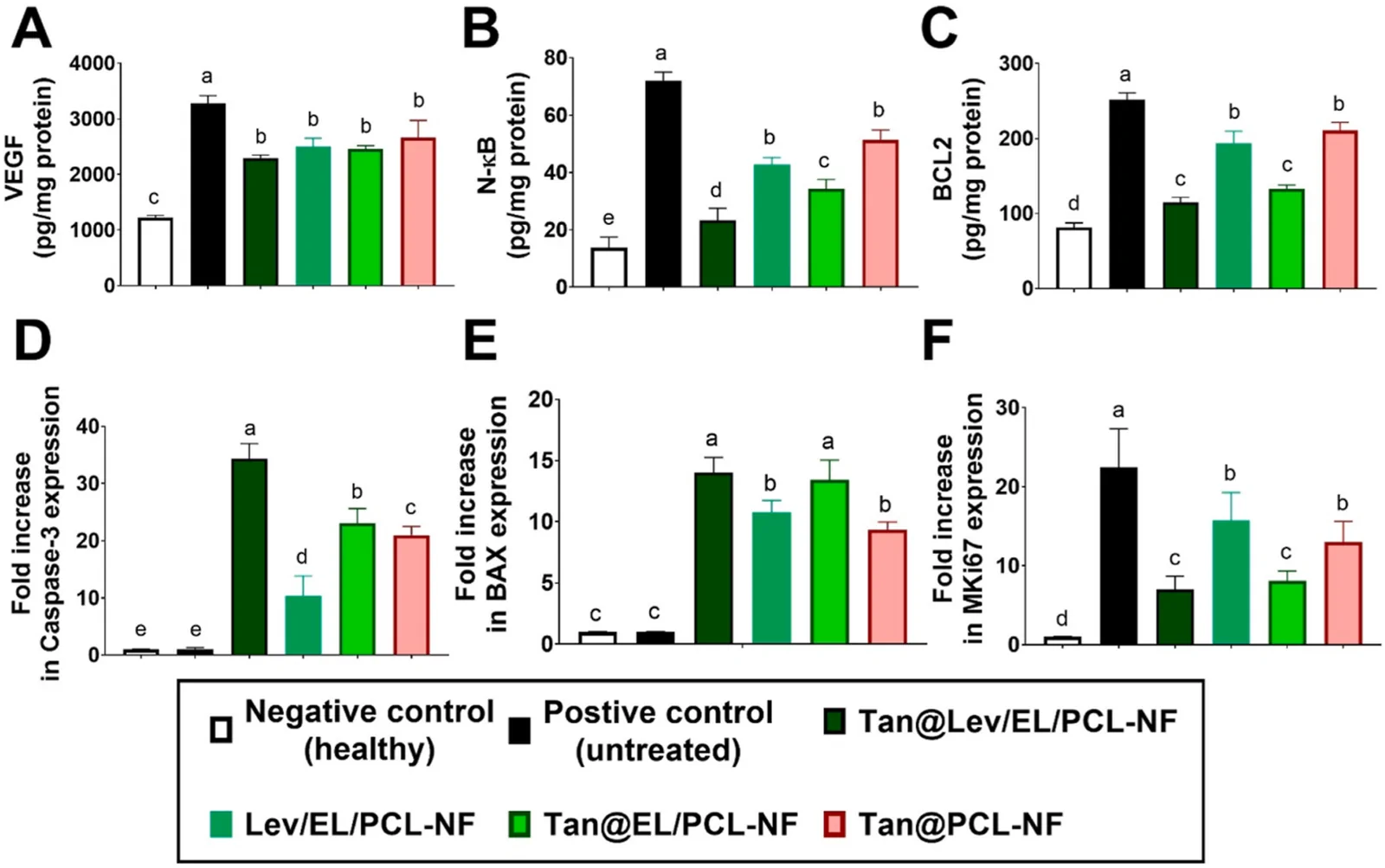
(A-F) Analysis of tumor biomarkers in eliminated skin tumors from diverse treatment groups in the in vivo study. Protein expression levels of VEGF (A), nuclear factor kappa B (NF-κB) (B), and B-cell lymphoma 2 (BCL-2) (C), quantified by ELISA. Relative gene expression levels of caspase-3 (D), BAX (E), and MKi67 (F), determined by quantitative real-time PCR (qRT-PCR) and normalized to glyceraldehyde 3-phosphate dehydrogenase) (GAPDH) expression as the housekeeping gene using the 2ˆ-ΔΔCt method. Data are presented as mean ± SD (*n* = 3). Columns labeled with different letters indicate statistically significant differences (*a* > *b* > *c* > *d* > *e*, *p* ≤ 0.05) [518]. Copyright 2023. Reproduced with Permission from Elsevier.

**Table 5:** Overview of ESNFs utilized in cancer diagnostics and therapy.

Delivery System	Fiber Diameter	Drug	Drug Loading Content	Drug Release Mechanism	Drug release time	Type of Cancer	Ref.
Poly-L-lactide	1033 nm	5-fluorouracil (5-FU)	1.6-12.8%	Diffusion	240 h	Human colorectal cancer HCT-116 cells	[523]
PCL/poly(glycerol monostearate co-ε-caprolactone) (PGC-C18)	7.2-7.7 μm	Camptothecin 11(CPT-11) and 7-ethyl-10-hydroxyc amptothecin (SN-38)	99%	Diffusion	90 d	Human colorectal cancer HT-29 cells	[524]
PLLA	1.52-2.52 μm	Titanocene dichloride	4.0-9.7%	Degradation of the polymeric matrix	184 h	Human lung cancer spc-a-1 cells	[525]
PCL/gelatin	607.4-536.6 nm	7-ethyl-10-hydroxy camptothecin (SN-38)	86.5-91.8%	Diffusion and anomalous transport	6 weeks	Human glioblastoma 251 and U87 cells	[526]
PLGA/PEO	200 nm	Ferulic acid	N/A	Diffusion and degra dation of polymeric matrix	240 h	Human breast carcinoma MCF-7 cells	[527]
PLA	300 nm	5-fluorouracil and oxaliplatin	4 and 20%	Diffusion	48 h	Human colorectal cancer HCT-8 cells and mouse colorectal cancer CT-26 cells	[528]
Poly(ethylene glycol) poly(l-lactic acid) (PEG-PLLA)	690-1350 nm	1,3-bis(2 chloroethyl)-1-nitro sourea (BCNU	5-30%	Diffusion	72 h	Rat glioma C6 cells	[529]

### 4.6 Biosensing

Biosensors integrate biorecognition elements with physicochemical transducers, a design that allows precise detection of target analytes while ensuring both high sensitivity and selectivity. These systems translate biomolecular interactions, such as antigen-antibody binding and enzyme-substrate catalysis, into measurable signals, including current, impedance, or fluorescence, thereby playing a crucial role in disease diagnostics and biomedical monitoring. The performance of biosensors largely relies on two key factors: the accuracy of molecular recognition and the efficiency of signal transduction. In this regard, NF-based substrates have emerged as favorable platforms for overcoming the limitations of conventional sensors, particularly in the development of wearable devices and health monitoring [530]. ESNFs are especially attractive because they can optimize the entire sensing cascade, including analyte recognition, signal transduction, and output generation, thereby facilitating the creation of intelligent, very sensitive, and user-friendly sensing systems [530], [531]. Owing to their exceptionally high surface-to-volume ratio, ESNFs provide abundant active sites for analyte interaction and effective immobilization of biorecognition molecules [531]. Moreover, their interconnected porous architecture minimizes diffusion barriers and promotes rapid mass transport toward active sensing regions, ultimately enhancing sensor responsiveness and detection efficiency [532]. Reliable sensing performance, however, requires careful optimization of nanofiber hydrophilicity, mechanical strength, and structural stability by appropriately selecting polymer compositions and ES parameters. To further improve analytical performance, hybrid ESNFs can be fabricated by incorporating nanoscale fillers that serve as catalytic, conductive, or affinity-enhancing components. Commonly employed nanomaterials include metallic NPs, graphene-derived quantum dots (QDs), CNTs, and graphene oxide sheets, all of which contribute to improved mechanical, optical, thermal, and electron-transfer properties while simultaneously providing numerous anchoring sites for sensing probes [533], [534]. In addition, various post-fabrication modification strategies, including LBL assembly, atomic layer deposition, dip-coating, spraying, plasma activation, and annealing, can be applied to tailor interfacial chemistry and construct hierarchical surface structures [535]. Among these techniques, dip-coating and spraying are particularly beneficial because they offer simple and cost-effective methods for decorating ESNFs with 0D-2D nanostructures, thereby enabling synergistic improvements in biosensor sensitivity, signal amplification, and overall analytical performance [536].

Maleki et al. [537] fabricated a biosensor by modifying a carbon screen-printed electrode (SPCE) with ESNFs containing HPMC, eudragit, and zeolitic imidazolate framework-8 (ZIF-8) NPs for the selective and sensitive electrochemical recognition of mesenchymal-epithelial transition factor (c-MET). The developed Eudragit/HPMC/ZIF-8 NFs, cast onto the SPCE as an effective modifier, enhanced electron transfers at the electrode surface and increased the electroactive surface area, thereby improving the conductivity and stability of the biosensor. Furthermore, the ZIF-8 NPs not only increased the electron transfer rate but also facilitated the stable immobilization of anti-c-MET on the modified electrode. The fabricated biosensor exhibited excellent analytical performance, including a wide detection range from 100 fg mL^−1^ to 100 ng mL^−1^, a LOD of ∼1.3 fg mL^−1^, enhanced selectivity, and long-term stability than the other c-MET biosensors. As a non-invasive sensing platform, this biosensor was effectively tested on human plasma samples. However, its major limitation was the relatively long analysis time [537].

Fu et al. [538] developed a non-enzymatic glucose sensor by decorating carbon NFs with Ni-Co layered double hydroxide (LDH) nanosheets (carbon nanofiber@Ni-Co LDH), resulting in a flake-like LDH shell uniformly grown on the carbon nanofiber backbones, with diameters ranging from 300 to 500 nm. The ES carbon nanofiber network provided a conductive and macroporous scaffold that reduced LDH agglomeration and improved electrolyte accessibility. At the same time, the Ni-Co hydroxide layer supplied abundant redox-active sites for glucose oxidation. Optimization of the Ni:Co ratio to 2:1 yielded the highest catalytic activity, as evidenced by pronounced Ni^2+^/Ni^3+^ and Co^2+^/Co^3+^ redox peaks and a rapid amperometric response. Under optimized conditions, the sensor revealed a wide linear detection range (1.0-2000 μM), an excellent low LOD of 0.03 μM, and a high sensitivity of 1.47 mA mM^−1^ cm^−2^, along with excellent selectivity and stability [538].

In another related study, transparent electrospun nanofibrous hydrogels based on PVA and β-cyclodextrin (β-CD) were prepared and integrated with reverse iontophoresis electrodes to develop a non-invasive biosensing platform for longitudinal glucose monitoring in interstitial fluid [539]. Fig. 57a illustrates the fabrication process of a patch-style glucose sensor based on PVA/1,2,3,4-butanetetracarboxylic acid (BTCA)/β-CD/GOx/AuNP nanofibrous hydrogels and the corresponding sensing mechanism for real-time sweat glucose monitoring. Fig. 57b,c presents the preparation process of the ES solution containing the PVA/BTCA/β-CD/GOx/AuNP complex, along with optical and SEM micrographs of the resulting transparent nanofibrous structures [539]. The prepared composite hydrogels showed excellent flexibility, biocompatibility, absorptive capacity, mechanical features in both dry and wet states, and high enzyme activity (around 76%). Given the distinct characteristics of the AuNP-incorporated nanofibrous hydrogels, including high permeability to biological molecules and fast electron transfer, the biosensor demonstrated outstanding sensing performance, characterized by a wide linear range, high sensitivity (47.2 μA mM^−1^), a low detection limit (0.01 mM), and a rapid response time (<15 s). The electrochemical behavior of the GOx-loaded nanofibrous hydrogels hydrated with PBS (pH 7.4) was evaluated by cyclic voltammetry (CV). Fig. 58a shows the CV curves of PVA/BTCA, PVA/BTCA/β-CD, PVA/BTCA/GOx, PVA/BTCA/β-CD/GOx, and PVA/BTCA/β-CD/GOx/AuNP hydrogels at a scan rate of 0.1 V s^−1^. A photograph of the fabricated hydrogel-based non-invasive biosensor patch is presented in Fig. 58b. Electrochemical characterization (Fig. 58c) revealed that the PVA/BTCA/β-CD/GOx/AuNP hydrogel exhibited quasi-reversible redox peaks at Epa = 0.624 V and Epc = −0.637 V. The AuNP-functionalized hydrogel showed a substantially higher background current than the other hydrogels, consistent with enhanced conductivity [539]. The formal potential was −6.5 mV, and the small ΔEp (∼13 mV) confirmed rapid, quasi-reversible electron transfer. CV measurements in PBS (10 mM, pH 7.4) demonstrated a linear decrease in cathodic peak current with increasing glucose concentrations (0.1-0.5 mM). Chronoamperometric experiments performed with PVA/BTCA/GOx, PVA/BTCA/β-CD/GOx, and PVA/BTCA/β-CD/GOx/AuNP hydrogel biosensors at room temperature (Fig. 58d–f) revealed clear differences in sensing behavior among the systems [539]. The resulting current profiles underscored substantial differences in sensing capabilities among the tested membranes. The PVA/BTCA/β-CD/GOx/AuNP hydrogel sensor demonstrated superior accuracy and responsiveness, whereas the PVA/BTCA/GOx hydrogel, which performed poorly due to diminished enzyme activity and stability. A calibration curve of steady-state current versus glucose concentration was constructed for the AuNP-functionalized sensor, further validating its enhanced performance (Fig. 58e, inset). While standard deviation increased with increasing glucose concentration, the sensor maintained strong linearity (R^2^ = 0.98) and delivered a rapid response time of <15 s. Key performance parameters included a detection limit of 0.01 mM, a linear range spanning 0-0.5 mM, and a sensitivity of 47.2 μA mM^−1^ [539]. Given its ease of fabrication, scalability, and excellent electrochemical properties, the transparent NF hydrogel biosensing platform represents a promising candidate for wearable monitoring, clinical diagnostics, and diverse biomedical applications [539].

**Fig. 57:**
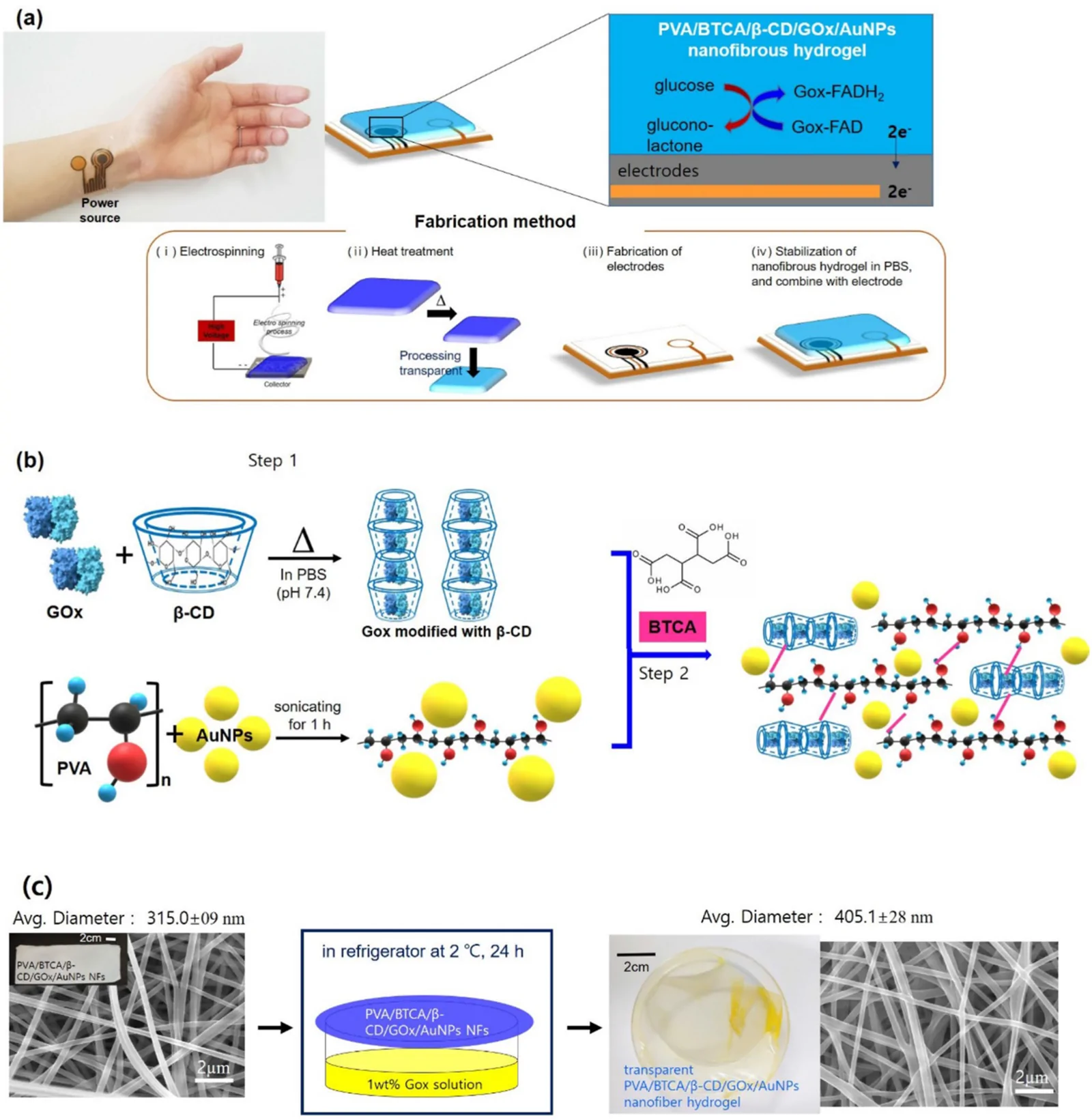
(a) Pictorial representation of a patch-type glucose sensor based on PVA/BTCA/β-CD/GOx/AuNP nanofibrous hydrogels and its glucose-sensing mechanism for non-invasive, real-time sweat glucose monitoring. (b) Graphic depiction of the preparation of the PVA/BTCA/β-CD/GOx/AuNP ES solution. (c) Fabrication process of transparent PVA/BTCA/β-CD/GOx/AuNP nanofibrous hydrogels, together with representative optical and SEM images [539]. Copyright 2020. Adapted from Springer Nature.

**Fig. 58:**
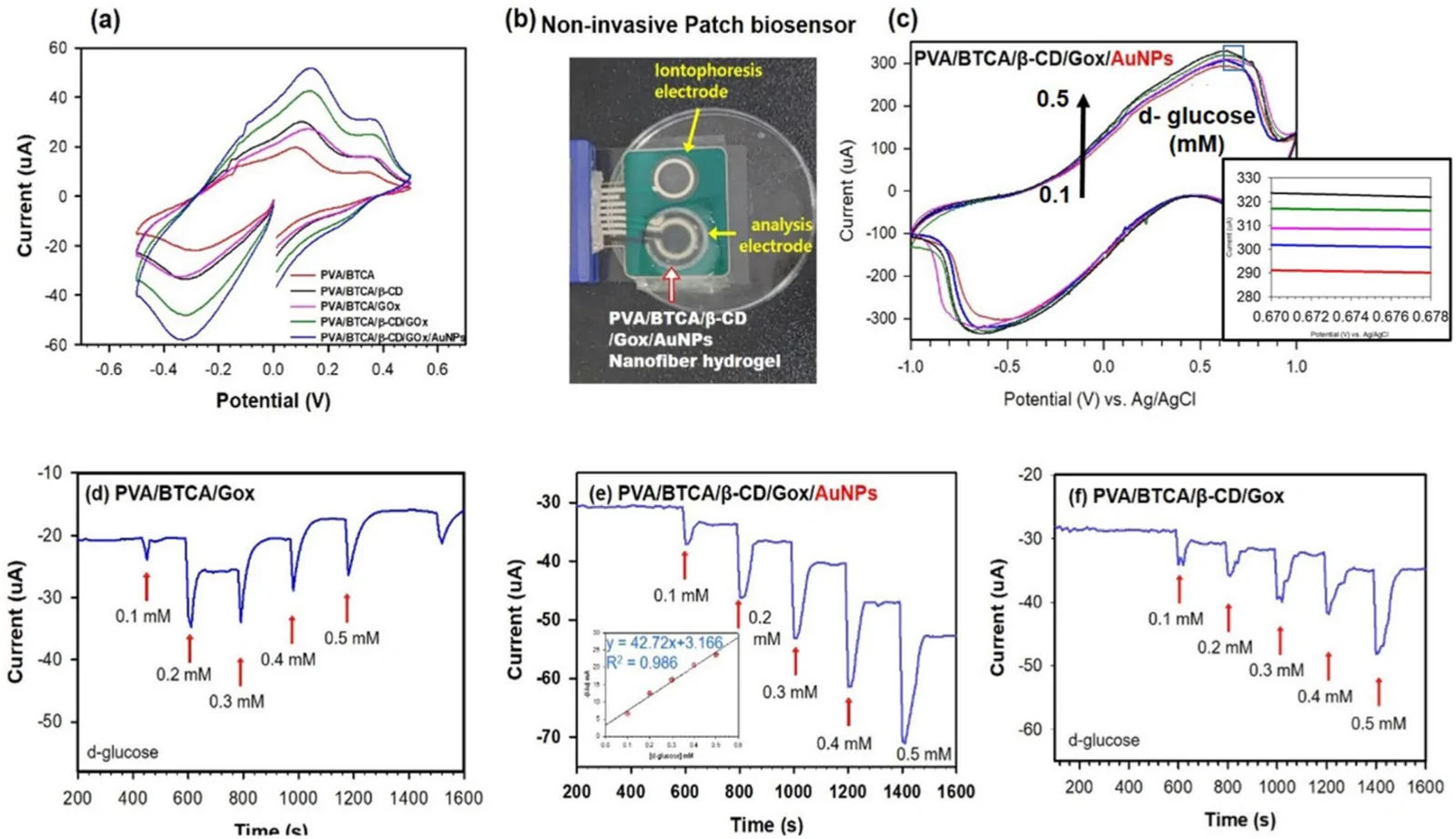
(a) CV analyses of PVA/BTCA, PVA/BTCA/β-CD, PVA/BTCA/GOx, PVA/BTCA/β-CD/GOx, and PVA/BTCA/β-CD/GOx/AuNP nanofibrous hydrogels prepared in PBS (pH 7.4), recorded at a scan rate of 0.1 V s^−1^. (b) Photograph of the patch-style AuNP based nanofibrous hydrogel biosensor during a three-electrode electrochemical measurement. (c) CV analyses of AuNP based nanofibrous hydrogel biosensor tested with different glucose concentrations in PBS (pH 7.4) at room temperature. Measurements were done at a scan rate of 0.1 V s^−1^. Chronoamperometric responses were measured at −0.2 V (vs. Ag/AgCl) for (d) PVA/BTCA/GOx, (e) PVA/BTCA/β-CD/GOx/AuNP, and (f) PVA/BTCA/β-CD/GOx nanofibrous hydrogel biosensors. The inset in panel (e) displays the calibration plot of biosensor [539]. Copyright 2020. Adapted from Springer Nature.

Mondal et al. developed a surface-functionalized, aligned mesoporous anatase TiO_2_ nanofiber (TiO_2_ NF) mat that, following oxygen plasma activation and EDC/NHS coupling, enabled the dense and stable attachment of cholesterol esterase (ChEt) and cholesterol oxidase (ChOx) on an indium tin oxide (ITO) substrate for the electrochemical sensing of esterified cholesterol [540]. The ES process and porosity were tuned by incorporating PVP as a sacrificial carrier polymer into the titanium isopropoxide precursor solution. For biofunctionalization, fresh solutions of ChEt and ChOx (1 mg mL^−1^ each) were mixed at a ratio of 1:1. Subsequently, 10 μL of the enzyme mixture was uniformly deposited onto the oxygen plasma-treated TiO_2_ nanofiber surface (cTiO_2_-NF/ITO) via physical adsorption and incubated at 4 °C for 12 h. The functional groups (-COOH, -CHO, and -OH) generated on the cTiO_2_-NF/ITO surface were used for the covalent immobilization of enzymes through interactions with terminal -NH_2_ groups, ensuing in the establishment of amide bonds, as shown in Fig. 59 [540]. The high mesoporosity (61%) and small fiber diameter (30-60 nm) significantly increased enzyme loading and facilitated rapid electron transfer, thereby enhancing sensing. As shown in Fig. 60a, CV measurements revealed a clear concentration-dependent increase in anodic peak current with increasing cholesterol oleate concentration from 25 to 400 mg dL^−1^. The inset highlights the pronounced increase in peak current at lower concentrations. Fig. 60b presents the calibration characteristics of the biosensor. The biosensor demonstrated a wide detection range and a high sensitivity of 181.6 μA mg^−1^ dL cm^−2^, which compares favorably with previously reported microfluidic anatase TiO_2_ nanoparticle-based cholesterol biosensors [541]. The increased analytical parameters can be attributed to the covalent immobilization of enzymes on oxygen-activated TiO_2_ NFs and to the conductive nanofibrous network, which promotes effective electron transfer between the biorecognition layer and the electrode. In addition, the enhanced sensitivity may be attributed to the formation of TiO_2_ NFs, which provide efficient pathways for electron transfer between the solution and the surface, thereby improving current density. Similar observations have been reported for aligned carbon nanofiber electrodes, where superior electrochemical performance was attributed to more efficient electron movement from the electroactive species to the signal detector [542]. Therefore, the exceptional charge-transfer characteristics of TiO_2_ NFs make them ideal for enhanced biosensing performance. Furthermore, the TiO_2_-NF biosensor achieved a low LOD of 19.3 mg dL^−1^ and demonstrated excellent selectivity and operational stability. As shown in Fig. 61i, various common interferents, such as ascorbic acid (AA), uric acid (UA), glucose, lactic acid, and urea, generated negligible current responses compared with cholesterol, proving the superior specificity of the sensing platform [540]. Fig. 61(ii) illustrates the reproducibility and stability of the biosensor. Repeated measurements (n = 10) at a cholesterol concentration of 200 mg dL^−1^ exhibited minimal signal variation, with a relative standard deviation (RSD) of only 3.19%. Moreover, the inset demonstrates consistent current responses over multiple measurement cycles. Collectively, these findings highlight the excellent sensitivity, selectivity, reproducibility, and operational durability of the TiO_2_ nanofiber-based biosensor for cholesterol detection [540].

**Fig. 59:**
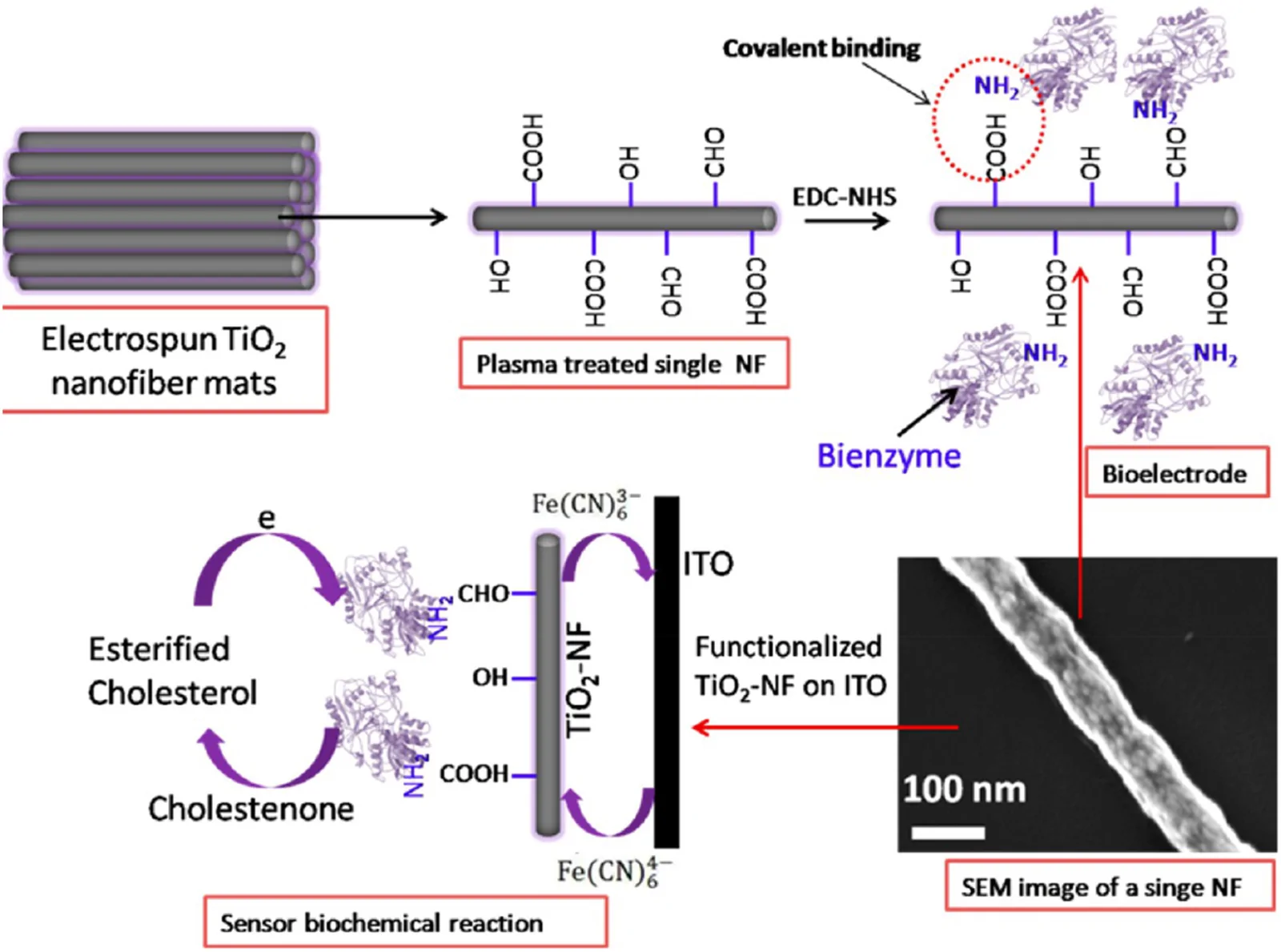
Schematic illustration of the biofunctionalization of mesoporous TiO_2_ NFs for the electrochemical detection of esterified cholesterol [540]. Copyright 2014. Reproduced with permission from American Chemical Society.

**Fig. 60:**
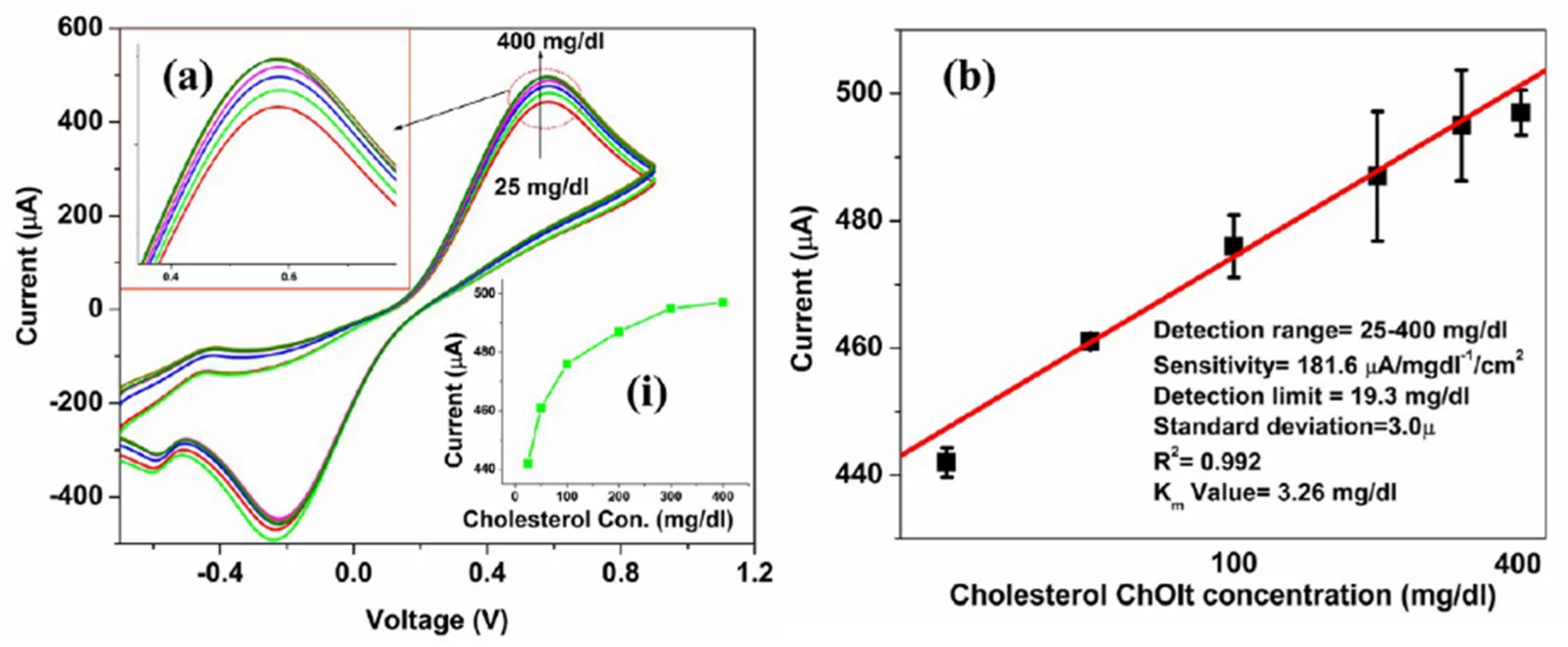
(a) CV response of the ChEt-ChOx/cTiO_2_-NF/ITO bioelectrode as a function of cholesterol oleate concentration (25-500 mg dL^−1^). Inset: magnified view of the anodic peak response. Inset (i): Plot of electrochemical current versus cholesterol oleate concentration. (b) Plot of electrochemical current versus logarithm of cholesterol oleate concentration [540]. Copyright 2014. Reproduced with permission from American Chemical Society.

**Fig. 61:**
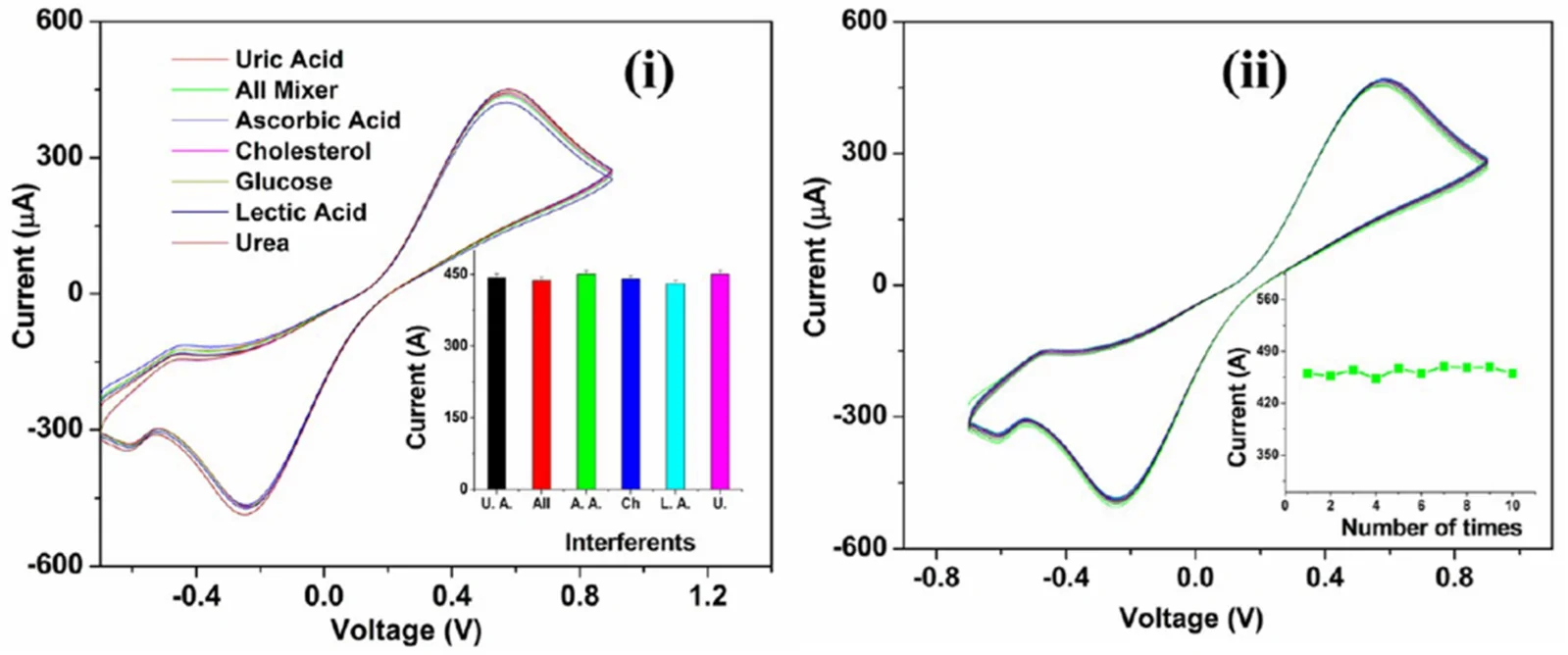
(i) Selectivity study of the ChEt-ChOx/cTiO_2_-NF/ITO bioelectrode in the presence of common interfering species. (ii) Reproducibility and stability evaluation of the ChEt-ChOx/cTiO_2_-NF/ITO bioelectrode. Inset: Current response of the ChEt-ChOx/cTiO_2_-NF/ITO bioelectrode during repeated measurements [540]. Copyright 2014. Reproduced with permission from American Chemical Society.

Electrospun platforms are also greatly employed in the development of non-enzymatic glucose sensors. Adabi et al. fabricated nickel-modified carbon nanofibers by ES of PAN polymer, followed by stabilization and carbonization to produce carbon nanofibers, and subsequent electrodeposition of Ni onto the nanofiber surface [543]. The average diameter of the fiber declined from 124.4 nm (ES PAN fibers) to 85.3 nm (after carbonization). SEM, EDS, and XRD assessments endorsed the successful and uniform incorporation of Ni into the carbon NF structure. The sensor exhibited a rapid response, a two-concentration-level linear detection range from 2.0 μM to 5.0 mM, a low LOD of 0.57 μM, and excellent stability and selectivity. Furthermore, the sensor demonstrated promising performance in real sample analysis [543]. In a related study, graphene and mixed Co/Mn oxide catalysts were incorporated into composite microfibers to fabricate a high-performance non-enzymatic glucose sensor [544]. Fig. 62a summarizes the synthesis procedure, in which graphene was first dispersed in DMF, followed by the addition of PVP to form a spinnable solution. Subsequently, cobalt and manganese acetates were introduced before ES [544]. The resulting fibers were electrospun under 18 kV applied voltage, a distance of 10 cm (from the collector), and a flow rate of 1 mL h^−1^. After drying at 75 °C, the fibers were calcined under an argon atmosphere at 650 °C to obtain graphene/metal oxide composite microfibers (GMCFs). Optimization of the operating conditions identified 0.2 M NaOH as the supporting electrolyte, +0.55 V as the working potential, and 4.0 mg mL^−1^ GMCFs as the optimum catalyst loading. Under these conditions, the GMCF-modified glassy carbon electrode (GCE) exhibited rapid and stable amperometric responses, reaching a steady-state current within 1.5 s following each successive glucose addition, as shown in Fig. 62b [544]. The sensor displayed a linear glucose response over a range of 5.0 nM to 800 μM with a correlation coefficient (*R*^2^) of 0.995. In addition, it achieved a high sensitivity of 1.81 mA mM^−1^ cm^−2^ and an exceptionally low detection limit of 1.0 nM, as illustrated in Fig. 62c [544].

**Fig. 62:**
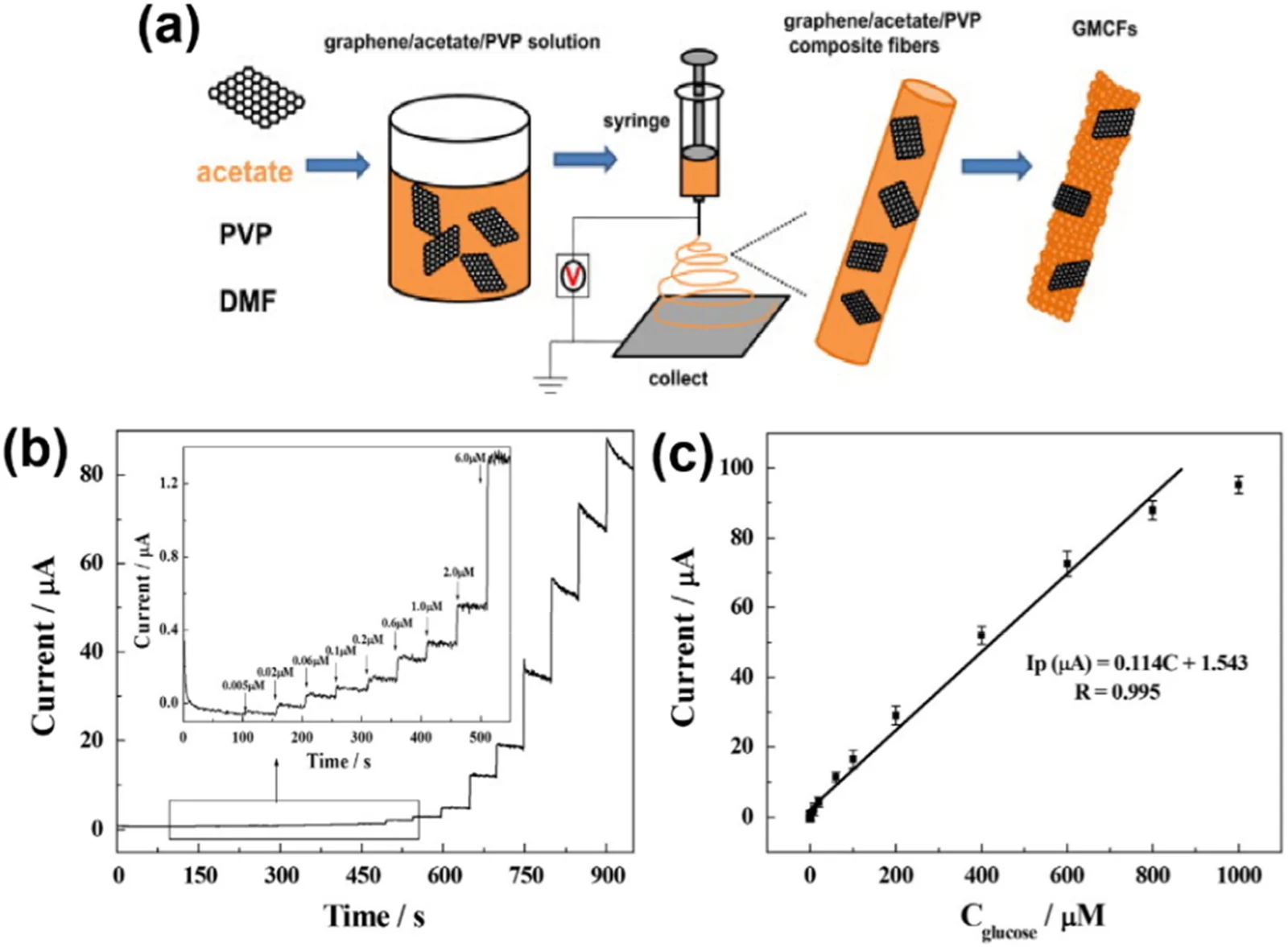
(a) Schematic of the major steps in GMCFs synthesis. (b) Current-time curves recorded at GMCF/GCE under varying glucose concentrations in 0.2 M NaOH at +0.55 V, with inset showing responses at low concentrations. (c) Correlation between amperometric signals and glucose concentration [544]. Copyright 2015. Reproduced with permission from Elsevier.

In another work, enzyme-loaded NFs directly interfaced with microstructured electrodes were fabricated by ES of PVA/PEI/glucose oxidase (PVA/PEI/GOx) blends onto gold microelectrodes, forming a conformal and bioactive nanofibrous coating with high enzyme loading and strong adhesion [545]. The resulting nanostructured gold electrodes were further functionalized using electrospun PVA/PEI/GOx fibers assembled on a 4-aminothiophenol self-assembled monolayer, followed by brief glutaraldehyde crosslinking and decoration with Au NPs to enhance conductivity and catalytic activity [545]. EIS measurements in PBS (0.1 M, pH 5) divulged that the charge-transfer resistance increased with glucose concentration (0-1.0 mM) only in the presence of GOx, whereas enzyme-free controls showed no measurable response. These measurements confirm that the impedance change originates from enzymatic reactions that produce electroactive species such as H_2_O_2_ and gluconic acid. Rinsing with PBS restored the baseline signal, indicating reversible interfacial product accumulation and stable immobilization of both GOx and Au NPs [545]. The resulting PVA/PEI/GOx/AuNP nanofiber-modified electrodes exhibited a linear response over 10-200 μM glucose with an LOD of 0.9 μM. The system also demonstrated preserved enzyme activity, good electrode-to-electrode reproducibility, and stable performance under repeated measurements. This study highlights how integration of enzyme-loaded NFs with microstructured gold electrodes, combined with nanoparticle-mediated conductivity enhancement, enables highly sensitive, stable, and miniaturizable electrochemical biosensing platforms [545].

Zhao et al. established a self-powered electrochemical biosensor in which electrospun PAN/carbon nanotube (PAN/CNT) NFs served as a conductive and structural backbone for immobilizing AuNPs and constructing a dual-electrode enzymatic biofuel cell (EBFC) platform for myoglobin (Mb) detection [546]. The system consists of a PAN/CNT-COOH-AuNP-GOx bioanode for glucose oxidation and a PAN/CNT-COOH-AuNP-aptamer biocathode for selective binding of Mb [546]. The ESNFs provide large surface area, adjustable porosity, and outstanding conductivity, while CNT incorporation enhances electron transport and AuNPs improve biocompatibility and enable aptamer immobilization via Au-S chemistry [547], [548]. As illustrated in Fig. 63, the presence of Mb modulates electron flow: in the absence of Mb, electrons generated at the GOx bioanode reduce the [Fe(CN)_6_]^3-^ redox mediator at the cathode, resulting in a high open-circuit voltage (E^OCV^). Upon Mb binding, steric hindrance at the aptamer-functionalized cathode impedes electron transfer, resulting in a concentration-dependent decrease in E^OCV^ [546]. This architecture enabled highly sensitive Mb detection over a range of 5.0 ng mL^−1^ to 5 × 10^3^ ng mL^−1^, with an ultralow LOD of 0.23 ng mL^−1^. Polarization studies further showed a decrease in peak power density from 19 μW cm^−2^ to 3.5 μW cm^−2^ upon Mb binding, supporting a steric hindrance-based sensing mechanism [546]. Selectivity tests demonstrated negligible interference from BSA, cytochrome *c*, and sulfite oxidase, even at fivefold higher concentrations (Fig. 64) [546]. Long-term stability studies showed nearly 100% signal retention after 2000 s, while four-day stability tests confirmed consistent E^OCV^ output. Inductively coupled plasma (ICP) analysis further indicated ∼98% AuNP retention [546]. The sensing mechanism is based on strong Au-S bonds that anchor the aptamer to the AuNP-modified cathode. In the absence of Mb, electrons from glucose oxidation at the anode are efficiently transferred to the cathode, where [Fe(CN)_6_]^3-^ is reduced to [Fe(CN)_6_]^4-^, generating a high open-circuit voltage [549]. Upon Mb binding, steric hindrance from the bulky protein structure inhibits electron transfer, resulting in a decreased E^OCV^ [550].

**Fig. 63:**
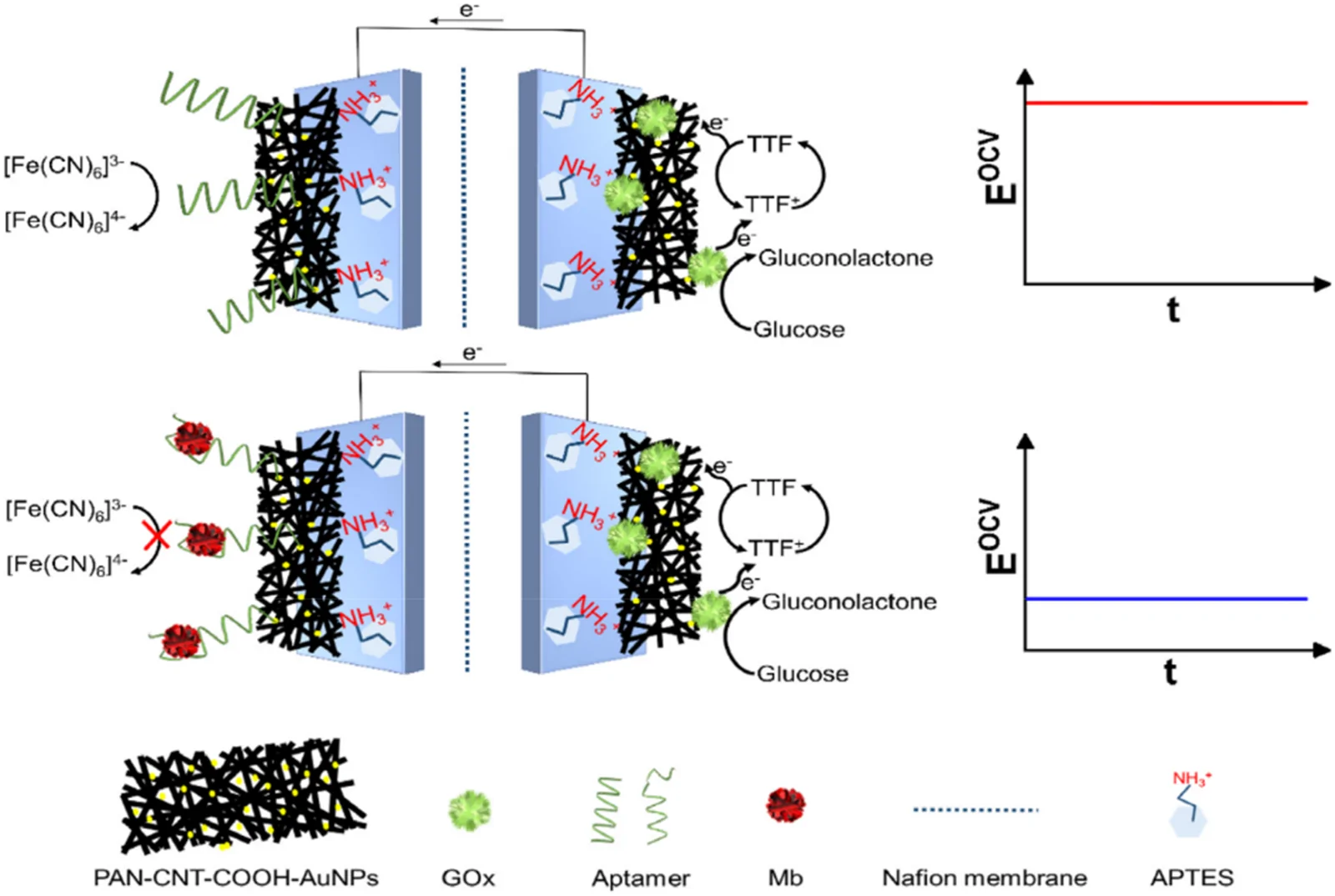
A representation of the foundational concept of the self-powered method for Mb detection [546]. Copyright 2025. Reproduced with permission from Elsevier.

**Fig. 64:**
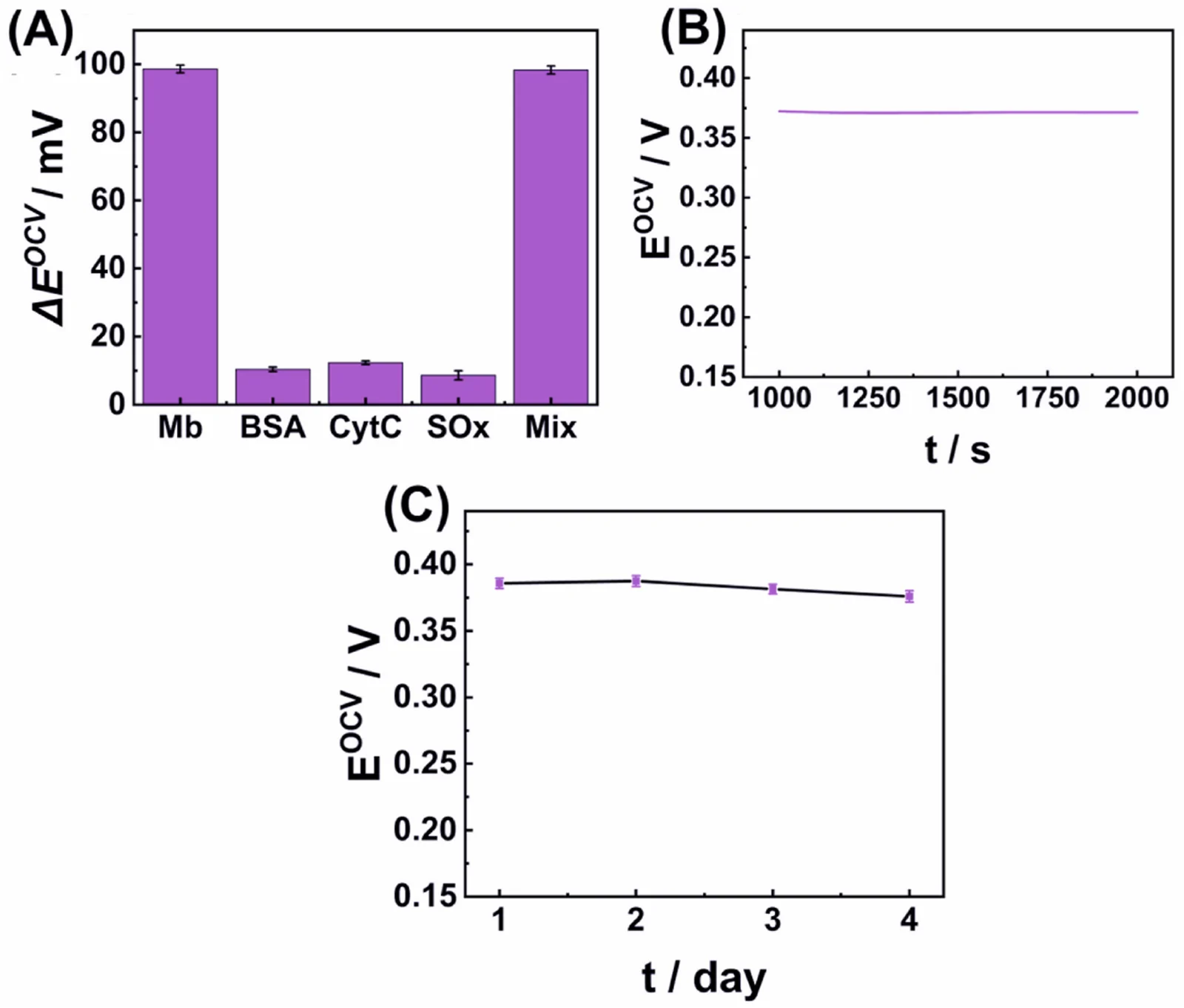
(A) Selectivity analysis of the self-powered biosensor, with error bars indicating the standard deviation of three independent measurements. (B) Long-term operational stability of the biosensor monitored over 2000 s. (C) Stability evaluation of the assembled biosensor over time [546]. Copyright 2025. Reproduced with permission from Elsevier.

Ozoemena et al. designed an onion-like carbon (OLC)-integrated PAN NFs to enhance spinnability and conductivity for DA detection [551]. Following carbonization, the OLC-carbon nanofibers exhibited an optimized pore structure and increased surface area, enabling efficient DA adsorption and redox cycling. The sensor achieved a sensitivity of 0.31 μA μM^−1^ and a LOD of 1.42 μM, demonstrating that polymer blending and hybrid carbon architectures can provide adaptable sensing platforms [551]. In another study, Ghorbanizamani et al. developed an electrochemical immunosensor by incorporating aminated MXene nanosheets into polypropylene carbonate (PPC) NFs, which were electrospun directly onto gold screen-printed electrodes, forming a high-surface-area, conductive, and antibody-compatible interface for the detection of synthetic cannabinoids [552]. The MXene-NH_2_/PPC NFs exhibited a cracked, flake-like morphology, which enhanced electron transfer and facilitated the efficient immobilization of monoclonal anti-K2 antibodies. Optimizing solvent composition, polymer concentration, MXene loading, and ES time yielded a uniform nanofibrous network with increased electroactive surface area and reduced charge-transfer resistance. Using differential pulse voltammetry (DPV), the immunosensor demonstrated a suitably wide linear range of 0.6-2000 ng mL^−1^ for JWH-018 and a LOD of 0.66 ng mL^−1^ in both PBS and saliva samples. The sensor also exhibited excellent repeatability (CV = 3.66%), reproducibility (CV = 1.25%), high recovery (∼96%), strong selectivity, and long-term stability under vacuum storage conditions [552].

Beyond biomolecular recognition, ESNFs have been combined with molecularly imprinted polymers (MIPs) to mimic natural receptor-ligand interactions. Compared with biological recognition elements, MIPs offer higher mechanical stability, lower cost, and resistance to harsh conditions; however, challenges such as binding heterogeneity and limited accessibility of recognition sites remain [553]. Incorporating MIPs into nanofibrous architectures mitigates these limitations by increasing the surface-to-volume ratio and improving site accessibility. MIP-nanofiber hybrids can be fabricated via several strategies, including (i) coating preformed NFs with an imprinted layer, (ii) surface imprinting on fibers, (iii) embedding MIP NPs within fibrous networks, and (iv) direct imprinting during ES [553]. For example, Yang et al. created a visible-light-responsive photoelectrochemical (PEC) sensor by integrating ES template-directed molecularly imprinted NFs (MI-ESNFs) with BiOI nanoflake arrays, forming a hierarchical MI-ESNFs@BiOI photoactive electrode [554]. The MIP NFs were prepared by ES using a CS/triphenyl phosphate (CS/TPhP) precursor with TPhP serving as the template. After ES, neutralization, and template removal, the resulting MI-ESNFs contained well-defined recognition cavities complementary to TPhP in size, shape, and functional groups. SEM analysis confirmed the presence of uniform fibers (∼50 nm) intertwined with BiOI nanoflakes, forming a 3D interconnected network that enhanced mass transport and provided abundant active sites. The sensing mechanism is assumed to be that rebinding of TPhP to the imprinted cavities induces steric hindrance, inhibiting the diffusion of electron donor (e.g. AA) and thereby decreasing photocurrent under visible-light irradiation. The resulting MI-ESNFs@BiOI electrode exhibited a linear response from 0.01 to 500 ng mL^−1^ and an ultralow LOD of 8.0 fg mL^−1^, outperforming conventional chromatographic methods. The sensor also showed excellent selectivity toward structurally related organophosphate flame retardants (OPFRs), as well as high reproducibility and long-term stability [554].

Enzyme-based assays remain widely used for early disease biomarker detection; however, they are often lengthy, costly, and require specialized laboratory instruments, limiting their application in underdeveloped settings. Accordingly, an increasing need for rapid, compatible, and standardized sensing platforms capable of detecting cancer biomarkers at ultralow concentrations. In this perspective, Tawfik et al. proposed an enzyme-free, cheap assay based on fluorescent molecularly imprinted conjugated polythiophenes (FMICPs) for cancer biomarker detection [555]. Leveraging the high interfacial advantage of NF networks, FMICP-based NFs enabled rapid detection of alpha-fetoprotein (AFP) and carcinoembryonic antigen (CEA) within 15 min. The nanofibrous structure enhanced sensitivity by approximately 80-fold compared with pristine FMICP, achieving LODs of 15 fg mL^−1^ (AFP) and 3.5 fg mL^−1^ (CEA), which are several-fold better than those of traditional enzyme-based counterparts. The sensor also demonstrated successful application in clinical liver cancer samples, with results consistent with standard ELISA measurements [555].

Hu et al. fabricated SnO_2_-doped carbon nanofibers via ES of PAN/SnCl_2_ precursors followed by stabilization and carbonization, producing uniform fibers (400-500 nm) with well-dispersed SnO_2_ confirmed by SEM, TEM, EDS, XRD, and XPS [556]. The modified GCE exhibited reduced charge-transfer resistance and enhanced electrocatalytic activity toward p-hydroxyacetophenone (p-HAP) and acetaminophen (APAP). Using DPV, the biosensor showed linear ranges of 0.5-700 μM (APAP) and 0.2-50 μM (p-HAP), with low LODs of 86 nM and 33 nM, respectively. The platform also demonstrated high selectivity, reproducibility, and effectiveness in pharmaceutical and serum samples [556]. Bahrami et al. designed a magnetic nanofiber-based sensor for morphine recognition using electrospun and thermally treated PVA/iron salt precursors, yielding fibers with diameters of 70-120 nm [557]. The resulting composite was mixed with graphite powder and paraffin oil to form a carbon paste electrode (CPE). Structural characterization (FTIR, SEM, and XRD) confirmed material integrity. Electrochemical analysis via CV and DPV revealed a wide linear range (3.3 nM-245 μM) and a LOD of 1.9 nM, demonstrating high sensitivity and reliability [557].

ESNFs have also been widely explored for heavy metal ion sensing. Ehzari et al. developed an aptasensor for Hg^2+^ detection by integrating electrospun polyethersulfone NFs decorated with CdTe QDs (NFs-QDs) onto a CPE, forming a highly porous electroactive platform that enhanced methylene blue (MB) signal amplification [558]. The sensing mechanism relies on specific T-Hg^2+^-T coordination, along with additional MB-Hg^2+^ interactions that increase binding site availability and signal intensity. The composite NFs improved electron transfer kinetics and provided abundant immobilization sites for amine-terminated DNA aptamers. The aptasensor exhibited a wide detection range (0.1-150 nM) and an ultralow LOD of 0.02 nM, with high selectivity against interfering metal ions, excellent reproducibility (RSD 2-3%), and successful application in spiked fruit juice samples [558].

Colorimetric sensing has also benefited from nanofibrous architectures. For instance, a reusable optical sensor based on polyaspartate (PASP) ESNF hydrogel membranes (ENHMs) was developed for the determination of Cu^2+^ and Fe^3+^ in aqueous media [559]. The PASP-ENHM sensor demonstrated tremendous sensitivity and selectivity, with visible color changes detectable by the naked eye: white to blue for Cu^2+^ (LOD = 0.3 mg L^−1^) and white to yellow for Fe^3+^ (LOD = 0.1 mg L^−1^). Desorption processes were also applicable, enabling the reusability of the biosensing platform [559]. Similarly, Tungsombatvisit et al. developed CA nanofiber-based rhodamine B derivative (RBD-CA NF) colorimetric strip for the detection of Cu^2+^ [560]. Alkaline treatment (0.1 M NaOH) improved wettability and reduced response time from 3300 s to 100 s. Consequently, the sensor demonstrated a detection range of 20-300 ppm, a detection limit of 18 ppm, and optimal performance at pH 6 [560].

In another work, novel Mn_2_O_3_-Ag NFs were fabricated via a facile 2-step process involving ES followed by calcination. An amperometric glucose biosensor was created by binding GOx onto highly porous Mn_2_O_3_-Ag NFs [561]. The resulting composite NFs exhibited a rough, nanoparticle-assembled morphology with a high surface-to-volume ratio, abundant catalytic sites, and superior electrical properties. Electrochemical studies of the Mn_2_O_3_-Ag nanofiber-GOx modified electrode indicated that the highly porous nanofibrous structure provides a favorable microenvironment for enzyme immobilization, stabilizes biological activity, and significantly enhances direct electron transfer (DET). This enhancement arises from the large specific interface, good biocompatibility, and the presence of multiple effective electron passage pathways within the network. The observed DET also suggests close electronic coupling between GOx and the NFs, enabling rapid monitoring of glucose-induced oxygen consumption without significant diffusion-related signal loss. When integrated onto GCE, the Mn_2_O_3_-Ag nanofiber matrix enabled efficient electron passage between the flavin adenine dinucleotide (FAD/FADH_2_) redox center of GOx and the electrode surface. Operating via an oxygen-reduction transduction mechanism, the biosensor exhibited a fast response time (5-10 s), a high sensitivity of 40.60 μA mM^−1^ cm^−2^, and a low LOD of 1.73 μM. It also showed a low apparent Michaelis-Menten constant (2.1 mM), indicating strong enzyme-substrate affinity, along with excellent selectivity against common interferents such as UA and AA. Overall, these results demonstrate that Mn_2_O_3_-Ag nanofiber-GOx composites are promising platforms for highly sensitive and selective electrochemical glucose sensing based on oxygen reduction [561].

In another study, Li et al. proposed a sensitive, label-free amperometric immunosensor for AFP using electrospun IrO_x_ NFs engineered into a wire-in-tube architecture [562]. The NFs were synthesized by ES of IrCl_3_/PVP precursors, followed by controlled annealing, during which calcination at 500 °C produced an optimal Ir/IrO_2_ mixed-phase structure with increased conductivity, large electroactive interface, and outstanding chemical stability. The resulting IrO_x_ NFs were dispersed in CS and drop-cast onto GCE, enabling efficient attachment of anti-AFP mAb and formation of a stable immunointerface. Stepwise CV and EIS confirmed successful assembly and showed that the wire-in-tube morphology significantly accelerated electron transfer. Under optimized conditions, the immunosensor exhibited a wide detection range between 50 pg mL^−1^ and 150 ng mL^−1^ and an ultralow LOD (20 pg mL^−1^), outperforming many noble-metal-based AFP sensors. The platform also demonstrated strong selectivity, reproducibility, stability, and reliable performance in human serum [562].

Filiz et al. developed crosslinked CS/PVA ESNFs as a stable and biocompatible platform for naked-eye glucose sensing, using a GOx/HRP cascade with o-dianisidine as the chromogenic substrate [563]. The optimized CS/PVA (1:4) blend produced uniform, bead-free NFs (∼378 nm) with high hydrophilicity and mechanical stability, enabling efficient enzyme immobilization and robust operation in aqueous environments. The glucose detection range of 2.7-13.8 mM covers clinically relevant physiological levels, including hypoglycemic, normal fasting, and diabetic conditions [564], [565]. Fig. 65 illustrates spectrophotometric and strip-based validation of the sensing platform [563]. In Fig. 65a, UV-Vis spectra show a monotonic improvement in absorbance at 480 nm with increasing glucose amounts, corresponding to HRP-mediated oxidation of o-dianisidine driven by GOx-generated H_2_O_2_. Fig. 65b presents the calibration curve (R^2^ = 0.9968), confirming excellent linearity across the physiological range. Fig. 65c shows the visual response of the nanofiber strip, which changes from white to brownish-orange as glucose concentration increases, reflecting the enzymatic cascade reaction. Fig. 65d illustrates the underlying mechanism: glucose oxidation by GOx generates H_2_O_2_, which is immediately consumed by HRP to oxidize o-dianisidine. This coupled cascade accelerates reaction kinetics and minimizes GOx deactivation by preventing H_2_O_2_ accumulation. Overall, the study demonstrates that CS/PVA nanofiber strips operate effectively within the clinically relevant glucose detection window reported for both electrochemical and colorimetric systems (typically 0.001-13 mM) [566], [567], while offering a low-cost, equipment-free, and visually interpretable alternative for glucose monitoring [563].

**Fig. 65:**
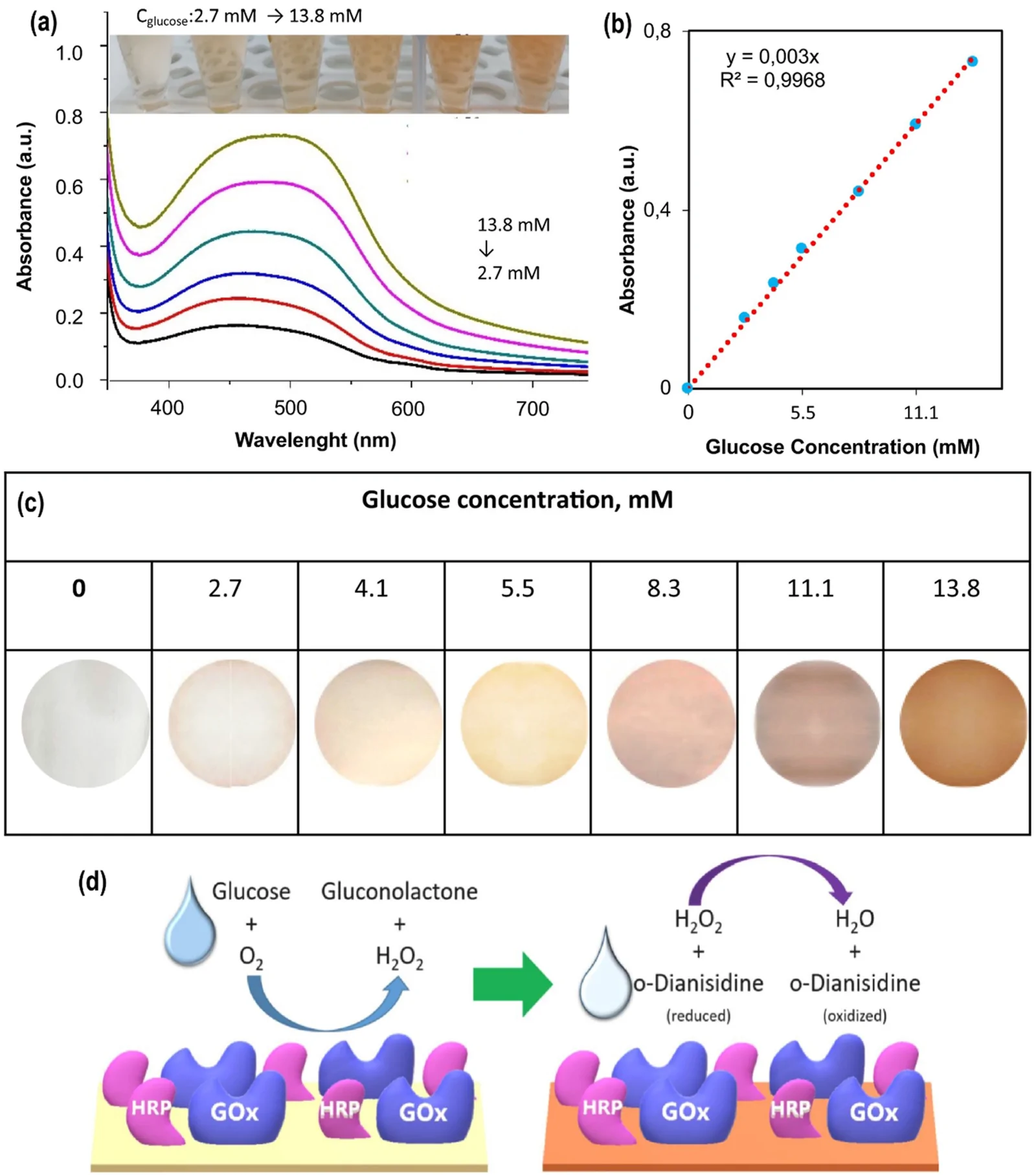
(a) UV-Vis spectra of oxidized o-dianisidine at different glucose concentrations in the presence of GOx and HRP. (b) Corresponding calibration curve for glucose determination. (c) Visual color changes of the C-CS/PVA-2 NFs at different glucose concentrations. (d) Schematic representation of the bi-enzymatic glucose sensing mechanism [563]. Copyright 2021. Reproduced with permission from Elsevier.

Wang et al. designed an ESNF-based electrochemiluminescent (ECL) immunosensor for the detection of the tumor suppressor protein p53 (TSP53) by integrating functional NFs with luminescent core-sheath nanocomposites [568]. Multi-walled CNTs (MWCNTs)-doped CS NFs (MWCNTs-CTS) were fabricated via a single-step ES and subsequently decorated in situ with AuNPs by electrodeposition, forming MWCNTs-CTS-AuNP structure for immobilization of capture antibodies (C_Ab_). These NFs provided enhanced loading capacity and improved stability for immobilized biomolecules. Meanwhile, Ru(bpy)_3_^2+^- and silver-doped silica NPs (RuAg/SiO_2_ NPs) were created via a water-in-oil microemulsion approach and used as highly luminescent labels for detection antibodies (D_Ab_). During the sandwich immunoassay, C_Ab_-functionalized NFs captured TSP53, while RuAg/SiO_2_ NPs@D_Ab_ conjugates acted as signal probes. The resulting electrode exhibited strong ECL emission intensity due to the synergistic electron-transfer efficiency between the nanofibrous network and the luminescent nanocomposites. As shown in Fig. 66a [568], the fabrication strategy integrates ES, electrodeposition, and antibody conjugation. Selectivity was evaluated using TSP53, hemoglobin, HSA, AFP, and mixed protein samples under identical conditions. As shown in Fig. 66b, only TSP53 produced a significant ECL signal, while the rest exhibited responses comparable to the blank, confirming high selectivity. In addition, the mixed-protein sample generated an ECL response similar to that of pure TSP53, further demonstrating the sensor's strong specificity. The ECL responses of various C_Ab_-TSP53-RuAg/SiO_2_ NPs@D_Ab_ combinations are demonstrated in Fig. 66c [568]. The calibration curve revealed a detection range between 1.0 pg mL^−1^ and 1.0 ng mL^−1^ (R^2^ = 0.9993) and a LOD of 0.5 pg mL^−1^. These results emphasize the ultrahigh sensitivity, excellent selectivity, and strong reproducibility of the developed ECL immunosensor.

**Fig. 66:**
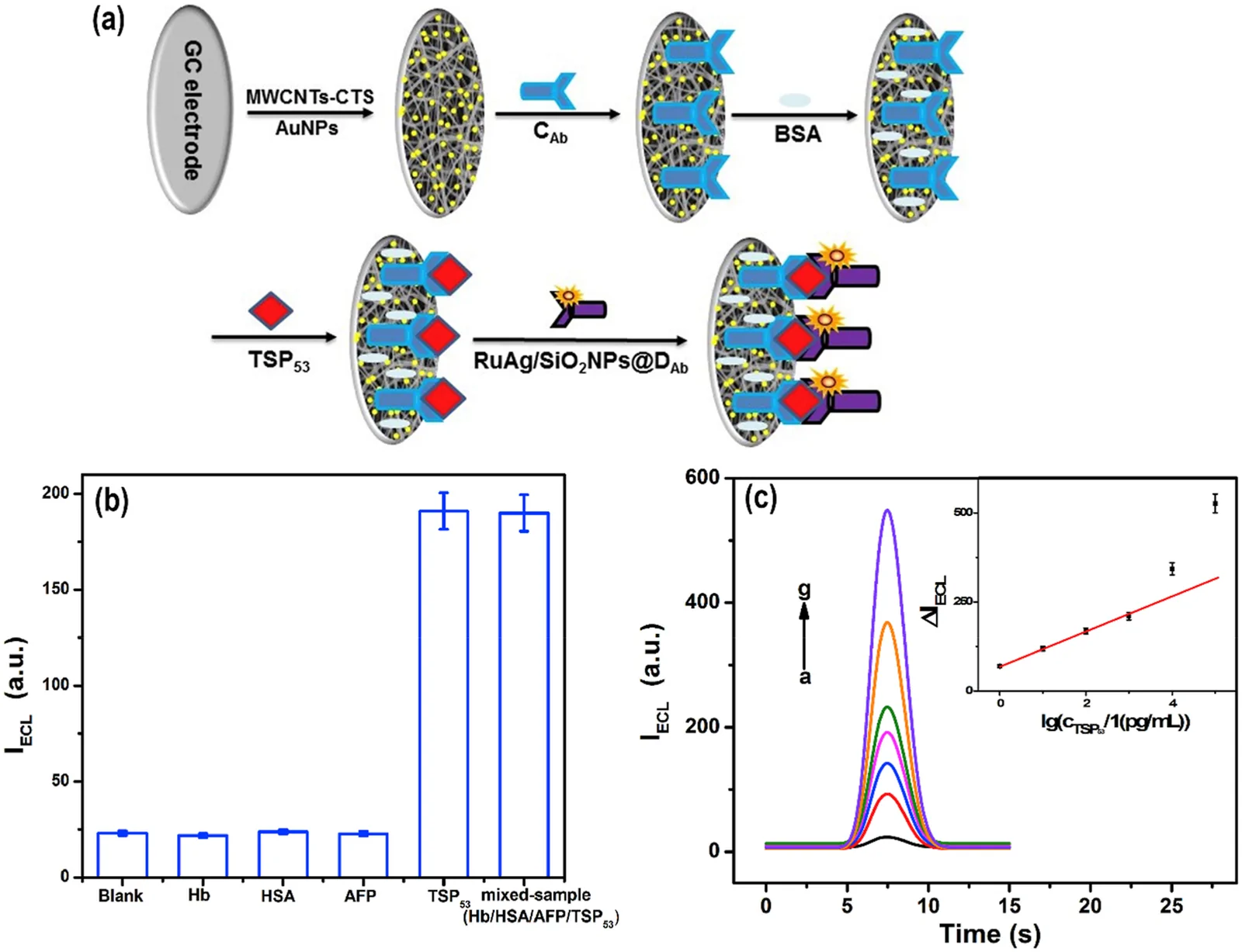
(a) Schematic of the fabrication process of the ESNF-based ECL immunosensor. (b) Comparison of the ECL intensity (I_ECL) of the C_Ab_ electrode upon exposure to TSP53, Hb, HSA, AFP, and a mixed sample (Hb/HSA/AFP/TSP53) at a concentration of 0.1 ng/mL (c) ECL response toward TSP53 at different concentrations ranging from 100 ng mL^−1^ to 1 pg mL^−1^. Inset: calibration curve showing linearity between 1 pg mL^−1^ and 100 ng mL^−1^. ECL measurements were performed in 10 mM PBS containing 1.0 mM TPrA (pH 7.4) under CV with a potential window of 0.0-1.2 V and a scan rate of 100 mV s^−1^[568]. Copyright 2018. Reproduced with permission from Elsevier.

Wang et al. fabricated water-stable electrospun PVA nanofibrous mats decorated with uniformly distributed AuNPs, creating an efficient substrate for HRP-based hydrogen peroxide (H_2_O_2_) sensing [569]. An in-situ glutaraldehyde crosslinking method was applied to produce mechanically robust, water-insoluble PVA NFs. At the same time, MPTES functionalization enabled dense Au-S bonding on the NFs, thereby enhancing both conductivity and enzyme loading. As illustrated in Fig. 67, the sensing mechanism relied on the HRP-catalyzed reduction of H_2_O_2_, with hydroquinone (HQ) serving as an electron mediator. In this cycle, HRP is oxidized by H_2_O_2_, while HQ reduces the oxidized enzyme. Subsequently, benzoquinone is reduced back to HQ, sustaining continuous electron transfer. This catalytic cycle generates a reduction current relative to the H_2_O_2_ amounts. The AuNP-PVA nanofibrous network significantly amplifies the electrochemical response by providing a large interfacial area and facilitating rapid electron transfer, resulting in a fast response time, a wide detection range (1.0-500 μM), and an adequate LOD (0.5 μM). Compared with other HRP-based sensing platforms, this nanofibrous system demonstrates both an extended dynamic range and rapid response attributed to the efficient diffusion of H_2_O_2_ through the porous AuNP-PVA matrix [569].

**Fig. 67:**
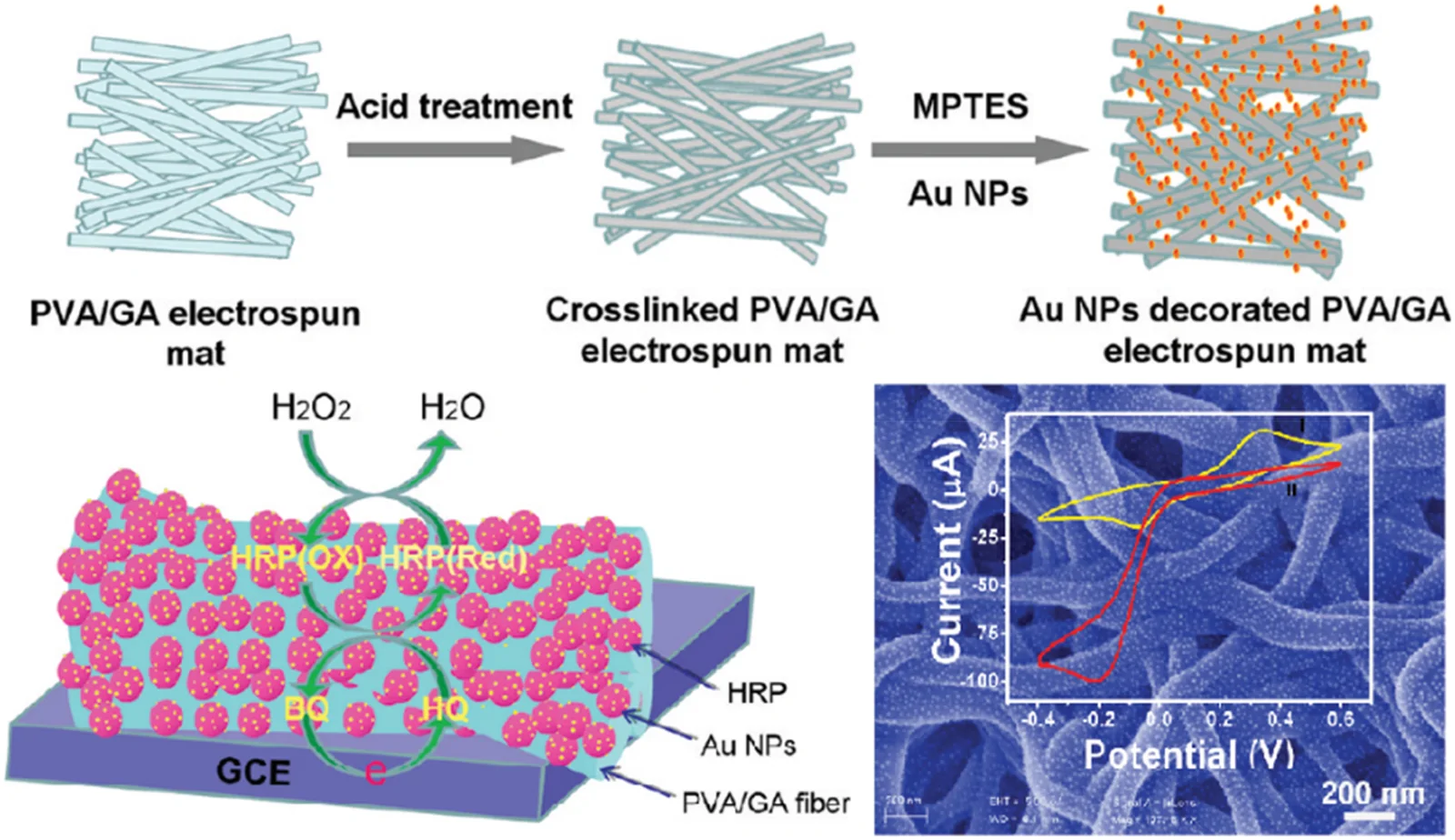
Illustration of the fabrication steps of AuNPs decorated PVA/GA electrospun mat, and the corresponding H_2_O_2_ biosensor based on the HRP/AuNPs-PVA-modified GCE [569]. Copyright 2012. Reproduced with permission from the American Chemical Society.

A nanofibrous biosensor based on burkholderia cepacia lipase (BCL) immobilized on MOF structures was fabricated using metal-organic NFs (MONFs) synthesized from amino acid and copper nitrate precursors [570]. These MONFs provided high electrochemical activity and chemical robustness, enabling sensitive detection of methyl parathion residues in vegetables. The NFs were integrated with CS as a binder to immobilize BCL@MOF composites onto GCE, resulting in a biosensor with a low LOD of 67 nM and a linear detection range of 0.1-38 μM. The system displayed good selectivity and working stability, highlighting its potential for pesticide monitoring in food matrices [570]. In another investigation, Alim et al. developed a glucose biosensor based on electrospun multiporous SnO_2_ NFs fabricated from tin-based precursors [571]. The porous nanofibrous architecture facilitated efficient electron transfer between the electrode and the redox-active GOx cluster, thereby enhancing electrochemical communication and catalytic performance. The resulting biosensor displayed a linear amperometric response over 5.0-100 μM, with a LOD of 1.8 μM. In addition, the sensor demonstrated excellent reproducibility and long-term stability, attributed to the ample surface area and excellent conductivity of the SnO_2_ nanofiber matrix [571].

Similarly, Chauhan et al. reported PAN NFs embedded with magnetic Fe_3_O_4_ NPs via ES to form Fe_3_O_4_-PAN NFs composites [572]. Nafion was used to improve the adhesion of the NFs onto an ITO substrate. The Fe_3_O_4_-PAN NFs/ITO electrode was further functionalized with NaOH to partially convert the nitrile groups in PAN to -COOH and -NH_2_ groups, providing a suitable microenvironment for anti-vitamin D (Anti-VD) immobilization. BSA was used to block non-specific binding sites on the Anti-VD/Fe_3_O_4_-PAN NFs/ITO immunoelectrode. The electrochemical response of the BSA/Anti-VD/Fe_3_O_4_-PAN NFs/ITO immunoelectrode toward vitamin D_3_ (Vit-D_3_) was investigated using DPV between 10 ng mL^−1^ and 100 ng mL^−1^, as shown in Fig. 68. During measurement, the immunoelectrode was incubated with Vit-D_3_ for approximately 5 min, and a linear increase in signal was observed up to 90 ng mL^−1^, followed by saturation at higher concentrations (Fig. 68a). This increase in current is attributed to the formation of an immunocomplex (antigen-antibody) at the electrode surface, which facilitates electron passage [572]. Fig. 68b shows the calibration curve of Vit-D_3_, yielding a sensitivity of 0.90 μA ng^−1^ mL cm^−2^ and a correlation coefficient (R^2^) of 0.98. The LOD was estimated around 0.12 ng mL^−1^. Furthermore, a Hanes-Woolf plot (Vit-D_3_ concentration vs. Vit-D_3_ concentration/peak current), shown in Fig. 68c, was used to determine the association (K_a_) and dissociation (K_d_) constants, reflecting the kinetics of immunocomplex formation at the electrode surface. The Ka value (74 ng mL^−1^) was obtained from the reciprocal of the slope, while the Kd value (4.5 ng mL^−1^) was derived from the product of Ka and the intercept. These results indicate that Fe_3_O_4_ NPs within the PAN NFs provide abundant binding sites for antibody immobilization, thereby significantly enhancing biosensing performance [572].

**Fig. 68:**
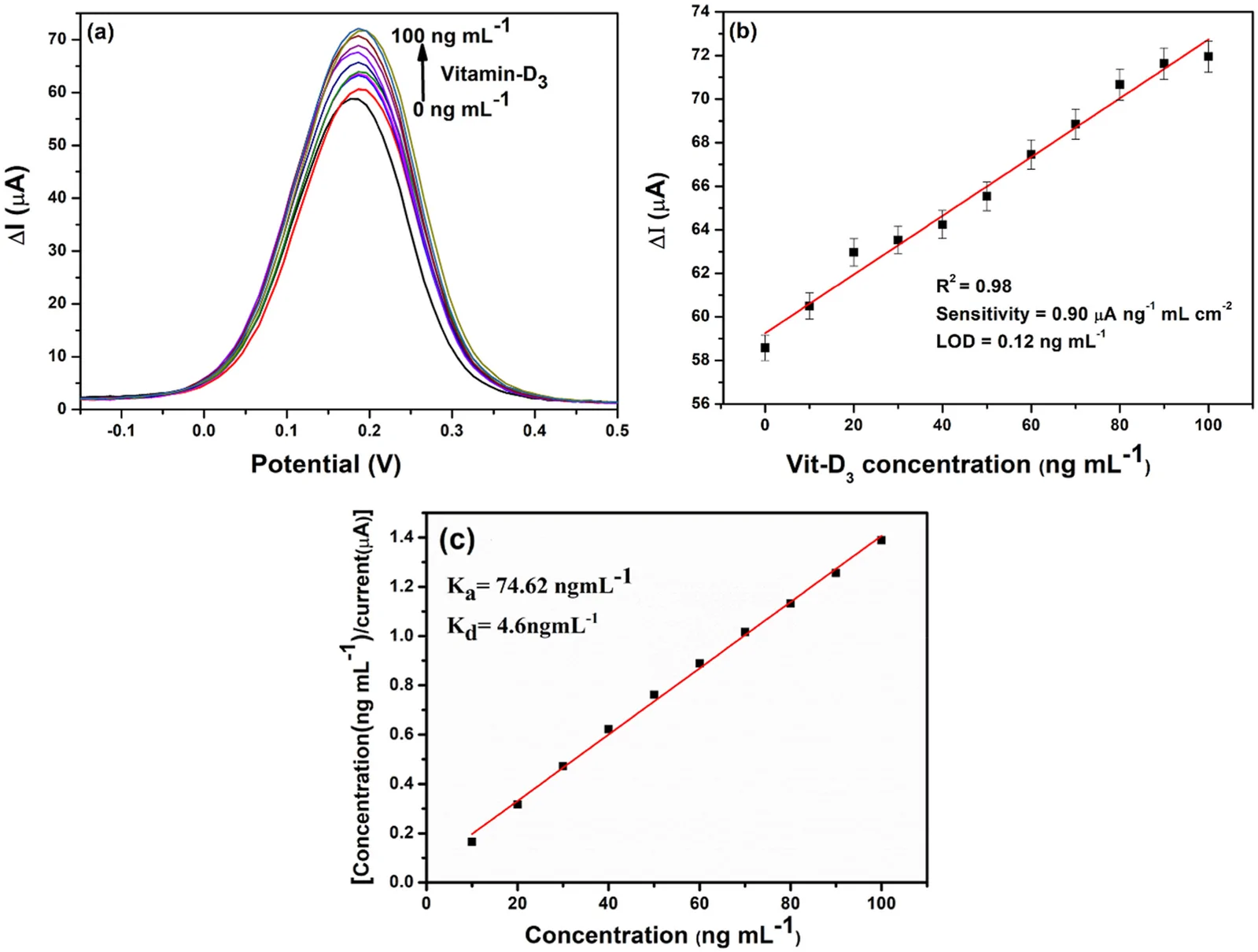
(a) DPV response of the immunoelectrode (BSA/Anti-VD/Fe_3_O_4_-PAN NFs/ITO) toward different concentrations of vitamin D_3_ (Vit-D_3_) antigen. (b) Calibration plot showing the relationship between DPV signals and Vit-D_3_ concentration. (c) Hanes-Woolf plot of Vit-D_3_ concentration versus (concentration/peak current) used for kinetic analysis of the immunoreaction [572]. Copyright 2018. Reproduced with Permission from Elsevier.

Another notable example of metal-based nanofibrous architectures involves hierarchical NiCo_2_S_4_ nanowire arrays grown on electrospun graphite fibers (EGF), as shown in Fig. 69, for glucose biosensing [573]. The EGF substrate was prepared via direct graphitization of electrospun polymeric fibers, followed by hydrothermal growth of NiCo_2_O_4_ precursors using Ni^2+^/Co^2+^ salts and urea. A subsequent sulfurization step with Na_2_S converted the oxide into NiCo_2_S_4_, forming an interwoven 3D nanowire architecture. The resulting NiCo_2_S_4_/EGF composite exhibited excellent mechanical stability and abundant electroactive sites due to its hierarchical core-sheath morphology and high surface area. These structural advantages are directly reflected in the amperometric behavior summarized in Fig. 70 [573]. Fig. 70A shows the current-time (I-t) response at low glucose concentrations, where successive additions down to the sub-micromolar level produce immediate and well-defined current steps. The sensor reaches a steady state within 5 s, indicating rapid catalytic kinetics. Fig. 70B extends the response to higher concentrations, demonstrating a wide detection range from 0.5 μM to 3.57 mM, with a proportional increase in current. The inset calibration plot confirms excellent linearity (R^2^ = 0.995) and a low LOD of 0.167 μM [573]. Comparative analysis shows that the NiCo_2_S_4_/EGF-7 configuration outperforms or matches related systems, including NiCo_2_S_4_/Pt [574], NiCo_2_S_4_/GCE [575], and NiCoO_2_@CNT [576]. The superior electrochemical performance of NiCo_2_S_4_/EGF-7 is attributed to three synergistic factors: (i) the core-sheath NiCo_2_S_4_ nanowire structure providing abundant active sites for glucose oxidation; (ii) strong coupling between NiCo_2_S_4_ and the conductive EGF backbone, which enhances electron transport and catalytic kinetics; and (iii) uniform nanowire dispersion on the fiber network, which prevents agglomeration and maximizes accessible surface area for charge transfer [573]. Furthermore, interference studies using AA, DA, UA, and common sugars (lactose, xylose, mannose) demonstrated high selectivity, with interference signals below 5% (Fig. 70C) [573]. Operational stability tests showed that the sensor lost 13.8% of its first response after 4600 s of continuous operation, while also exhibiting good storage stability (Fig. 70D) [573]. Overall, these characteristics, combined with rapid response time and enzyme-free selectivity, establish NiCo_2_S_4_/EGF-7 as an efficient platform for glucose sensing within physiologically relevant ranges (3.0-8.0 mM in human serum) [577]. In addition to these examples, the literature in this field is extensive, and Table 6 summarizes selected reports on nanofiber-based electrochemical glucose sensors.

**Fig. 69:**
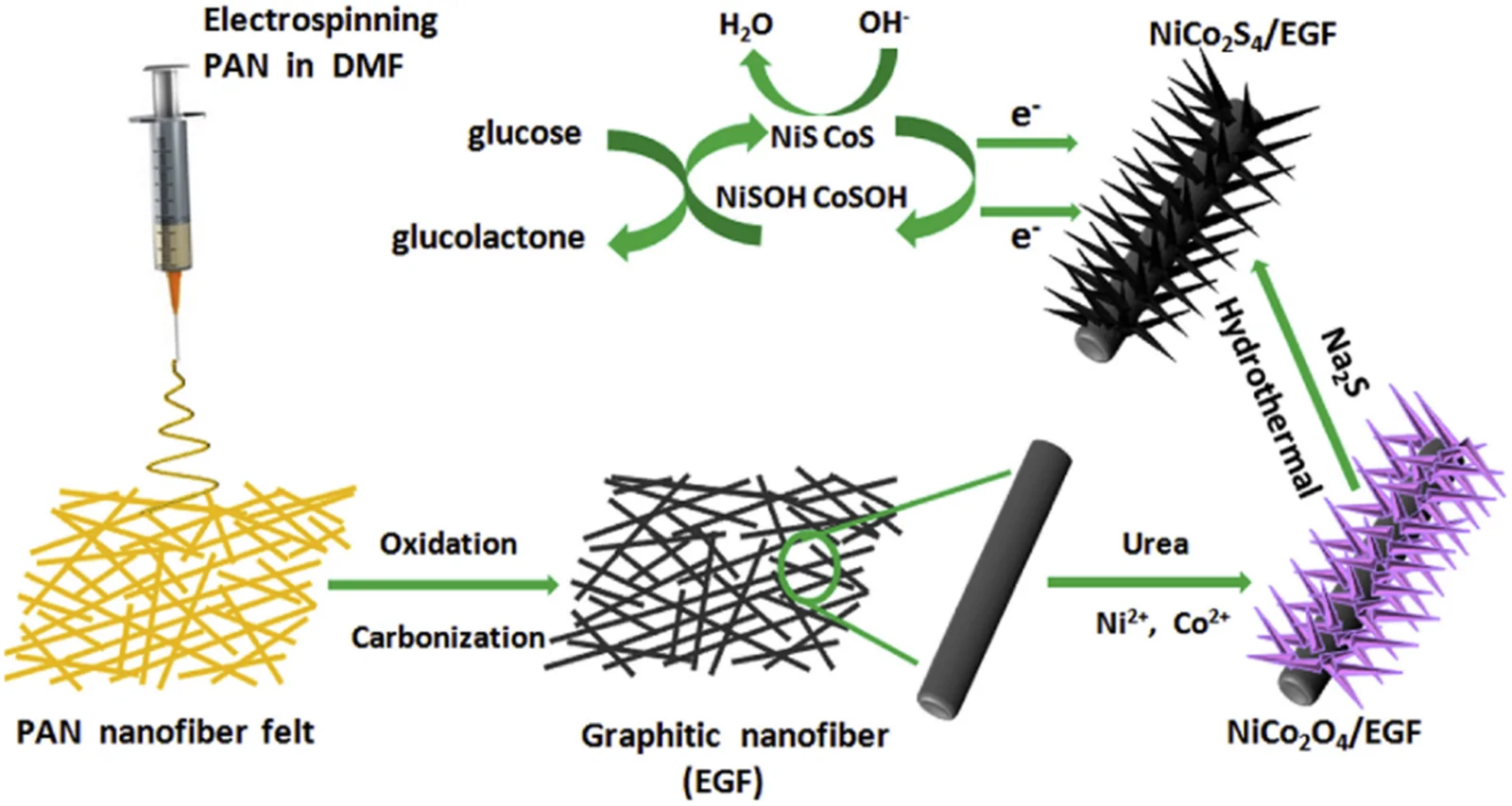
The approaches for the fabrication of NiCo_2_S_4_/EGF [573]. Copyright 2020. Reproduced with permission from Elsevier.

**Fig. 70:**
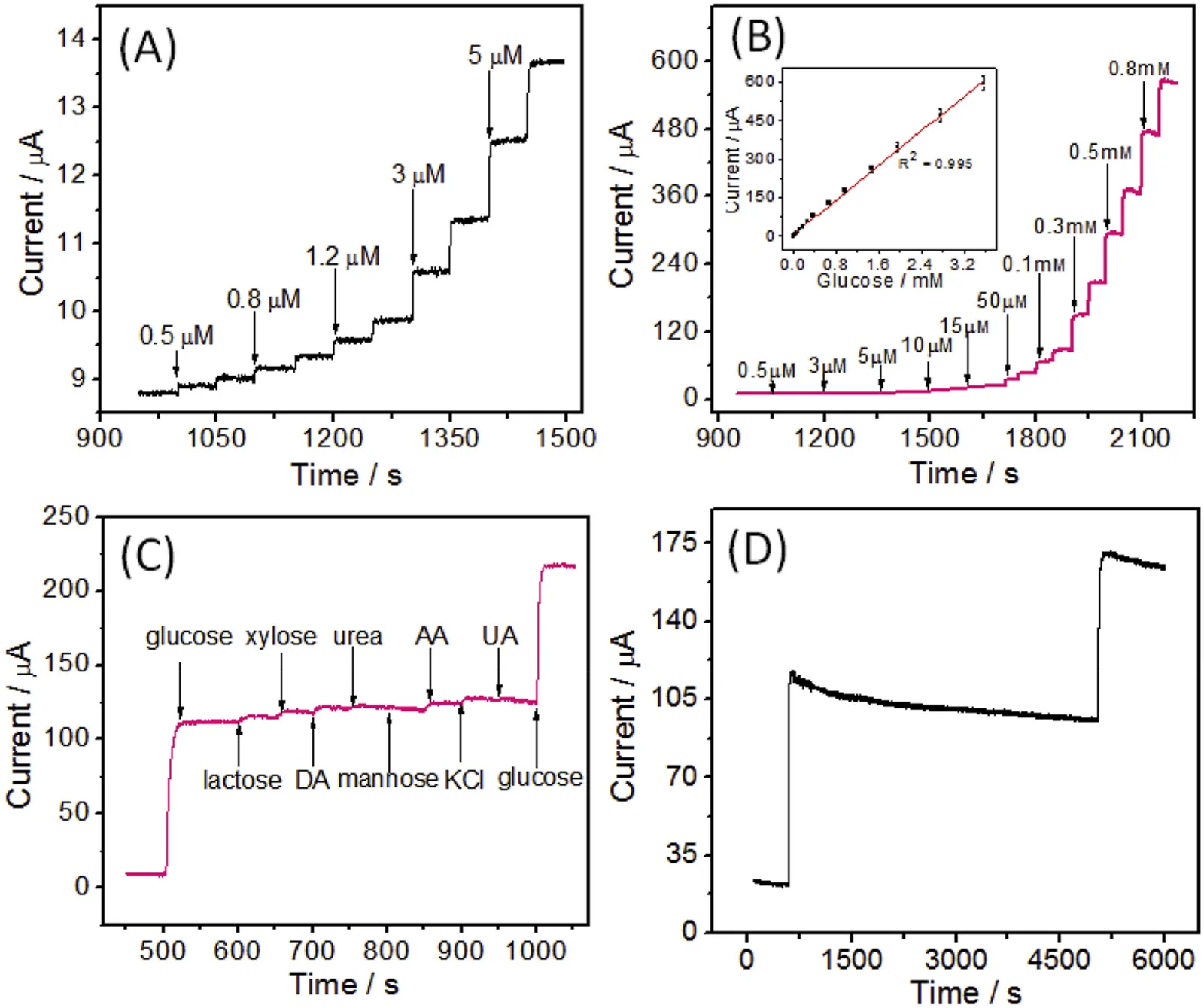
Amperometric performance and robustness of the NiCo_2_S_4_/EGF-7 glucose sensor. (A) Sensor response upon successive additions of low glucose concentrations. (B) Amperometric response across the full concentration range with corresponding linear calibration inset. (C) Selectivity assessment against common interferents. (D) Long-term operational stability assessment [573]. Copyright 2020. Reproduced with permission from Elsevier.

**Table 6:** Electrochemical glucose sensors based on ESNFs.

Sensor	Linear Range	LOD	Ref.
PAN/PPy/PPy3COOH	20 nM - 2.0 M	2.0 nM	[466]
CNF@Ni-CoLDH	1.0 μM - 2.0 mM	0.03 μM	[538]
NiCo_2_O_4_/EGF	0.5 μM −3.57 mM	0.167 μM	[573]
Cu-nanoflower@AuNPs-GONFs	1.0 - 100 μM	18 mM	[578]
Ni/CoOCNF	0.25 - 600 M	0.03 μM	[579]
CA/CS	5.0 M − 0.75 mM	4.8 μM	[580]
CS/GO	0.05-20 mM	0.02 mM	[581]
PEI/PVA	2-8 mM, 10-30 mM	0.3 mM	[582]
SnO_2_ multiporous NFs	0.5-5.0 mM	0.05 mM	[583]
Cellulose/β-CD	0.0 - 1.0 mM	9.35 × 10^−5^ M	[584]

## 5 Challenges and future perspectives

Functionalized ESNFs have attracted considerable attention as advanced materials for biomedical applications owing to their tunable porosity, high surface-to-volume ratio, structural versatility, and ability to incorporate, immobilize, or spatially organize bioactive agents. These characteristics have enabled their investigation in DD, wound healing, TE, biosensing, and other therapeutic applications. Despite substantial progress, several challenges remain, including biocompatibility and potential cytotoxicity, controlled-release behavior, mechanical robustness, long-term functional stability, manufacturing scalability, reproducibility, and regulatory compliance. These limitations continue to hinder the broader clinical translation and industrial adoption of functionalized ESNFs. Beyond these challenges, however, recent advances in ES technique have substantially expanded the structural design space of ESNFs, creating new opportunities for the development of next-generation nanofibrous systems with precisely controlled architectures and functions.

### 5.1 Structural versatility and emerging electrospinning architectures

The development of nanoscale architectures represents an important manifestation of progress in nanoscience and nanotechnology [585]. ES, electrospraying, and electrohydrodynamic jetting/printing are representative electrohydrodynamic fabrication approaches that exploit interactions between working fluids and electrostatic forces to generate micro- and nanoscale structures [586], [587], [588], [589], [590]. Among these approaches, ES is particularly attractive because of its remarkable structural versatility and its ability to generate nanofibrous architectures with controlled composition, spatial organization, and compartmentalization. Depending on the configuration of the ES process and the properties of the working fluids, ES can produce conventional monolithic nanofibers as well as increasingly sophisticated structures, including core–sheath, hollow, Janus, multi-chamber Janus, and hybrid Janus/core–sheath nanofibers [586], [587], [588], [589], [590], [591], [592], [593], [594]. Monolithic nanofibers provide a relatively simple and versatile platform for biomedical applications [586], whereas core–sheath nanofibers enable spatial separation and controlled presentation of different materials, including therapeutic agents and polymers with distinct or gradient distributions [587], [588], [589]. Hollow nanofibers and surface-engineered nanofibrous structures provide additional control over internal morphology and interfacial characteristics [590], [591]. More recently, Janus nanofibers, multi-chamber Janus fibers, and hybrid Janus/core–sheath architectures have further expanded the structural and functional design space of ES [592], [593], [594]. These architectures provide multiple compartments or spatially distinct domains within a single nanofibrous structure, thereby enabling more precise control over drug loading, molecular distribution, interfacial interactions, sequential or differential release, and multifunctional performance [591], [595], [596], [597]. Such advances demonstrate that the progress of ES extends beyond the production of conventional nanoscale fibers toward the rational construction of complex nanostructures with programmed spatial organization and functionality. This structural versatility may be particularly valuable for biomedical applications in which therapeutic agents with different physicochemical properties, release requirements, or biological targets need to be incorporated into a single platform. Nevertheless, increasing structural complexity should be accompanied by careful consideration of fabrication reproducibility, characterization, stability, and manufacturing feasibility. Thus, future development should focus not simply on creating increasingly complex architectures, but on identifying those structural features that provide a clear functional or therapeutic advantage.

### 5.2 Scale-up, bulk production, and sustainable manufacturing

A critical barrier to the clinical translation and industrial adoption of functionalized ESNFs is the gap between laboratory-scale fabrication and large-scale manufacturing under Good Manufacturing Practice (GMP)-compatible conditions. Translating ESNF systems from laboratory studies to clinically relevant production requires standardized fabrication protocols, batch-to-batch reproducibility, rigorous quality control, and regulatory compliance, all of which can substantially increase process complexity and cost. Conventional needle-based ES provides precise control over fiber formation and functionalization but generally has relatively low productivity, limiting its suitability for large-scale manufacturing. In contrast, NLES, multinozzle systems, and other high-throughput ES approaches offer substantially greater productivity and therefore represent promising routes toward industrial production.

Recent studies have demonstrated the potential of bulk and batch production strategies to increase nanofiber productivity while maintaining appropriate structural and functional characteristics [598], [599]. Such developments are particularly important because industrialization requires not only an increase in fiber yield but also the preservation of critical quality attributes across larger production areas and longer processing periods. During scale-up, changes in electric-field distribution, fluid supply, environmental conditions, jet interactions, and solvent evaporation can influence fiber morphology, diameter distribution, porosity, drug loading, and functional-agent distribution. Consequently, maintaining structural and functional uniformity while increasing productivity remains a major technical challenge.

Future research should therefore place greater emphasis on scalable and continuous manufacturing strategies capable of preserving the sophisticated architectures achieved at laboratory scale. High-throughput electrospinning platforms, multinozzle and needleless systems, automated process control, and real-time monitoring of critical process parameters may facilitate more reproducible production. Integration of ES with established manufacturing technologies and complementary fabrication approaches may provide additional routes toward scalable production and accelerate the industrialization of nanofiber-based drug delivery systems [600]. Importantly, scale-up should be evaluated according to a comprehensive set of critical quality attributes, including fiber diameter and its distribution, porosity, surface chemistry, drug loading, functional-agent distribution, mechanical properties, degradation behavior, and release characteristics. Process analytical technologies and data-driven or machine-learning-assisted optimization may further facilitate the development of robust manufacturing processes.

Sustainability should also become an increasingly important consideration during process development. The large-scale use of organic solvents, energy-intensive processing, and extensive post-fabrication modification may increase the environmental burden of ESNF production. Accordingly, future studies should explore safer and more environmentally compatible solvents, aqueous or solvent-minimized processing where feasible, energy-efficient high-throughput systems, and manufacturing strategies that reduce material waste. Importantly, sustainability should be considered together with product quality, safety, and process performance rather than as an independent criterion. The development of scalable ESNF technologies should therefore aim to achieve an appropriate balance among productivity, structural precision, functional performance, environmental impact, and economic feasibility.

### 5.3 Functionalization trade-offs and design principles

The use of novel crosslinkers, solvents, grafting reagents, and functionalization agents introduces additional considerations regarding toxicity, residual chemicals, degradation products, long-term biocompatibility, and regulatory acceptability. Although functionalization can substantially improve surface bioactivity, drug loading, controlled release, or cell–material interactions, these benefits may be accompanied by changes in mechanical properties, degradation behavior, structural stability, and long-term functional durability. Chemical crosslinking and post-fabrication modification, for example, may improve aqueous stability or functional-agent retention but can introduce residual reagents, additional processing steps, heterogeneous modification, or changes in polymer degradation. Similarly, incorporation of multiple therapeutic or functional agents may enhance biological performance or enable sophisticated release profiles while increasing formulation complexity and potentially compromising reproducibility and stability. These trade-offs become particularly important during scale-up, where increasing material and process complexity may negatively affect batch-to-batch consistency, functional-agent distribution, sterilization, quality control, manufacturing cost, and regulatory evaluation.

Importantly, functionalization should therefore not be considered a universally beneficial modification. Each functionalization strategy should instead be evaluated according to its overall benefit–risk and complexity–performance balance. Increasing functional complexity is justified only when the resulting improvement provides a meaningful and reproducible therapeutic advantage. From a translational perspective, the optimal functionalized ESNF is unlikely to be the system with the highest degree of functionalization or structural complexity. Rather, it is likely to be the system in which each additional functional component or architectural feature provides a clearly demonstrated benefit while maintaining acceptable material stability, safety, manufacturability, and long-term performance.

Long-term stability and biological fate also require greater attention. Although numerous studies have demonstrated improved biological performance following the incorporation or immobilization of peptides, proteins, growth factors, drugs, and other bioactive molecules, relatively limited information is available regarding their long-term stability and preservation of bioactivity under physiological conditions. Changes in surface chemistry, functional-agent conformation, degradation products, and biological interactions over extended periods require systematic investigation. Future studies should therefore establish standardized long-term evaluation protocols and clarify the relationships among ESNF architecture, physicochemical properties, functional-agent presentation, biological response, degradation, and eventual in vivo fate.

### 5.4 Clinical translation and emerging research directions

Several electrospun biomedical systems have progressed beyond proof-of-concept research and provide useful benchmarks for understanding the factors that may favor clinical translation. One representative example is Restrata®, a synthetic electrospun hybrid-scale fiber matrix composed of polyglactin 910 and polydioxanone, which has received FDA 510(k) clearance for wound management. The use of clinically established, bioresorbable polymers with medical-device precedent, together with a relatively simple nonwoven fiber architecture and a clearly defined therapeutic purpose, has supported its clinical application [601]. Clinical studies have further provided evidence supporting the use of synthetic electrospun fiber matrices in wound management. Another notable example is Spincare™, a portable in situ ES system that directly deposits a nanofibrous matrix onto the wound. Spincare™ has progressed from preclinical development to clinical evaluation in patients with partial-thickness burns and has subsequently been investigated in chronic wound management [602]. These examples illustrate that electrospun systems based on well-characterized materials, relatively simple architectures, and clearly defined therapeutic functions may currently have greater translational potential than highly complex multifunctional systems [603].

Clinical and commercial experience with electrospun components in vascular and cardiovascular devices provides another important translational pathway [604], [605]. In particular, AVflo™ represents an example in which an electrospun nanofabric forms the principal functional structure of a vascular access graft, whereas PK Papyrus represents an alternative strategy in which an electrospun polyurethane layer is integrated into an established cardiovascular stent platform [604], [605]. These examples suggest that electrospinning may reach clinical application through more than one route: either as the primary structural component of a medical device or as a functional component incorporated into a clinically established platform. Although these clinically translated systems do not necessarily incorporate the full range of advanced functionalization strategies discussed in this review, they provide important translational benchmarks for understanding which characteristics of electrospun biomedical systems have already demonstrated clinical feasibility.

From our perspective, the functionalization strategies most likely to achieve clinical translation are those that combine meaningful therapeutic benefits with manufacturing simplicity, reproducibility, and a well-understood safety profile. In particular, surface functionalization using clinically relevant biomolecules, incorporation of established therapeutic agents into well-characterized polymer systems, and relatively simple core–sheath or surface-engineered architectures appear to offer promising translational opportunities. Such strategies can provide controlled presentation or release of therapeutic agents while limiting the number of novel materials and processing steps requiring extensive safety and regulatory evaluation.

Stimuli-responsive, multifunctional, and personalized ESNF systems also represent promising longer-term research directions. However, their clinical potential will depend on demonstrating predictable responses under physiologically relevant conditions, long-term stability, reproducible manufacturing, and adequate control of inter-batch variability. Similarly, complex hierarchical architectures, multiple functionalization agents, poorly characterized crosslinkers, extensive post-fabrication modification, and approaches requiring substantial quantities of non-biocompatible solvents or unstable biological components may face greater barriers to clinical translation. Although these systems can achieve sophisticated functions at the laboratory scale, their increasing structural and compositional complexity may complicate quality control, sterilization, stability assessment, manufacturing, and regulatory evaluation.

Accordingly, future research should increasingly move from proof-of-concept demonstrations toward application-oriented design criteria. Studies should define the specific therapeutic problem to be addressed, identify the minimum level of structural or functional complexity required to solve that problem, and evaluate the resulting system using clinically relevant performance metrics. Standardized characterization protocols and performance benchmarks will also be essential for enabling meaningful comparisons among polymers, functionalization strategies, architectures, and fabrication platforms. Greater integration of materials science, process engineering, pharmacology, toxicology, and regulatory considerations at the early development stage may further accelerate the translation of promising ESNF technologies.

### 5.5 Overall perspective

Taken together, recent advances demonstrate that the future of functionalized ESNFs will depend not only on the ability to introduce new functional agents or construct increasingly sophisticated architectures, but also on the ability to translate these innovations into reproducible, scalable, safe, and clinically meaningful products. The capability of ES to construct monolithic, core–sheath, hollow, Janus, multi-chamber, and hybrid architectures provides a powerful foundation for designing nanofibrous systems with precisely programmed functions. However, the most successful candidates for clinical translation are likely to be those in which added architectural or functional complexity produces a clear therapeutic advantage without introducing disproportionate manufacturing, safety, degradation, durability, environmental, or regulatory burdens.

Accordingly, future progress should focus on high-throughput and bulk production, reproducible and sustainable manufacturing, clinically acceptable materials and functionalization strategies, standardized critical quality attributes, long-term safety assessment, and regulatory-aware design**.** Integration of ES with established manufacturing technologies may further facilitate the transition from laboratory-scale fabrication to clinically and industrially relevant production. At the same time, advanced architectures such as core–sheath, Janus, hollow, and multi-chamber nanofibers should be developed according to application-specific needs rather than structural complexity alone. Ultimately, we envision the field moving toward optimized rather than maximal functionalization and structural complexity, in which each additional functional component or architectural feature is justified by a measurable therapeutic benefit and balanced against its effects on mechanical stability, degradation, long-term durability, manufacturing, sustainability, safety, and regulatory feasibility. This balance between functional sophistication and translational simplicity will be critical for converting promising ESNF concepts into clinically relevant and scalable products.

## 6 Conclusions

This review provides an in-depth analysis of recent developments in functionalized ESNFs and their expanding roles across diverse biomedical applications. Emphasis was placed on innovative modification strategies and contemporary techniques to enhance the structural and functional attributes of these nanofibrous scaffolds. Given their unique features, ESNFs demonstrate remarkable appeal across fields such as tissue regeneration, wound healing, targeted DD, and biosensing. Their tunable physicochemical properties and design flexibility further underscore their appeal as multifunctional platforms for both fundamental research and translational biomedical technologies. Nevertheless, fabricating NFs with precisely controlled morphology, superior mechanical and biochemical properties, and high production yield remains a technical challenge. Achieving these goals requires advanced control over the ES process and post-processing functionalization techniques. The use of various functionalization methods, such as plasma treatment, UV irradiation, chemical and biological crosslinking, and LBL deposition, has enabled researchers to tailor the surface chemistry and physico-mechanical properties of NFs to meet specific biomedical requirements. These functionalized NFs can be strategically engineered to direct specific cellular behaviors, including adhesion, proliferation, migration, and differentiation, thereby serving as potent platforms for targeted biomedical interventions. A particularly promising area of application is TE, where NF-based scaffolds are constructed to mimic the architecture and biomechanical characteristics of natural extracellular matrices. These scaffolds support tissue organization in vitro and offer the possibility for in vivo transplantation. ESNFs have also demonstrated potential in facilitating nerve regeneration, bone repair, and VTE and STE. Yet, a key obstacle lies in the tendency of some natural polymer NFs to degrade or lose functional integrity when exposed to physiological conditions, thereby limiting their long-term performance and biocompatibility. As such, identifying novel materials or combining synthetic and natural polymers may offer more reliable alternatives. Despite these advances, significant gaps remain. Most studies to date have used small-animal models, such as rodents, which may not adequately reflect human physiological and immunological conditions. Therefore, validating these findings in larger, clinically relevant animal models is critical before transitioning toward human trials. Furthermore, long-term studies are needed to evaluate the in vivo stability, degradation profiles, immune responses, and therapeutic outcomes of these scaffolds.

## CRediT authorship contribution statement

**Kalim Deshmukh:** Writing – review & editing, Writing – original draft, Visualization, Validation, Project administration, Funding acquisition, Conceptualization. **Mayank Pandey:** Writing – review & editing, Writing – original draft, Project administration, Formal analysis, Data curation. **Hichem Moulahoum:** Writing – review & editing, Validation, Software, Resources. **Faezeh Ghorbanizamani:** Writing – review & editing, Validation, Software, Formal analysis. **Tomáš Kovářík:** Writing – review & editing, Validation, Resources, Project administration, Funding acquisition. **Agata Przekora-Kuśmierz:** Writing – review & editing, Validation, Resources, Formal analysis. **Hemalata Jena:** Writing – review & editing, Writing – original draft, Formal analysis, Data curation. **Miral Vora:** Writing – review & editing, Writing – original draft, Formal analysis, Data curation. **Gautam Patel:** Writing – review & editing, Validation, Supervision, Resources. **Priyabrat Pradhan:** Writing – review & editing, Writing – original draft, Validation, Formal analysis, Data curation.

## Data availability statement

The preprint copy of this article is available at: https://doi.org/10.5281/zenodo.20488276, and the dataset is available at: https://doi.org/10.5281/zenodo.20489438.

## Ethics approval and consent to participate

This review article does not require any ethical approval or allied consents for publication.

## Declaration of competing interest

The authors declare that they have no known competing financial interests or personal relationships that could have appeared to influence the work reported in this paper.
